# The Role of the Fused Ring in Bicyclic Triazolium Organocatalysts: Kinetic, X-ray, and DFT Insights

**DOI:** 10.1021/acs.joc.1c03073

**Published:** 2022-03-01

**Authors:** Jiayun Zhu, Inmaculada Moreno, Peter Quinn, Dmitry S. Yufit, Lijuan Song, Claire M. Young, Zhuan Duan, Andrew R. Tyler, Paul G. Waddell, Michael J. Hall, Michael R. Probert, Andrew D. Smith, AnnMarie C. O’Donoghue

**Affiliations:** † Department of Chemistry, Durham University, South Road, Durham DH1 3LE, U.K.; ‡ Dpto. de Química Física, Facultad de Ciencias y Tecnologías Químicas, Universidad de Castilla - La Mancha, Avda. Camilo José Cela s/N, 13071 Ciudad Real, Spain; § School of Science, Harbin Institute of Technology (Shenzhen), Shenzhen, 518055, China; ∥ EaStCHEM, School of Chemistry, University of St Andrews, North Haugh, St Andrews, Fife KY16 9ST, U.K.; ⊥ Chemistry, School of Natural and Environmental Sciences, Newcastle University, Newcastle upon Tyne NE1 7RU, U.K.

## Abstract

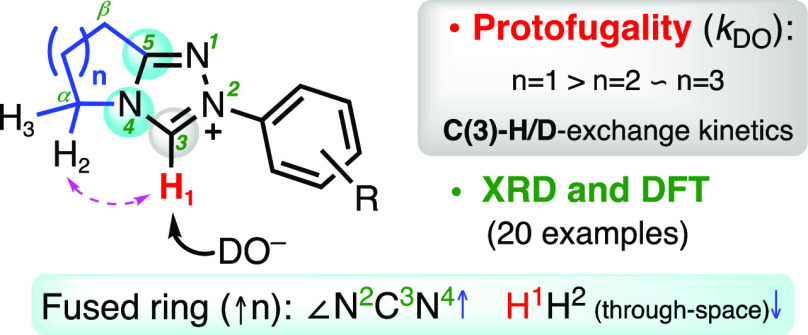

Bicyclic triazolium scaffolds are widely employed in *N*-heterocyclic carbene (NHC) organocatalysis. While the incorporation of a fused ring was initially for synthetic utility in accessing chiral, modular triazolyl scaffolds, recent results highlight the potential for impact upon reaction outcome with the underpinning origins unclear. The common first step to all triazolium-catalyzed transformations is C(3)-H deprotonation to form the triazolylidene NHC. Herein, we report an analysis of the impact of size of the fused (5-, 6-, and 7-membered, *n* = 1, 2, and 3, respectively) ring on the C(3) proton transfer reactions of a series of bicyclic triazolium salts. Rate constants for the deuteroxide-catalyzed C(3)-H/D-exchange of triazolium salts, *k*_DO_, were significantly influenced by the size of the adjacent fused ring, with the kinetic acidity trend, or protofugalities, following the order *k*_DO_ (*n* = 1) > *k*_DO_ (*n* = 2) ≈ *k*_DO_ (*n* = 3). Detailed analyses of X-ray diffraction (XRD) data for 20 triazolium salts (including 16 new structures) and of computational data for the corresponding triazolylidene NHCs provide insight on structural effects of alteration of fused ring size. In particular, changes in internal triazolyl NCN angle and positioning of the most proximal CH_2_ with variation in fused ring size are proposed to influence the experimental protofugality order.

## Introduction

**Figure 1:**
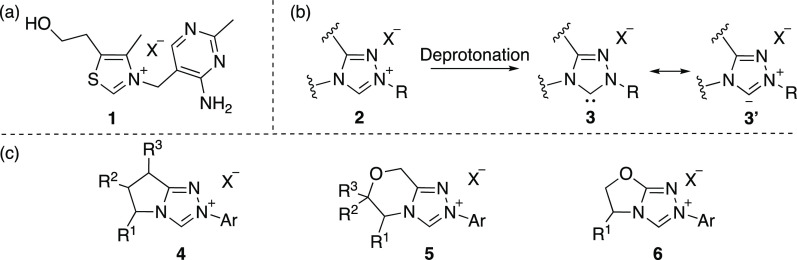
(a) Structure of thiamin **1** (vitamin B_1_); (b) C*-*(3) deprotonation of triazolium precatalyst **2**; (c) pyrrolidinone-, morpholinone-, and oxazolidinone-derived triazolium salts **4**–**6**.

Since Ukai first utilized naturally occurring thiazolium salt **1**, or vitamin B_1_, to catalyze the benzoin reaction of aldehydes,^1^ and later seminal studies by Breslow in establishing the catalytic mechanism,^2^ a broad range of related transformations catalyzed by different heterocyclic azolium salts continue to be investigated.^3,4^ As organocatalysts, azolium salts have been used to catalyze a diverse spread of reactions, including the benzoin and related acyloin reactions,^2,5^ Stetter reaction,^6^ cycloadditions,^7^ dearomatizations,^8^ among many others. Although a range of heterocyclic azolium classes have been employed in organocatalysis, triazolium precatalysts **2** are most often utilized^9^ (Figure 1). Typically, the reactions are initiated by a deprotonation step, which involves *in situ* conversion of the azolium salt **2** to the reactive *N*-heterocyclic carbene (NHC) **3**/ylide **3′**. In particular, chiral bicyclic triazolylidene scaffolds, initially developed by Knight and Leeper,^10^ have been widely investigated, with the development of modular syntheses allowing improved stereoselectivities.^3a^ A range of fused ring triazolium salt precatalysts have been employed, with those derived from pyrrolidinone,^11^ morpholinone,^12^ or oxazolidinone^13^ scaffolds (**4**–**6**, respectively) common.

**Figure 2:**
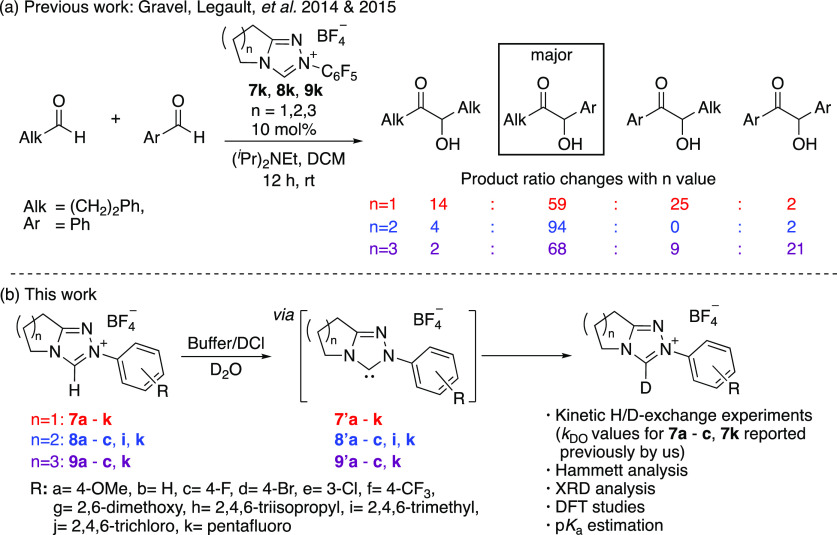
(a) Previous work: demonstration by Gravel et al. of catalyst control of product selectivity in the cross-benzoin reaction by variation of triazolium fused ring size. (b) This work.

Recent investigations have recognized that the role of the fused ring is not solely limited to providing a structural scaffold for chiral modification toward asymmetric catalysis. Gravel and co-workers reported that the chemoselectivity of the benzoin condensation could be influenced by a simple change in both the size and composition of the fused ring for bicyclic triazolium salts **7k**, **8k**, and **9k** (Figure 2a).^14^ Piperidinone-derived triazolium salt **8k** (*n* = 2) with a *N*-pentafluorophenyl substituent was shown to catalyze highly chemoselective cross-benzoin reactions between aliphatic and aromatic aldehydes. Although still yielding the same major product, analogous pyrrolidinone- and ε-caprolactam-derived triazolium salts, **7k** (*n* = 1) and **9k** (*n* = 3), showed significantly reduced chemoselectivities under these reaction conditions. This remains one of the few examples of a chemoselective cross-benzoin reaction that is catalyst- rather than substrate-controlled. Notably, selectivity and yield were shown to be invariant to either base or solvent choice. In a follow-on computational study supported by ^1^H NMR and crossover experiments, Gravel, Legault, and co-workers explored the origin of the chemoselectivity differences for **7k**, **8k**, and **9k**, although this study was not extended to triazolium salts with additional *N*-aryl substituents.^14b^ The superior selectivity observed for the piperidinone-derived catalyst **8k** was attributed to the kinetic control of reaction conditions. Furthermore, it was suggested that the steric interactions of proximal methylenes on the catalyst backbone with the aldehyde could play a significant role in obtaining chemoselectivity.

Despite the enormous current interest in NHC-organocatalysis, an understanding of chemoselectivity (as represented by the cross-benzoin reaction) remains one of the key unsolved challenges, and so the influence of the fused ring is potentially of importance across many transformations. As an initial deprotonation step is common to the majority of these transformations, we herein report an analysis of the impact of fused ring size on the C(3) proton transfer reactions of a series of triazolium salts (Figure 2b). Following on from our earlier studies,^15^ we undertook a hydrogen–deuterium exchange study to evaluate the effect of ring size on the kinetic acidities, or protofugalities,^16^ of the C(3)-hydrogens in a series of bicyclic triazolium ions. Experimental second-order rate constants for deuteroxide-catalyzed H/D-exchange, *k*_DO_ (M^–1^ s^–1^), could be used in Hammett structure–activity analysis in addition to providing estimates of C(3)-H p*K*_a_ values. We have performed X-ray diffraction and computational studies to afford additional insight. Despite the substantive literature focused on triazolium catalysis, there is no report to our knowledge comparing structural data for a large series of triazolium salts. We have analyzed experimental X-ray diffraction data for 20 triazolium salts (Figure 2b), which includes 16 previously unreported X-ray crystal structures. In addition, a DFT computational evaluation of the structures of these 20 triazolium ions and corresponding triazolylidenes has been performed. These data provide new insights into the role of fused ring size on the initial proton transfer step common to all triazolium-catalyzed transformations.

## Results and Discussion

### Hydrogen–Deuterium Exchange Kinetic Studies

Hydrogen–deuterium or hydrogen–tritium exchange has long been used as a method to estimate the kinetic lability of protons attached to carbon^17^ and to provide insight into factors influencing carbon acidity. For catalytic applications, the knowledge of rates of proton transfer between active species, and the corresponding activation barriers, is equally as important as p*K*_a_. In early studies, several groups reported rate constants for the base-catalyzed exchange of the C(2)-H of thiamin **1** providing initial evidence for the role of the conjugate base NHC/ylide as the active form of catalyst.^17c−17f,18^ The later isolation and structural characterization of a range of NHCs by Bertrand and Arduengo,^19^ including thiazolylidenes^20^ and triazolylidenes,^21^ provided further support for their relatively long lifetimes versus many other carbene classes. We have previously reported H/D-exchange kinetic studies for a range of conjugate acids of NHCs/ylides.^15a−15d^ This included rate-p*D* profiles for the C(3)-H/D exchange of pyrrolidinone-derived triazolium salts **7a–c,i,k** with 5-membered fused rings.^15a−15c^

**Figure 3:**
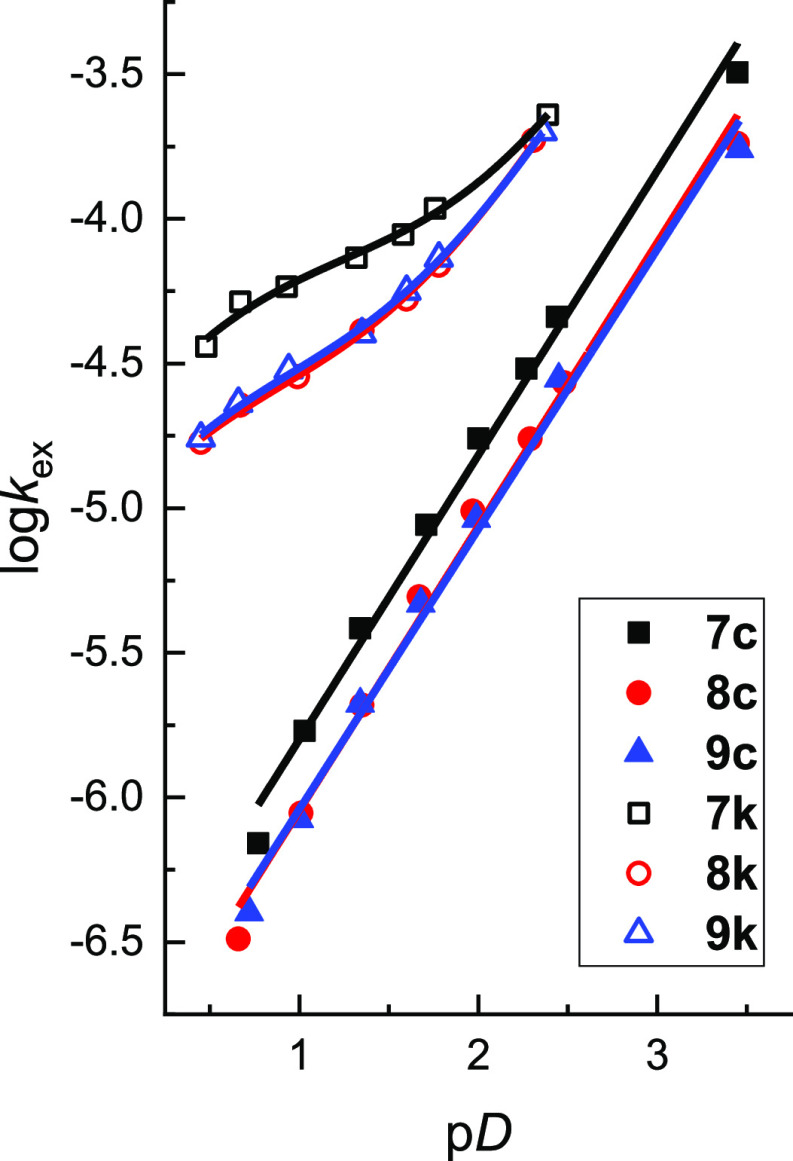
Comparison of p*D* rate profiles of C(3)-H/D exchange for triazolium salts **7**–**9c** and **7**–**9k** in D_2_O solution at 25 °C.

**Scheme 1:**
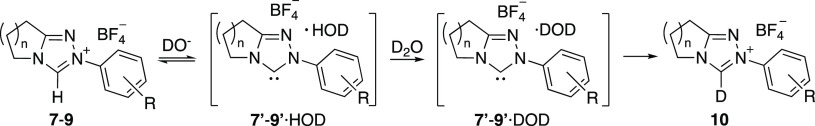
Mechanism for C(3)-H/D Exchange for Triazolium Salts **7**–**9**

In order to analyze the effect of ring size on proton transfer, analogous H/D-exchange kinetic studies have been performed for piperidinone-derived triazolium salts **8a–c,k** (with 6-membered fused rings) and for ε-caprolactam-derived triazolium salts **9a–c,k** (with 7-membered fused rings). To ensure consistency and accuracy, hydrogen–deuterium exchange experiments for **7a–c,k** are repeated herein and showed excellent agreement with our earlier data.^22^ The kinetic methods utilized (section S1.4, Supporting Information) were identical to those employed in our previous work. First-order rate constants for H/D-exchange were determined in aqueous solution at a range of p*D* values with ionic strength *I* = 1.0 (KCl) at 25 °C and with tetramethylammonium deuteriosulfate as internal standard. Using ^1^H NMR spectroscopy, the decrease in the area of the C(3)-proton of each substrate could be quantified over time. Figures S1–S12 include representative spectral overlays of reaction progress for **7a–c,k**, **8a–c,k**, and **9a–c,k**. The reactions were analyzed in the p*D* range of 0.5–3.5, because outside of this range the exchange reactions were too fast (p*D* > 3.5) or slow (p*D* < 0.5) for NMR kinetic analysis. During these experiments there was no decrease of other NMR resonances or appearance of additional signals, confirming that no side reactions, such as hydrolysis or decomposition of triazolium salt substrates, were occurring under these conditions. The observed pseudo first-order rate constants for deuterium exchange of the C(3)-proton, *k*_ex_ (s^–1^), were obtained from the slopes of semilogarithmic plots (Figures S13–S24) of reaction progress against time according to eq 1, where *f*(*s*) is the value of the fraction of unexchanged substrate. Tables S1–S12 summarize all first-order rate constants, *k*_ex_ (s^–1^), as a function of p*D*, and Figures S25–S28 include the corresponding log *k*_ex_–p*D* profiles. No corrections were applied for data acquired in buffer solution, because in all previous studies no evidence of buffer catalysis was observed.
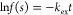
1
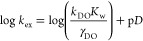
2
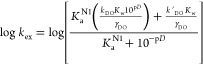
3Figure 3 compares the log *k*_ex_–p*D* profiles for the deuterium exchange reactions of **7c**, **8c**, and **9c** with those for **7k**, **8k**, and **9k**. For *N*-4-fluorophenyl triazolium salts **7c**, **8c**, and **9c**, good linear fits of log *k*_ex_–p*D* data to eq 2 were observed; in eq 2, *k*_DO_ (M^–1^ s^–1^) is the second-order rate constant for deuteroxide-catalyzed exchange, *K*_w_ the ion product of D_2_O at 25 °C,^23^ and γ_DO_ the activity coefficient for deuteroxide ion under our experimental conditions. Data for all other triazolium salts studied herein also showed good linear fits to eq 2 (Figures S25–S27) with the exception of **7k**, **8k**, and **9k**. This increase in log *k*_ex_ with p*D*, and first-order dependence on deuteroxide ion, is consistent with a single mechanism for deuteroxide-catalyzed deuterium exchange as shown in Scheme 1. In this mechanism, deprotonation of the triazolium salts **7**–**9** by deuteroxide results in the formation of a complex between NHCs **7′**–**9′** and a molecule of HOD (in Scheme 1, the ylidic resonance structures of NHCs **7′–9′** have been excluded for clarity). Subsequent reorganization of **7′**–**9′·**HOD to **7′**–**9′·**DOL (L = H or D) to allow for delivery of deuterium, followed by deuteration, leads to exchange product **10**.^15a,15d,24^ Owing to the large excess of bulk solvent D_2_O over substrate, the deuteration step is effectively irreversible; thus, *k*_ex_ reflects rate-limiting formation of solvent-equilibrated NHC from the triazolium salt and deuteroxide ion.

As a result of a change from a simple first-order dependence on deuteroxide ion at lower p*D* values, and hence from a slope of unity, log *k*_ex_–p*D* data for *N*-pentafluorophenyl salts **7k**, **8k**, and **9k** instead show excellent fits to eq 3. The altered dependence of log *k*_ex_ on p*D* is consistent with the onset of alternative pathways for deuterium exchange, which we have discussed in detail previously.^15a,15b,15f^ The most likely mechanistic explanation is a pathway via N(1)-deuteration at lower p*D* values allowing for hydrogen–deuterium exchange of the N(1)-deuterated dicationic triazolium salt (Scheme S2, section S1.4.4.4, Supporting Information). The additional terms in eq 3 (versus eq 2) include *k*′_DO_ (M^–1^ s^–1^), the second-order rate constant for deuteroxide-catalyzed C(3)-H/D exchange of the dicationic salt, and *K*_a_^N1^, the equilibrium constant of N(1)-protonation. The extent of dominance of this alternative pathway at lower p*D*s is dependent on p*K*_a_^N1^. The similar overall behavior observed for **7k**, **8k**, and **9k** suggests that the biggest influence on the onset of alternative H/D-exchange mechanisms is the *N-*aryl substituent rather than the size of the fused ring within the triazolium salt.

Table 1 summarizes values of the second-order rate constant for deuteroxide-catalyzed exchange, *k*_DO_ (M ^–1^s^–1^), for all triazolium salts studied herein. By definition, the second-order rate constant, *k*_DO_ (M^–1^ s^–1^), is the observed *k*_ex_ value in 1 M DO^–^ solution (p*D* ≈ 14). Experimentally, hydrogen–deuterium exchange for all triazolium ions is many orders of magnitude too fast to directly monitor in 1 M DO^–^ (half-lives ≈ nanoseconds); thus, *k*_DO_ values are obtained by assessment of a range of *k*_ex_ values at lower p*D*’s as described above. The comparison of reactivities toward deprotonation by a common base, *k*_DO_, allows for comparison of the kinetic acidities, or protofugalities,^16^ of the C(3)-hydrogens in the series of bicyclic triazolium ions. As DO^–^ is the relevant conjugate base of water and of similar basicity to widely used alkoxide ions, and with an increasing general focus on organocatalysis in more sustainable solvent media such as water,^25^ it is an appropriate choice as a reference base.

**Table 1:**
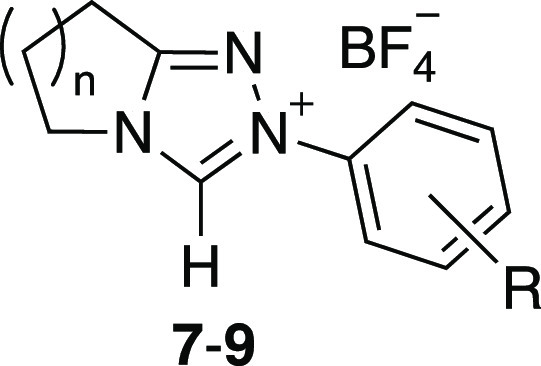
Summary of Second-Order Rate Constants for C(3)-H/D Exchange (*k*_DO_) for **7a–c,k** (*n* = 1); **8a–c,k** (*n* = 2); **9a**–**c**, **9k** (*n* = 3) and Calculated C(3)-H p*K*_a_ Values

triazolium salt	*n*	R =	*k*_DO_ (M^–1^ s^–1^)^a^	*k*_DO_^rel^^d^	p*K*_a_^e^
**7a**	1	4-OMe	4.55 (±0.36) × 10^7^	2.26	17.7
**7a**			4.20 (±0.23) × 10^7^^b^	2.09	17.8
**8a**	2		2.11 (±0.18) × 10^7^	1.05	17.9
**9a**	3		2.01 (±0.20) × 10^7^	1	18.0
**7b**	1	H	6.99 (±0.18) × 10^7^	2.12	17.5
**7b**			6.82 (±0.13) × 10^7^^b^	2.07	17.5
**8b**	2		3.43 (±0.15) × 10^7^	1.04	17.8
**9b**	3		3.29 (±0.15) × 10^7^	1	17.8
**7c**	1	4-F	8.97 (±0.27) × 10^7^	2.04	17.4
**7c**			8.66 (±0.11) × 10^7^^b^	1.97	17.4
**8c**	2		5.00 (±0.31) × 10^7^	1.14	17.7
**9c**	3		4.39 (±0.16) × 10^7^	1	17.7
**7k**	1	–F_5_	3.52 (±0.37) × 10^8^^c^	0.87	16.8
**7k**			6.82 (±0.25) × 10^8^^b^,^c^	1.69	16.5
**8k**	2		4.16 (±0.19) × 10^8^	1.03	16.7
**9k**	3		4.04 (±0.19) × 10^8^	1	16.7

a This work.

b Values of *k*_DO_ (M^–1^ s^–1^) obtained previously.

c The error quoted for **7k** is that obtained based on the overall fit to eq 3 for all of the data. The larger variance from our previous value for **7k** is due to the unavoidably small number of data points in the region of unit slope. The *k*_DO_ value obtained previously is deemed more reliable owing to the lower overall error in fitting to eq 3 as a result of having a greater number of data points.

d Calculated as *k*_DO_^rel^ = *k*_DO_^*n*^/*k*_DO_^*n*=3^.

e p*K*_a_ values calculated using experimental *k*_DO_ values (*vide infra*).

Obeying the common trend observed previously,^15a,15c,15d^ electron-withdrawing *N*-aryl substituents increase *k*_DO_, regardless of the size of the fused ring, with a span of ∼26-fold across the series in Table 1. Thus, triazolium salts **7k**, **8k**, and **9k** bearing the strongly electron-withdrawing *N*-pentafluorophenyl substituent have the highest *k*_DO_ values, whereas analogues **7a**, **8a**, and **9a** with the electron-donating *N*-4-methoxyphenyl substituent have the lowest protofugalities. Electron-withdrawing *N*-aryl substituents destabilize the cationic triazolium carbon acid **7**–**9** relative to the formally neutral NHC conjugate base **7′**–**9′** (Scheme 1), thus favoring the deprotonation process.

Table 1 also includes *k*_rel_ (= *k*_DO_^*n*^/*k*_DO_^*n*=3^) values, which allow for a comparison of the effect of the fused ring for a given *N*-aryl substituent. Notably, despite the distance from *C*(3)-H, the size of the fused ring clearly alters the protofugalities of the triazolium salts. In all cases, the 5-membered ring fused triazolium salts **7** have the highest rate constants for deuteroxide-catalyzed exchange, while the 6- and 7-membered ring fused salts **8** and **9** have lower values (*n* = 1 > *n* = 2 ≈ *n* = 3). The differences observed are relatively small (*k*_rel_ ≤ 2-fold); however, they are substantially outside experimental error, with added confidence provided from the observation of the same trend for different *N*-aryl substituents. In addition, the first-order rate constants for H/D-exchange (*k*_ex_), which are used to calculate *k*_DO_, are consistently higher for triazolium salts **7** compared to **8** and **9** (see log *k*_ex_–p*D* profiles Figures S25–S28).

Figure S29 includes a Hammett analysis of protofugalities (*k*_DO_) as a function of fused ring size. In the case of the *N*-pentafluorophenyl substituent, a σ substituent constant calculated by Taft^26^ is utilized. Hammett ρ values, obtained as slopes of the correlations in Figure S29, are positive for the three series as expected for a process favored by electron-withdrawing substituents. The Hammett substituent dependencies demonstrated by 5-, 6-, and 7-membered fused rings are closely similar in magnitude (ρ = 0.65^*n*=1^, ρ = 0.65^*n*=2^, 0.67^*n*=3^). Notably, these experimental ρ values are all less than unity, indicating a similar but smaller *N*-aryl substituent dependence than for the reference acid dissociation of benzoic acids despite having the same three bond distance from the acidic hydrogen to the *ipso*-aryl carbon. For **7**–**9**, the inherent stabilization provided by the three nitrogen atoms within the central NHC heterocycle could decrease the dependence on the nature of the *N*-aryl substituent. Furthermore, the closely similar experimental ρ values suggest that the *N*-aryl substituent effect is independent of the fused ring.

### X-ray Crystallographic Studies

**Figure 4:**
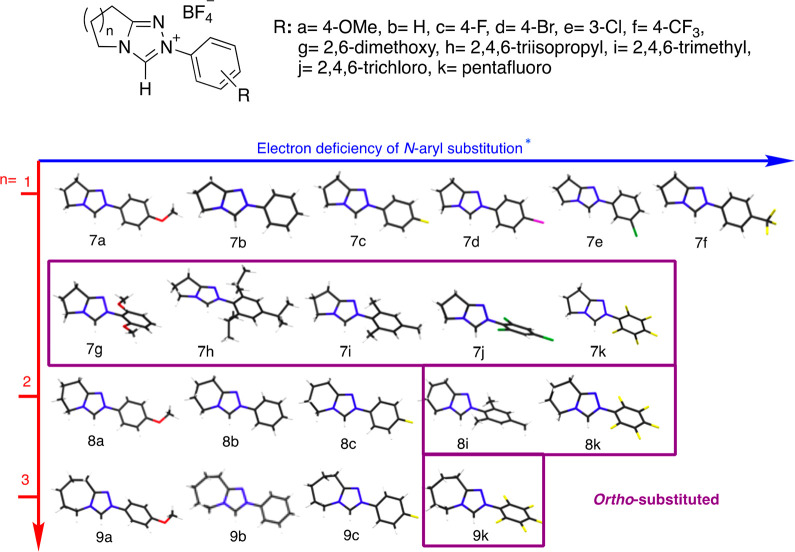
Summary of the single-crystal X-ray structures of 20 triazolium salts determined herein. (*Electron deficiency ordering based on Hammett substituent parameters; Table S13.)

During the syntheses of bicyclic triazolium salts for our kinetic studies, we determined 20 single-crystal X-ray structures (Figure 4), which includes 16 previously unreported structures (section S1.6, Supporting Information, and CCDC 2124937–2124950; 2124952–2124958). The crystals of triazolium salts **7a–g, 7i–k, 8a–c, 8i**, and **9a–b, 9k** were obtained by slow evaporation of methanol. The growth of single crystals of **7h**, **8k**, and **9c** suitable for single-crystal X-ray diffraction analysis was performed via a modified high-throughput encapsulated nanodroplet crystallization (ENaCt) approach (section S1.6.1).^27^

The X-ray crystallographic data was analyzed for structural insight on the role of the size of the fused ring and potential structure–kinetic activity trends. The bond lengths and bond angles of individual triazolium salt core structures are summarized in Tables S17 and S18. In general, triazolium salts with the same fused ring size were found to result in similar core structural parameters irrespective of the *N*-aryl substituent. Within each series (*n* = 1 (11 structures), *n* = 2 (5 structures), and *n* = 3 (4 structures)) the average bond angles and bond lengths of triazolium salts were calculated and are summarized in Tables 2 and S19. The differences in average angles and distances between series (*n* = 1 vs *n* = 2 and *n* = 2 vs *n* = 3) are also included in Table 2.

**Table 2:**
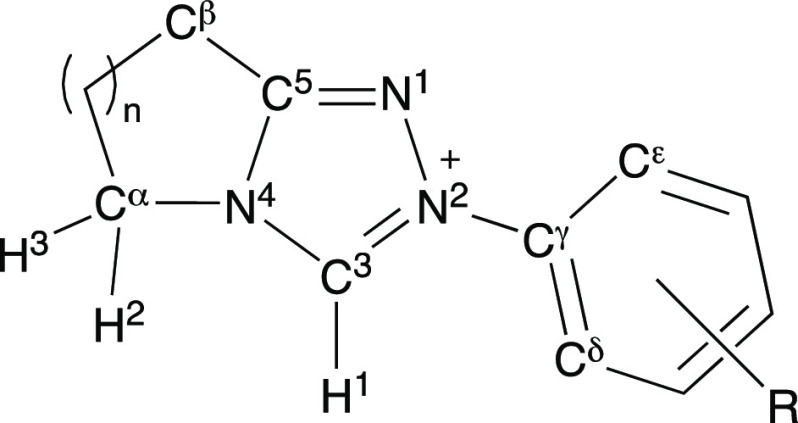
Summary of Average Bond Angles and Distances of Triazolium Salts **7a**–**k** (*n* = 1); **8a–c, 8i**, **8k** (*n* = 2); **9a**–**c**, **9k** (*n* = 3) Obtained from Single-Crystal X-ray Structural Analysis

	triazolium salts: average angles and distances^b^					
		*n* = 1	*n* = 2	*n* = 3	*n* = 1 vs *n* = 2^c^	*n* = 2 vs *n* = 3^d^
bond angles (deg)^a^	C^β^C^5^N^4^	110.6 [0.5]	122.2 [0.5]	124.2 [0.5]	11.6	2.0
	C^5^N^4^C^α^	113.7 [0.4]	125.6 [0.7]	127.6 [0.3]	11.8	2.0
	N^4^C^5^N^1^	111.9 [0.2]	111.3 [0.6]	110.7 [0.2]	–0.6	–0.6
	C^3^N^4^C^5^	107.4 [0.2]	106.9 [0.5]	106.9 [0.3]	–0.5	0.0
	N^1^N^2^C^3^	111.9 [0.4]	111.7 [0.4]	110.8 [0.7]	–0.2	–0.9
	N^2^C^3^N^4^	106.1 [0.3]	106.8 [0.3]	107.4 [0.3]	0.8	0.6
	C^5^N^1^N^2^	102.8 [0.3]	103.3 [0.1]	104.1 [0.4]	0.6	0.7
distance (Å)	H^1^H^2^^e^	3.06 [0.04]	2.76 [0.02]	2.49 [0.03]	–0.31	–0.26
	H^1^H^3^^e^	3.29 [0.05]	3.09 [0.03]	3.37 [0.05]	–0.19	0.28
torsion angle (deg)	H^1^C^3^C^α^H^2^^e^	43.6 [1.9]	38.2 [2.3]	1.7 [0.9]	–5.4	–36.5
	H^1^C^3^C^α^H^3^^e^	69.6 [1.6]	70.9 [2.2]	105.3 [1.6]	1.3	34.4

a Average of bond angle values obtained by X-ray diffraction measurement for triazolium salts **7a**–**k** (*n* = 1); **8a–c, 8i**, **8k** (*n* = 2); **9–c**, **9k** (*n* = 3).

b Standard deviation of average values is shown in square brackets.

c Difference = Average(*n* = 2) – Average(*n* = 1).

d Difference = Average(*n* = 3) – Average(*n* = 2).

e The C^α^ hydrogens with the shorter and longer through-space distances from H^1^ are labeled as H^2^ and H^3^, respectively.

**Figure 5:**
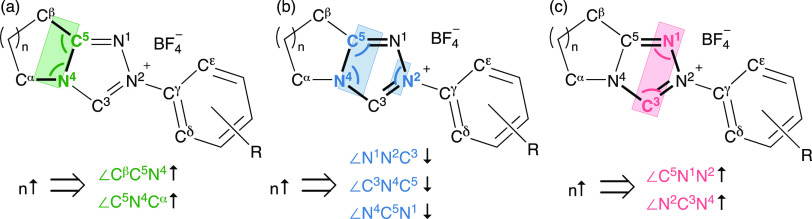
Bond angles (a) C^β^C^5^N^4^ and C^5^N^4^C^α^ increase; (b) N^1^N^2^C^3^, C^3^N^4^C^5^, and N^4^C^5^N^1^ decrease; and (c) C^5^N^1^N^2^ and N^2^C^3^N^4^ increase as *n* increases.

Focusing first on average bond angles in the triazolyl and fused rings, it can be observed that significant changes in bond angles occur between the 5-, 6-, and 7-membered ring series (Table 2 and Figure 5). With expansion of the size of the fused ring (from 5- through to 6- and 7-membered ring series), bond angles at the fusion point within the fused ring increase, and this impacts the bond angles in the adjacent triazolyl ring. Changing from 5- to 6-membered rings (*n* = 1 to 2) results in larger increases for the C^β^C^5^N^4^ and C^5^N^4^C^α^ average angles by 11.6° and 11.8°, with smaller further increases of 2.0° for both angles upon changing from 6- to 7-membered rings (*n* = 2 to *n* = 3, Figure 5a). As a result, three of the five angles of the central triazolyl ring decrease (C^3^N^4^C^5^, N^4^C^5^N^1^, and N^1^N^2^C^3^; Figure 5b), while the remaining two angles increase (N^2^C^3^N^4^ and C^5^N^1^N^2^; Figure 5c) owing to the increasing fused ring size.

The observed angular changes at the fusion point within the fused ring upon moving from the 5- to 6-membered ring series are substantially larger (∼11–12° from *n* = 1 to *n* = 2) than between the 6- and 7-membered ring series (∼2° from *n* = 2 to *n* = 3), which parallels the observed effects of ring size on protofugality. Irrespective of *N-*aryl substituent, 5-membered ring fused triazolium salts **7** have the highest *k*_DO_ values, while 6- and 7-fused salts **8** and **9** have smaller but similar values (*n* = 1 > *n* = 2 ≈ *n* = 3). The angular changes within the triazolyl ring appear more similar with increasing fused ring size; however, its significance cannot be unequivocally ascertained owing to the smaller changes involved. Of most relevance to the H/D exchange studies herein, there is a small but significant increase in the N^2^C^3^N^4^ angle at the acidic C(3)-H position from *n* = 1 to *n* = 3 in all cases. This ranged from an increase of 1.8° for **7–9a**, 1.8° for **7–9b**, 1.3° for **7–9c**, and 1.0° for **7–9k**.

We also analyzed the through-space distances from the C(3)-hydrogen (H^1^) to the most proximal methylene hydrogens (C^α^-H^2^ and H^3^) with changing fused ring size, but note the caveat of a greater uncertainty in hydrogen atom positioning from standard X-ray diffraction analysis. The through-space distances are reproduced in our DFT analysis of all the triazolium ions (*vide infra*), giving confidence in these values. The C^α^ hydrogens with the shorter and longer distances from H^1^ are labeled as H^2^ and H^3^, respectively (Table 2). These H^1^H^2^ and H^1^H^3^ distances are used as one measure of differences in the magnitude of the steric effect of the proximal methylene group as a function of changing fused ring size. The average H^1^H^2^ distance decreases from 3.06 to 2.49 Å with expansion of the size of the fused ring indicative of a corresponding increased steric effect of the methylene from 5- through to 6- and 7-membered ring series. This parallels the overall observed decrease in protofugality, and rate constants for deuteroxide-catalyzed C(3)-H/D exchange, with increasing fused ring size presumably owing to greater steric hindrance of the approach of DO^–^ to H^1^ when closer to H^2^.

The through-space distances of H^1^ from H^2^ and H^3^ are linked with changes in the torsion angles H^1^C^3^C^α^H^2^ and H^1^C^3^C^α^H^3^, respectively. Notably, C^α^H^2^ is almost coplanar with C^3^H^1^ when *n* = 3 as the average H^1^C^3^C^α^H^2^ dihedral angle is only 1.7°. By contrast, C^α^H^2^ is not coplanar with C^3^H^1^ when *n* = 1 and *n* = 2 with similar average H^1^C^3^C^α^H^2^ torsion angles of 43.6° and 38.2° for the 5- and 6-membered series. We postulate that the almost coplanar relationship of C^3^H^1^ and C^α^H^2^ (*n* = 3), in addition to the shorter through-space distance of H^1^ and H^2^ in this case, may contribute to an increased steric hindrance on the approach of the base and a resultant decrease in protofugalities. While these through-space distance and torsion angle trends align with the overall decrease in protofugalities from *n* = 1 to *n* = 3, clearly additional factors are needed to explain the observation of higher *k*_DO_ values for the 5-fused triazolium series with similar, smaller values for 6- and 7-fused salts **8** and **9** (*n* = 1 > *n* = 2 ≈ *n* = 3).

Finally, alteration of the triazolium *N-*aryl group changes the N^1^N^2^C^γ^C^δ^ dihedral angle in the solid-state structure (Tables S17 and S18). Typically, *N-*aryl groups without *ortho*-substituents are close to coplanar with the central triazolium ring and have N^1^N^2^C^γ^C^δ^ dihedral angles of ∼0°. Unsurprisingly, the introduction of bulky *ortho*-substituents on the *N-*aryl ring increases N^1^N^2^C^γ^C^δ^ with the largest dihedral angle of 91.2° observed for di-*ortho*-^*i*^propylphenyl-substituted **7h**. Alteration of the *N*-aryl group is external to the triazolium core structure and removed from the fused ring. Thus, changes in the N^1^N^2^C^γ^C^δ^ dihedral angle are mainly dictated by the *N*-aryl group rather than fused ring size (*n* = 1, 2, 3). This is consistent with the observation of closely similar ρ-values in our Hammett analysis of *k*_DO_ for **7**, **8**, and **9**, respectively.

### DFT Calculations

To further understand the key structural factors underpinning observed differences in protofugalities with fused ring size, the 20 triazolium salts **7**–**9** in Figure 2 and their corresponding carbenes **7′**–**9′**, were studied computationally (Supporting Information, section S2). The B3LYP and M062X functional with the polarized diffuse split-valence 6-311++g(d,p) basis set and the polarizable continuum model (PCM) with the solvent option of water were used in all cases.^28^ Several conformations of each molecule as starting geometries were optimized to find the most stable (lowest energy) geometry. The vibrational frequencies of these most stable geometries were then calculated at the same level of theory and revealed no imaginary frequencies, confirming them as true minima. The calculated bond angles and bond lengths of the core bicyclic structures of the 20 individual triazolium salts **7**–**9**, and corresponding carbenes **7′**–**9′**, are listed in Tables S21–S24 and S28–S31. The lowest-energy conformations calculated computationally for the triazolium ions **7**–**9** matched the solid-state experimental X-ray structures. Pleasingly, the trends in computed average angles and distances for the triazolium series **7**–**9** (Table S25 and S26) also match closely with the experimental values in Table 2. To our knowledge, while there is a published X-ray crystal structure for a 2,4,5-triphenyltriazolylidene,^29^ there are no reported experimental structural data for triazolylidenes **7′**–**9′**. The excellent computational reproduction of experimental trends for the fused ring effect in the triazolium series **7**–**9** gives confidence in a similar analysis of only computational data for the corresponding triazolyl NHCs **7′**–**9′**.

The average bond angles of triazolylidenes **7′**–**9′** obtained by calculation are summarized in Tables 3, S32, and S33. For a given size of the fused ring, bond angles within the heterocyclic ring are significantly influenced by conversion of the triazolium conjugate acid **7**–**9** to the corresponding triazolyl carbene **7′**–**9′**. The largest changes in average bond angle is for N^2^C^3^N^4^, which decreases by ∼6° upon formation of the carbene (Table 2 versus Table 3).

**Table 3:**
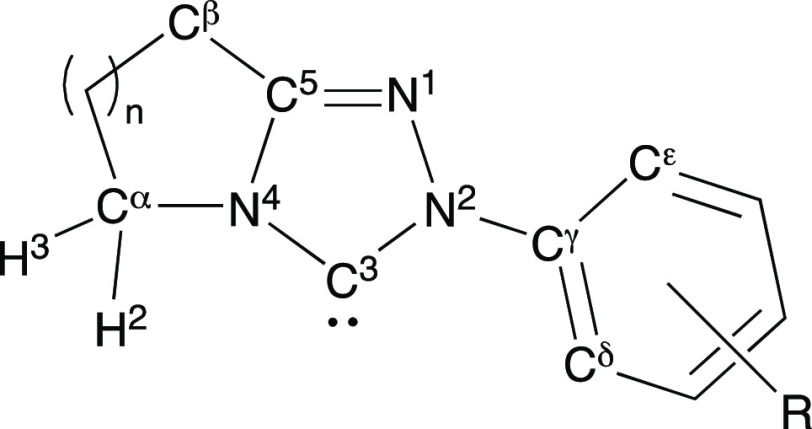
Summary of Average Bond Angles and Distances of Triazolylidenes **7′a–k** (*n* = 1); **8′a–c, 8′i**, **8′k** (*n* = 2); **9′a–c**, **9′k** (*n* = 3) Obtained from DFT Calculation (M062X)

	triazolyl carbene: calculated average angles and distances^b^					
		*n* = 1	*n* = 2	*n* = 3	*n* = 1 vs *n* = 2^c^	*n* = 2 vs *n* = 3^d^
bond angle (deg)^a^	C^β^C^5^N^4^	111.1 [0.0]	122.9 [0.0]	124.5 [0.1]	11.9	1.6
	C^5^N^4^C^α^	112.7 [0.1]	124.1 [0.1]	124.7 [0.1]	11.5	0.5
	N^4^C^5^N^1^	110.9 [0.1]	110.1 [0.1]	110.0 [0.1]	–0.8	–0.1
	C^3^N^4^C^5^	111.6 [0.1]	110.8 [0.2]	110.8 [0.1]	–0.8	0.0
	N^1^N^2^C^3^	115.9 [0.3]	115.4 [0.3]	115.3 [0.3]	–0.5	–0.1
	N^2^C^3^N^4^	99.6 [0.2]	100.5 [0.2]	100.6 [0.3]	0.9	0.1
	C^5^N^1^N^2^	102.1 [0.2]	103.2 [0.3]	103.3 [0.3]	1.1	0.2
distance (Å)	C^3^H^2^^e^	2.92 [0.00]	2.69 [0.00]	2.53 [0.00]	–0.22	–0.16
	C^3^H^3^^e^	3.05 [0.00]	2.91 [0.00]	3.18 [0.00]	–0.14	0.28
torsion angle (deg)	N^1^C^3^C^α^H^2^^e^	50.6 [0.1]	42.1 [0.3]	2.9 [0.3]	–8.5	–39.2
	N^1^C^3^C^α^H^3^^e^	85.4 [0.1]	82.6 [0.2]	121.5 [0.3]	–2.7	38.8

a Average of bond angle values obtained by DFT calculation (M062X) for triazolylidenes **7′a–k** (*n* = 1); **8′a–c, 8′i**, **8′k** (*n* = 2); **9′a–c**, **9′k** (*n* = 3).

b Standard deviation of average values is shown in square brackets.

c Difference = Average(*n* = 2) – Average(*n* = 1).

d Difference = Average(*n* = 3) – Average(*n* = 2).

e The C^α^ hydrogens with the shorter and longer distances from C^3^ are labeled as H^2^ and H^3^, respectively.

Closely similar effects are observed upon increase in fused ring size for the bicyclic triazolyl carbenes **7′**–**9′** as observed for the triazolium ions **7**–**9**. Changing from 5- to 6-membered fused ring series (*n* = 1 to 2) results in large increases in the average C^β^C^5^N^4^ and C^5^N^4^C^α^ angles of the triazolylidenes **7′**–**9′** (11.8° and 11.4°, respectively), with smaller further increases of 1.6° (C^β^C^5^N^4^) and 0.6° (C^5^N^4^C^α^) upon changing to 6- and 7-membered series (*n* = 2 to *n* = 3). Three of the five angles of the central triazolyl ring decrease (C^3^N^4^C^5^, N^4^C^5^N^1^, and N^1^N^2^C^3^), while the remaining two angles increase (N^2^C^3^N^4^ and C^5^N^1^N^2^) owing to the increasing fused ring size. From XRD data, the average N^2^C^3^N^4^ angle of triazolium salts **7** with *n* = 1 equals 106.1° (±0.3°), and this number increases to 106.8° (±0.3°) and 107.4° (±0.3°) with *n* = 2 and 3 for **8** and **9**. From computational data for the corresponding NHCs **7′**–**9′** there is a similar increase in the average N^2^C^3^N^4^ angle from 99.6° (±0.2°) for *n* = 1 to 100.5° (±0.2°) and 100.6° (±0.3°) for *n* = 2 and *n* = 3.

X-ray structural analysis allowed for an evaluation of the distance of the most proximal methylene hydrogens of the fused ring (C^α^-H^2^ and H^3^) from the C(3)-H^1^, and changes in the dihedral angles H^1^C^3^C^α^H^2^ and H^1^C^3^C^α^H^3^, with changing fused ring size in the triazolium series **7**–**9**. Similar conclusions can be drawn from an analysis of the computational data for the triazolylidenes **7′**–**9′** in this case represented by the through-space distances of H^2^ and H^3^ to the carbenic C(3)-center and torsional angles N^1^C^3^C^α^H^2^ and N^1^C^3^C^α^H^3^ (Table 3). Again, the methylene hydrogens H^2^ and H^3^ are closer to the carbenic position (C^3^) for the largest fused ring size (*n* = 3). Using these through-space distances and torsion angles as one measure of the steric effect of the most proximal methylene on the fused ring suggests that the steric impediment to approach of another reagent (e.g., DO^–^) to either the triazolium or carbene is largest when *n* = 3 and falls off for the smaller fused rings sizes (*n* = 2 ∼ *n* = 1).

As the structural and computational data discussed thus far relate only to the lowest-energy conformations of the triazolium ions and triazolylidenes, we have additionally evaluated the role of conformational flexibility in the fused ring through DFT modeling (section S2.5). By fixing the torsion angle between C^3^H^1^ and C^α^H^2^ for **7**–**9b** and between N^1^C^3^ and C^α^H^2^ for **7′**–**9′b**, the energy changes caused by conformation in the fused ring in the vicinity of the carbenic position could be evaluated (Tables S35–S40). The Boltzmann distribution of conformer mole fraction can then be obtained from the energy difference compared with the lowest-energy conformation (Figures S51 and S52). These plots clearly highlight the favored coplanar conformation of C^α^-H^2^ and the carbenic position for **9b** and **9′b** (*n* = 3), whereas for **7**/**7′b** (*n* = 1) and **8**/**8′b** (*n* = 2) the minimum energy preference is for a larger torsion angle of 35–42°. Despite having different minimum energy starting points, the energy-torsion angle profiles for all structures probed are essentially superimposable after applying starting point corrections (Figure S53).^30^

### NCN Angle Effects on Proton Transfer

**Figure 6:**
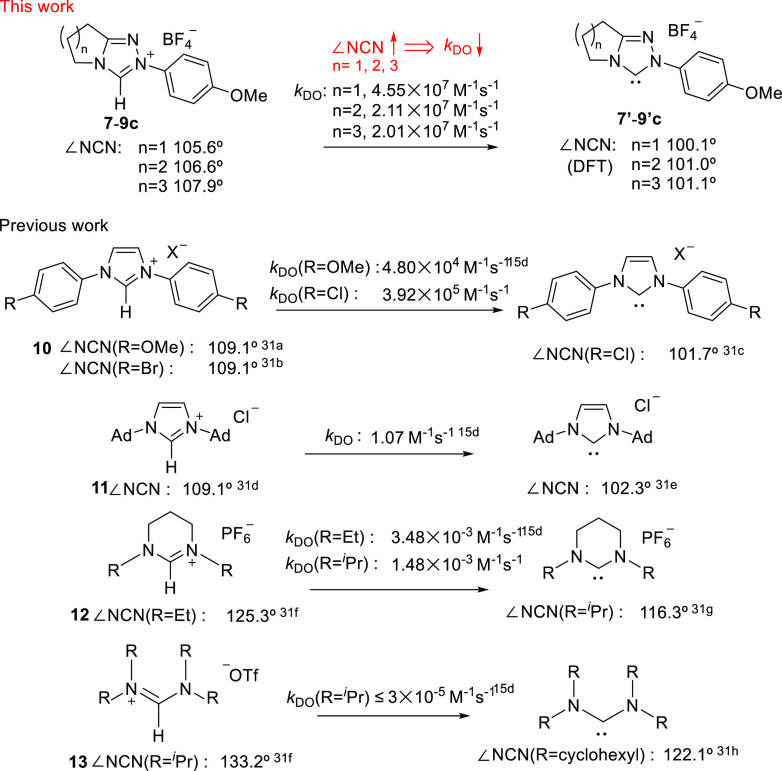
Decrease in protofugality (*k*_DO_) with increasing NCN angle.^15d,31^

**Figure 7:**
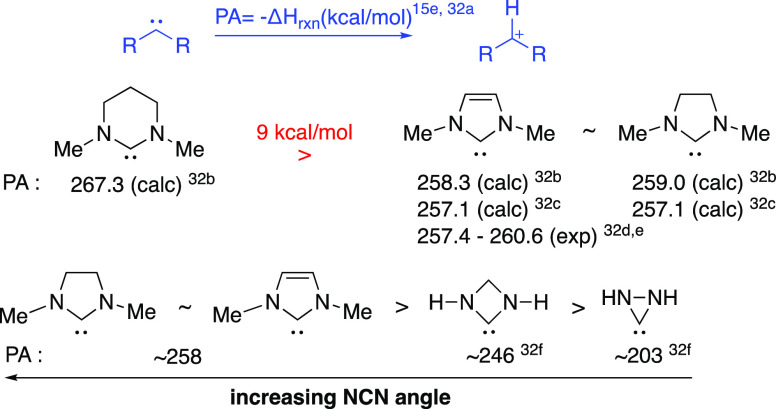
Increase in proton affinity (PA) with increasing NCN angle.^15e,32^

In our previous H/D-exchange studies we observed substantial decreases in *k*_DO_ with increase in the internal NCN angle of the NHC. For example, protofugalities for imidazolium ions **10** and **11**, which are the conjugate acids of imidazolyl NHCs, are 3–8 orders of magnitude higher than for tetrahydropyrimidinium ions **12**. In the imidazolyl case the internal NCN angles within the five-membered heterocyclic ring are substantially smaller than within the six-membered ring of **12** (Figure 6). Specifically, comparing two examples with *N-*alkyl substituents, **11** and **12**, for an increase in NCN angle of ∼13° there is a corresponding drop in *k*_DO_ of 720-fold. Similarly, comparing **12** and **13** with *N*-^*i*^Pr substituents, an increase in NCN angle of ∼7° is accompanied by a drop in *k*_DO_ by ∼50-fold. Although other factors, including the number and type of NHC heteroatoms, aromaticity, and *N*-substitution, influence protofugalities, the NCN angle changes are clearly also important. We previously argued that an enforced increase in internal NCN angle through alteration of heterocyclic ring size is better accommodated by the conjugate acid (azolium ion) than the conjugate base (NHC), thus contributing to a decrease in acidity.^15d^ This argument is supported by closely similar trends for carbene proton affinity (PA) measurements with higher PAs observed for NHCs with larger ring sizes (Figure 7).^15e^

Although substantially smaller in the present case, the increase in N^2^C^3^N^4^ bond angle (by ∼2°) at the acidic position with changing fused ring size from *n* = 1 to *n* = 3 also correlates with decreases in *k*_DO_ (*k*_DO_^rel^ ≤ 2). The increase in N^2^C^3^N^4^ angle with changing fused ring size is observed consistently for different *N-*aryl substituents, supporting the generality of this trend.

In general an increase in angle toward 180° at a carbene center is associated with an increase in p-character of the nonbonding orbitals bearing the negative charge. Similarly, in the present case the small increase in NCN bond angle moving from *n* = 1 to *n* = 3 could be qualitatively associated with a concomitant increase in p-character, or decreased s-character, of the hybrid orbital at the carbenic center.^33^ Parallels can be drawn with the common textbook explanation for the increase in carbon acidity along the alkane (*sp*^3^*C*-H, p*K*_a_ ∼ 45–70), alkene (*sp*^2^*C*-H, p*K*_a_ ∼ 40–45), and alkyne (*sp C*-H, p*K*_a_ ∼ 20–25) hydrocarbon series, where the negative charge formed upon deprotonation is more stable in an orbital of greater *s*-character.^34^ Using this argument, we can speculate that the higher protofugalities of the 5-fused triazolyl series might result from increased *s*-character at the C(3)-position stemming from smaller NCN angles relative to the 6- and 7-fused series.

### Estimation of Carbon Acid p*K*_a_ Values

There is an increasingly large body of work on the conjugate acid p*K*_a_’s of NHCs in a range of solvents, including water, acetonitrile, and DMSO, in addition to related gas-phase acidities and proton affinities.^15a−15d,24,32b,35^ Several approaches may be employed to access these carbon acid p*K*_a_ values, which we have reviewed previously.^15e^ In aqueous solution, the main difficulty associated with the *direct* determination of conjugate acid p*K*_a_ values of NHCs is the well-established leveling effect. Owing to the substantially higher basicities of most NHCs relative to the conjugate base of solvent (HO^–^), quantitative deprotonation of solvent occurs.

**Scheme 2:**
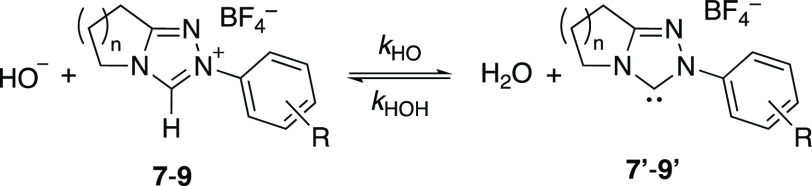
Equilibrium for Acid Dissociation of Triazolium Ions

In the determination of aqueous carbon acidities of weakly acidic species, one solution long-employed to circumvent this problem is the calculation of p*K*_a_ from the rate constants for the forward and reverse directions of the proton transfer equilibrium.^36^ We and others have previously employed this kinetic method for the determination of carbon acid p*K*_a_ values for deprotonation at C(3) for a series of triazolium salts including **7** using eq 4 derived for Scheme 2.^15a−15d,24^ In this equation, *k*_HO_ (M^–1^ s^–1^) is the second-order rate constant for deprotonation at C(3) by hydroxide ion, which may be calculated from the corresponding *k*_DO_ value using a value of *k*_DO_/*k*_HO_ = 2.4 for the secondary solvent isotope effect on the basicity of HO^–^ in H_2_O versus DO^–^ in D_2_O. As discussed previously,^15a,15d^ the absence of significant general base catalysis of deuterium exchange provides good evidence that the reverse protonation of the triazol-3-ylidene NHC by water is equal or close to the limiting rate constant for the physical process of dielectric relaxation of the solvent (*k*_HOH_ ≤ *k*_reorg_ = 10^11^ s^–1^). Rate constants of this magnitude are challenging to access experimentally, generally requiring femtosecond transient absorption spectroscopy; however, values for the dielectric relaxation of water have been determined reliably.^37^ Because the main error in p*K*_a_ determination using this method is associated with the value assumed for *k*_HOH_, these p*K*_a_ values provide upper limit estimations.
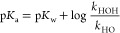
4

Using the *k*_DO_ values determined herein, and by application of eq 4, we have calculated p*K*_a_ values for triazolium salts **8a–c,k** with 6-fused rings and **9a–c,k** with 7-fused rings (Table 1). Owing to the ∼2-fold effect of fused ring size on *k*_DO_, and the logarithmic relationship to p*K*_a_ in eq 4, the resulting p*K*_a_ values for a given *N-*aryl substituent are closely similar. These triazolium p*K*_a_’s in the 16.7–18 range are ∼4 and 6 units below typical aqueous values for diaryl- and dialkylimidazolium ions, respectively, whereas only ∼1–2 units below typical *N-*alkylthiazolium p*K*_a_’s.^15a−15d,24^ The overall effect of the central ring heteroatoms on p*K*_a_ is larger than either *N*-aryl or fused ring substituent effects. The influence of fused ring size on kinetic acidities or protofugalities, however, is significantly larger than on p*K*_a_. Thus, rates and half-lives of NHC generation by C(3)-deprotonation of the conjugate acid, which is of direct relevance to catalysis, will be significantly influenced by the nature of the fused ring size in the bicyclic triazolium precursor.

## Conclusion

In conclusion, this work has probed the effect of the size of the fused rings (5-, 6-, and 7-membered; *n* = 1, 2, and 3 respectively) within a range of bicyclic triazolium salts on their kinetic acidities, or protofugalities. Rate constants for the deuteroxide-catalyzed H/D-exchange of triazolium salts, *k*_DO_, were observed to be significantly influenced by the size of the adjacent fused ring, with the trend following the order *k*_DO_(5-membered ring, *n* = 1) > *k*_DO_(6-membered ring, *n* = 2) ≈ *k*_DO_(7-membered ring, *n* = 3). Hammett ρ values, obtained as slopes of Hammett correlations of *k*_DO_ values with varying NHC *N*-aryl substituent, were positive for the three series (5-, 6-, and 7-membered rings, *n* = 1–3) consistent with a deprotonation process favored by electron-withdrawing substituents. Hammett *N*-aryl substituent dependencies were found to be closely similar (ρ = 0.65^*n*=1^, ρ = 0.64^*n*=2^, 0.67^*n*=3^). Detailed analyses of X-ray diffraction structural data and computational analysis for 20 triazolium salts/triazolylidenes provide insight on the structural effects of altering fused ring size, which could be aligned with observed effects on *k*_DO_. The most proximal methylene of the fused ring is closest and almost coplanar with the carbenic center when *n* = 3, indicative of a greater steric effect; this parallels our observation of a decrease in rates of deprotonation by DO^–^ when *n* = 3. Changing from 5- to 6-membered fused ring (*n* = 1 to 2) results in significant increases (by ∼11–12°) in the C^β^C^5^N^4^ and C^5^N^4^C^α^ angles within the fused ring, with smaller further increases of ∼2° upon changing from 6- to 7-membered ring (*n* = 2 to *n* = 3). A resultant change in internal angles in the adjacent NHC ring is also observed. In particular, the resulting ∼2° increase in internal NCN angle is consistent with a drop in acidity (lower *k*_DO_ values) with increased fused ring size. Finally, carbon acid p*K*_a_ values of the bicyclic triazolium salts were estimated using a kinetic method.

Given the prevalence of bicyclic structures in NHC organocatalytic scaffolds and their importance for many C–C bond-forming reactions, we hope this work provides quantitative and structural insight into the role of a simple incremental change in the number of methylenes in the fused ring. Clearly, for the proton transfer step between a triazolium salt and triazolyl carbene in aqueous solvent, the size of the fused ring size is important, with five membered fused rings (*n* = 1) giving significantly higher rates of deprotonation. Ongoing studies in our laboratories are focused on kinetic analysis of the role of the fused ring size in later reaction steps in triazolium-catalyzed benzoin and related acyloin reactions.

## Supporting Information Available

Synthetic procedures, ^1^H NMR and ^13^C spectral data, and HRMS data; kinetic procedures, fitting, and data; single-crystal X-ray diffraction data; computational details (PDF)

## Accession Codes

CCDC 2124937–2124950 and 2124952–2124958 contain the supplementary crystallographic data for this paper. These data can be obtained free of charge via www.ccdc.cam.ac.uk/data_request/cif, or by emailing data_request@ccdc.cam.ac.uk, or by contacting The Cambridge Crystallographic Data Centre, 12 Union Road, Cambridge CB2 1EZ, UK; fax: +44 1223 336033.
