# Supplementary material for: The Role of the Fused Ring in Bicyclic Triazolium Organocatalysts: Kinetic, X-ray, and DFT Insights
Source: J Org Chem. 2022 Mar 1;87(6):4241–53. doi: 10.1021/acs.joc.1c03073 (PMC8938951; doi:10.1021/acs.joc.1c03073)
Supplement: Supplementary file 1 — jo1c03073_si_001.pdf [file jo1c03073_si_001.pdf]

# Electronic Supporting Information

**The Role of the Fused Ring in Bicyclic Triazolium Organocatalysts: Kinetic, X-ray and DFT Insights**

Jiayun Zhu,[a] Inmaculada Moreno,[a,b] Peter Quinn,[a] Dmitry S. Yufit,[a] Lijuan Song,[c] Claire M. Young,[d] Zhuan Duan,[d] Andrew R. Tyler,[e] Paul G. Waddell,[e] Michael J. Hall,[e] Michael R. Probert,[e] Andrew D. Smith[d,]* and AnnMarie C. O'Donoghue[a,] *

[a]*Department of Chemistry, Durham University, South Road, Durham DH1 3LE, UK*

[b]*Dpto. de Química Física, Facultad de Ciencias y Tecnologías Químicas, Universidad de Castilla - La Mancha, Avda. Camilo José Cela s/N, 13071 Ciudad Real, Spain*

[c]*School of Science, Harbin Institute of Technology (Shenzhen), Shenzhen, 518055, China*

[d]*EaStCHEM, School of Chemistry, University of St Andrews, North Haugh, St Andrews, Fife, KY16 9ST, UK*

[e]*Chemistry, School of Natural and Environmental Sciences, Newcastle University, Newcastle upon Tyne, NE1 7RU, UK*

Corresponding Author Email Addresses: annmarie.odonoghue@durham.ac.uk; ads10@st-andrews.ac.uk

## Table of Contents

*S1. Experimental* .......... *3*

S1.1 General Instrumentation .......... 3

S1.2 Materials .......... 4

S1.3 Syntheses of Triazolium Tetrafluoroborate Salts .......... 4

S1.4 Deuterium Exchange and p$K_a$ Measurements .......... 9

S1.5 Hammett Analysis of Protofugalities ($k_{DO}$). .......... 39

S1.6 Single-crystal X-ray Crystallography .......... 41

S1.7 Synthetic $^1$H and $^{13}$C NMR Spectra for New Triazolium Salts .......... 63

S1.8 Synthetic $^1$H NMR Spectra for Known Triazolium Salts .......... 70

*S2. DFT Calculations* .......... *77*

S2.1 Conformation Search and Total Energies .......... 77

S2.2 Coordinates of Triazolium salts .......... 88

S2.3 Coordinates of Carbenes .......... 153

S2.4 Summary of Bond Lengths, Bond Angles, and Dihedral Angles from Computational Analysis .......... 216

S2.5 Computational Analysis of Conformational Changes in Fused Ring .......... 233

S2.6 NBO analysis .......... 240

*S3. References* .......... *241*

## S1. Experimental

### S1.1 General Instrumentation

**NMR**: NMR samples were prepared in deuterated chloroform and deuterium oxide. NMR spectra were recorded on Oxford Varian Unity Inova 300 and 500 MHz, Varian Unity 300 MHz, and Bruker Ultrashield 400 MHz NMR spectrometers. $^{1}$H and $^{13}$C NMR chemical shifts in $CDCl_3$ are reported relative to $CHCl_3$ at 7.27 ppm and 77.0 ppm respectively. In $D_2O$, $^{1}$H NMR chemical shifts are reported relative to HOD at 4.67 ppm. Coupling constants (J) are reported in Hz. Multiplicities are indicated by: br s (broad singlet), s (singlet), d (doublet), t (triplet), q (quartet) and m (multiplet).

**Mass spectrometry**: (*m/z*) data were acquired by electrospray ionisation (ESI). Low resolution ESI MS was carried out on a Waters Micromass ZQ4000 spectrometer and low resolution EI and CI MS was carried out on a Micromass Quattro II spectrometer. High resolution ESI and ESI MS was carried out on a Finnigan MAT 900 XLT or a Finnigan MAT 95 XP; a Thermofisher LTQ Orbitrap XL spectrometer was also used to obtain high resolution ESI MS for accurate mass determination but also provided fragmentation data for the characterisation of samples. Values are quoted as a ratio of mass to charge in Daltons.

**Melting points:** Melting points were determined using an electrothermal 9100 melting point apparatus.

## S1.2 Materials

Deuterium oxide-d$_2$ (99.9 atom % D), deuterium chloride (35 wt %, 99 atom % D) were purchased from Goss Scientific Instruments Ltd. Chloroform-d$_1$ (99.8 atom % D) was purchased from Apollo Scientific, and Euriso-top. Unless stated, all other chemicals were reagent grade and used without further purification. Reactions involving air or moisture sensitive reagents were performed under an argon atmosphere using oven-dried glassware. Solvents were dried prior to use using an Innovative Technology Inc. solvent purification system.

## S1.3 Syntheses of Triazolium Tetrafluoroborate Salts

Triazolium tetrafluoroborate salts **7a-d, 7g, 7i-k, 8b, 8i, 8k,** and **9b, 9k** were synthesized according to literature procedures, with all physical and spectroscopic data in agreement with the literature[S1].

Triazolium tetrafluoroborate salts **7e, 7f, 7h, 8a, 8c, 9a,** and **9c** were not reported previously; the syntheses of these compounds were adapted from the same literature procedures used above[S1], with their physical and spectroscopic data presented in the following sections.

**General procedure for the preparation of Triazolium tetrafluoroborate salts 7e, 7f, 7h, 8a, 8c, 9a, and 9c:**

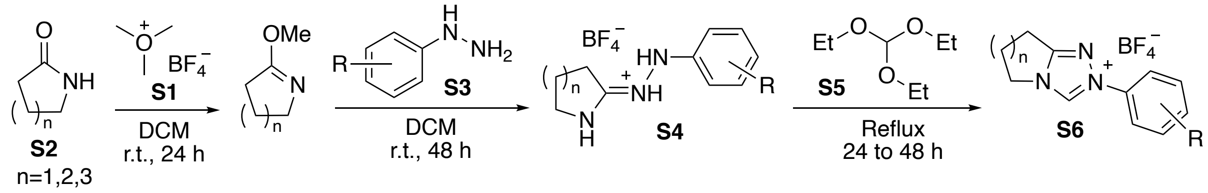


Scheme S1. General synthetic procedure for triazolium salts **7e**, **7f**, **7h**, **8a**, **8c**, **9a**, and **9c**.

Trimethyloxonium tetrafluoroborate (1.1 equiv; **S1**) was added to a solution of the relevant lactam (1 equiv; **S2** (n=1) for **7e**, **7f**, **7h**; **S2** (n=2) for **8a**, **8c**; **S2** (n=3) for **9a**, **9c**) in dichloromethane (30 mL per 1.00g lactam) and stirred for 24 h at r.t. under an inert atmosphere. The appropriate aryl hydrazine (1 equiv; **S3**) was added to the reaction mixture and stirred for a further 48 h at r.t. under an inert atmosphere to form the corresponding amidrazone (**S4**). The solvent was removed under reduced pressure, and the residue dissolved in triethylorthoformate (5 mL per 1 g; **S5**), and the reaction mixture was refluxed for 24 to 48 hours. Solvent was removed and the triazolium salts (**S6**) were purified by recrystallisation from DCM/Diethyl ether or MeOH/Diethyl ether.

X-ray diffraction structural analysis was performed for all 20 triazolium salts **7a-k**, **8a-c**, **j**, **k** and **9a-c**, **k** and is reported in Section S1.6. The crystal structures of **7a**, **7b**, **7g**, and **7k** were previously reported[S1g-h] but were re-determined by us for consistency in structural analysis across the full series.

## Synthesis of 2-(3-chlorophenyl)-6,7-dihydro-5*H*-pyrrolo[2,1-c]triazol-2-ium tetrafluoroborate 7e

Triazolium tetrafluoroborate **7e** was obtained as a pale yellow solid (3.83 g, 83 %); mp = 188-190 °C; $^{1}$H NMR (400 MHz, DMSO-$d_6$) δ 10.76 (s, 1H), 8.10 – 8.00 (m, 1H), 7.92 – 7.86 (m, 1H), 7.76 – 7.69 (m, 2H), 4.49 – 4.31 (m, 2H), 3.22 (dd, *J* = 8.2, 7.1 Hz, 2H), 2.84 – 2.66 (m, 2H).; $^{13}$C{1H} NMR (101 MHz, DMSO-$d_6$) δ 163.6, 139.5, 137.1, 134.8, 132.5, 130.8, 121.1, 119.9, 47.5, 27.1, 21.7.; HRMS (ESI) *m/z*: $[M–BF_4]^+$ Calculated for $C_{11}H_{11}N_3Cl$ 220.0642; Found 220.0638.

## Synthesis of 2-(4-trifluoromethylphenyl)-6,7-dihydro-5*H*-pyrrolo[2,1-c]triazol-2-ium tetrafluoroborate 7f

Triazolium tetrafluoroborate **7f** was obtained as a white solid (1.02 g, 72 %); mp = 216-218 °C; $^{1}$H NMR (400 MHz, DMSO-$d_6$) δ 10.84 (s, 1H), 8.28 – 7.99 (m, 4H), 4.44 (dd, J = 8.1, 6.6 Hz, 2H), 3.24 (dd, J = 8.2, 7.2 Hz, 2H), 2.83 – 2.70 (m, 2H). $^{13}$C{1H} NMR (101 MHz, DMSO) δ 163.8, 139.7, 138.9, 131.2, 130.9, 130.5, 130.2, 128.1, 128.1, 128.1, 128.0, 125.4, 122.7, 121.9, 47.6, 27.1, 21.7. HRMS (ES+): $[M–BF_4]^+$ $C_{12}H_{11}N_3F_3$ requires: 254.0905, found: 254.0903.

## Synthesis of 2-(2,4,6-triisopropylphenyl)-6,7-dihydro-5*H*-pyrrolo[2,1-c][1,2,4] triazol-2-ium chloride 7h

Triazolium tetrafluoroborate **7h** was obtained as a pale yellow powder (0.45 g, 29 %); mp = 142-145 °C; $^{1}$H NMR (700 MHz, DMSO-$d_6$) δ 10.37 (s, 1H), 7.27 (s, 2H), 4.44 (t, *J* = 7.4 Hz, 2H), 3.18 (t, *J* = 7.7 Hz, 2H), 2.98 (p, *J* = 6.9 Hz, 1H), 2.73 (p, *J* = 7.6 Hz, 2H), 2.37 (h, *J* =

6.7 Hz, 2H), 1.23 (d, $J$ = 6.9 Hz, 6H), 1.14 (d, $J$ = 6.8 Hz, 6H), 1.08 (d, $J$ = 6.8 Hz, 6H).; $^{13}C\{1H\}$ NMR (176 MHz, DMSO-$d_6$) δ 163.6, 153.1, 146.0, 142.2, 129.8, 122.6, 47.8, 34.2, 28.1, 26.7, 24.4, 24.2, 24.1, 21.9.; HRMS (ESI) *m/z*: $[M–BF_4]^+$ Calculated for $C_{20}H_{30}N_3$ 312.2440; Found 312.2433.

**Synthesis of 2-(4-methoxyphenyl)-5,6,7,8-tetrahydro-[1,2,4]triazolo[4,3-a]pyridin-2-ium tetrafluoroborate 8a**

Triazolium tetrafluoroborate **8a** was obtained as a white solid (1.05 g, 65%). mp = 190-192 °C; $^1H$ NMR (400 MHz, Chloroform-$d$) δ 10.07 (s, 1H), 7.82 – 7.74 (m, 2H), 7.06 – 6.99 (m, 2H), 4.55 (t, $J$ = 5.8 Hz, 2H), 3.88 (s, 3H), 3.13 (t, $J$ = 6.2 Hz, 2H), 2.20 – 2.06 (m, 4H).; $^{13}C\{1H\}$ NMR (101 MHz, Chloroform-$d$) δ 161.3, 152.9, 138.8, 126.9, 122.3, 115.3, 55.7, 46.2, 21.4, 21.2, 18.8.; *m/z* (ES+): 230 ($[M–BF_4]^+$, 100%). HRMS (ESI) *m/z*: $[M–BF_4]^+$ Calculated for $C_{13}H_{16}N_3O$ 230.1293; Found 230.1290.

**Synthesis of 2-(4-fluorophenyl)-5,6,7,8-tetrahydro-[1,2,4]triazolo[4,3-a]pyridin-2-ium tetrafluoroborate 8c**

Triazolium tetrafluoroborate **8c** was obtained as a white solid (0.90 g, 49%). mp = 108-109 °C; $^1H$ NMR (400 MHz, Chloroform-$d$) δ 9.99 (s, 1H), 7.89 – 7.76 (m, 2H), 7.26 – 7.13 (m, 2H), 4.48 (t, $J$ = 5.7 Hz, 2H), 3.10 (t, $J$ = 6.0 Hz, 2H), 2.11 (qd, $J$ = 6.8, 6.2, 3.8 Hz, 4H).; $^{13}C\{1H\}$ NMR (101 MHz, Chloroform-$d$) δ 164.6, 162.1, 153.2, 139.7, 131.3, 131.2, 123.2, 123.1, 117.2, 117.0, 46.1, 21.4, 21.0, 18.6.; *m/z* (ES+): 304 ($[M–BF_4]^+$, 100%). HRMS (ESI) *m/z*: $[M–BF_4]^+$ Calculated for $C_{12}H_{13}N_3F$ 218.1094: Found 218.1093.

## Synthesis of 2-(4-methoxyphenyl)-6,7,8,9-tetrahydro-5*H*-[1,2,4]triazolo[4,3-a]azepin-2-ium tetrafluoroborate 9a

Triazolium tetrafluoroborate **9a** was obtained as an off-white powder (0.73 g, 22%). mp = 148-152 °C; $^{1}$H NMR (400 MHz, Chloroform-*d*) δ 10.09 (s, 1H), 7.84 – 7.73 (m, 2H), 7.08 – 6.99 (m, 2H), 4.56 (s, 2H), 3.88 (s, 3H), 3.26 – 3.05 (m, 2H), 2.02 (s, 4H), 1.90 (s, 2H).; $^{13}$C{1H} NMR (101 MHz, Chloroform-*d*) δ 161.1, 158.4, 140.0, 127.9, 122.3, 115.2, 55.7, 49.6, 29.7, 27.4, 26.1, 24.5.; *m/z* (ES+): 244 ([M–$BF_4$]$^+$, 100%). HRMS (ESI) *m/z*: [M–$BF_4$]$^+$ Calculated for $C_{14}H_{18}N_3O$ 244.1437; Found 244.1442.

## Synthesis of 2-(4-fluorophenyl)-6,7,8,9-tetrahydro-5*H*-[1,2,4]triazolo[4,3-a]azepin-2-ium tetrafluoroborate 9c

Triazolium tetrafluoroborate **9c** was obtained as a white solid (0.342 g, 11%). mp = 118-122 °C; $^{1}$H NMR (400 MHz, Chloroform-*d*) δ 10.03 (s, 1H), 7.96 – 7.77 (m, 2H), 7.27 – 7.10 (m, 2H), 4.52 (t, *J* = 4.1 Hz, 2H), 3.29 – 3.05 (m, 2H), 1.99 (dt, *J* = 5.6, 2.7 Hz, 6H).; $^{13}$C{1H} NMR (101 MHz, Chloroform-*d*) δ 164.6, 162.1, 158.6, 140.9, 131.1, 131.0, 123.1, 123.0, 117.3, 117.0, 49.8, 29.6, 27.2, 26.1, 24.4.; HRMS (ESI) *m/z*: [M–$BF_4$]$^+$ Calculated for $C_{13}H_{15}N_3F$ 232.1250; Found 232.1252.

## S1.4 Deuterium Exchange and p$K_a$ Measurements

### S1.4.1 Preparation of Solutions

The deuterium exchange reactions were monitored by $^1$H NMR spectroscopy in $D_2O$ solution, with the p*D* values of all the experiment in between 0.59 – 3.50. The solution p*D* values were controlled by DCl or acetic acid buffer, and the internal standard, tetramethylammonium deuteriosulfate, were used to monitor the potential decomposition of the triazolium salts. KCl was used to control the ionic strength, $I$ = 1.0.

### S1.4.2 Measurement of p*D* in $D_2O$ Solution and Determination of [$DO^-$]

The p*H* values of buffer solutions were determined at 25 °C using a MeterLabTM PHM 290 p*H*-Stat Controller equipped with a radiometer (p*H* 1.68 - 4.00 @ 25 °C) combination electrode, that could be standardised between p*H* 1.68 – 4.00 to encompass the p*H* of the buffer solution. All the solutions were incubated in a thermostated water bath with temperature at 25 ± 0.1 °C.

The p*D* (± 0.03) was calculated by adding 0.4 to the observed reading of the p*H* meter in the $D_2O$ solution.[S2] The concentration of deuteroxide, [$DO^-$] (M), was calculated using Eq s1, where $K_w = 10^{-14.87}$ $M^2$ is the ion product of $D_2O$ at 25 °C.[S3] The apparent activity coefficient of deuteroxide ion, $\gamma_{DO}$ = 0.73, was determined from the measured p*H* of solutions of known [$OH^-$] in water at $I$ = 1.0 (KCl) at 25 °C, with the assumption that $\gamma_{DO} = \gamma_{HO}$. For these measurements, the p*H* apparatus was standardized at 7.00 and at 12.47 with calcium hydroxide that was saturated at 21 °C.[S3] The p*D* values for each experiment were recorded at the beginning and end of reactions, and were found to be constant within error (± 0.03).

$$[DO^-] = \frac{(10^{pD-pK_W})}{\gamma_{DO}} \qquad (s1)$$

### S1.4.3 NMR Parameters

$^1$H NMR spectra of triazolium ions **7-9** were recorded on either Varian 400 or Oxford Varian Inova 500 MHz spectrometers. Spectra were run with 32 transients and a relaxation delay of 20 sec, sweep width of 8298.76 Hz, acquisition time of 4 sec and a 90° pulse angle. The total running time for each spectrum equals to 12 min 48 sec. The $^1$H NMR spectral baselines were subject to a first-order drift correction before integration of the peak areas.

### S1.4.4 Determination of Rate Constants for Deuteroxide-Catalyzed Exchange in Water

Deuterium exchange reactions were monitored in buffered $D_2O$ solutions for triazolium salts **7a-c**, **7k**, **8a-c**, **8k**, **9a-c**, **9k**. A deuterium exchange study of **7a-c, 7k** has been previously reported by us as part of a larger study,[S4] however, these experiments were repeated herein to demonstrate reproducibility and for consistency in analysis . Hydrogen-deuterium exchange of the C(3)-H results in a decrease of the singlet due to the C(3)-H of triazolium salts at ~10 ppm relative to the broad triplet at 3.3 ppm due to the methyl hydrogens of internal standard, tetramethylammonium deuteriosulphate. Substrate and product peak areas were compared with the peak of internal standard to confirm that no parallel reactions including hydrolysis or decomposition of triazolium salt substrates were occurring under our conditions. Values for the fraction of remaining substrate could be calculated using Eq s2 by comparing the intergrated areas of the singlet due to the C(3)-H ($A_{C(3)-H}$) with those of the internal standard

($A_{std}$). The observed first order rate constant for deuterium exchange, $k_{ex}$ ($s^{-1}$), at a given p*D* could be obtained as the slope of a semilogarithmic plot of the fraction of remaining substrate against time according to Eq s3. For triazolium salts **7a**-**c**, **8a**-**c**, **9a**-**c**, good linear fits of log $k_{ex}$ – p*D* data to Eq 2 are observed whereas data for pentafluorophenyl triazolium salts **7k**, **8k** and **9k** instead show excellent fits to Eq 3 (main manuscript).

$$f(s) = \frac{(A_{C(3)-H}/A_{std})_t}{(A_{C(3)\text{-}H}/A_{std})_0} \quad (s2)$$

$$ln\, f(s) = -\, k_{ex}t \quad (s3)$$

#### S1.4.4.1 Representative ¹H NMR Spectral Overlays of C(3)-H/D Exchange

For triazolium salts **7-9a**, **7-9b**, **7-9c** and **7-9k**, representative ¹H NMR spectral overlays are presented below showing the progress of C(3)-H/D exchange at one p*D* value in each case. For a given N-aryl substituent (*e.g.* N-Ar = 4-methoxyphenyl for **7-9a**), the closely similar p*D* values are chosen for the representative spectral overlays to enable a comparison of the effect of fused ring size on the progress of C(3)-H/D exchange.

Figure S1. Representative $^1H$ NMR spectra at 400MHz of triazolium salt **7a** (10mM, p*D* 1.78), obtained during exchange of C(3)-H (s, 9.99 ppm) for deuterium in $D_2O$ at 25 ºC and I = 1.0 M (KCl). [Internal standard, tetramethylammonium deuteriosulfate (s, 3.17 ppm)]

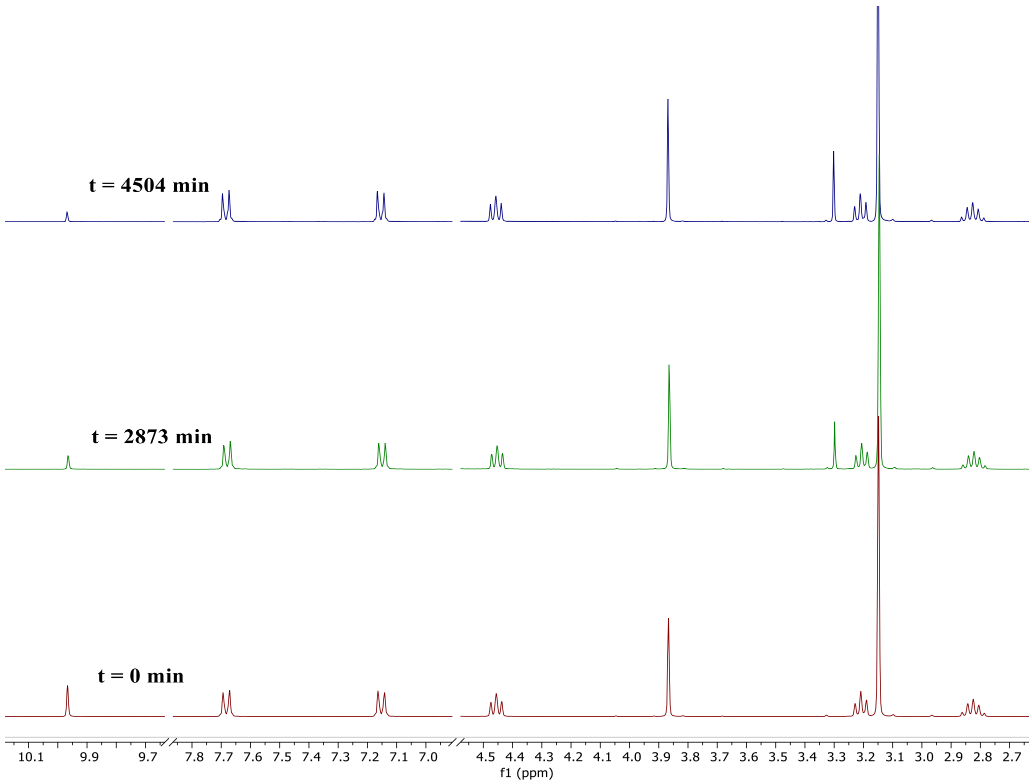

Figure S2. Representative $^{1}$H NMR spectra at 400MHz of triazolium salt **8a** (10mM, p*D* 1.78), obtained during exchange of C(3)-H (s, 10 ppm) for deuterium in $D_2O$ at 25 ºC and I = 1.0 M (KCl). [Internal standard, tetramethylammonium deuteriosulfate (s, 3.17 ppm)]

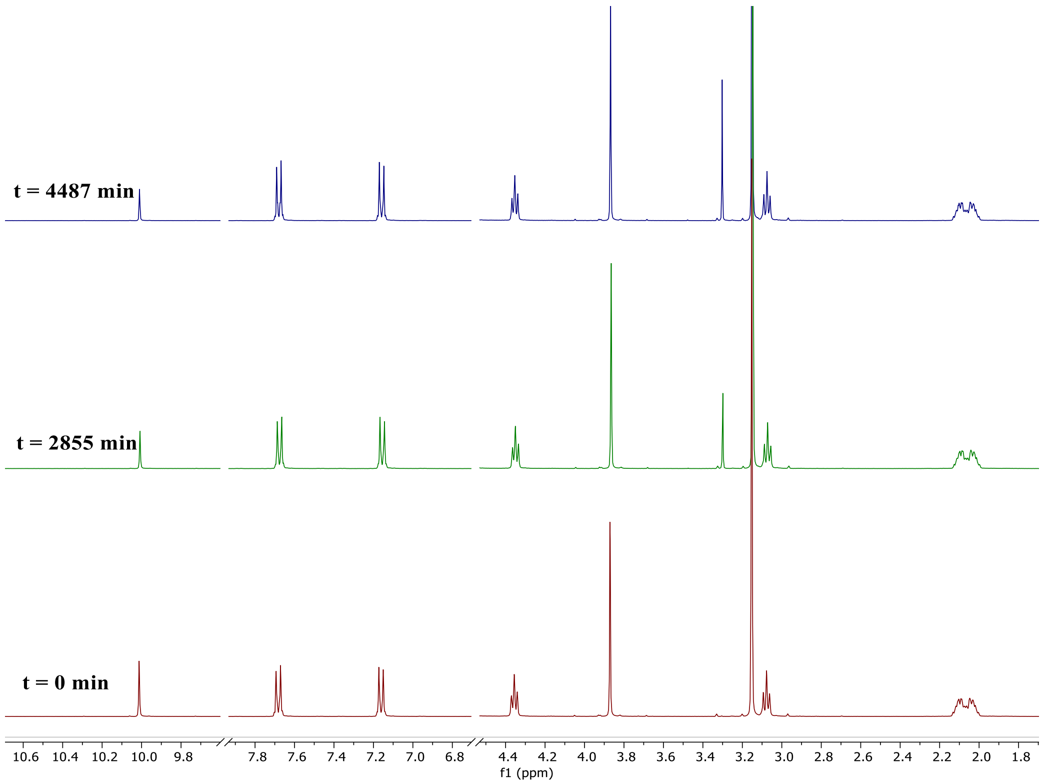

Figure S3. Representative $^{1}H$ NMR spectra at 400MHz of triazolium salt **9a** (10mM, p*D* 1.79), obtained during exchange of C(3)-H (s, 10 ppm) for deuterium in $D_2O$ at 25 ºC and I = 1.0 M (KCl). [Internal standard, tetramethylammonium deuteriosulfate (s, 3.17 ppm)]

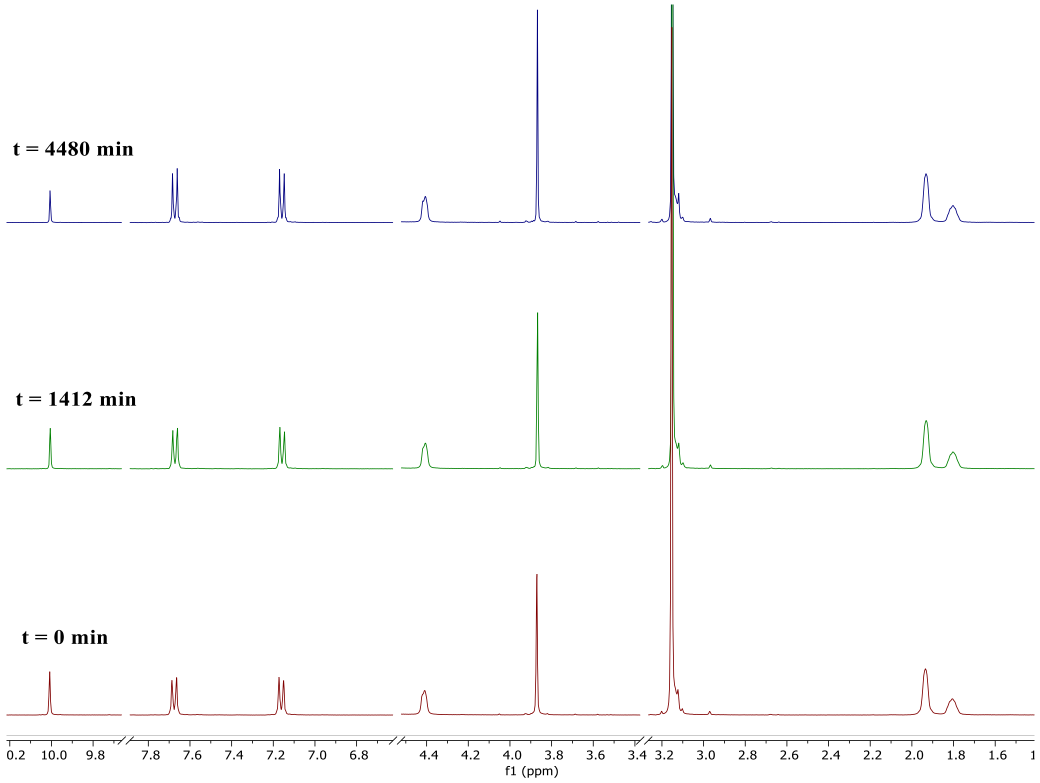

Figure S4. Representative $^{1}$H NMR spectra at 400MHz of triazolium salt **7b** (10mM, p*D* 1.77), obtained during exchange of C(3)-H (s, 10.1 ppm) for deuterium in $D_2O$ at 25 ºC and I = 1.0 M (KCl). [Internal standard, tetramethylammonium deuteriosulfate (s, 3.17 ppm)]

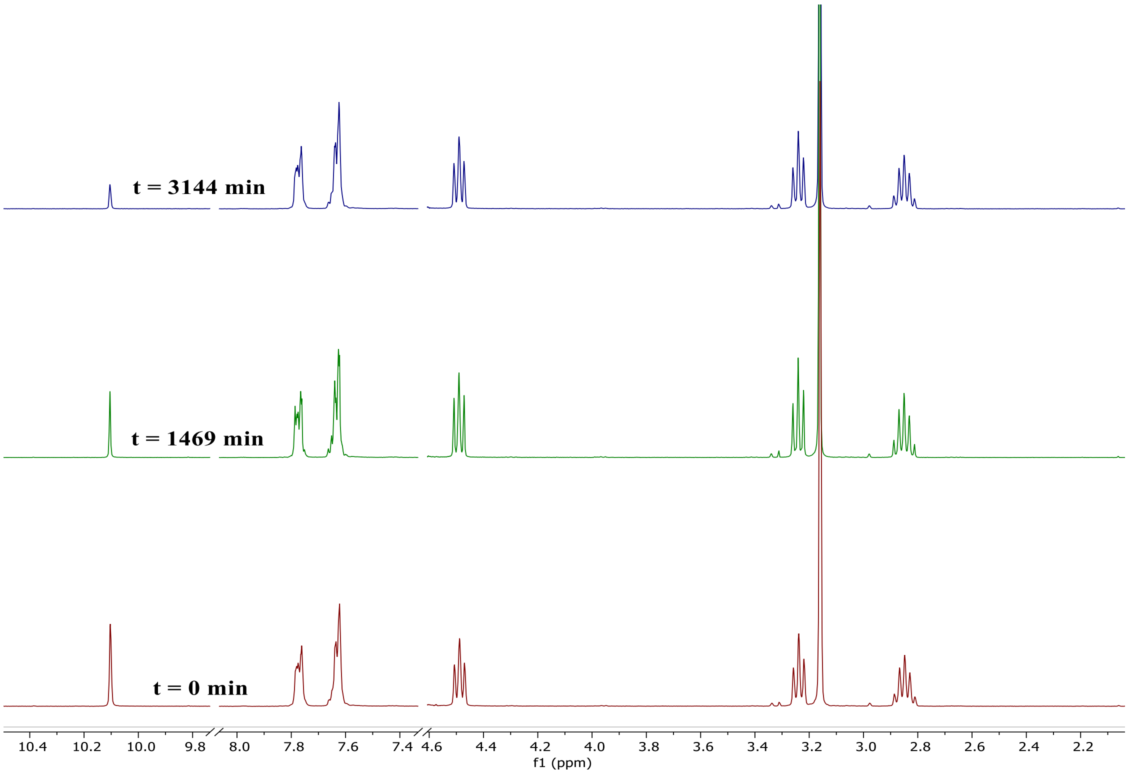

Figure S5. Representative $^{1}H$ NMR spectra at 400MHz of triazolium salt **8b** (10mM, p*D* 1.69), obtained during exchange of C(3)-H (s, 10.1 ppm) for deuterium in $D_2O$ at 25 ºC and I = 1.0 M (KCl). [Internal standard, tetramethylammonium deuteriosulfate (s, 3.17 ppm)]

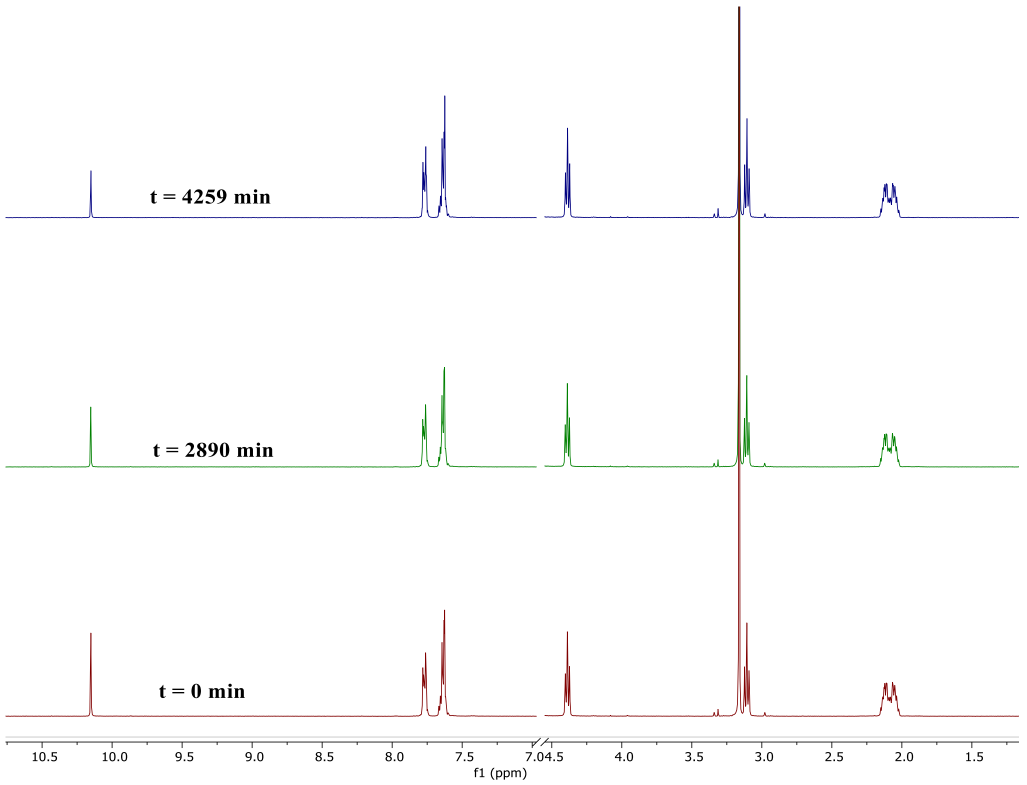

Figure S6. Representative $^1$H NMR spectra at 400MHz of triazolium salt **9b** (10mM, p*D* 1.73), obtained during exchange of C(3)-H (s, 10.2 ppm) for deuterium in $D_2O$ at 25 ºC and I = 1.0 M (KCl). [Internal standard, tetramethylammonium deuteriosulfate (s, 3.17 ppm)]

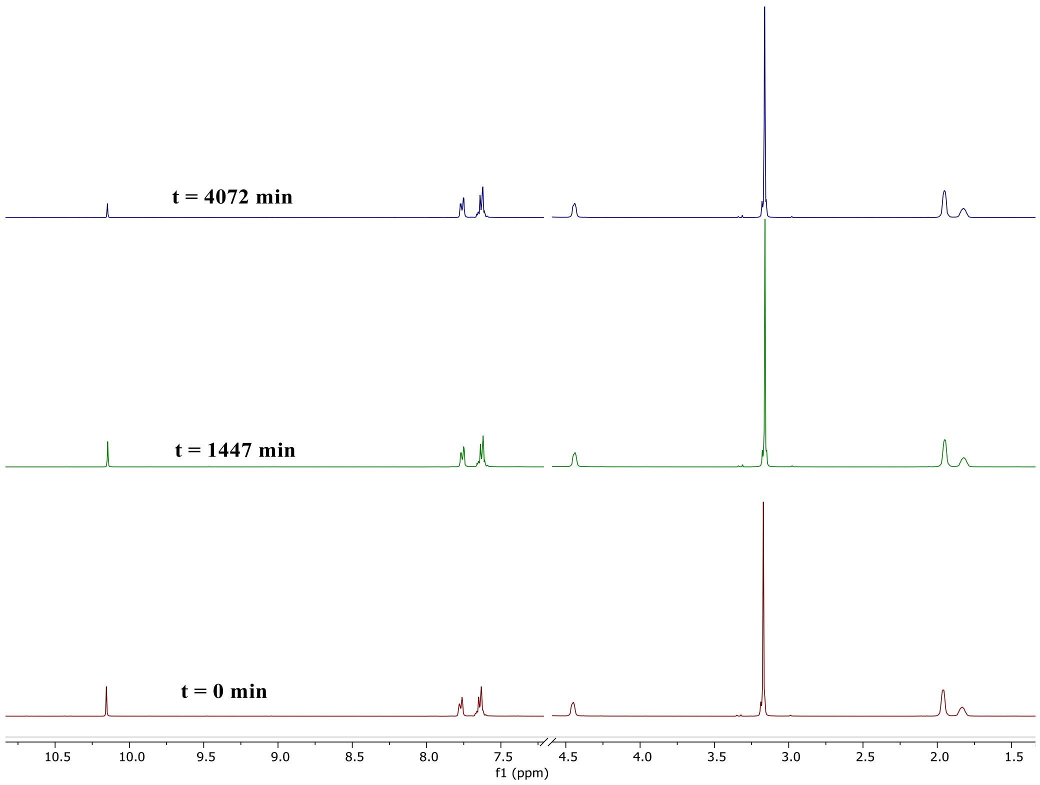

Figure S7. Representative $^{1}H$ NMR spectra at 400MHz of triazolium salt **7c** (10mM, p*D* 1.77), obtained during exchange of C(3)-H (s, 10.1 ppm) for deuterium in $D_2O$ at 25 ºC and I = 1.0 M (KCl). [Internal standard, tetramethylammonium deuteriosulfate (s, 3.17 ppm)]

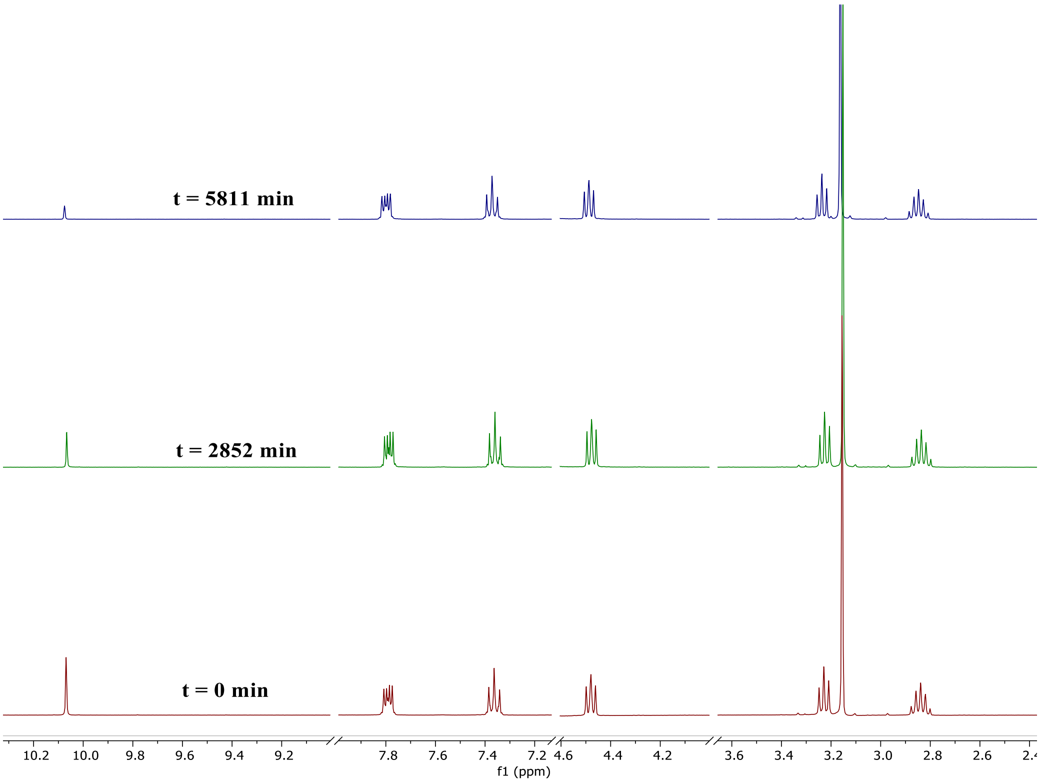

Figure S8. Representative $^{1}$H NMR spectra at 400MHz of triazolium salt **8c** (10mM, p*D* 2.29), obtained during exchange of C(3)-H (s, 10.1 ppm) for deuterium in $D_2O$ at 25 ºC and I = 1.0 M (KCl). [Internal standard, tetramethylammonium deuteriosulfate (s, 3.17 ppm)]

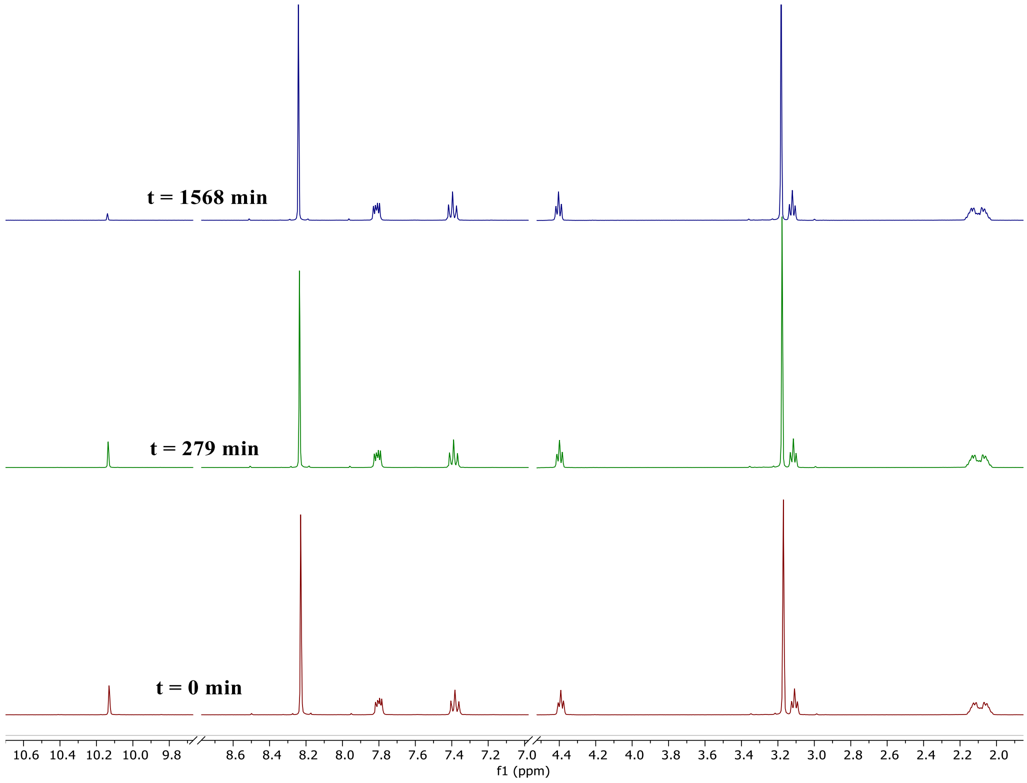

Figure S9. Representative $^1$H NMR spectra at 400MHz of triazolium salt **9c** (10mM, p*D* 1.99), obtained during exchange of C(3)-H (s, 10.1 ppm) for deuterium in $D_2O$ at 25 ºC and I = 1.0 M (KCl). [Internal standard, tetramethylammonium deuteriosulfate (s, 3.17 ppm)]

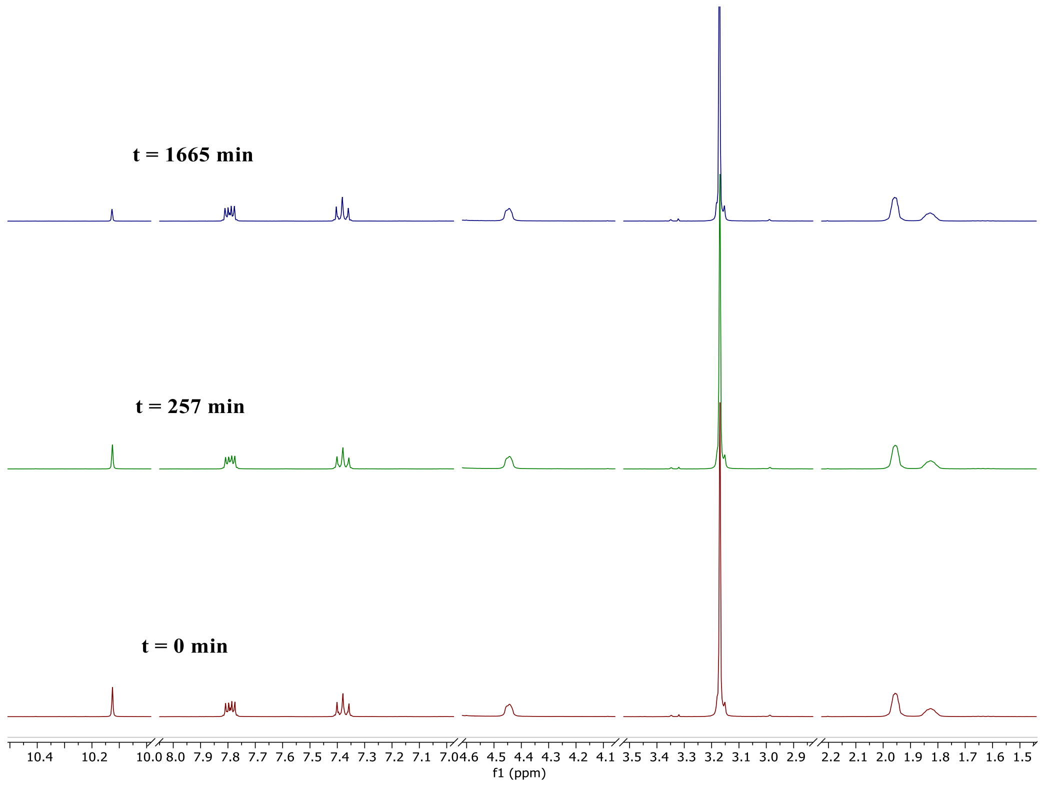

Figure S10. Representative $^1$H NMR spectra at 400MHz of triazolium salt **7k** (10mM, p*D* 1.58), obtained during exchange of C(3)-H (s, 10 ppm) for deuterium in $D_2O$ at 25 ºC and I = 1.0 M (KCl). [Internal standard, tetramethylammonium deuteriosulfate (s, 3.17 ppm)]

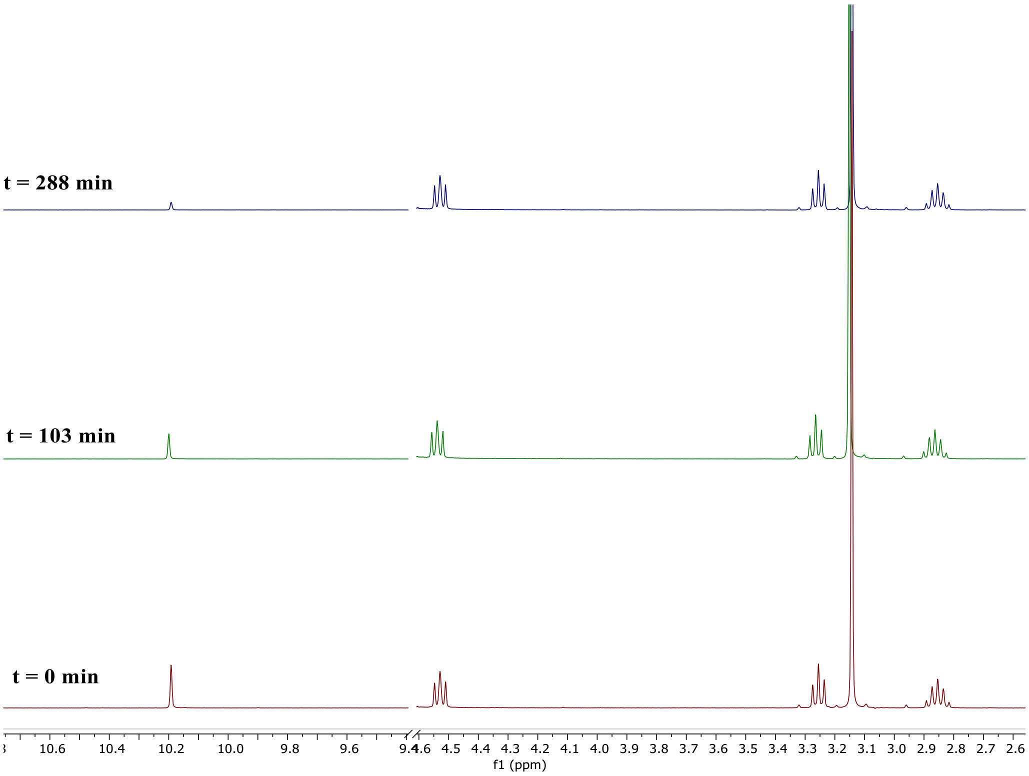

Figure S11. Representative $^{1}$H NMR spectra at 400MHz of triazolium salt **8k** (10mM, p*D* 1.58), obtained during exchange of C(3)-H (s, 10 ppm) for deuterium in $D_2O$ at 25 ºC and I = 1.0 M (KCl). [Internal standard, tetramethylammonium deuteriosulfate (s, 3.17 ppm)]

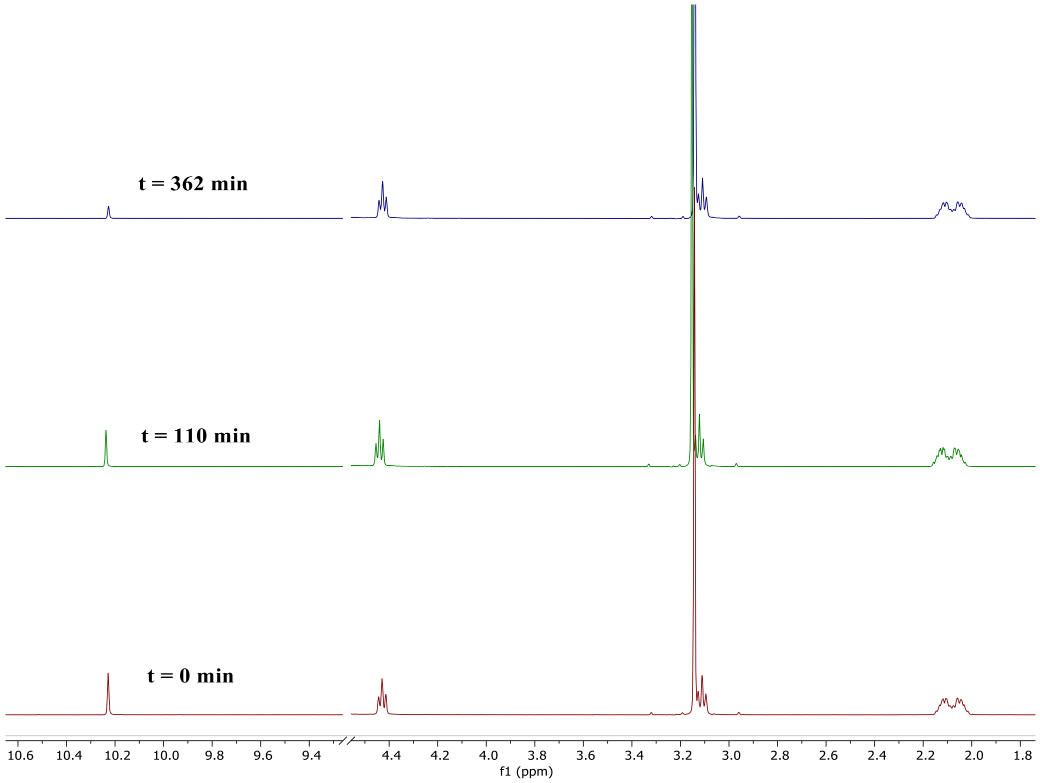

Figure S12. Representative $^{1}$H NMR spectra at 400MHz of triazolium salt **9k** (10mM, p*D* 1.56), obtained during exchange of C(3)-H (s, 10 ppm) for deuterium in $D_2O$ at 25 ºC and I = 1.0 M (KCl). [Internal standard, tetramethylammonium deuteriosulfate (s, 3.17 ppm)]

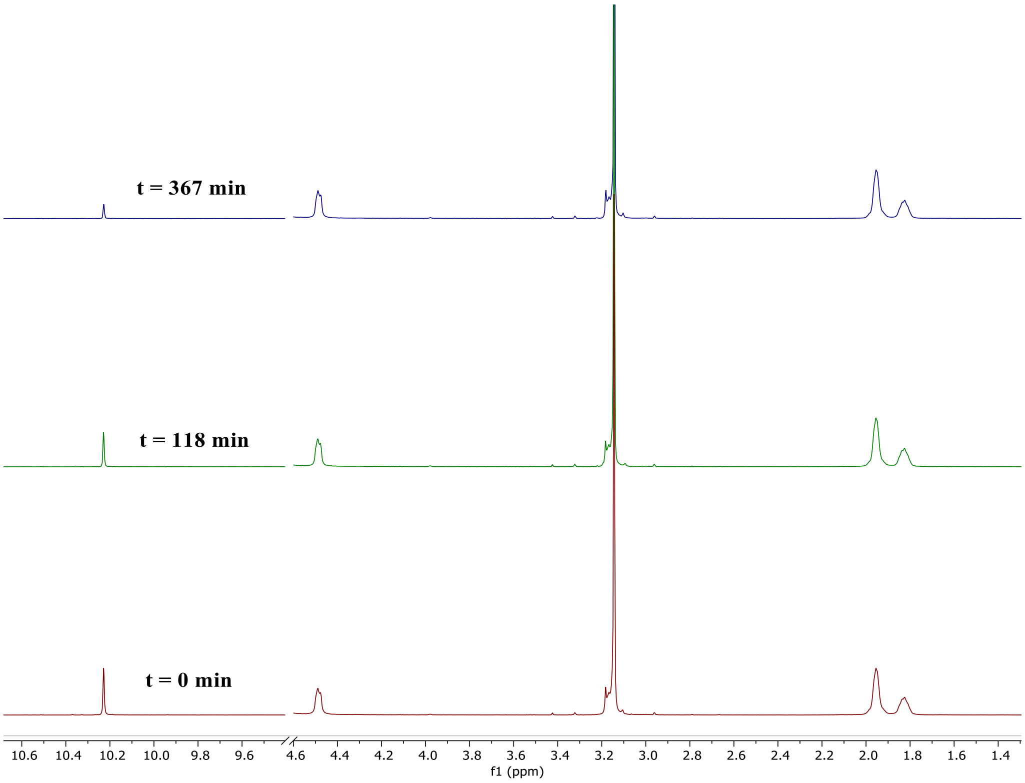


## S1.4.4.2 Semilogarithmic Plots of *f*(s) versus Time

For triazolium salts **7-9a**, **7-9b**, **7-9c** and **7-9k**, all semilogarithmic plots of the fraction of unexchanged substrate (*f*(s)) versus time at different p*D* values are included below. The observed first order rate constant for deuterium exchange, $k_{ex}$ ($s^{-1}$), at a given p*D* could be obtained as the slope of the semilogarithmic plot of the fraction of remaining substrate against time according to Eq s3.

Figure S13. Semilogarithmic plots of the fraction of unexchanged substrate against time for the deuterium exchange reaction of **7a** in solutions of DCl in $D_2O$ at 25 ºC and I = 1.0 M (KCl).

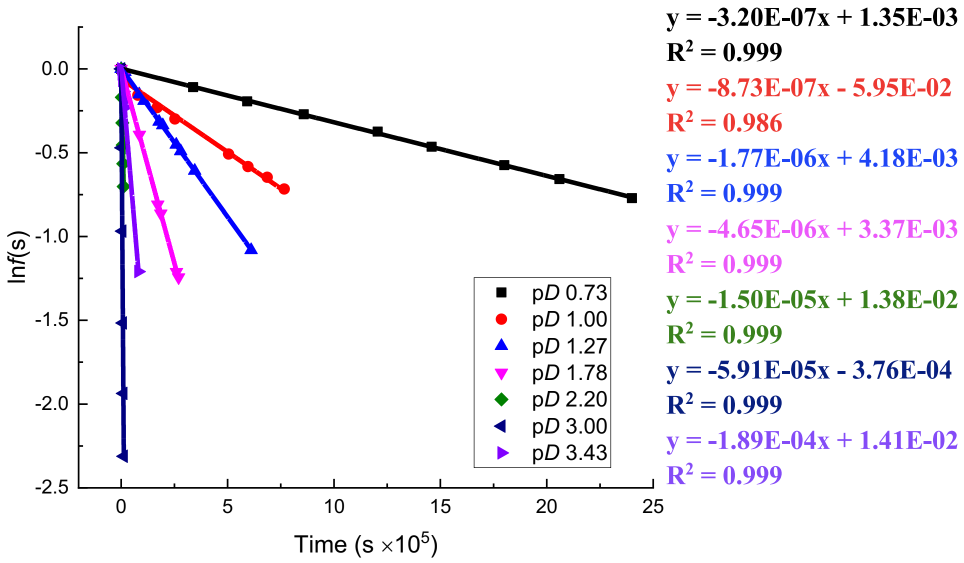


Figure S14. Semilogarithmic plots of the fraction of unexchanged substrate against time for the deuterium exchange reaction of **8a** in solutions of DCl in $D_2O$ at 25 ºC and I = 1.0 M (KCl).

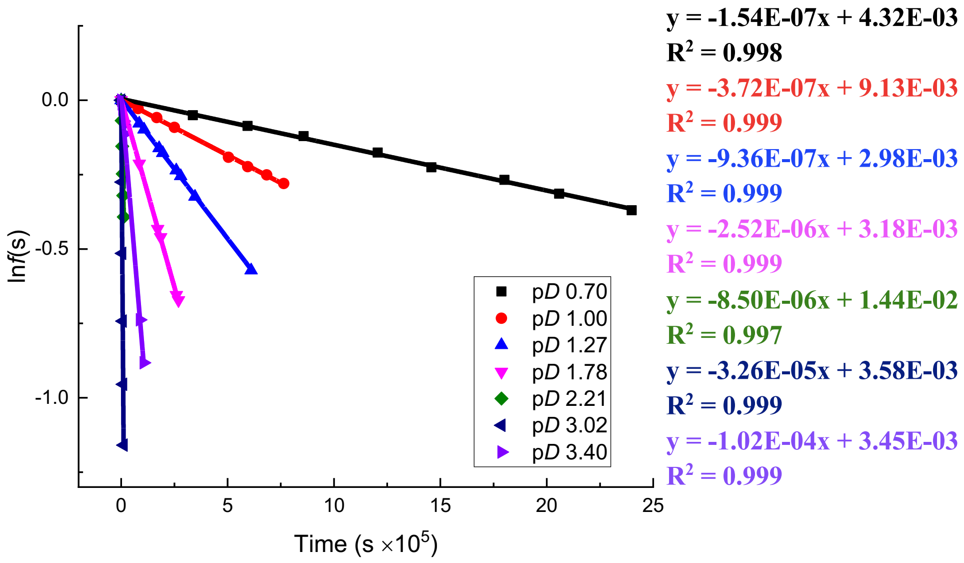

Figure S15. Semilogarithmic plots of the fraction of unexchanged substrate against time for the deuterium exchange reaction of **9a** in solutions of DCl in $D_2O$ at 25 ºC and I = 1.0 M (KCl).

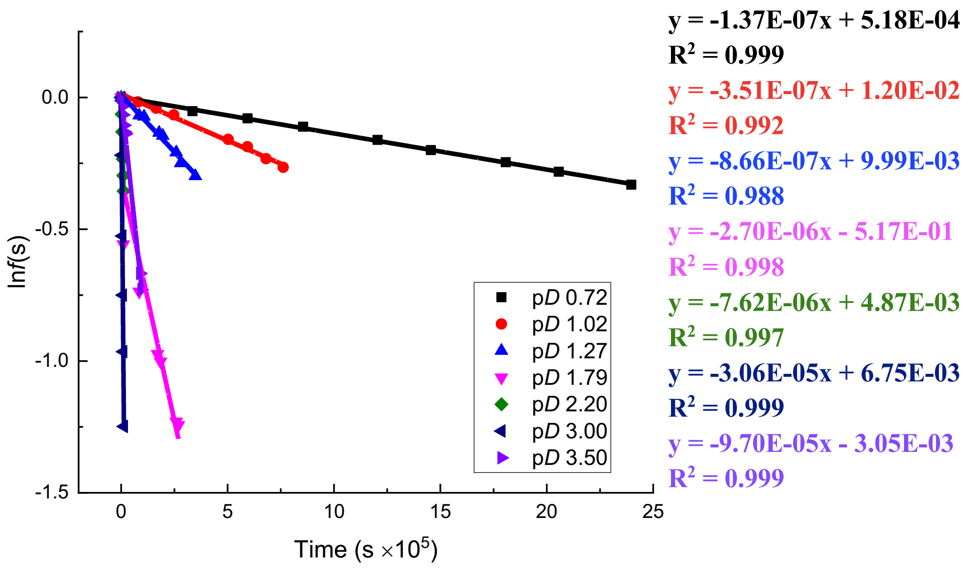


Figure S16. Semilogarithmic plots of the fraction of unexchanged substrate against time for the deuterium exchange reaction of **7b** in solutions of DCl in $D_2O$ at 25 ºC and I = 1.0 M (KCl).

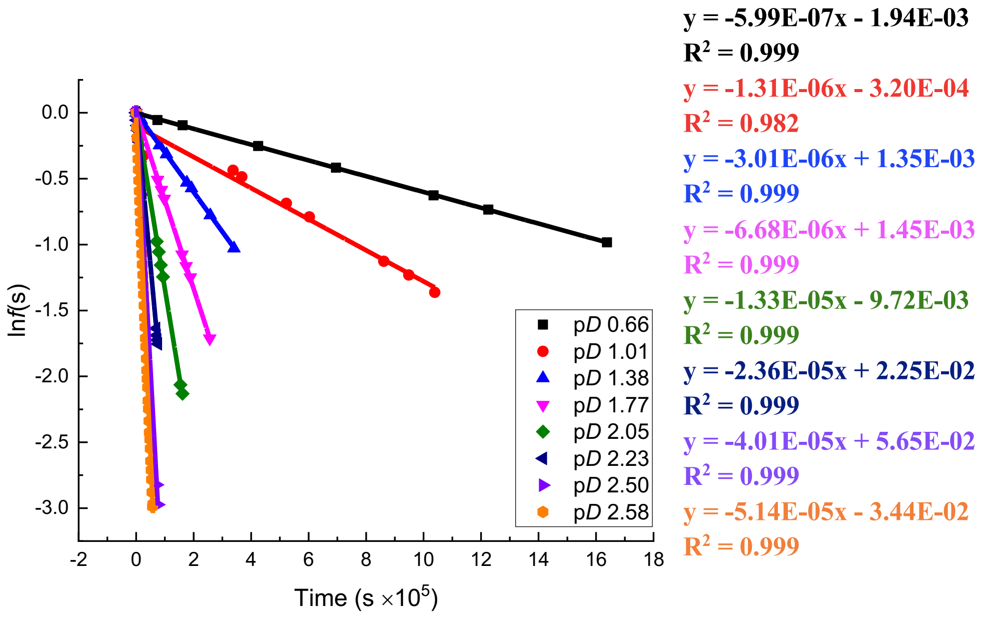

Figure S17. Semilogarithmic plots of the fraction of unexchanged substrate against time for the deuterium exchange reaction of **8b** in solutions of DCl in $D_2O$ at 25 ºC and I = 1.0 M (KCl).

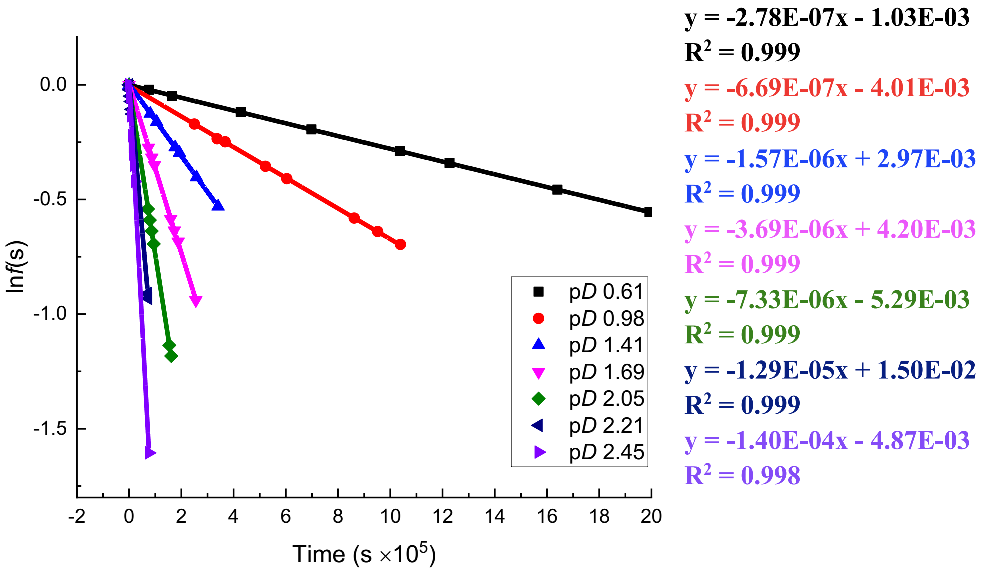


Figure S18. Semilogarithmic plots of the fraction of unexchanged substrate against time for the deuterium exchange reaction of **9b** in solutions of DCl in $D_2O$ at 25 ºC and I = 1.0 M (KCl).

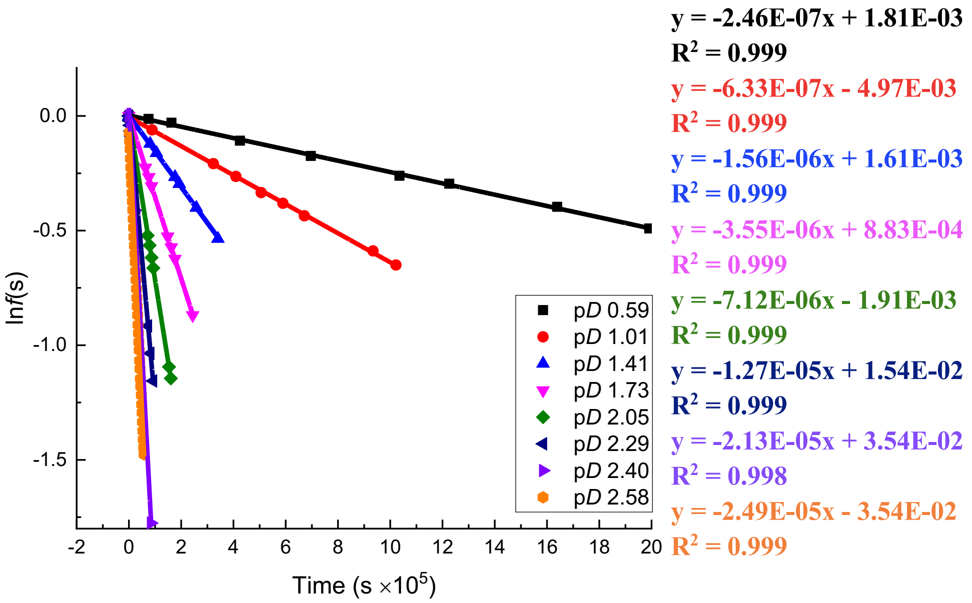

Figure S19. Semilogarithmic plots of the fraction of unexchanged substrate against time for the deuterium exchange reaction of **7c** in solutions of DCl in $D_2O$ at 25 ºC and I = 1.0 M (KCl).

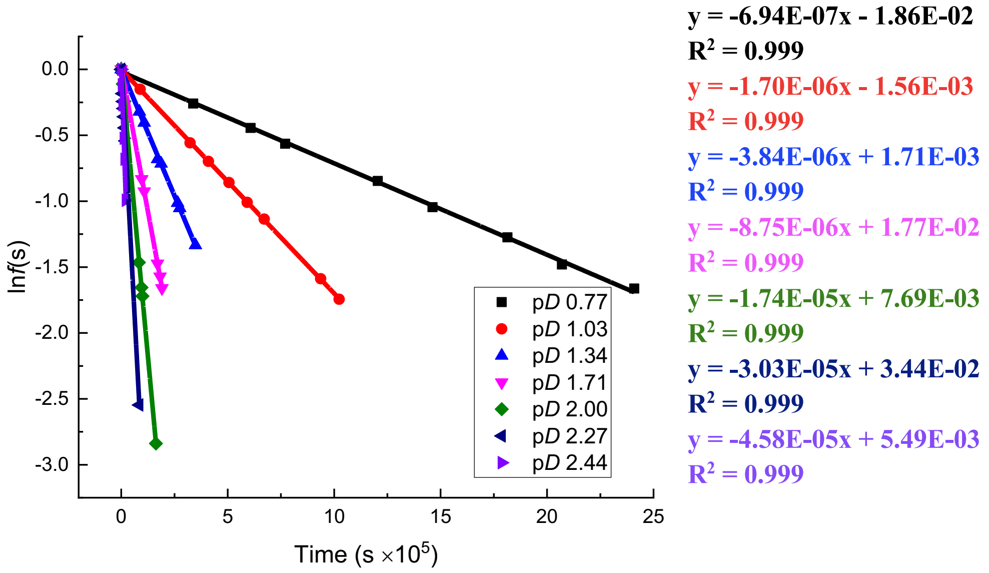


Figure S20. Semilogarithmic plots of the fraction of unexchanged substrate against time for the deuterium exchange reaction of **8c** in solutions of DCl in $D_2O$ at 25 ºC and I = 1.0 M (KCl).

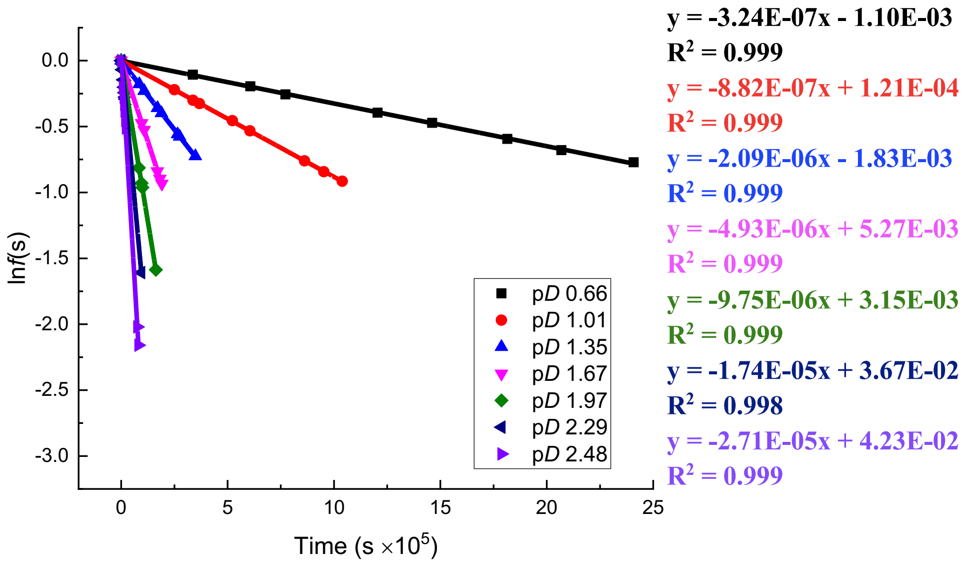

Figure S21. Semilogarithmic plots of the fraction of unexchanged substrate against time for the deuterium exchange reaction of **9c** in solutions of DCl in $D_2O$ at 25 ºC and I = 1.0 M (KCl).

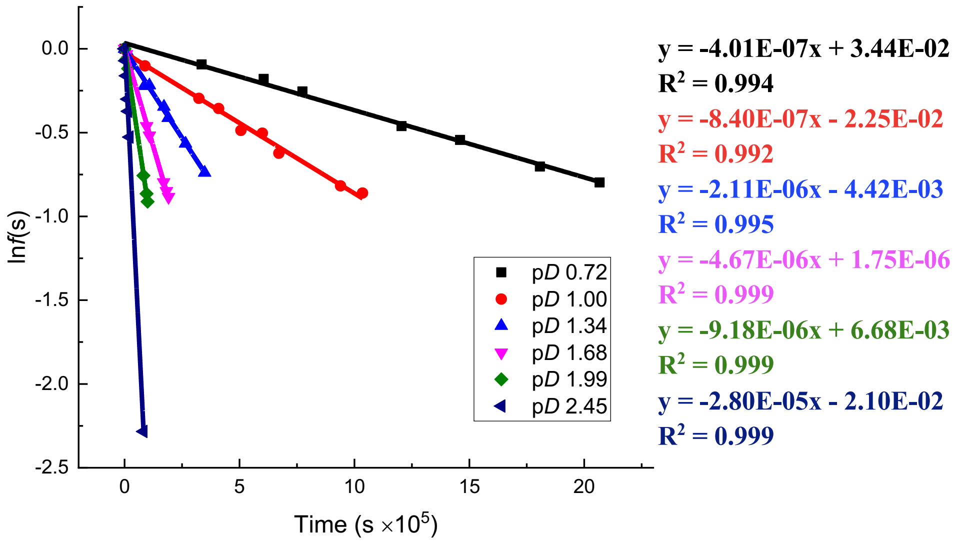


Figure S22. Semilogarithmic plots of the fraction of unexchanged substrate against time for the deuterium exchange reaction of **7k** in solutions of DCl in $D_2O$ at 25 ºC and I = 1.0 M (KCl).

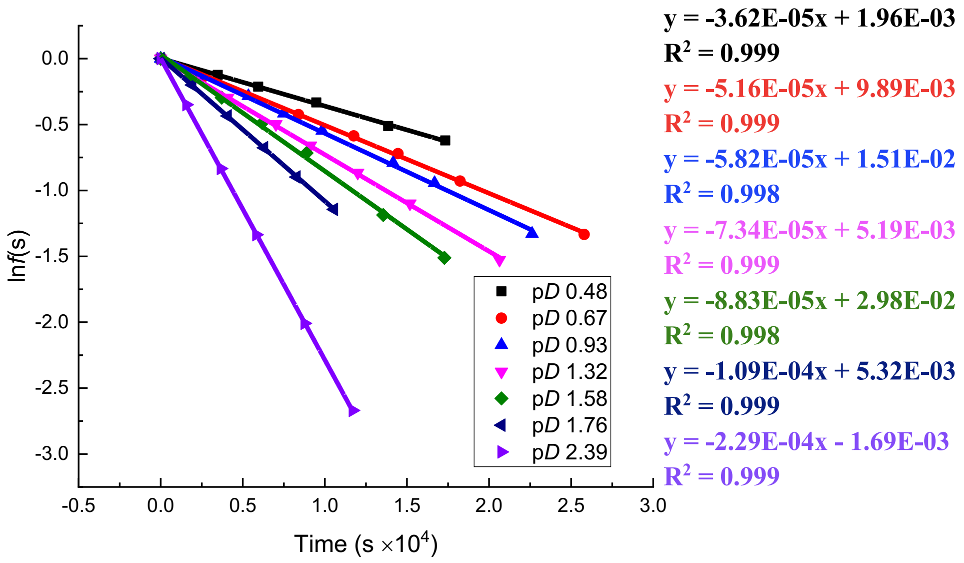

Figure S23. Semilogarithmic plots of the fraction of unexchanged substrate against time for the deuterium exchange reaction of **8k** in solutions of DCl in $D_2O$ at 25 ºC and I = 1.0 M (KCl).

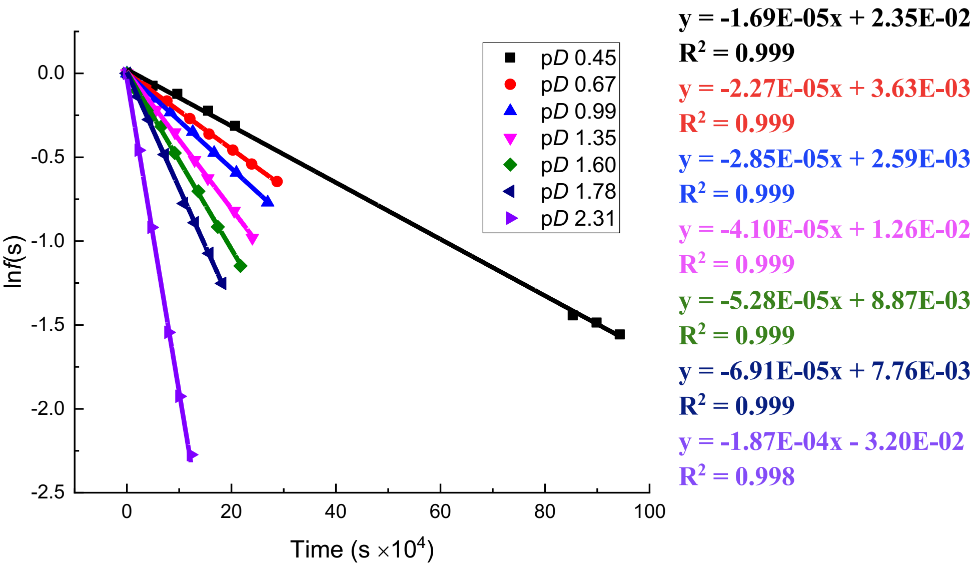


Figure S24. Semilogarithmic plots of the fraction of unexchanged substrate against time for the deuterium exchange reaction of **9k** in solutions of DCl in $D_2O$ at 25 ºC and I = 1.0 M (KCl).

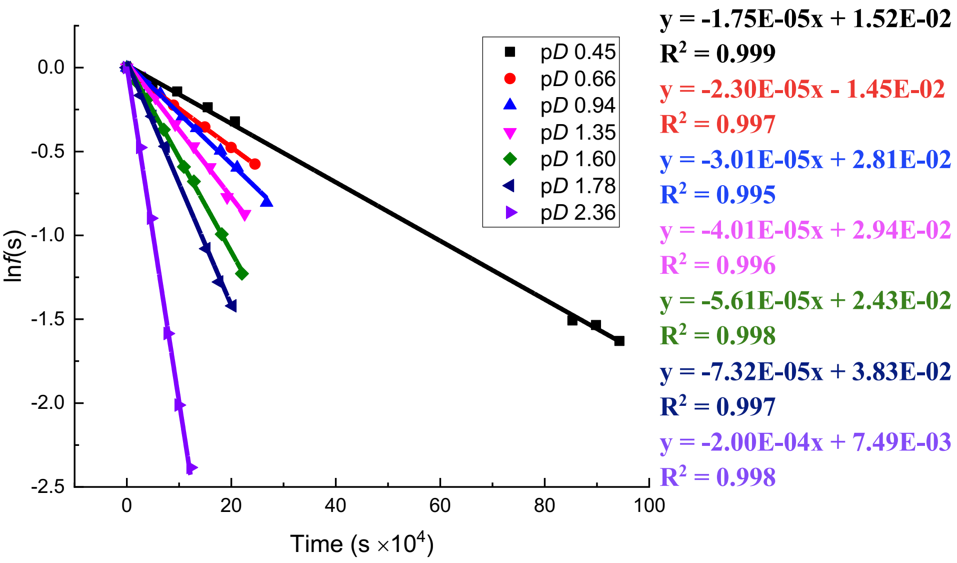

### S1.4.4.3 Log $k_{ex}$ – p*D* Profiles for 7a-c, 8a-c and 9a-c

For triazolium salts **7a-c**, **8a-c** and **9a-c**, good linear fits of log $k_{ex}$ – p*D* data to Eq 2 (main manuscript) are observed. The log $k_{ex}$ – p*D* profiles are included below for **7-9a**, **7-9b** and **7-9c** showing fits to Eq 2 (Figures S25-27, respectively).

Figure S25. Plot of log $k_{ex}$ against p*D* for the C(3)-H/D exchange reaction of triazolium salt **7-9a** in solutions of DCl in $D_2O$ at 25 ºC and I = 1.0 M (KCl).

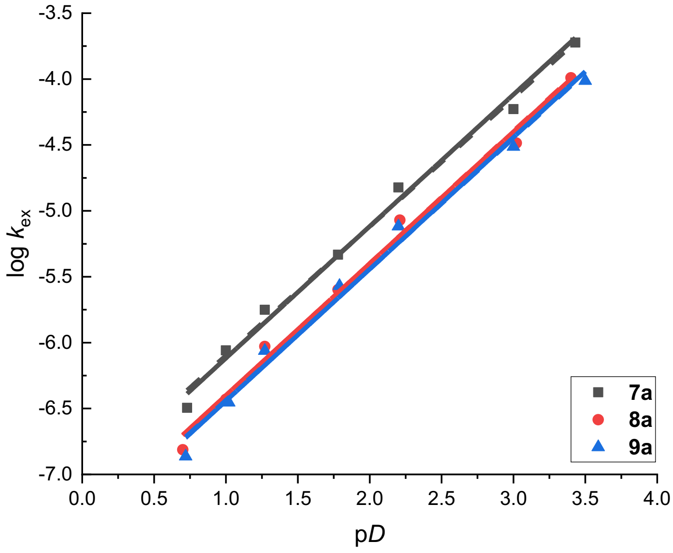


**Fitting with slope fixed = 1**

**y = x – 7.12 $R^2$ = 0.992**
**y = x – 7.40 $R^2$ = 0.992**
**y = x – 7.44 $R^2$ = 0.990**

**Fitting without restriction**

**y = 0.98x – 7.08 $R^2$ = 0.993**
**y = 1.00x – 7.41 $R^2$ = 0.992**
**y = 0.99x – 7.43 $R^2$ = 0.990**

Figure S26. Plot of log $k_{ex}$ against p$D$ for the C(3)-H/D exchange reaction of triazolium salt **7-9b** in solutions of DCl in $D_2O$ at 25 ºC and I = 1.0 M (KCl).

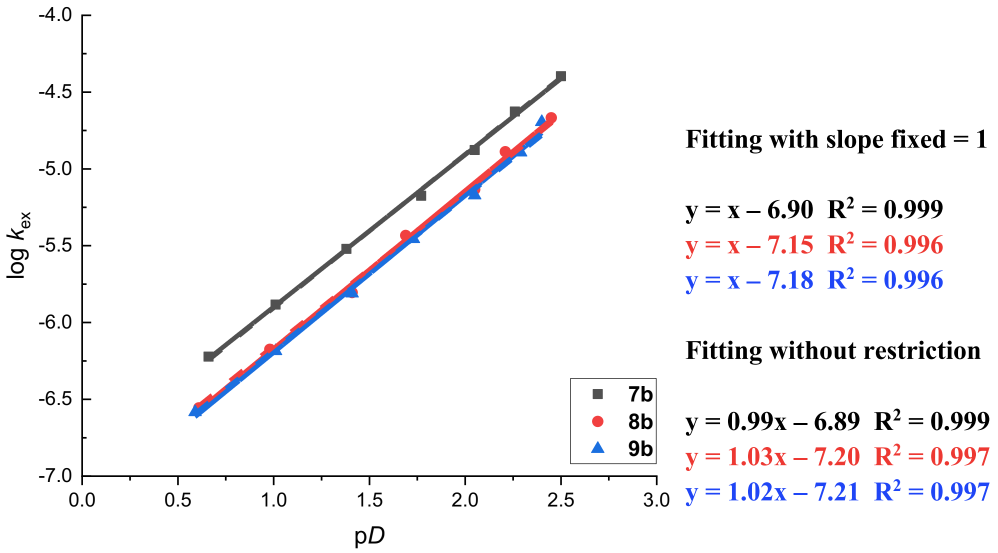


Figure S27. Plot of log $k_{ex}$ against p$D$ for the C(3)-H/D exchange reaction of triazolium salt **7-9c** in solutions of DCl in $D_2O$ at 25 ºC and I = 1.0 M (KCl).

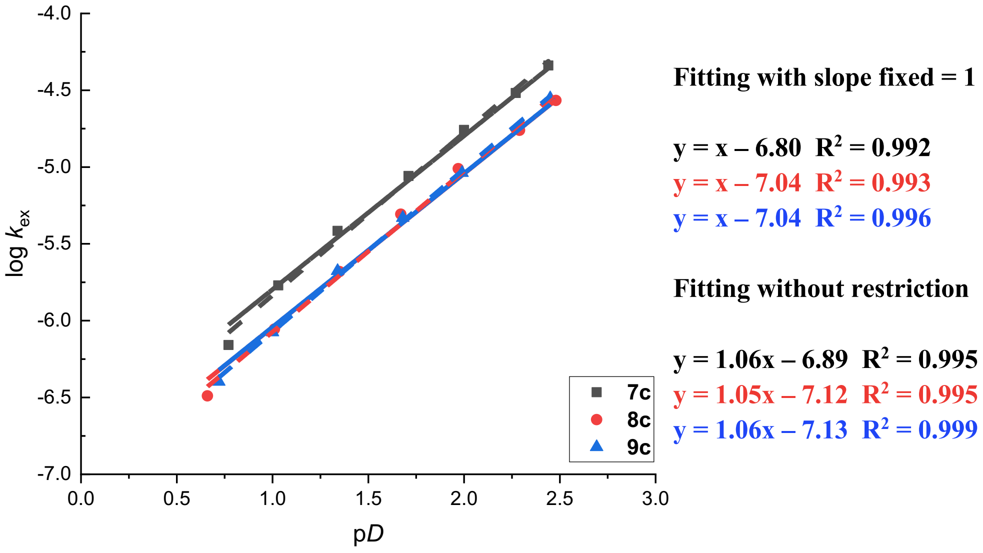


### S1.4.4.4 Log $k_{ex}$ – p$D$ Profiles for 7k, 8k and 9k

Log $k_{ex}$ – p$D$ data for pentafluorophenyl triazolium salts **7k**, **8k** and **9k** do not fit to Eq 2 and instead show excellent fits to Eq 3 (main manuscript). The log $k_{ex}$ – p$D$ profiles are included

below for **7k**, **8k** and **9k** showing fits to equation 3 (solid lines). The altered dependence of log $k_{ex}$ on p*D* as the p*D* decreases is consistent with the onset of alternative pathways for deuterium exchange, which we have discussed in detail previously.[S4-5] The most likely mechanistic explanation, as discussed previously for **7k**,[S4] is a pathway *via* N(1)-deuteration at lower p*D* values allowing for hydrogen-deuterium exchange of the N(1)-deuterated dicationic triazolium salt (Scheme S2).

Figure S28. Plot of log $k_{ex}$ against p*D* for the H/D exchange reaction of triazolium salt **7-9k** in solutions of DCl in $D_2O$ at 25 ºC and I = 1.0 M (KCl).

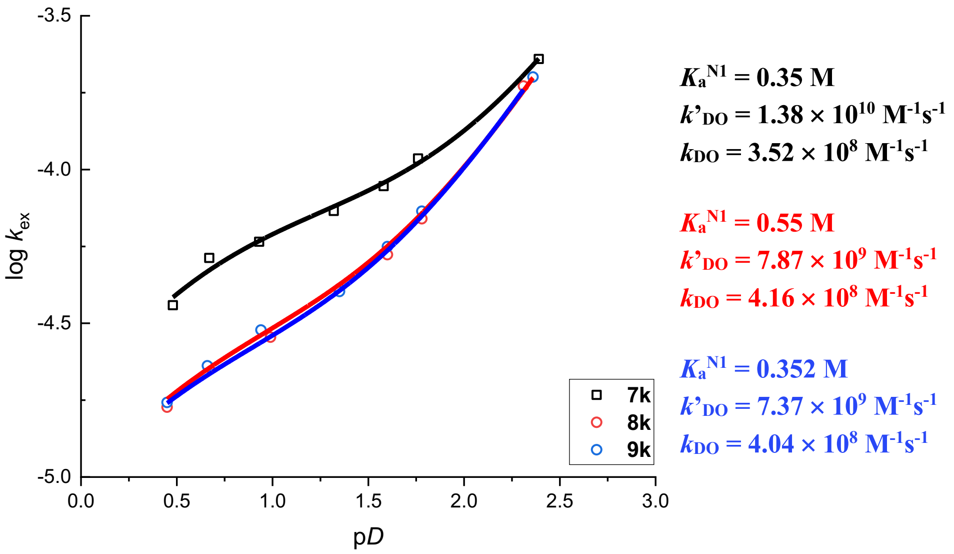

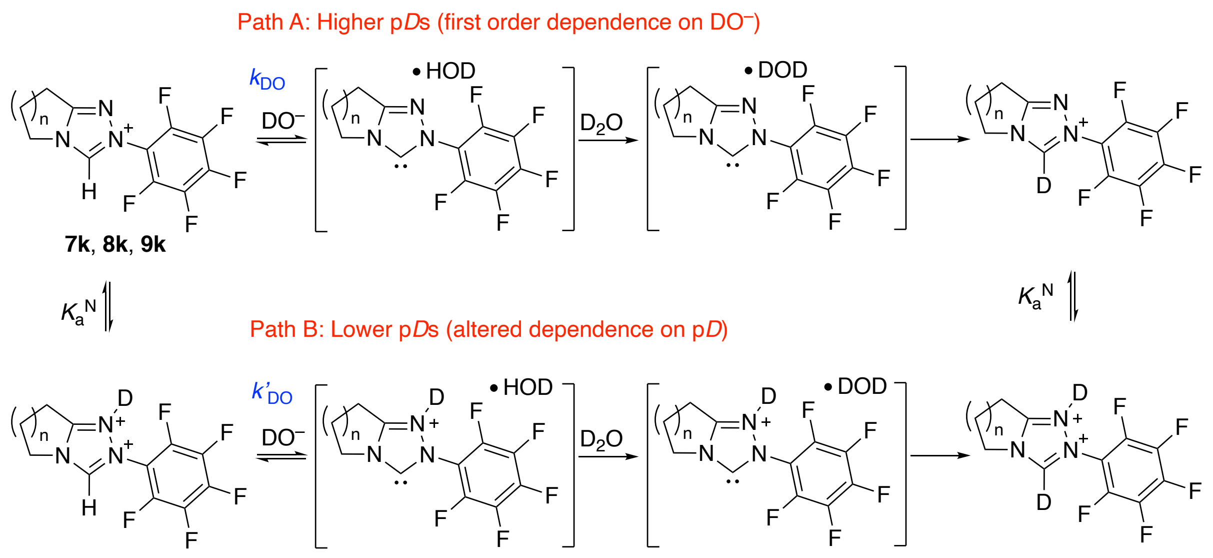


Scheme S2. Mechanisms of C(3)-H/D exchange for **7k**, **8k** and **9k** (tetrafluoroborate counterion excluded for clarity).

### S1.4.4.5 Reaction Data, First Order Rate Constants for Exchange ($k_{ex}$, $s^{-1}$) and Second Order Rate Constants for Exchange ($k_{DO}$, $M^{-1}s^{-1}$)

Table S1. First and second-order rate constants for exchange of the C(3)-H of triazolium salt **7a** for deuterium in solutions of DCl in $D_2O$ at 25 ºC and I = 1.0 M (KCl).

| p*D* | [DO⁻], M | $k_{ex}$, $s^{-1}$ [a] | $k_{DO}$, $M^{-1}s^{-1}$ |
|---|---|---|---|
| 0.73 | $9.92 \times 10^{-15}$ | $3.19 \times 10^{-7}$ | |
| 1.00 | $1.85 \times 10^{-14}$ | $8.72 \times 10^{-7}$ | |
| 1.27 | $3.44 \times 10^{-14}$ | $1.77 \times 10^{-6}$ | |
| 1.78 | $1.11 \times 10^{-13}$ | $4.65 \times 10^{-6}$ | $4.55 \times 10^{7}$ [b] |
| 2.20 | $2.93 \times 10^{-13}$ | $1.50 \times 10^{-5}$ | |
| 3.00 | $1.85 \times 10^{-12}$ | $5.91 \times 10^{-5}$ | |
| 3.43 | $4.97 \times 10^{-12}$ | $1.89 \times 10^{-4}$ | |

[a]First order rate constants for C(3)-H/D exchange ($k_{ex}$, $s^{-1}$) were obtained as the slopes of plots shown in Figure S13. [b]The second-order rate constant ($k_{DO}$, $M^{-1}s^{-1}$) was obtained from the fit of log $k_{ex}$ – p*D* data to Eq 2 (Figure S25).

Table S2. First and second-order rate constants for exchange of the C(3)-H of triazolium salt **8a** for deuterium in solutions of DCl in $D_2O$ at 25 °C and I = 1.0 M (KCl).

| p*D* | **[DO⁻], M** | $k_{ex}$, $s^{-1}$ [a] | $k_{DO}$, $M^{-1}s^{-1}$ |
|---|---|---|---|
| 0.70 | $9.26 \times 10^{-15}$ | $1.54 \times 10^{-7}$ | |
| 1.00 | $1.85 \times 10^{-14}$ | $3.72 \times 10^{-7}$ | |
| 1.27 | $3.44 \times 10^{-14}$ | $9.35 \times 10^{-7}$ | |
| 1.78 | $1.11 \times 10^{-13}$ | $2.52 \times 10^{-6}$ | $2.11 \times 10^{7}$ [b] |
| 2.21 | $2.99 \times 10^{-13}$ | $8.50 \times 10^{-6}$ | |
| 3.02 | $1.93 \times 10^{-12}$ | $3.26 \times 10^{-5}$ | |
| 3.40 | $4.64 \times 10^{-12}$ | $1.02 \times 10^{-4}$ | |

[a]First order rate constants for C(3)-H/D exchange ($k_{ex}$, $s^{-1}$) were obtained as the slopes of plots shown in Figure S14. [b]The second-order rate constant ($k_{DO}$, $M^{-1}s^{-1}$) was obtained from the fit of log $k_{ex}$ – p*D* data to Eq 2 (Figure S25).

Table S3. First and second-order rate constants for exchange of the C(3)-H of triazolium salt **9a** for deuterium in solutions of DCl in $D_2O$ at 25 °C and I = 1.0 M (KCl).

| p*D* | **[DO⁻], M** | $k_{ex}$, $s^{-1}$ [a] | $k_{DO}$, $M^{-1}s^{-1}$ |
|---|---|---|---|
| 0.72 | $9.70 \times 10^{-15}$ | $1.37 \times 10^{-7}$ | |
| 1.02 | $1.93 \times 10^{-14}$ | $3.51 \times 10^{-7}$ | |
| 1.27 | $3.44 \times 10^{-14}$ | $8.66 \times 10^{-7}$ | |
| 1.79 | $1.14 \times 10^{-13}$ | $2.69 \times 10^{-6}$ | $2.01 \times 10^{7}$ [b] |
| 2.20 | $2.93 \times 10^{-13}$ | $7.62 \times 10^{-6}$ | |
| 3.00 | $1.85 \times 10^{-12}$ | $3.06 \times 10^{-5}$ | |
| 3.50 | $5.84 \times 10^{-12}$ | $9.70 \times 10^{-5}$ | |

[a]First order rate constants for C(3)-H/D exchange ($k_{ex}$, $s^{-1}$) were obtained as the slopes of plots shown in Figure S15. [b]The second-order rate constant ($k_{DO}$, $M^{-1}s^{-1}$) was obtained from the fit of log $k_{ex}$ – p*D* data to Eq 2 (Figure S25).

Table S4. First and second-order rate constants for exchange of the C(3)-H of triazolium salt **7b** for deuterium in solutions of DCl in $D_2O$ at 25 °C and I = 1.0 M (KCl).

| p***D*** | **[DO⁻], M** | $k_{ex}$**, s⁻¹** [a] | $k_{DO}$**, M⁻¹s⁻¹** |
|---|---|---|---|
| 0.66 | $8.45 \times 10^{-15}$ | $5.99 \times 10^{-7}$ | |
| 1.01 | $1.89 \times 10^{-14}$ | $1.31 \times 10^{-6}$ | |
| 1.38 | $4.43 \times 10^{-14}$ | $3.01 \times 10^{-6}$ | |
| 1.77 | $1.09 \times 10^{-13}$ | $6.68 \times 10^{-6}$ | $6.99 \times 10^{7}$ [b] |
| 2.05 | $2.07 \times 10^{-13}$ | $1.33 \times 10^{-5}$ | |
| 2.26 | $3.36 \times 10^{-13}$ | $2.36 \times 10^{-5}$ | |
| 2.5 | $5.84 \times 10^{-13}$ | $4.01 \times 10^{-5}$ | |
| 2.58 | $7.03 \times 10^{-13}$ | $4.90 \times 10^{-5}$ | |

[a]First order rate constants for C(3)-H/D exchange ($k_{ex}$, s⁻¹) were obtained as the slopes of plots shown in Figure S16. [b]The second-order rate constant ($k_{DO}$, M⁻¹s⁻¹) was obtained from the fit of log $k_{ex}$ – p*D* data to Eq 2 (Figure S26).

Table S5. First and second-order rate constants for exchange of the C(3)-H of triazolium salt **8b** for deuterium in solutions of DCl in $D_2O$ at 25 °C and I = 1.0 M (KCl).

| p***D*** | **[DO⁻], M** | $k_{ex}$**, s⁻¹** | $k_{DO}$**, M⁻¹s⁻¹** |
|---|---|---|---|
| 0.61 | $7.53 \times 10^{-15}$ | $2.78 \times 10^{-7}$ | |
| 0.98 | $1.76 \times 10^{-14}$ | $6.69 \times 10^{-7}$ | |
| 1.41 | $4.75 \times 10^{-14}$ | $1.57 \times 10^{-6}$ | |
| 1.69 | $9.05 \times 10^{-14}$ | $3.69 \times 10^{-6}$ | $3.43 \times 10^{7}$ [b] |
| 2.05 | $2.07 \times 10^{-13}$ | $7.33 \times 10^{-6}$ | |
| 2.21 | $2.99 \times 10^{-13}$ | $1.29 \times 10^{-5}$ | |
| 2.45 | $5.21 \times 10^{-13}$ | $2.15 \times 10^{-5}$ | |

[a]First order rate constants for C(3)-H/D exchange ($k_{ex}$, s⁻¹) were obtained as the slopes of plots shown in Figure S17. [b]The second-order rate constant ($k_{DO}$, M⁻¹s⁻¹) was obtained from the fit of log $k_{ex}$ – p*D* data to Eq 2 (Figure S26).

Table S6. First and second-order rate constants for exchange of the C(3)-H of triazolium salt **9b** for deuterium in solutions of DCl in $D_2O$ at 25 ºC and I = 1.0 M (KCl).

| p*D* | **[DO⁻], M** | $k_{ex}$, $s^{-1}$ | $k_{DO}$, $M^{-1}s^{-1}$ |
|---|---|---|---|
| 0.59 | $7.18 \times 10^{-15}$ | $2.61 \times 10^{-7}$ | |
| 1.01 | $1.89 \times 10^{-14}$ | $6.52 \times 10^{-7}$ | |
| 1.41 | $4.74 \times 10^{-14}$ | $1.55 \times 10^{-6}$ | |
| 1.73 | $9.92 \times 10^{-14}$ | $3.50 \times 10^{-6}$ | $3.29 \times 10^{7}$ [b] |
| 2.05 | $2.07 \times 10^{-13}$ | $6.74 \times 10^{-6}$ | |
| 2.29 | $3.60 \times 10^{-13}$ | $1.29 \times 10^{-5}$ | |
| 2.40 | $4.64 \times 10^{-13}$ | $2.02 \times 10^{-5}$ | |
| 2.58 | $7.03 \times 10^{-13}$ | $3.01 \times 10^{-5}$ | |

[a]First order rate constants for C(3)-H/D exchange ($k_{ex}$, $s^{-1}$) were obtained as the slopes of plots shown in Figure S18. [b]The second-order rate constant ($k_{DO}$, $M^{-1}s^{-1}$) was obtained from the fit of log $k_{ex}$ – p*D* data to Eq 2 (Figure S26).

Table S7. First and second-order rate constants for exchange of the C(3)-H of triazolium salt **7c** for deuterium in solutions of DCl in $D_2O$ at 25 ºC and I = 1.0 M (KCl).

| p*D* | **[DO⁻], M** | $k_{ex}$, $s^{-1}$ [a] | $k_{DO}$, $M^{-1}s^{-1}$ |
|---|---|---|---|
| 0.77 | $1.09 \times 10^{-14}$ | $6.94 \times 10^{-7}$ | |
| 1.03 | $1.98 \times 10^{-14}$ | $1.70 \times 10^{-6}$ | |
| 1.34 | $4.04 \times 10^{-14}$ | $3.84 \times 10^{-6}$ | |
| 1.71 | $9.47 \times 10^{-14}$ | $8.75 \times 10^{-6}$ | $8.97 \times 10^{7}$ [b] |
| 2.00 | $1.85 \times 10^{-13}$ | $1.74 \times 10^{-5}$ | |
| 2.27 | $3.44 \times 10^{-13}$ | $3.03 \times 10^{-5}$ | |
| 2.44 | $5.09 \times 10^{-13}$ | $4.58 \times 10^{-5}$ | |

[a]First order rate constants for C(3)-H/D exchange ($k_{ex}$, $s^{-1}$) were obtained as the slopes of plots shown in Figure S19. [b]The second-order rate constant ($k_{DO}$, $M^{-1}s^{-1}$) was obtained from the fit of log $k_{ex}$ – p*D* data to Eq 2 (Figure S27).

Table S8. First and second-order rate constants for exchange of the C(3)-H of triazolium salt **8c** for deuterium in solutions of DCl in $D_2O$ at 25 ºC and I = 1.0 M (KCl).

| p*D* | $[DO^-]$, M | $k_{ex}$, $s^{-1}$ [a] | $k_{DO}$, $M^{-1}s^{-1}$ |
|---|---|---|---|
| 0.66 | $8.44 \times 10^{-15}$ | $3.24 \times 10^{-7}$ | |
| 1.01 | $1.89 \times 10^{-14}$ | $8.82 \times 10^{-7}$ | |
| 1.35 | $4.13 \times 10^{-14}$ | $2.09 \times 10^{-6}$ | |
| 1.67 | $8.64 \times 10^{-14}$ | $4.93 \times 10^{-6}$ | $5.00 \times 10^{7}$ [b] |
| 1.97 | $1.72 \times 10^{-13}$ | $9.75 \times 10^{-6}$ | |
| 2.29 | $3.60 \times 10^{-13}$ | $1.74 \times 10^{-5}$ | |
| 2.48 | $5.58 \times 10^{-13}$ | $2.71 \times 10^{-5}$ | |

[a]First order rate constants for C(3)-H/D exchange ($k_{ex}$, $s^{-1}$) were obtained as the slopes of plots shown in Figure S20. [b]The second-order rate constant ($k_{DO}$, $M^{-1}s^{-1}$) was obtained from the fit of log $k_{ex}$ – p*D* data to Eq 2 (Figure S27).

Table S9. First and second-order rate constants for exchange of the C(3)-H of triazolium salt **9c** for deuterium in solutions of DCl in $D_2O$ at 25 ºC and I = 1.0 M (KCl).

| p*D* | $[DO^-]$, M | $k_{ex}$, $s^{-1}$ [a] | $k_{DO}$, $M^{-1}s^{-1}$ |
|---|---|---|---|
| 0.72 | $9.70 \times 10^{-15}$ | $4.01 \times 10^{-7}$ | |
| 1.00 | $1.85 \times 10^{-14}$ | $8.40 \times 10^{-7}$ | |
| 1.34 | $4.04 \times 10^{-14}$ | $2.10 \times 10^{-6}$ | $4.39 \times 10^{7}$ [b] |
| 1.68 | $8.84 \times 10^{-14}$ | $4.67 \times 10^{-6}$ | |
| 1.99 | $1.80 \times 10^{-13}$ | $9.17 \times 10^{-6}$ | |
| 2.45 | $5.20 \times 10^{-13}$ | $2.80 \times 10^{-5}$ | |

[a]First order rate constants for C(3)-H/D exchange ($k_{ex}$, $s^{-1}$) were obtained as the slopes of plots shown in Figure S21. [b]The second-order rate constant ($k_{DO}$, $M^{-1}s^{-1}$) was obtained from the fit of log $k_{ex}$ – p*D* data to Eq 2 (Figure S27).

Table S10. First and second-order rate constants for exchange of the C(3)-H of triazolium salt **7k** for deuterium in solutions of DCl in $D_2O$ at 25 ºC and I = 1.0 M (KCl).

| p*D* | [DO$^-$], M | $k_{ex}$, s$^{-1}$ $^a$ | Results of Fitting to Eq 3 |
|---|---|---|---|
| 0.48 | 5.58 x $10^{-15}$ | 3.62 x $10^{-5}$ | |
| 0.67 | 8.64 x $10^{-15}$ | 5.16 x $10^{-5}$ | |
| 0.93 | 1.57 x $10^{-14}$ | 5.82 x $10^{-5}$ | $k_{DO}$ = 3.52 x $10^8$ M$^{-1}$s$^{-1}$ $^b$ |
| 1.32 | 3.86 x $10^{-14}$ | 7.34 x $10^{-5}$ | $k'_{DO}$ = 1.38 x $10^{10}$ M$^{-1}$s$^{-1}$ $^c$ |
| 1.58 | 7.03 x $10^{-14}$ | 8.83 x $10^{-5}$ | $K_a^{N1}$ = 0.35 M $^d$ |
| 1.76 | 1.06 x $10^{-13}$ | 1.09 x $10^{-4}$ | |
| 2.39 | 4.53 x $10^{-13}$ | 2.29 x $10^{-4}$ | |

[a]First order rate constants for C(3)-H/D exchange ($k_{ex}$, s$^{-1}$) were obtained as the slopes of plots shown in Figure S22. [b]The second-order rate constant ($k_{DO}$, M$^{-1}$s$^{-1}$) was obtained from the fit of log $k_{ex}$ – p*D* data to Eq 3 (Figure S28). [c]Values of $k'_{DO}$ (M$^{-1}$s$^{-1}$) obtained by fitting log $k_{ex}$ - p*D* data to equation 3 (Figure S28). [d]Values of $K_a^{N1}$ (M) obtained by fitting log $k_{ex}$ - p*D* data to equation 3 (Figure S28).

Table S11. First and second-order rate constants for exchange of the C(3)-H of triazolium salt **8k** for deuterium in solutions of DCl in $D_2O$ at 25 ºC and I = 1.0 M (KCl).

| p*D* | [DO$^-$], M | $k_{ex}$, s$^{-1}$ $^a$ | Results of Fitting to Eq 3 |
|---|---|---|---|
| 0.45 | 5.21 x $10^{-15}$ | 1.69 x $10^{-5}$ | |
| 0.67 | 8.64 x $10^{-15}$ | 2.27 x $10^{-5}$ | |
| 0.99 | 1.81 x $10^{-14}$ | 2.85 x $10^{-5}$ | $k_{DO}$ = 4.16 x $10^8$ M$^{-1}$s$^{-1}$ $^b$ |
| 1.35 | 4.13 x $10^{-14}$ | 4.10 x $10^{-5}$ | $k'_{DO}$ = 7.87 x $10^9$ M$^{-1}$s$^{-1}$ $^c$ |
| 1.60 | 7.36 x $10^{-14}$ | 5.28 x $10^{-5}$ | $K_a^{N1}$ = 0.55 M $^d$ |
| 1.78 | 1.11 x $10^{-13}$ | 6.91 x $10^{-5}$ | |
| 2.31 | 3.77 x $10^{-13}$ | 1.87 x $10^{-4}$ | |

[a]First order rate constants for C(3)-H/D exchange ($k_{ex}$, s$^{-1}$) were obtained as the slopes of plots shown in Figure S23. [b]The second-order rate constant ($k_{DO}$, M$^{-1}$s$^{-1}$) was obtained from the fit of log $k_{ex}$ – p*D* data to Eq 3 (Figure S28). [c]Values of $k'_{DO}$ (M$^{-1}$s$^{-1}$) obtained by fitting log $k_{ex}$ - p*D* data to equation 3 (Figure S28). [d]Values of $K_a^{N1}$ (M) obtained by fitting log $k_{ex}$ - p*D* data to equation 3 (Figure S28).

Table S12. First and second-order rate constants for exchange of the C(3)-H of triazolium salt **9k** for deuterium in solutions of DCl in $D_2O$ at 25 ºC and I = 1.0 M (KCl).

| p*D* | [$DO^-$], M | $k_{ex}$, $s^{-1}$ [a] | Results of Fitting to Eq 3 |
|---|---|---|---|
| 0.45 | $5.21 \times 10^{-15}$ | $1.75 \times 10^{-5}$ | |
| 0.66 | $8.45 \times 10^{-15}$ | $2.30 \times 10^{-5}$ | |
| 0.94 | $1.61 \times 10^{-14}$ | $3.01 \times 10^{-5}$ | $k_{DO} = 4.04 \times 10^8$ $M^{-1}s^{-1}$ [b] |
| 1.35 | $4.13 \times 10^{-14}$ | $4.01 \times 10^{-5}$ | $k'_{DO} = 7.37 \times 10^9$ $M^{-1}s^{-1}$ [c] |
| 1.60 | $7.36 \times 10^{-14}$ | $5.61 \times 10^{-5}$ | $K_a^{N1} = 0.46$ M [d] |
| 1.78 | $1.11 \times 10^{-13}$ | $7.32 \times 10^{-5}$ | |
| 2.36 | $4.23 \times 10^{-13}$ | $2.00 \times 10^{-4}$ | |

[a]First order rate constants for C(3)-H/D exchange ($k_{ex}$, $s^{-1}$) were obtained as the slopes of plots shown in Figure S24. [b]The second-order rate constant ($k_{DO}$, $M^{-1}s^{-1}$) was obtained from the fit of log $k_{ex}$ – p*D* data to Eq 3 (Figure S28). [c]Values of $k'_{DO}$ ($M^{-1}s^{-1}$) obtained by fitting log $k_{ex}$ - p*D* data to equation 3 (Figure S28). [d]Values of $K_a^{N1}$ (M) obtained by fitting log $k_{ex}$ - *p*D data to equation 3 (Figure S28).

## S1.5 Hammett Analysis of Protofugalities ($k_{DO}$).

Figure S29. Semilogarithmic plots of log $k_{DO}$ against Hammett substituent constant, σ, for triazolium salts **7a-c, 7k (■); 8a-c, 8k (●); 9a-c, 9k (▲)**.

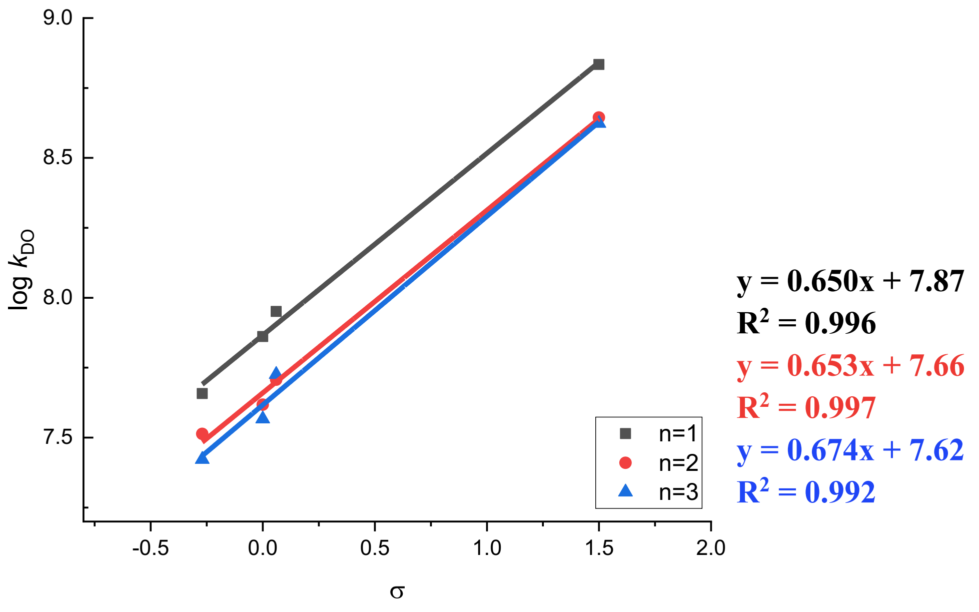

Table S13. Hammett substituent constants, $\sigma$

| | $\sigma_m$ or $\sigma_p$[a] |
|---|---|
| 4-MeO | -0.27 |
| H | 0 |
| 4-F | 0.06 |
| 4-Br | 0.23 |
| 3-Cl | 0.37 |
| 4-$CF_3$ | 0.54 |
| 2,6-Di-MeO | -0.54[b] |
| 2,4,6-Tri-$^i$Pr | -0.45[b] |
| 2,4,6-Tri-Me | -0.51[b] |
| 2,4,6-Tri-Cl | 0.69[b] |
| $F_5$ | 1.50[c] |

[a]Taken from Hansch, Leo and Taft.[S6] [b] For the purpose of ordering of *ortho*-substituted examples in Figure 3, we have assumed an additive substituent effect in the absence of available literature N-aryl substituent constants.[S7] [c]Based on Taft's Hammett $\sigma$ value.[S8]

## S1.6 Single-crystal X-ray Crystallography.

The crystal structures of **7a**, **7b**, **7g** and **7k** have been reported previously (the CCDC codes are ISOXEU [1], ISOXIY [1], LEBMIQ [2] and ISOXOE [1] respectively)[S1g-h] but re-determination of these structures was deemed to be necessary for consistency in analysis.

The X-ray single crystal data for compounds **7a**, **7c**, **7d**, **7e**, **7f**, **7g**, **7j**, **7ja**, **7k**, **8a**, **8b**, **8c**, **8i**, **9a** and **9k** have been collected on a Bruker D8Venture (Photon100 CMOS detector, IμS-microsource, focusing mirrors) 3-circle diffractometer. The data for compounds **7h**and **9c** were collected on an Oxford Diffraction , Gemini-Ultra 4-circle diffractometer (Atlas CCD detector, fine-focus sealed tube, graphite monochromator). The data for compound **8k** were collected on a Bruker D8Venture (Photon 2 CMOS detector, IμS-microsource, focusing mirrors) 3-circle diffractometer. The data for compound **7i** were collected on an Agilent XCalibur 4-circle diffractometer (Sapphire-3 CCD detector, fine-focus sealed tube, graphite monochromator); for compound **7b** on a Bruker D8Venture (Photon III MM C7 CPAD detector, IμS microsource, focusing mirrors) 3-circle diffractometer; for compound **9b** on a Bruker D8Venture (Photon III MM C14 CPAD detector, IμS III microsource, focusing mirrors) 3-circle diffractometer and for compound **8a** on a Bruker D8Venture (Photon II CPAD detector, fine-focus sealed tube, focusing mirrors) 3-circle diffractometer.

The sources with λ CuKα = 1.54184 Å radiation were used for compounds **7h**, **8k**, **9b** and **9c**; in all other cases λMoKα radiation (λ = 0.71073Å) was used. The temperature on the crystals (150.0(2)K for compounds **7h**, **8k** and **9c**; 120.0(2)K for all other compounds) was maintained by Oxford Cryosystems CryostreamPlus open-flow $N_2$ cooling devices. Corresponding instrument's software was used for cell refinement, data collection, data reduction and empirical absorption correction. All structures were solved by either direct or intrinsic phase methods and refined by full-matrix least squares on $F^2$ for all data using Olex2 [3] and

SHELXTL [4] software. All non-disordered non-hydrogen atoms were refined in anisotropic approximation, the hydrogen atoms in most of the structures were placed in the calculated positions and refined in riding mode. Several structures (**7b**, **7c**, **7g**, **7i**, **7k**, **8c** and **9b**) contain disordered fragments (usually $BF_4^-$ anions). Various appropriate constrains and restrains were used in refinement of these fragments. Two polymorphs of the compound **7j** were found in different available samples. Crystal data and parameters of refinement are listed in Tables S14-16. Crystallographic data for the structures have been deposited with the Cambridge Crystallographic Data Centre as supplementary publications CCDC 2124937-2124950; 2124952-2124958.

### S1.6.1 Analysis of Triazolium Salts 7h, 8k and 9c via Encapsulated Nanodroplet Crystallisation (ENaCt)/Single Crystal X-ray Diffraction

The growth of single crystals of **7h**, **8k** and **9c**, suitable for single crystal X-ray diffraction analysis was performed via a modified high-throughput Encapsulated Nanodroplet Crystallisation (ENaCt) approach.[S9] Stock solutions of **7h**, **8k** and **9c** were prepared in DMSO and DMF, droplets of which (50 nL) were then dispensed via an STP Labtech Mosquito liquid-handling robot into 96 well glass plates (SWISSCI LCP Modular, 100 μm spacer) containing an appropriate crystallization oil (50-300 nL of PDMSO, FC40, FYR or mineral oil). Experiments were performed with or without the addition of a secondary solvent (25-250 nL of $H_2O$, toluene, butanol, chlorobenzene or 2-methyl-2,4-pentanediol (MPD)). The plates were sealed with a glass cover slip, allowed to stand at room temperature in the dark and assessed visually and by cross-polarized light microscopy for crystal growth every few days. After 7 days, wells containing crystals were opened and suitable crystals of **7h**, **8k** and **9c** selected for single crystal X-ray analysis. Specific ENaCt crystallisation conditions for each molecule are

given below: **7h** 50 nL DMF (33 mg/mL) in 200 nL of FYR; **8k** 50 nL DMSO (50 mg/mL), 100 nL 2-methyl-2,4-pentanediol (MPD) in 200 nL of FYR; **9c** 50 nL DMSO (50 mg/mL) in 150 nL of FC40.

### S1.6.2 Crystal Data and Structure Refinement Parameters

Table S14. Crystal data and structure refinement parameters.

| Compound | **7a** | **7b** | **7c** | **7d** | **7e** | **7f** | **7g** |
|---|---|---|---|---|---|---|---|
| Empirical formula | $C_{12}H_{14}N_3OBF_4$ | $C_{11}H_{12}BF_4N_3$ | $C_{11}H_{11}FN_3BF_4$ | $C_{11}H_{11}BrN_3BF_4$ | $C_{11}H_{11}N_3ClBF_4$ | $C_{12}H_{11}F_3N_3BF_4$ | $C_{13}H_{16}N_3O_2BF_4$ |
| Formula weight | 303.07 | 273.05 | 291.04 | 351.95 | 307.49 | 341.05 | 333.10 |
| Temperature/K | 120.0 | 120.0 | 120.0 | 120.0 | 120.0 | 120.0 | 120.0 |
| Crystal system | orthorhombic | orthorhombic | orthorhombic | monoclinic | monoclinic | monoclinic | orthorhombic |
| Space group | Iba2 | $P2_12_12_1$ | Pbca | $P2_1/n$ | $P2_1/c$ | $P2_1/n$ | Pbca |
| a/Å | 10.614(3) | 6.7732(3) | 7.8707(4) | 7.0877(3) | 9.0461(9) | 7.0168(4) | 10.5264(4) |
| b/Å | 34.391(11) | 10.5577(4) | 10.9345(5) | 12.1656(5) | 8.0475(8) | 12.7166(7) | 13.0742(4) |
| c/Å | 7.410(2) | 17.0417(7) | 29.0299(14) | 15.7597(7) | 17.6103(17) | 16.1197(9) | 21.4933(7) |
| α/° | 90 | 90 | 90 | 90 | 90 | 90 | 90 |
| β/° | 90 | 90 | 90 | 100.3689(15) | 93.866(4) | 101.332(2) | 90 |
| γ/° | 90 | 90 | 90 | 90 | 90 | 90 | 90 |
| Volume/Å$^3$ | 2704.8(15) | 1218.64(9) | 2498.4(2) | 1336.71(10) | 1279.1(2) | 1410.32(14) | 2958.00(17) |
| Z | 8 | 4 | 8 | 4 | 4 | 4 | 8 |
| $\rho_{calc}$g/cm$^3$ | 1.489 | 1.488 | 1.547 | 1.749 | 1.597 | 1.606 | 1.496 |
| μ/mm$^{-1}$ | 0.133 | 0.133 | 0.146 | 3.114 | 0.339 | 0.162 | 0.134 |
| F(000) | 1248.0 | 560.0 | 1184.0 | 696.0 | 624.0 | 688.0 | 1376.0 |
| Radiation | MoKα | MoKα | MoKα | MoKα | MoKα | MoKα | MoKα |
| Reflections collected | 17438 | 15661 | 35825 | 28802 | 25412 | 26012 | 43211 |
| Independent refl. $R_{int}$ | 2953, 0.1164, | 3538, 0.0439 | 3321, 0.0378 | 3866, 0.0287 | 3398, 0.0598 | 3236, 0.0633 | 3924, 0.0445 |
| Data/restraints/parameters | 2953/1/192 | 3538/10/185 | 3321/28/241 | 3866/0/181 | 3398/0/225 | 3236/15/248 | 3924/0/252 |
| Goodness-of-fit on F$^2$ | 1.003 | 1.031 | 1.035 | 1.036 | 1.025 | 1.045 | 1.025 |
| Final $R_1$ [I≥2σ (I)] | 0.0583 | 0.0514 | 0.0488 | 0.0213 | 0.0462 | 0.0618 | 0.0391 |
| Final $wR_2$ [all data] | 0.1317 | 0.1167 | 0.1272 | 0.0567 | 0.1213 | 0.1560 | 0.0997 |
| Largest diff. peak/hole,eÅ$^{-3}$ | 0.27/-0.23 | 0.23/-0.21 | 0.43/-0.35 | 0.51/-0.36 | 0.51/-0.61 | 1.19/-0.63 | 0.28/-0.23 |
| Flack parameter | 0.2(8) | 0.0(4) | n/a | n/a | n/a | n/a | n/a |

Table S15. Crystal data and structure refinement parameters (cont.)

| Compound | **7h** | **7i** | **7j** | **7ja** | **7k** | **8a** | **8b** |
|---|---|---|---|---|---|---|---|
| Empirical formula | $C_{20}H_{32.95}ClN_3O_{1.48}$ | $C_{14}H_{18}N_3BF_4$ | $C_{11}H_9Cl_3N_3BF_4$ | $C_{11}H_9Cl_3N_3BF_4$ | $C_{11}H_7F_5N_3BF_4$ | $C_{13}H_{16}N_3OBF_4$ | $C_{12}H_{14}N_3BF_4$ |
| Formula weight | 374.58 | 315.12 | 376.37 | 376.37 | 363.01 | 317.10 | 287.07 |
| Temperature/K | 150.0 | 120.0 | 120.0 | 120.0 | 120.0 | 120.0 | 120.0 |
| Crystal system | triclinic | monoclinic | monoclinic | orthorhombic | monoclinic | orthorhombic | orthorhombic |
| Space group | P-1 | $P2_1/n$ | $P2_1/n$ | $Pca2_1$ | $P2_1$ | $Pca2_1$ | $Pca2_1$ |
| a/Å | 8.0473(4) | 9.4928(6) | 9.6158(5) | 12.2618(8) | 8.2073(9) | 10.7585(10) | 11.0268(10) |
| b/Å | 8.4438(4) | 7.8963(3) | 7.9497(5) | 6.9776(5) | 7.6843(8) | 18.0168(17) | 15.7797(13) |
| c/Å | 16.4764(9) | 20.5873(12) | 19.1105(11) | 34.736(2) | 10.7857(11) | 7.4206(7) | 7.5490(7) |
| α/° | 77.912(4) | 90.00 | 90 | 90 | 90 | 90 | 90 |
| β/° | 83.956(4) | 94.597(5) | 91.871(2) | 90 | 101.768(4) | 90 | 90 |
| γ/° | 83.405(4) | 90.00 | 90 | 90 | 90 | 90 | 90 |
| Volume/Å$^3$ | 1083.69(10) | 1538.22(14) | 1460.08(15) | 2971.9(3) | 665.93(12) | 1438.4(2) | 1313.5(2) |
| Z | 2 | 4 | 4 | 8 | 2 | 4 | 4 |
| $\rho_{calc}$g/cm$^3$ | 1.148 | 1.361 | 1.712 | 1.682 | 1.810 | 1.464 | 1.452 |
| μ/mm$^{-1}$ | 1.666 | 0.115 | 0.668 | 0.656 | 0.198 | 0.129 | 0.127 |
| F(000) | 406.0 | 656.0 | 752.0 | 1504.0 | 360.0 | 656.0 | 592.0 |
| Radiation | CuKα | MoKα | MoKα | MoKα | MoKα | MoKα | MoKα |
| Reflections collected | 14921 | 20156 | 22432 | 30024 | 9298 | 18591 | 16998 |
| Independent refl. $R_{int}$ | 3826, 0.0572 | 3525, 0.0931 | 4060, 0.0277 | 7055, 0.0712 | 3197, 0.0483 | 3141, 0.0767 | 2858, 0.0767 |
| Data/restraints/parameters | 3826/195/256 | 3525/28/214 | 4060/0/235 | 7055/1/397 | 3197/7/216 | 3141/1/200 | 2858/1/181 |
| Goodness-of-fit on F$^2$ | 1.028 | 1.047 | 1.050 | 1.039 | 1.031 | 1.029 | 1.007 |
| Final $R_1$ [I≥2σ (I)] | 0.0447 | 0.0684 | 0.0259 | 0.0619 | 0.0485 | 0.0464 | 0.0430 |
| Final $wR_2$ [all data] | 0.1170 | 0.2134 | 0.0668 | 0.1246 | 0.1174 | 0.1006 | 0.0937 |
| Largest diff. peak/hole,eÅ$^{-3}$ | 0.25/-0.22 | 0.43/-0.28 | 0.39/-0.36 | 0.51/-0.42 | 0.35/-0.36 | 0.19/-0.23 | 0.17/-0.19 |
| Flack parameter | n/a | n/a | n/a | 0.05(5) | 0.2(4) | -0.4(6) | -0.1(6) |

Table S16. Crystal data and structure refinement parameters (cont.)

| Compound | **8c** | **8i** | **8k** | **9a** | **9b** | **9c** | **9k** |
|---|---|---|---|---|---|---|---|
| Empirical formula | $C_{12}H_{13}FN_3BF_4$ | $C_{15}H_{20}N_3BF_4$ | $C_{12}H_9F_5N_3BF_4$ | $C_{14}H_{18}N_3OBF_4$ | $C_{13}H_{16}N_3BF_4$ | $C_{13}H_{15}N_3BF_5$ | $C_{13}H_{11}F_5N_3BF_4$ |
| Formula weight | 305.06 | 329.15 | 377.03 | 331.12 | 301.10 | 319.09 | 391.06 |
| Temperature/K | 120.0 | 120.0 | 150.0 | 120.0 | 120.0 | 150.0 | 120.0 |
| Crystal system | orthorhombic | orthorhombic | orthorhombic | orthorhombic | monoclinic | orthorhombic | monoclinic |
| Space group | $Pca2_1$ | Pbca | $Pca2_1$ | $Pna2_1$ | C2/c | Pbca | $P2_1/n$ |
| a/Å | 11.4598(16) | 10.6066(9) | 22.5508(6) | 14.4005(8) | 65.320(2) | 11.7546(3) | 17.8389(8) |
| b/Å | 15.4396(19) | 7.7396(7) | 8.2325(2) | 11.7102(7) | 11.2304(4) | 7.59636(17) | 7.5945(3) |
| c/Å | 7.5533(10) | 39.222(3) | 7.8305(2) | 17.9974(10) | 7.7571(3) | 32.2135(9) | 23.1992(10) |
| α/° | 90 | 90 | 90 | 90 | 90 | 90 | 90 |
| β/° | 90 | 90 | 90 | 90 | 90.847(3) | 90 | 105.7240(17) |
| γ/° | 90 | 90 | 90 | 90 | 90 | 90 | 90 |
| Volume/Å$^3$ | 1336.4(3) | 3219.8(5) | 1453.73(6) | 3035.0(3) | 5689.8(4) | 2876.41(13) | 3025.4(2) |
| Z | 4 | 8 | 4 | 8 | 16 | 8 | 8 |
| $\rho_{calc}$g/cm$^3$ | 1.516 | 1.358 | 1.723 | 1.449 | 1.406 | 1.474 | 1.717 |
| μ/mm$^{-1}$ | 0.140 | 0.113 | 1.686 | 0.126 | 1.049 | 1.176 | 0.181 |
| F(000) | 624.0 | 1376.0 | 752.0 | 1376.0 | 2496.0 | 1312.0 | 1568.0 |
| Radiation | MoKα | MoKα | CuKα | MoKα | CuKα | CuKα | MoKα |
| Reflections collected | 15307 | 33230 | 12774 | 41084 | 38152 | 16278 | 45266 |
| Independent refl. $R_{int}$ | 2914, 0.0542 | 3332, 0.1113 | 2631, 0.0304 | 6971, 0.0663 | 5466, 0.1207 | 2540, 0.0427 | 8048, 0.0506 |
| Data/restraints/parameters | 2914/29/187 | 3332/0/209 | 2631/1/227 | 6971/1/417 | 5466/39/407 | 2540/0/200 | 8048/0/557 |
| Goodness-of-fit on $F^2$ | 1.030 | 1.049 | 1.075 | 1.014 | 1.027 | 1.036 | 1.032 |
| Final $R_1$ indexes [I≥2σ (I)] | 0.0873 | 0.0672 | 0.0292 | 0.0508 | 0.0795 | 0.0335 | 0.0480 |
| Final $wR_2$ [all data] | 0.2537 | 0.1434 | 0.0801 | 0.1221 | 0.2394 | 0.0880 | 0.1110 |
| Largest diff. peak/hole, e Å$^{-3}$ | 0.60/-0.65 | 0.32/-0.30 | 0.28/-0.18 | 0.44/-0.26 | 0.30/-0.29 | 0.23/-0.22 | 0.45/-0.44 |
| Flack parameter | 0.5(4) | n/a | 0.45(16) | 0.4(3) | n/a | n/a | n/a |

### S1.6.3 ORTEP Diagrams for X-ray Crystal Structures

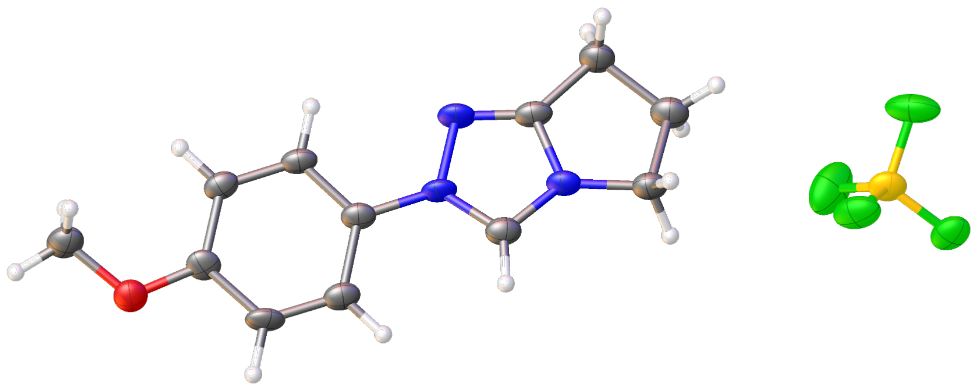

Figure S30. ORTEP of **7a** showing thermal ellipsoids at the 50% probability level.

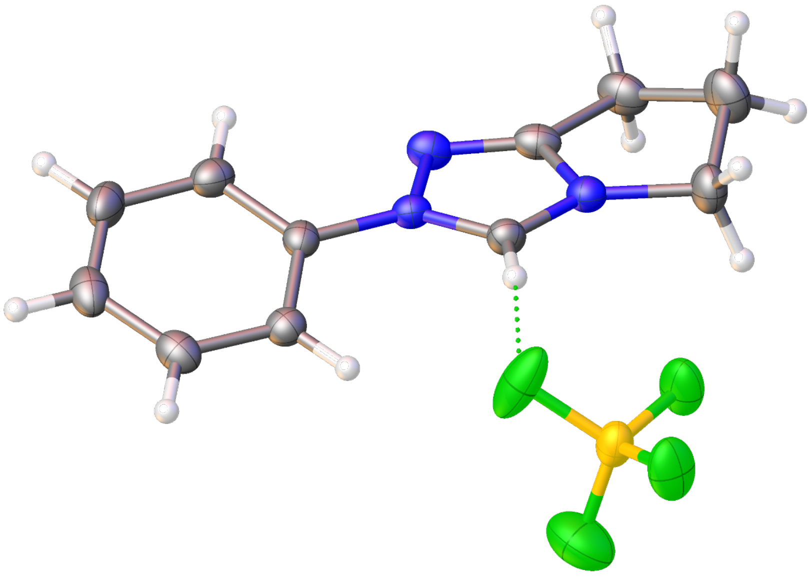

Figure S31. ORTEP of **7b** showing thermal ellipsoids at the 50% probability level.

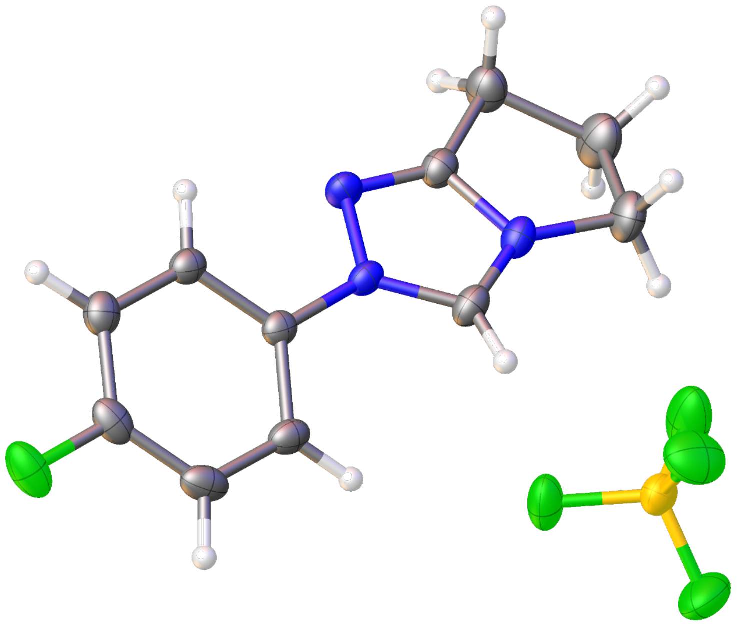

Figure S32. ORTEP of **7c** showing thermal ellipsoids at the 50% probability level.

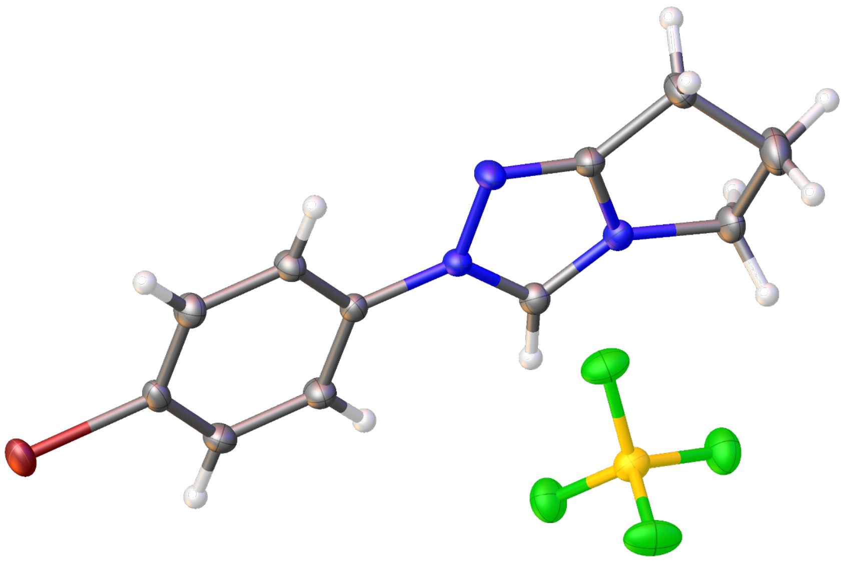

Figure S33. ORTEP of **7d** showing thermal ellipsoids at the 50% probability level.

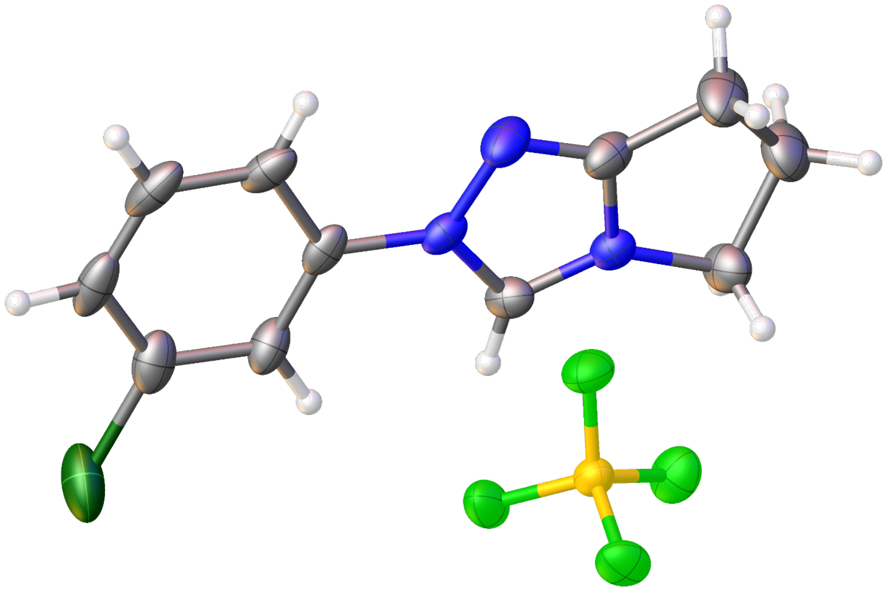

Figure S34. ORTEP of **7e** showing thermal ellipsoids at the 50% probability level.

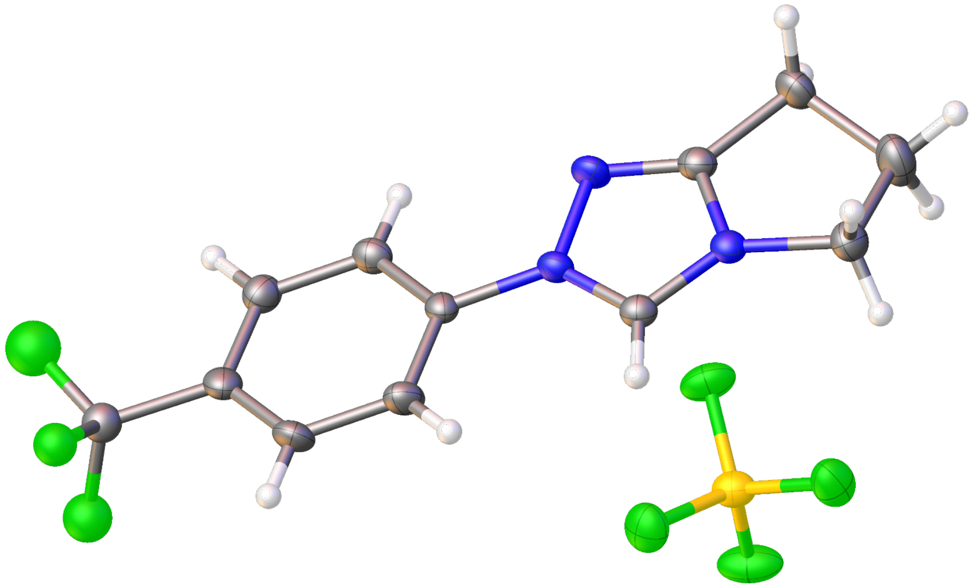

Figure S35. ORTEP of **7f** showing thermal ellipsoids at the 50% probability level.

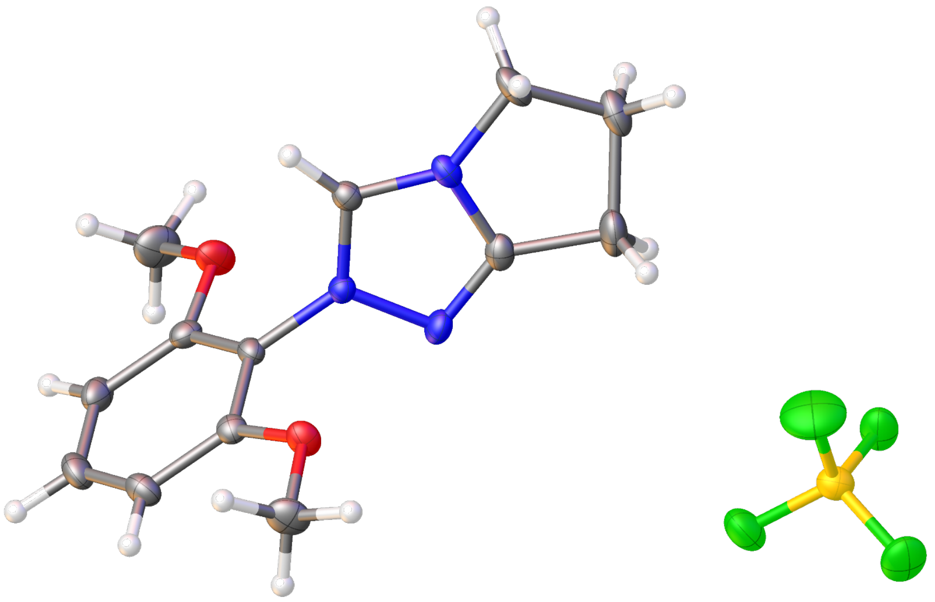

Figure S36. ORTEP of **7g** showing thermal ellipsoids at the 50% probability level.

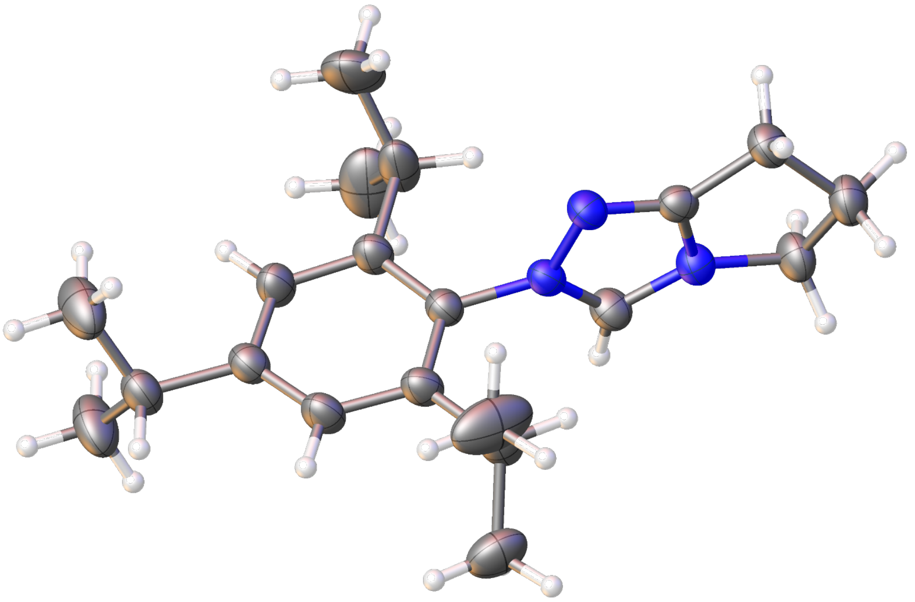

Figure S37. ORTEP of **7h** showing thermal ellipsoids at the 50% probability level.

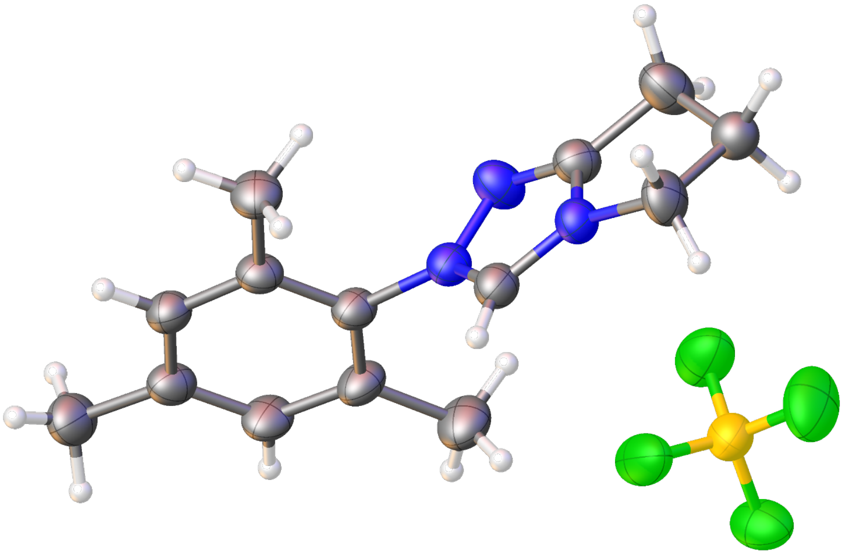

Figure S38. ORTEP of **7i** showing thermal ellipsoids at the 50% probability level.

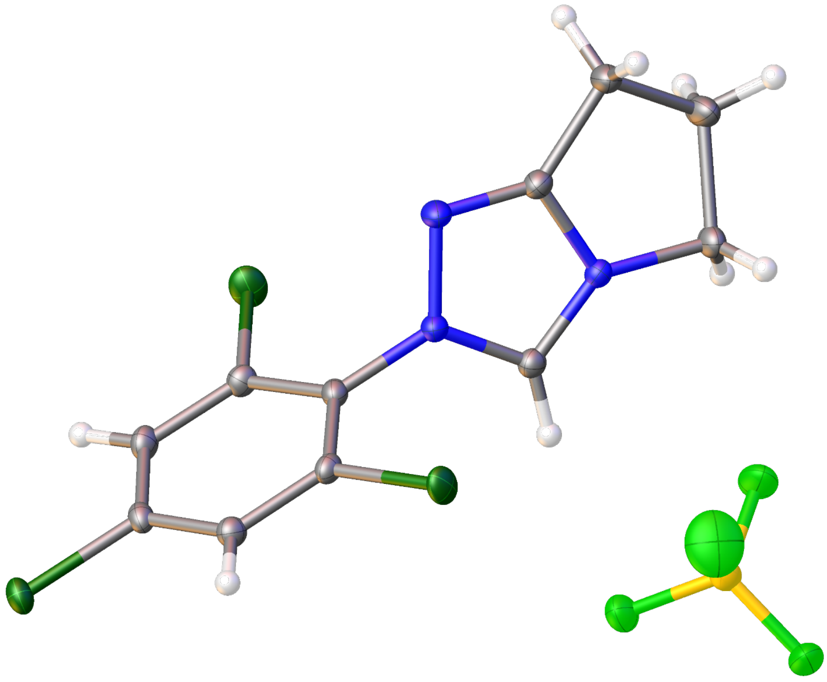

Figure S39. ORTEP of **7j** showing thermal ellipsoids at the 50% probability level.

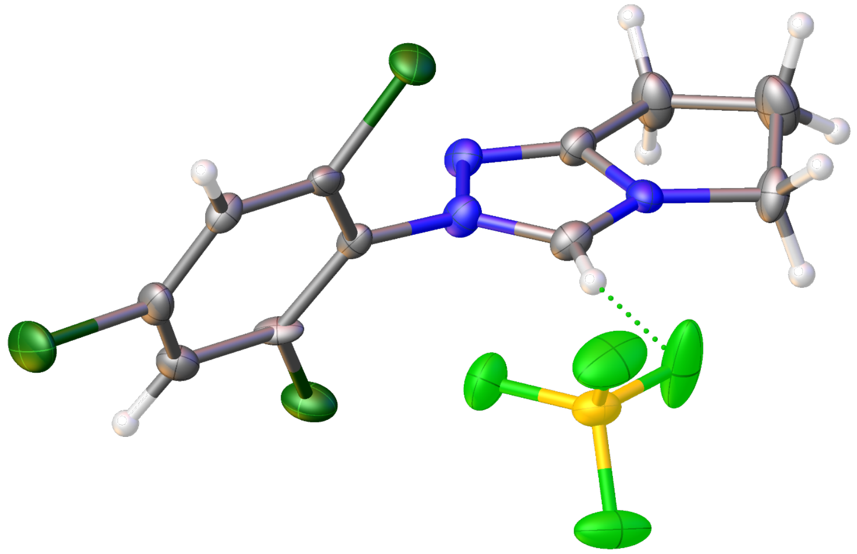

Figure S40.   ORTEP of **7ja** showing thermal ellipsoids at the 50% probability level.

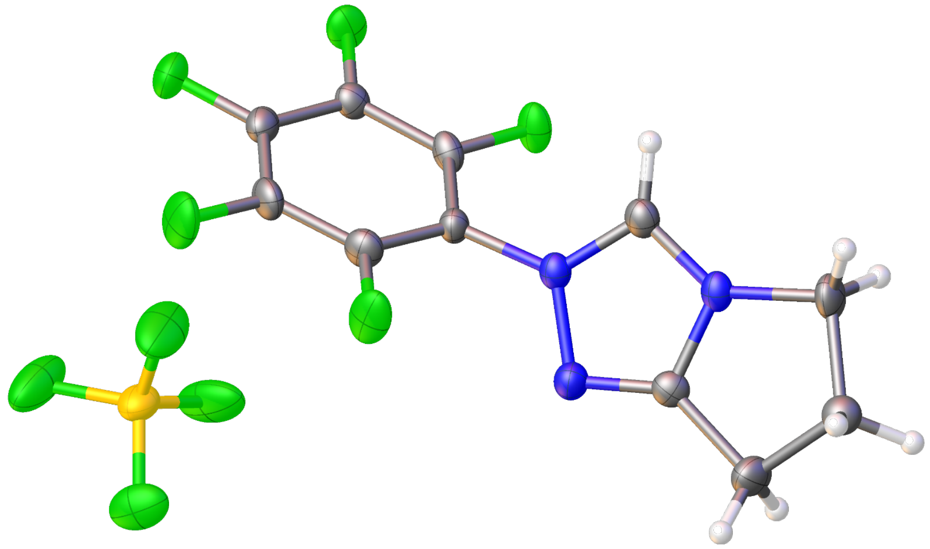

Figure S41.   ORTEP of **7k** showing thermal ellipsoids at the 50% probability level.

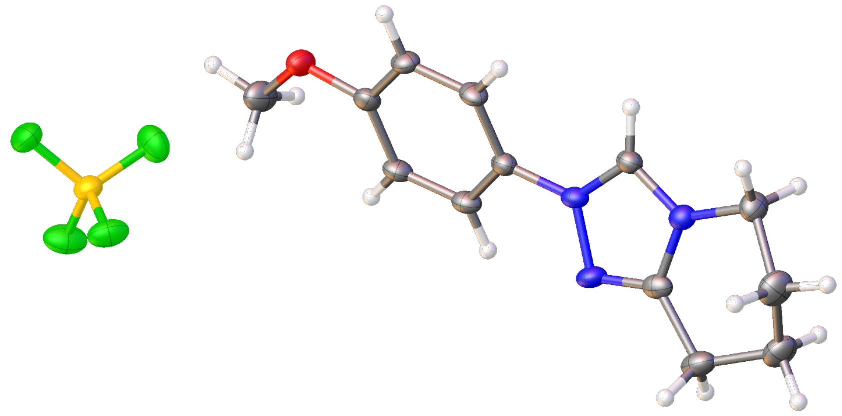

Figure S42. ORTEP of **8a** showing thermal ellipsoids at the 50% probability level.

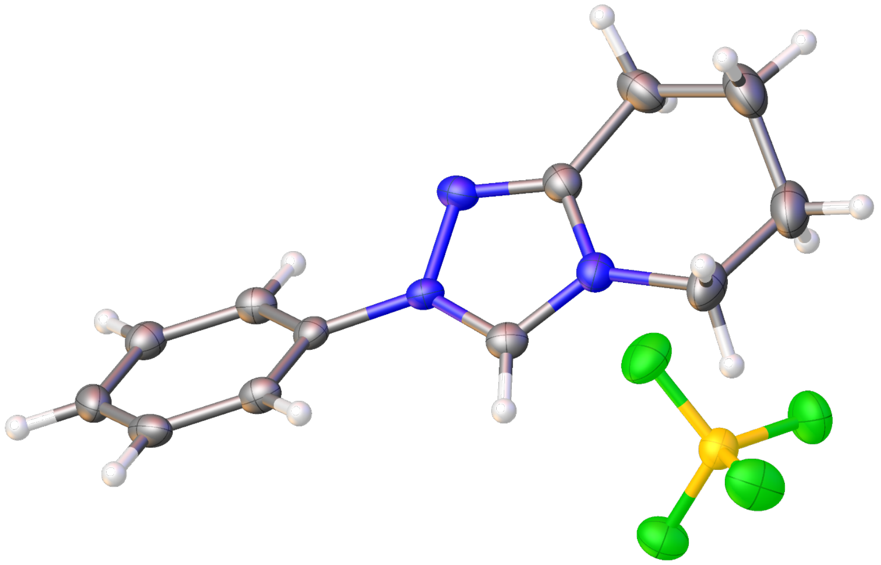

Figure S43. ORTEP of **8b** showing thermal ellipsoids at the 50% probability level.

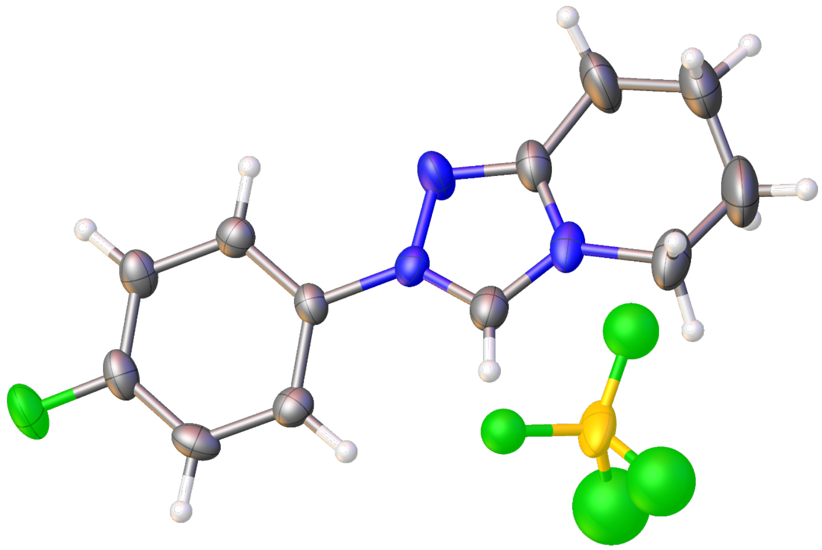

Figure S44. ORTEP of **8c** showing thermal ellipsoids at the 50% probability level.

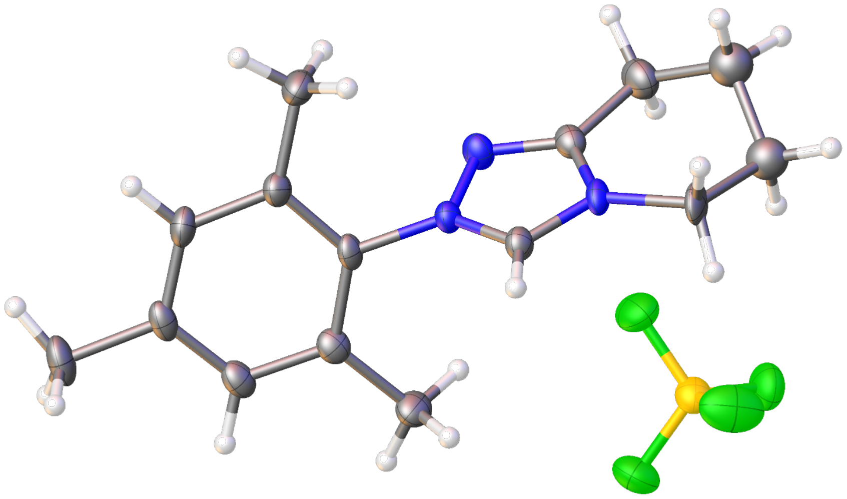

Figure S45. ORTEP of **8i** showing thermal ellipsoids at the 50% probability level.

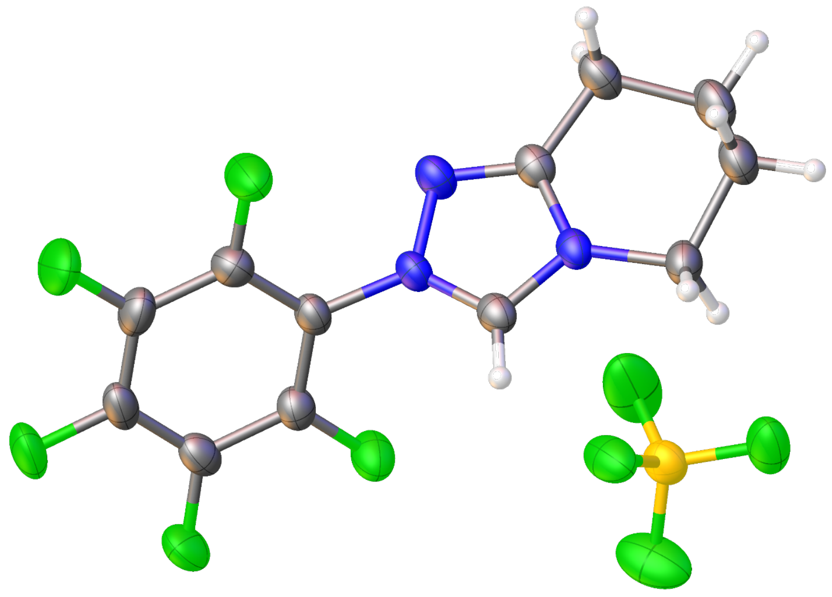

Figure S46. ORTEP of **8k** showing thermal ellipsoids at the 50% probability level.

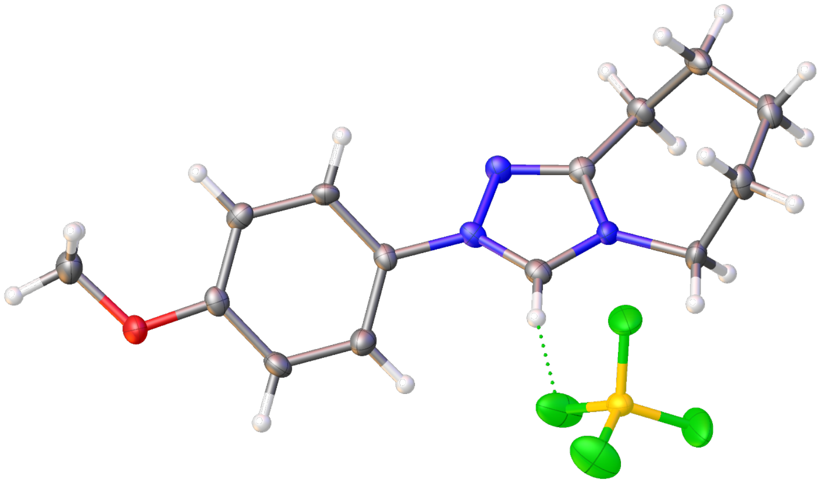

Figure S47. ORTEP of **9a** showing thermal ellipsoids at the 50% probability level.

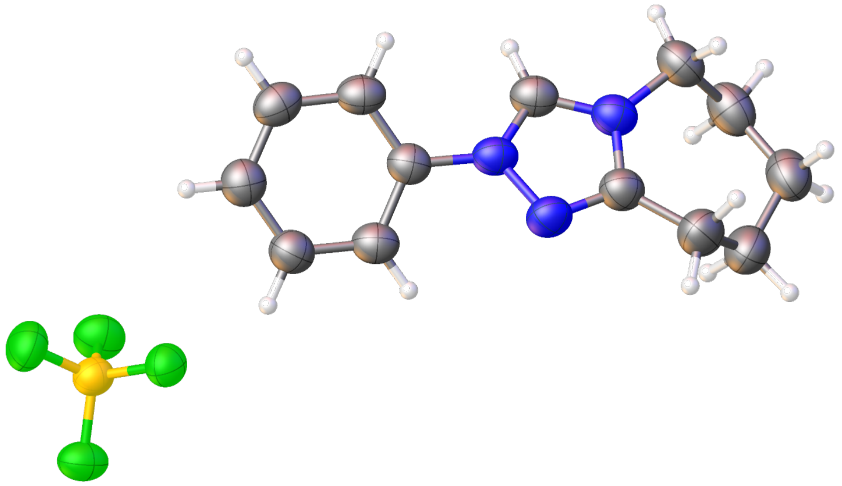

Figure S48.   ORTEP of **9b** showing thermal ellipsoids at the 50% probability level.

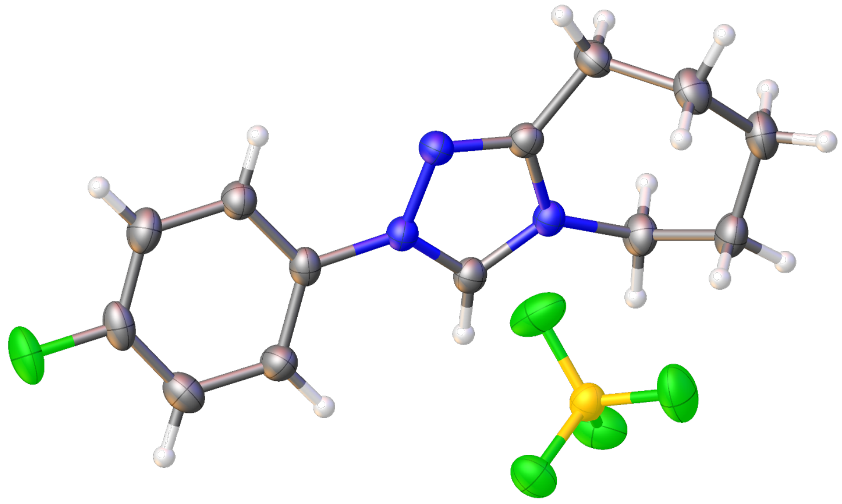

Figure S49.   ORTEP of **9c** showing thermal ellipsoids at the 50% probability level.

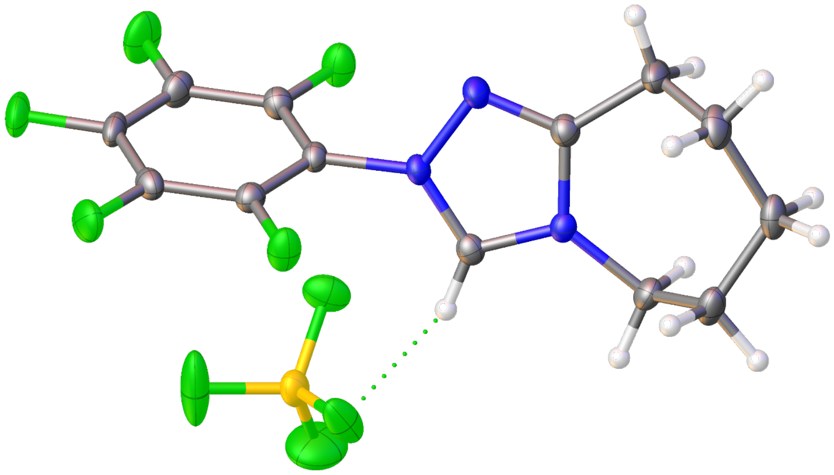

Figure S50. ORTEP of **9k** showing thermal ellipsoids at the 50% probability level.

### S1.6.4 Summary of Bond Lengths, Bond Angles, and Dihedral Angles

Table S17. Structural data for individual triazolium salts **7a-k** (n=1) obtained from single crystal X-ray diffraction measurements.

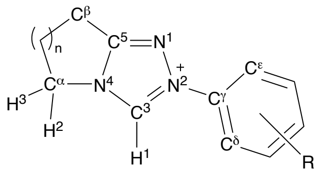


| Backbone Bond Length | **7a** | **7b** | **7c** | **7d** | **7e** | **7f** | **7g** | **7h** | **7i** | **7j** | **7k** |
|---|---|---|---|---|---|---|---|---|---|---|---|
| $N^1N^2$ | 1.38 | 1.38 | 1.38 | 1.38 | 1.39 | 1.39 | 1.38 | 1.39 | 1.39 | 1.38 | 1.38 |
| $N^2C^3$ | 1.32 | 1.33 | 1.33 | 1.33 | 1.33 | 1.32 | 1.32 | 1.31 | 1.32 | 1.32 | 1.31 |
| $C^3N^4$ | 1.33 | 1.33 | 1.32 | 1.33 | 1.32 | 1.32 | 1.32 | 1.33 | 1.33 | 1.32 | 1.32 |
| $N^4C^5$ | 1.36 | 1.37 | 1.36 | 1.36 | 1.37 | 1.36 | 1.35 | 1.36 | 1.35 | 1.35 | 1.36 |
| $C^5N^1$ | 1.30 | 1.30 | 1.30 | 1.30 | 1.30 | 1.30 | 1.30 | 1.30 | 1.31 | 1.30 | 1.30 |
| $C^5C^\beta$ | 1.47 | 1.49 | 1.49 | 1.49 | 1.49 | 1.49 | 1.49 | 1.49 | 1.49 | 1.49 | 1.50 |
| $N^4C^\alpha$ | 1.46 | 1.48 | 1.47 | 1.48 | 1.48 | 1.48 | 1.47 | 1.47 | 1.48 | 1.47 | 1.47 |
| $N^2C^\gamma$ | 1.43 | 1.43 | 1.43 | 1.43 | 1.43 | 1.43 | 1.43 | 1.45 | 1.44 | 1.43 | 1.43 |
| $C^\gamma C^\delta$ | 1.39 | 1.39 | 1.39 | 1.39 | 1.40 | 1.38 | 1.39 | 1.40 | 1.40 | 1.38 | 1.38 |
| $C^\gamma C^\varepsilon$ | 1.37 | 1.39 | 1.39 | 1.39 | 1.39 | 1.39 | 1.40 | 1.39 | 1.39 | 1.38 | 1.38 |
| **C-H Bond Length** | | | | | | | | | | | |
| $C^3H^1$ | 0.95 | 0.95 | 0.95 | 0.95 | 0.90 | 0.93 | 0.96 | 0.95 | 0.95 | 0.91 | 0.95 |
| $C^\alpha H^2$ | 0.99 | 0.99 | 0.97 | 0.99 | 0.95 | 0.93 | 0.99 | 0.99 | 0.99 | 0.92 | 0.99 |
| $C^\alpha H^3$ | 0.99 | 0.99 | 0.99 | 0.99 | 0.94 | 0.97 | 0.99 | 0.99 | 0.99 | 0.93 | 0.99 |
| **H-H Distance** | | | | | | | | | | | |
| $H^1H^2$ | 3.06 | 3.09 | 3.04 | 3.08 | 3.02 | 2.99 | 3.12 | 3.12 | 3.08 | 3.04 | 3.07 |
| $H^1H^3$ | 3.30 | 3.33 | 3.23 | 3.34 | 3.24 | 3.24 | 3.32 | 3.28 | 3.34 | 3.22 | 3.32 |
| **Bond Angles** | | | | | | | | | | | |
| $C^5N^1N^2$ | 102.8 | 103.2 | 103.0 | 102.9 | 102.5 | 102.7 | 102.6 | 102.5 | 102.8 | 103.2 | 102.3 |
| $N^1N^2C^3$ | 112.3 | 111.8 | 111.6 | 111.8 | 112.2 | 111.8 | 112.0 | 112.0 | 111.4 | 111.3 | 112.5 |
| $N^2C^3N^4$ | 105.6 | 105.9 | 106.2 | 105.9 | 105.9 | 106.2 | 106.0 | 106.4 | 106.3 | 106.4 | 105.9 |
| $C^3N^4C^5$ | 107.5 | 107.5 | 107.7 | 107.5 | 107.5 | 107.3 | 107.4 | 106.8 | 107.6 | 107.4 | 107.2 |
| $N^4C^5N^1$ | 111.8 | 111.6 | 111.6 | 111.9 | 111.9 | 112.0 | 112.0 | 112.2 | 111.9 | 111.6 | 112.1 |
| $C^\beta C^5N^4$ | 110.7 | 110.3 | 110.9 | 110.9 | 110.9 | 110.7 | 110.9 | 110.3 | 111.2 | 110.2 | 109.6 |
| $C^5N^4C^\alpha$ | 114.6 | 113.5 | 113.6 | 113.6 | 113.5 | 113.5 | 113.5 | 114.2 | 113.4 | 113.4 | 114.1 |
| **Torsion Angles** | | | | | | | | | | | |
| $H^1C^3*C^\alpha H^2$ | 44.4 | 41.7 | 46.3 | 42.3 | 46.0 | 40.7 | 43.6 | 45.5 | 41.9 | 43.9 | 42.9 |
| $H^1C^3*C^\alpha H^3$ | 68.4 | 71.0 | 68.1 | 70.4 | 67.9 | 72.5 | 68.8 | 67.1 | 70.9 | 70.4 | 69.6 |
| $N^1N^2*C^\gamma C^\delta$ | 16.1 | 19.9 | 25.2 | 3.8 | 73.7 | 4.1 | 76.6 | 91.2 | 79.9 | 2.8 | 62.1 |
| $N^1N^2*C^\gamma C^\varepsilon$ | 163.9 | 160.5 | 155.2 | 177.4 | 105.6 | 175.7 | 102.3 | 88.6 | 97.9 | 177.0 | 122.9 |

Table S18. Structural data for individual triazolium salts **8a-c, i, k** (n=2) and **9a-c**, **k** (n=3) obtained from single crystal X-ray diffraction measurements.

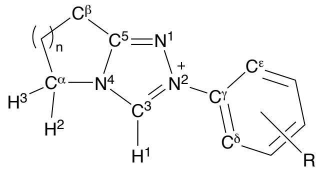


| Backbone Bond Length | **8a** | **8b** | **8c** | **8i** | **8k** | **9a** | **9b** | **9c** | **9k** |
|---|---|---|---|---|---|---|---|---|---|
| $N^1N^2$ | 1.38 | 1.38 | 1.37 | 1.38 | 1.38 | 1.37 | 1.38 | 1.37 | 1.38 |
| $N^2C^3$ | 1.32 | 1.31 | 1.31 | 1.31 | 1.32 | 1.31 | 1.32 | 1.32 | 1.31 |
| $C^3N^4$ | 1.34 | 1.33 | 1.33 | 1.33 | 1.33 | 1.33 | 1.32 | 1.33 | 1.32 |
| $N^4C^5$ | 1.36 | 1.37 | 1.37 | 1.36 | 1.37 | 1.37 | 1.37 | 1.38 | 1.37 |
| $C^5N^1$ | 1.31 | 1.30 | 1.32 | 1.30 | 1.31 | 1.31 | 1.31 | 1.31 | 1.31 |
| $C^5C^\beta$ | 1.49 | 1.49 | 1.48 | 1.49 | 1.49 | 1.48 | 1.48 | 1.48 | 1.49 |
| $N^4C^\alpha$ | 1.47 | 1.47 | 1.47 | 1.48 | 1.49 | 1.48 | 1.48 | 1.47 | 1.48 |
| $N^2C^\gamma$ | 1.43 | 1.44 | 1.44 | 1.44 | 1.43 | 1.43 | 1.43 | 1.43 | 1.43 |
| $C^\gamma C^\delta$ | 1.39 | 1.38 | 1.39 | 1.39 | 1.38 | 1.38 | 1.39 | 1.39 | 1.38 |
| $C^\gamma C^\varepsilon$ | 1.38 | 1.39 | 1.38 | 1.39 | 1.38 | 1.38 | 1.39 | 1.39 | 1.38 |
| C-H Bond Length | | | | | | | | | |
| $C^3H^1$ | 0.95 | 0.95 | 0.95 | 0.95 | 0.95 | 0.95 | 0.95 | 0.93 | 0.93 |
| $C^\alpha H^2$ | 0.99 | 0.99 | 0.99 | 0.99 | 0.99 | 0.99 | 0.99 | 0.97 | 0.95 |
| $C^\alpha H^3$ | 0.99 | 0.99 | 0.99 | 0.99 | 0.99 | 0.99 | 0.99 | 0.97 | 0.99 |
| H-H Distance | | | | | | | | | |
| $H^1H^2$ | 2.72 | 2.76 | 2.77 | 2.77 | 2.76 | 2.51 | 2.51 | 2.50 | 2.45 |
| $H^1H^3$ | 3.14 | 3.10 | 3.10 | 3.06 | 3.07 | 3.43 | 3.40 | 3.36 | 3.31 |
| Bond Angles | | | | | | | | | |
| $C^5N^1N^2$ | 103.5 | 103.5 | 103.4 | 103.2 | 103.2 | 104.3 | 104.2 | 104.3 | 103.5 |
| $N^1N^2C^3$ | 111.4 | 111.4 | 112.3 | 111.4 | 112.0 | 111.1 | 110.5 | 110.0 | 111.6 |
| $N^2C^3N^4$ | 106.6 | 107.1 | 106.6 | 107.2 | 106.5 | 107.4 | 107.8 | 107.4 | 106.9 |
| $C^3N^4C^5$ | 107.0 | 106.2 | 107.4 | 106.7 | 107.2 | 106.8 | 106.9 | 106.5 | 107.2 |
| $N^4C^5N^1$ | 111.5 | 111.7 | 110.3 | 111.5 | 111.1 | 110.4 | 110.7 | 110.8 | 110.8 |
| $C^\beta C^5N^4$ | 122.9 | 121.8 | 122.7 | 121.8 | 121.9 | 123.5 | 124.5 | 124.5 | 124.3 |
| $C^5N^4C^\alpha$ | 126.0 | 125.8 | 124.3 | 126.1 | 125.7 | 127.6 | 127.2 | 127.9 | 127.7 |
| Torsion Angles | | | | | | | | | |
| $H^1C^3{*}C^\alpha H^2$ | 35.1 | 37.4 | 38.4 | 41.3 | 39.0 | 0.6 | 1.3 | 2.6 | 2.4 |
| $H^1C^3{*}C^\alpha H^3$ | 74.1 | 71.7 | 70.4 | 68.1 | 70.1 | 107.5 | 105.6 | 103.9 | 104.3 |
| $N^1N^2{*}C^\gamma C^\delta$ | 21.7 | 32.1 | 21.0 | 72.9 | 65.0 | 44.3 | 21.0 | 17.0 | 73.8 |
| $N^1N^2{*}C^\gamma C^\varepsilon$ | 157.3 | 147.3 | 158.4 | 105.7 | 119.3 | 135.3 | 159.5 | 163.3 | 105.9 |

Table S19. Summary of average bond angles and distances of triazolium salts **7a-k** (n=1); **8a-c, 8i**, **8k** (n=2); **9a-c**, **9k** (n=3) and corresponding standard deviations obtained from single crystal X-ray structural analysis. Data highlighted in purple is included in Table 2 in the main text.

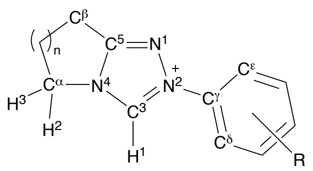

| Backbone Bond Length | Average (n=1) | SD (n=1) | Average (n=2) | SD (n=2) | Average (n=3) | SD (n=3) | Differences of averaged values | n=1 vs n=2 | n=2 vs n=3 |
|---|---|---|---|---|---|---|---|---|---|
| $N^1N^2$ | 1.38 | 0.00 | 1.38 | 0.00 | 1.38 | 0.01 | | -0.01 | 0.00 |
| $N^2C^3$ | 1.32 | 0.01 | 1.31 | 0.01 | 1.32 | 0.01 | | -0.01 | 0.00 |
| $C^3N^4$ | 1.33 | 0.00 | 1.33 | 0.01 | 1.33 | 0.00 | | 0.01 | 0.00 |
| $N^4C^5$ | 1.36 | 0.01 | 1.36 | 0.00 | 1.37 | 0.00 | | 0.00 | 0.01 |
| $C^5N^1$ | 1.30 | 0.00 | 1.31 | 0.01 | 1.31 | 0.00 | | 0.01 | 0.00 |
| $C^5C^\beta$ | 1.49 | 0.01 | 1.49 | 0.01 | 1.48 | 0.01 | | 0.00 | 0.00 |
| $N^4C^\alpha$ | 1.47 | 0.01 | 1.47 | 0.01 | 1.48 | 0.00 | | 0.00 | 0.00 |
| $N^2C^\gamma$ | 1.43 | 0.01 | 1.43 | 0.01 | 1.43 | 0.00 | | 0.00 | 0.00 |
| $C^\gamma C^\delta$ | 1.39 | 0.01 | 1.39 | 0.01 | 1.38 | 0.01 | | 0.00 | -0.01 |
| $C^\gamma C^\varepsilon$ | 1.39 | 0.01 | 1.38 | 0.01 | 1.38 | 0.00 | | 0.00 | 0.00 |
| C-H Bond Length | | | | | | | | | |
| $C^3H^1$ | 0.94 | 0.02 | 0.95 | 0.00 | 0.94 | 0.01 | | 0.01 | -0.01 |
| $C^\alpha H^2$ | 0.97 | 0.03 | 0.99 | 0.00 | 0.97 | 0.02 | | 0.02 | -0.02 |
| $C^\alpha H^3$ | 0.98 | 0.02 | 0.99 | 0.00 | 0.99 | 0.01 | | 0.01 | 0.00 |
| H-H Distance | | | | | | | | | |
| $H^1H^2$ | 3.06 | 0.04 | 2.76 | 0.02 | 2.49 | 0.03 | | -0.31 | -0.26 |
| $H^1H^3$ | 3.29 | 0.05 | 3.09 | 0.03 | 3.37 | 0.05 | | -0.19 | 0.28 |
| Bond Angles | | | | | | | | | |
| $C^5N^1N^2$ | 102.8 | 0.3 | 103.3 | 0.1 | 104.1 | 0.4 | | 0.6 | 0.7 |
| $N^1N^2C^3$ | 111.9 | 0.4 | 111.7 | 0.4 | 110.8 | 0.7 | | -0.2 | -0.9 |
| $N^2C^3N^4$ | 106.1 | 0.3 | 106.8 | 0.3 | 107.4 | 0.3 | | 0.8 | 0.6 |
| $C^3N^4C^5$ | 107.4 | 0.2 | 106.9 | 0.5 | 106.9 | 0.3 | | -0.5 | 0.0 |
| $N^4C^5N^1$ | 111.9 | 0.2 | 111.3 | 0.6 | 110.7 | 0.2 | | -0.6 | -0.6 |
| $C^\beta C^5N^4$ | 110.6 | 0.5 | 122.2 | 0.5 | 124.2 | 0.5 | | 11.6 | 2.0 |
| $C^5N^4C^\alpha$ | 113.7 | 0.4 | 125.6 | 0.7 | 127.6 | 0.3 | | 11.8 | 2.0 |
| Torsion Angles | | | | | | | | | |
| $H^1C^3{*}C^\alpha H^2$ | 43.6 | 1.9 | 38.2 | 2.3 | 1.7 | 0.9 | | -5.4 | -36.5 |
| $H^1C^3{*}C^\alpha H^3$ | 69.6 | 1.6 | 70.9 | 2.2 | 105.3 | 1.6 | | 1.3 | 34.4 |
| $N^1N^2{*}C^\gamma C^\delta$ | 41.2 | 34.8 | 42.5 | 24.7 | 39.0 | 26.1 | | 1.38 | -3.54 |
| $N^1N^2{*}C^\gamma C^\varepsilon$ | 139.1 | 35.1 | 137.6 | 23.8 | 141.0 | 26.5 | | -1.46 | 3.39 |

Table S20. Summary of average* bond angles and distances of triazolium salts **7a-c, k** (n=1); **8a-c, 8k** (n=2); **9a-c**, **9k** (n=3) and corresponding standard deviations (SD) obtained from single crystal X-ray structural analysis.

*Averages calculated using only data for the 12 triazolium salts used for C(3)-H/D exchange studies. These average values and trends are in excellent agreement with data in Table S19 as expected given the small standard deviations.

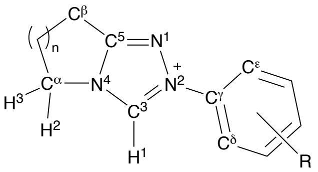


| Backbone Bond Length | Average (n=1) | SD (n=1) | Average (n=2) | SD (n=2) | Average (n=3) | SD (n=3) |
|---|---|---|---|---|---|---|
| $N^1N^2$ | 1.38 | 0.00 | 1.38 | 0.00 | 1.38 | 0.01 |
| $N^2C^3$ | 1.32 | 0.01 | 1.31 | 0.01 | 1.32 | 0.01 |
| $C^3N^4$ | 1.33 | 0.01 | 1.33 | 0.00 | 1.33 | 0.00 |
| $N^4C^5$ | 1.36 | 0.00 | 1.36 | 0.01 | 1.37 | 0.00 |
| $C^5N^1$ | 1.30 | 0.00 | 1.31 | 0.01 | 1.31 | 0.00 |
| $C^5C^\beta$ | 1.49 | 0.01 | 1.49 | 0.01 | 1.48 | 0.01 |
| $N^4C^\alpha$ | 1.47 | 0.01 | 1.47 | 0.01 | 1.48 | 0.00 |
| $N^2C^\gamma$ | 1.43 | 0.00 | 1.43 | 0.01 | 1.43 | 0.00 |
| $C^\gamma C^\delta$ | 1.39 | 0.01 | 1.39 | 0.01 | 1.38 | 0.01 |
| $C^\gamma C^\varepsilon$ | 1.38 | 0.01 | 1.38 | 0.00 | 1.38 | 0.00 |
| C-H Bond Length | | | | | | |
| $C^3H^1$ | 0.95 | 0.00 | 0.95 | 0.00 | 0.94 | 0.01 |
| $C^\alpha H^2$ | 0.98 | 0.01 | 0.99 | 0.00 | 0.97 | 0.02 |
| $C^\alpha H^3$ | 0.99 | 0.00 | 0.99 | 0.00 | 0.99 | 0.01 |
| H-H Distance | | | | | | |
| $H^1H^2$ | 3.07 | 0.02 | 2.75 | 0.02 | 2.49 | 0.03 |
| $H^1H^3$ | 3.29 | 0.04 | 3.10 | 0.03 | 3.37 | 0.05 |
| Bond Angles | | | | | | |
| $C^5N^1N^2$ | 102.8 | 0.4 | 103.4 | 0.1 | 104.1 | 0.4 |
| $N^1N^2C^3$ | 112.1 | 0.4 | 111.8 | 0.4 | 110.8 | 0.7 |
| $N^2C^3N^4$ | 105.9 | 0.2 | 106.7 | 0.3 | 107.4 | 0.3 |
| $C^3N^4C^5$ | 107.5 | 0.2 | 106.9 | 0.5 | 106.9 | 0.3 |
| $N^4C^5N^1$ | 111.8 | 0.3 | 111.2 | 0.6 | 110.7 | 0.2 |
| $C^\beta C^5N^4$ | 110.4 | 0.6 | 122.3 | 0.5 | 124.2 | 0.5 |
| $C^5N^4C^\alpha$ | 114.0 | 0.5 | 125.4 | 0.8 | 127.6 | 0.3 |

| Torsion Angles | | | | | | |
|---|---|---|---|---|---|---|
| $H^1C^3*C^{\alpha}H^2$ | 43.8 | 2.0 | 37.5 | 1.7 | 1.7 | 0.9 |
| $H^1C^3*C^{\alpha}H^3$ | 69.3 | 1.3 | 71.6 | 1.8 | 105.3 | 1.6 |
| $N^1N^2*C^{\gamma}C^{\delta}$ | 30.8 | 21.2 | 35.0 | 20.7 | 39.0 | 26.1 |
| $N^1N^2*C^{\gamma}C^{\varepsilon}$ | 150.6 | 18.8 | 145.6 | 18.2 | 141.0 | 26.5 |

## S1.7 Synthetic $^1$H and $^{13}$C{1H} NMR Spectra for New Triazolium Salts

**7e**

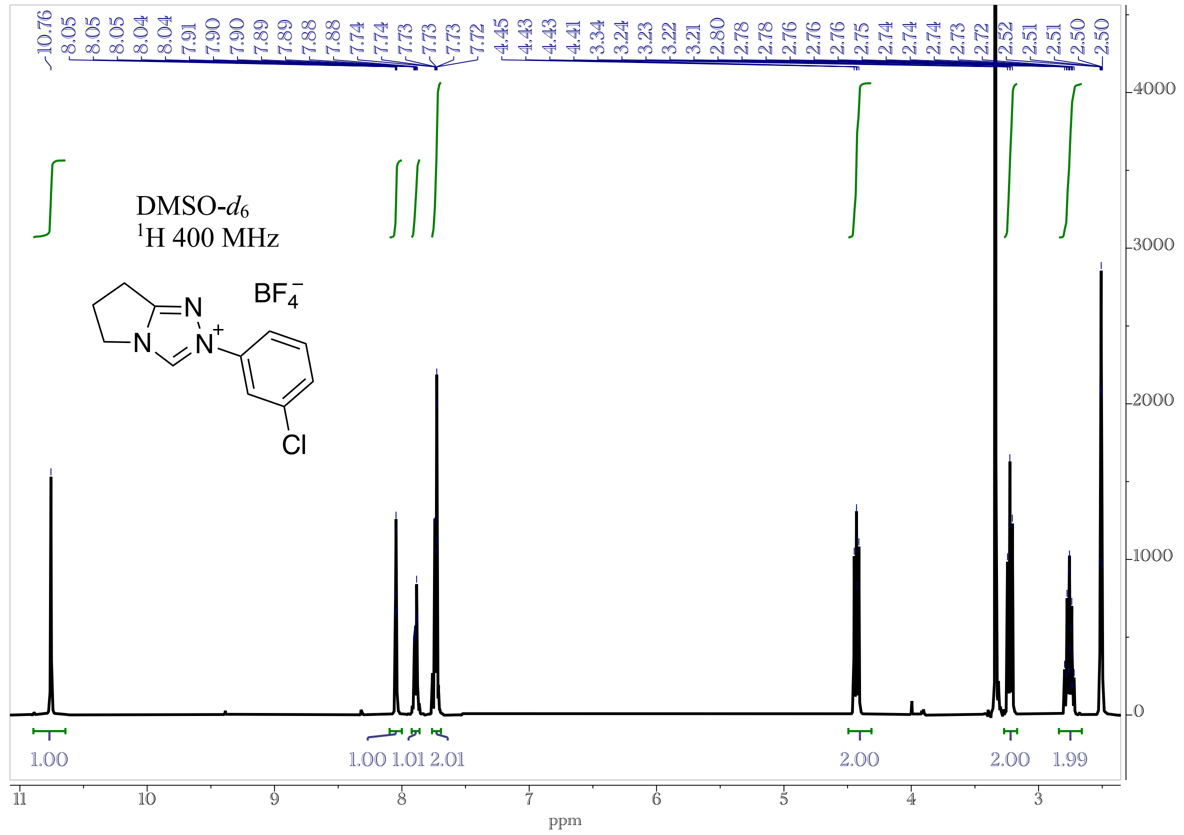


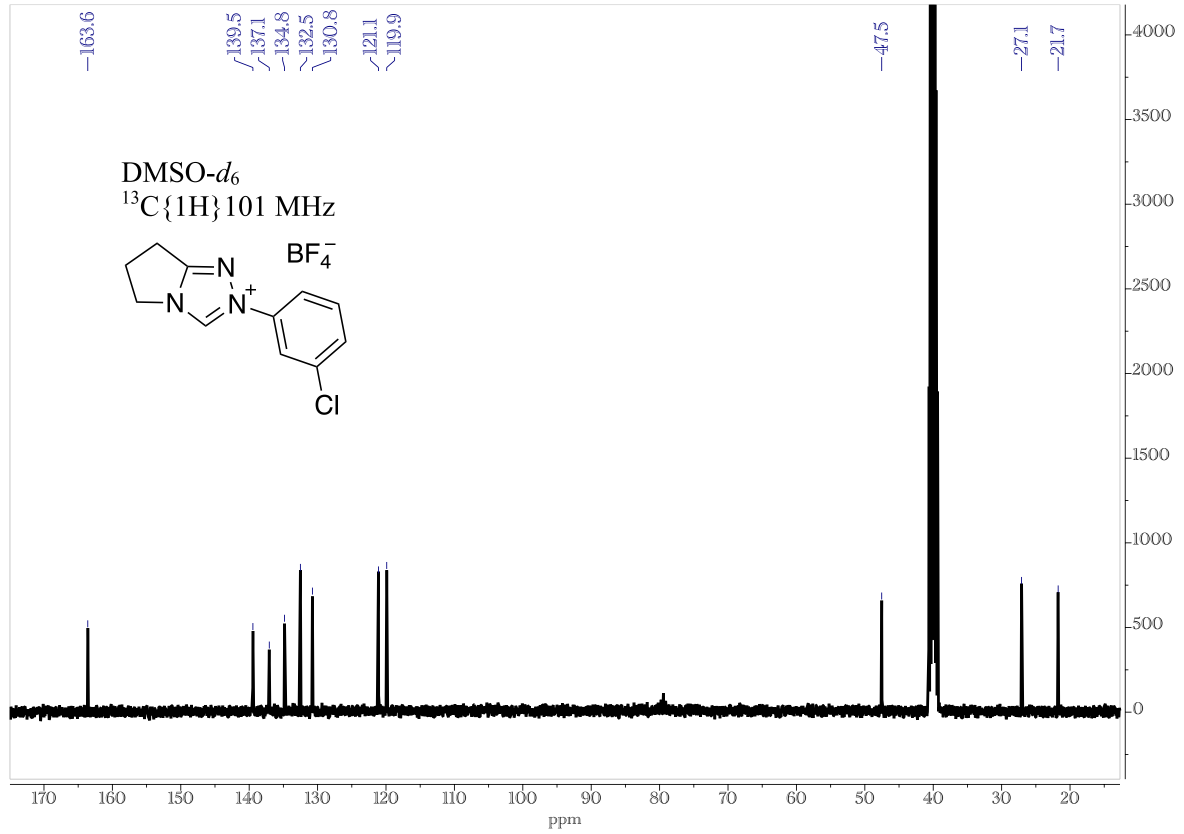

**7f**

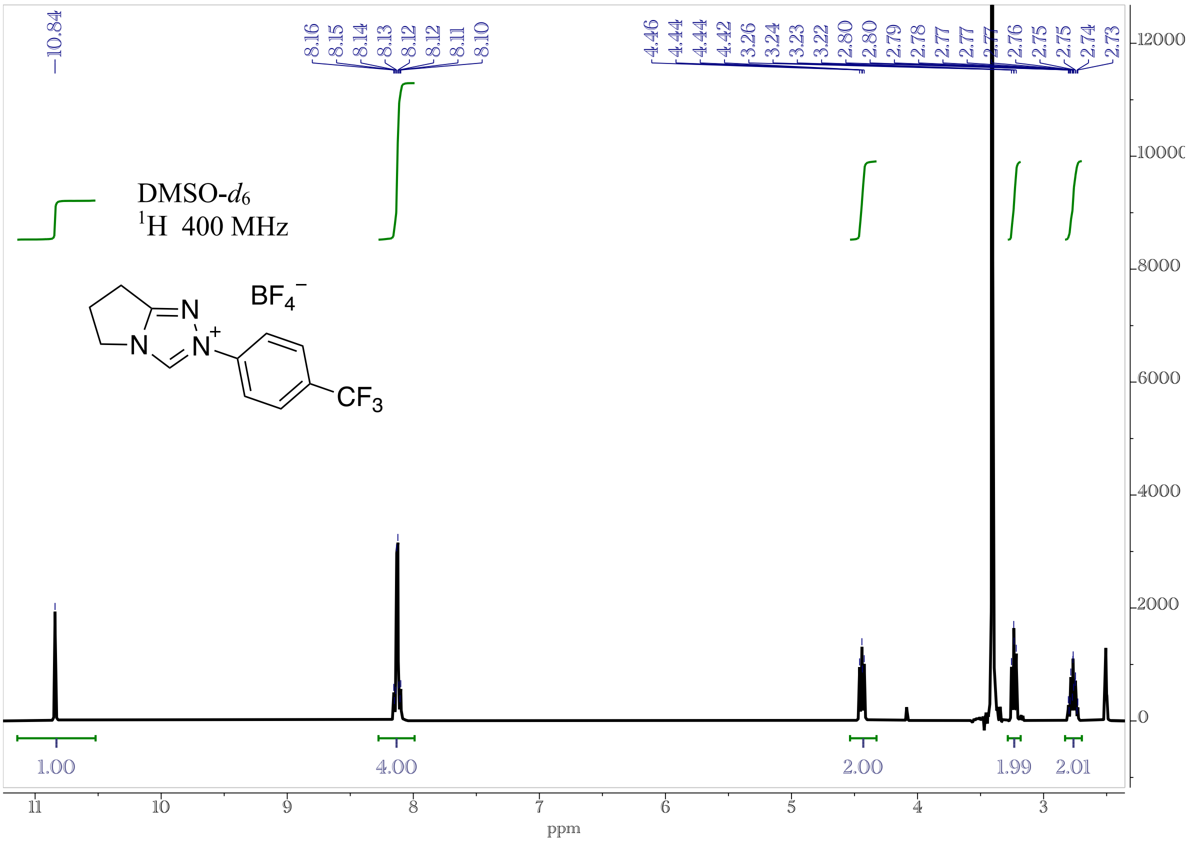
DMSO-$d_6$
$^1$H 400 MHz
BF4−
CF3
N
N+
N
1.00
4.00
2.00
1.99
2.01
ppm

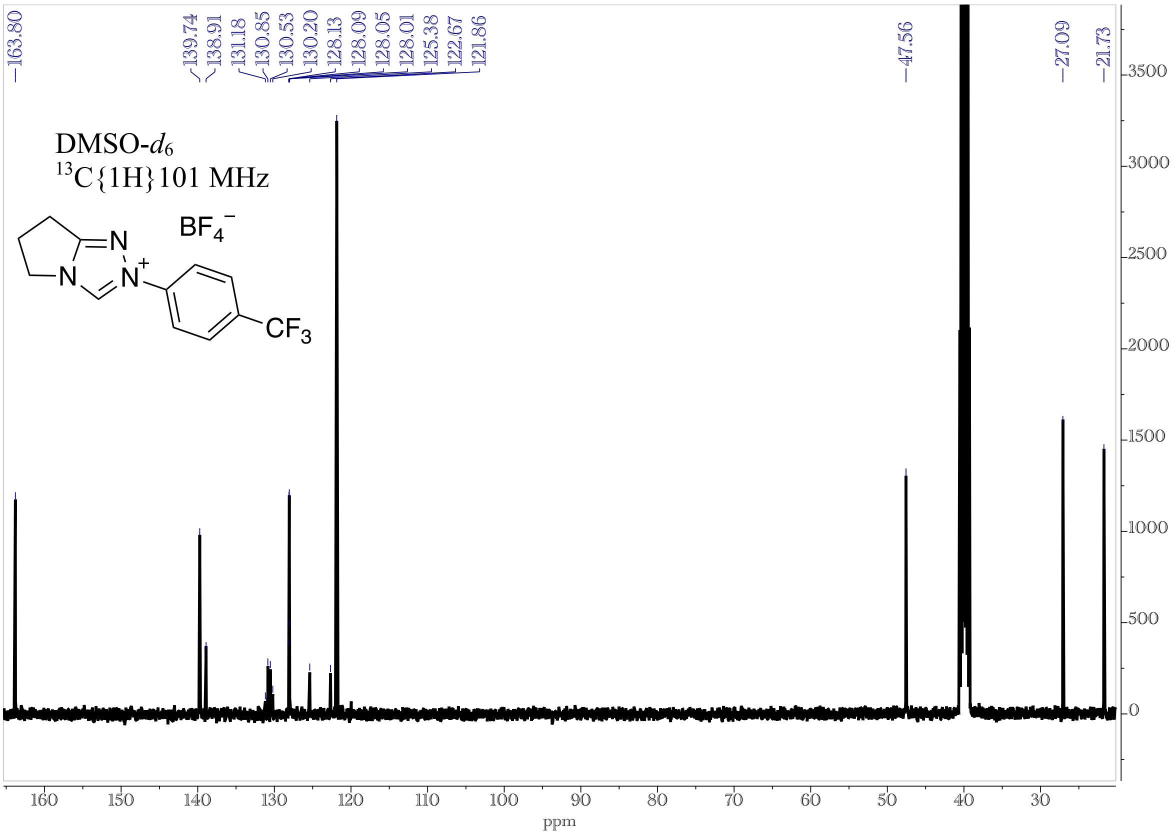
DMSO-$d_6$
$^{13}$C{1H}101 MHz
BF4−
CF3
163.80
139.74
138.91
131.18
130.85
130.53
130.20
128.13
128.09
128.05
128.01
125.38
122.67
121.86
47.56
27.09
21.73
ppm

**7h**

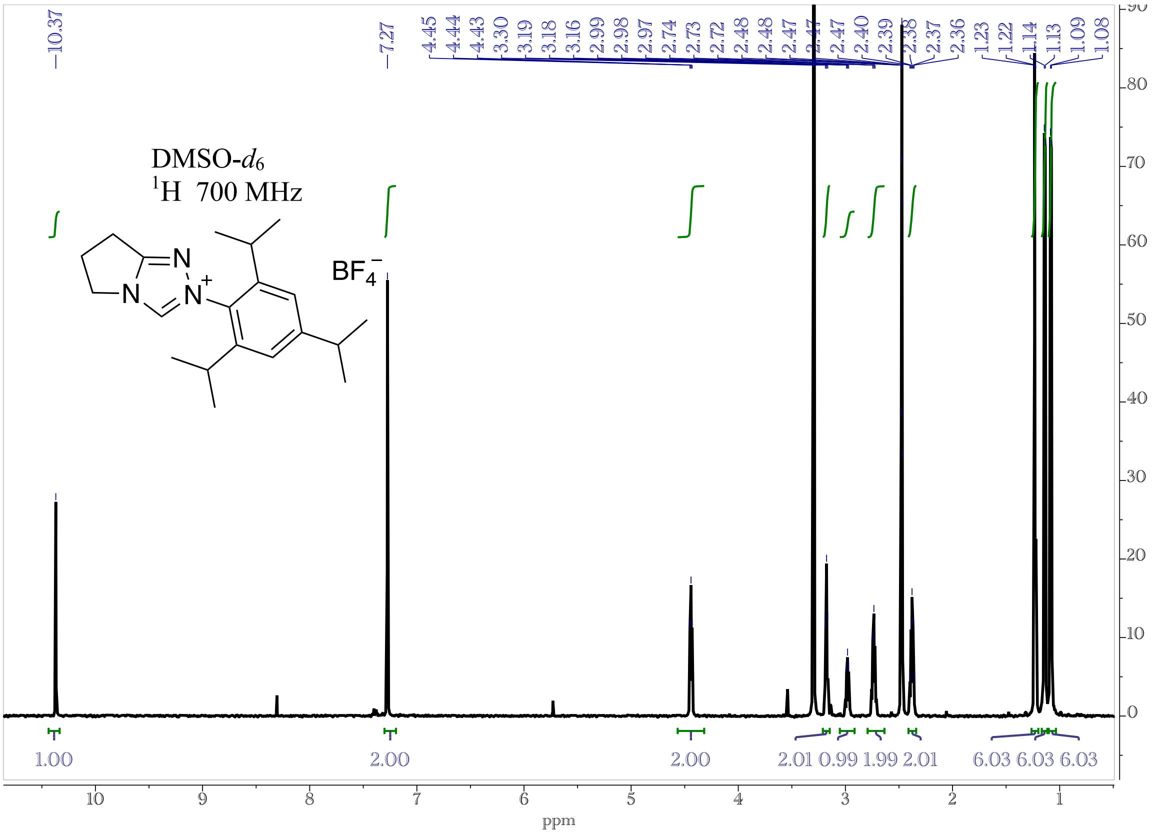
DMSO-$d_6$
$^1$H 700 MHz
BF$_4^-$
10.37
7.27
4.45
4.44
4.43
3.30
3.19
3.18
3.16
2.99
2.98
2.97
2.74
2.73
2.72
2.48
2.48
2.47
2.47
2.47
2.40
2.39
2.38
2.37
2.36
1.23
1.22
1.14
1.13
1.09
1.08
1.00
2.00
2.00
2.01
0.99
1.99
2.01
6.03
6.03
6.03
ppm

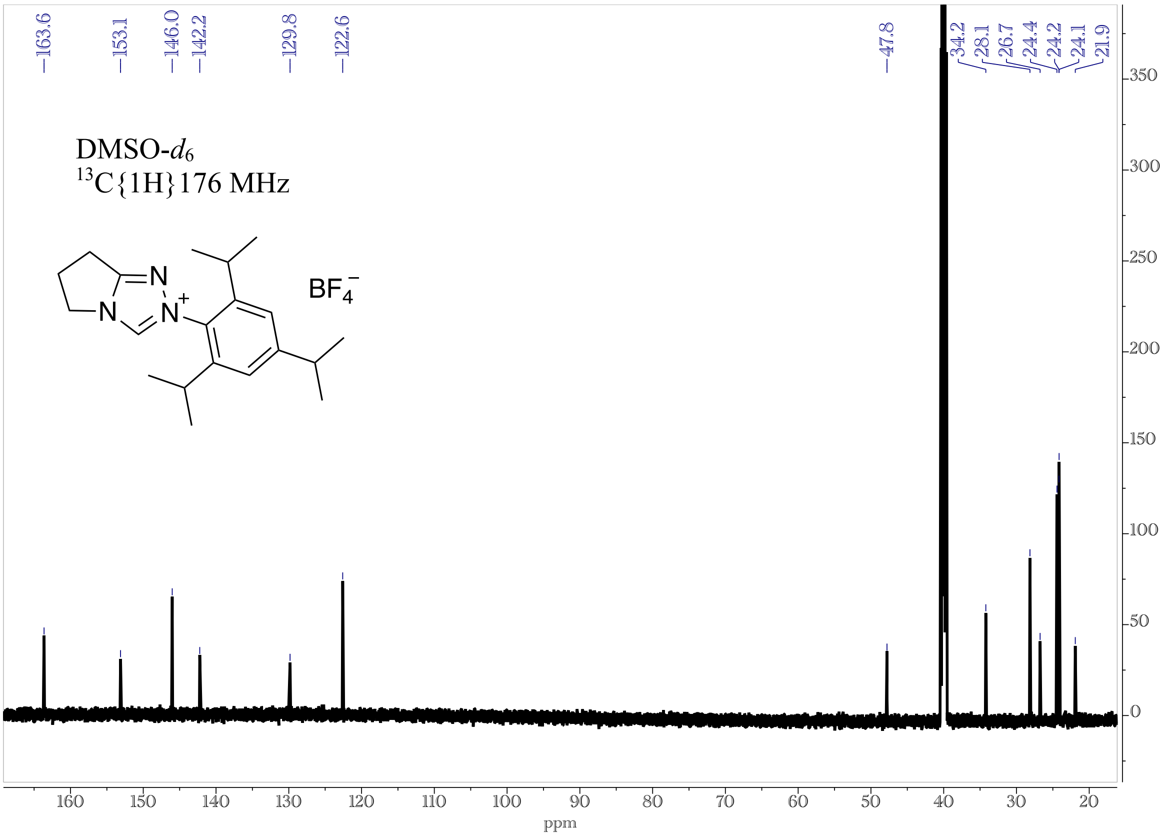
DMSO-$d_6$
$^{13}$C{1H} 176 MHz
BF$_4^-$
163.6
153.1
146.0
142.2
129.8
122.6
47.8
34.2
28.1
26.7
24.4
24.2
24.1
21.9
ppm

**8a**

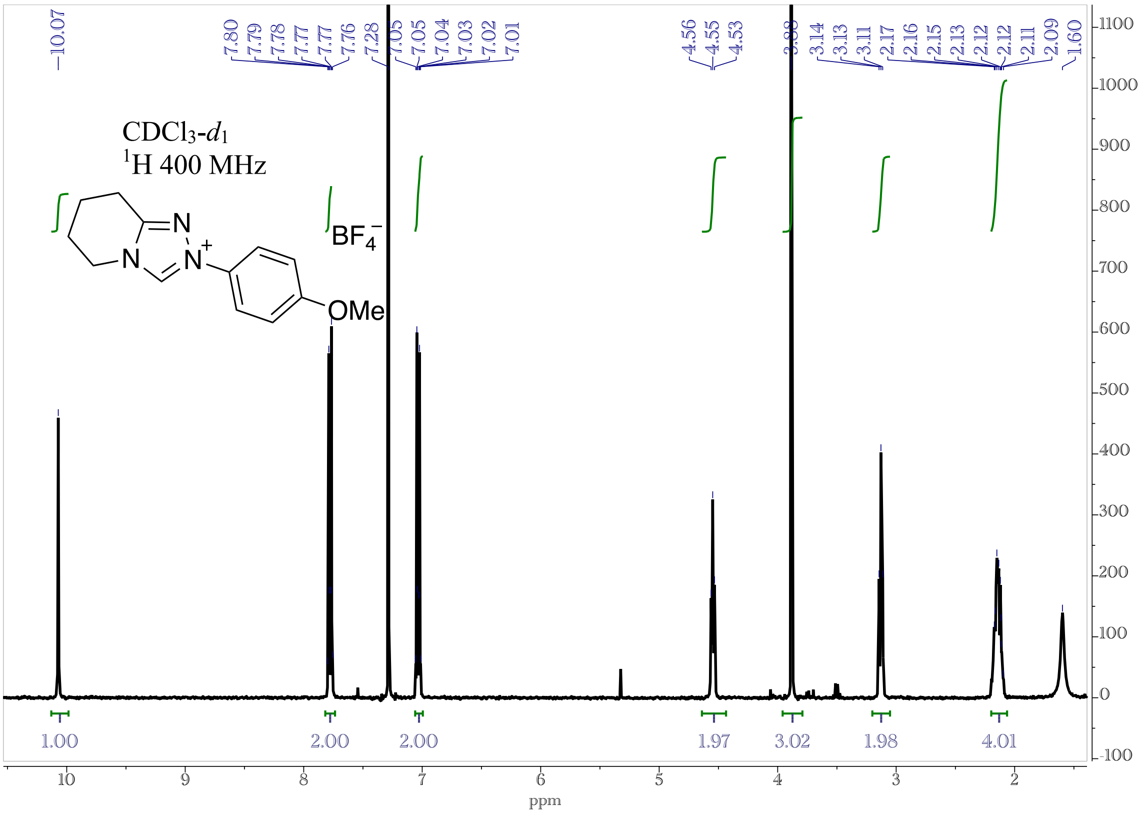

CDCl3-d1
1H 400 MHz
BF4
OMe
10.07
7.80 7.79 7.78 7.77 7.77 7.76 7.28 7.05 7.05 7.04 7.03 7.02 7.01
4.56 4.55 4.53
3.14 3.13 3.11 2.17 2.16 2.15 2.13 2.12 2.12 2.11 2.09 1.60
1.00 2.00 2.00 1.97 3.02 1.98 4.01
ppm


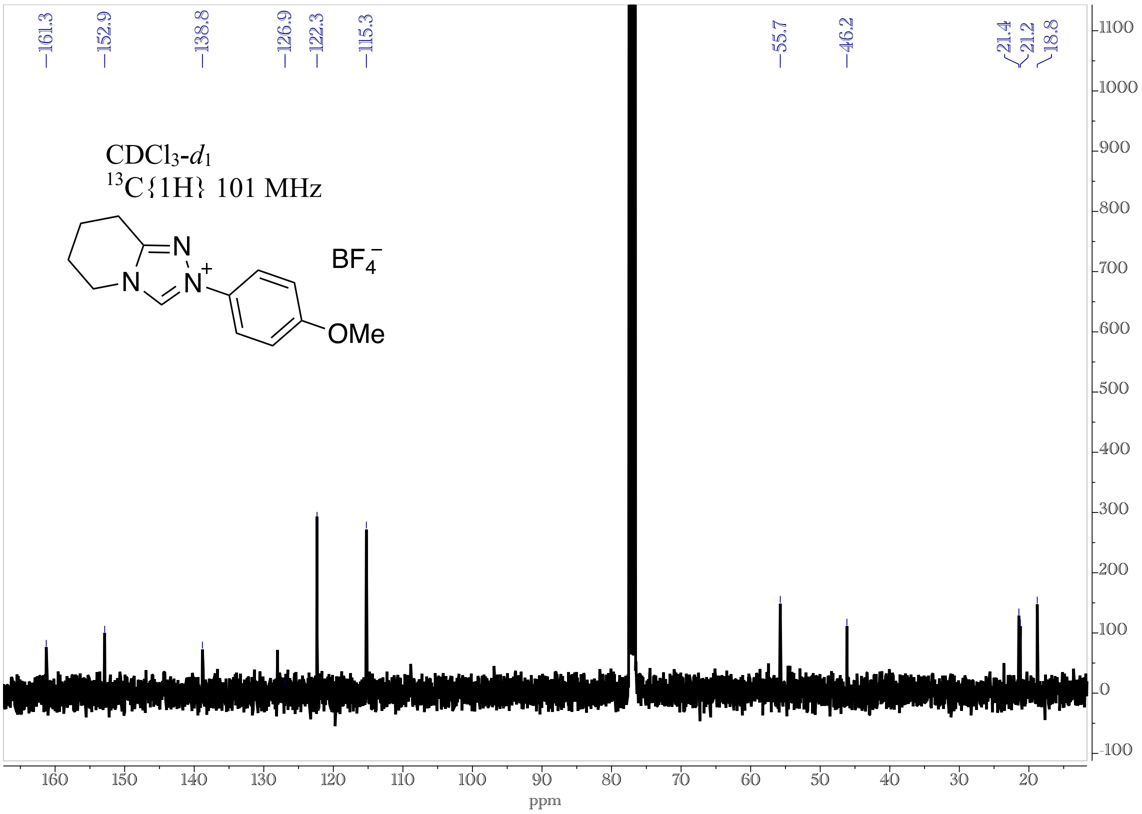

CDCl3-d1
13C{1H} 101 MHz
BF4
OMe
161.3 152.9 138.8 126.9 122.3 115.3 55.7 46.2 21.4 21.2 18.8
ppm

**8c**

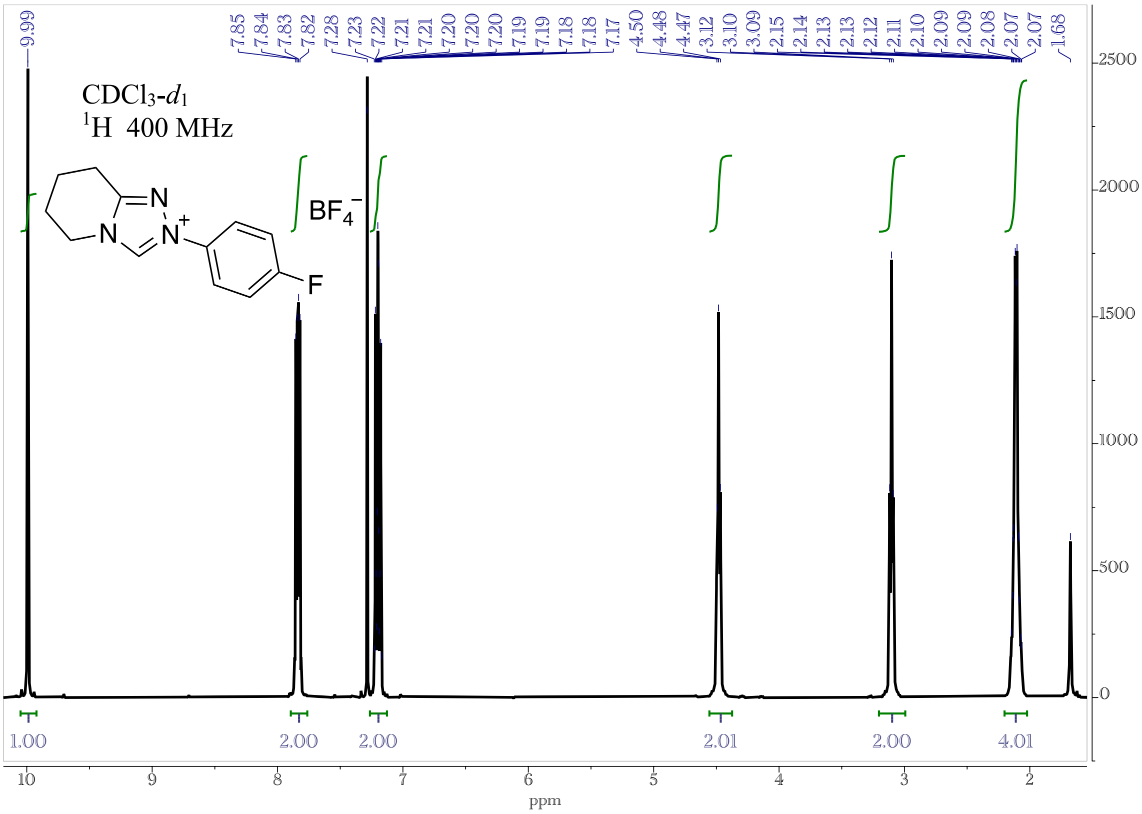
CDCl3-d1
1H 400 MHz
BF4−
F
1.00
2.00
2.00
2.01
2.00
4.01
ppm

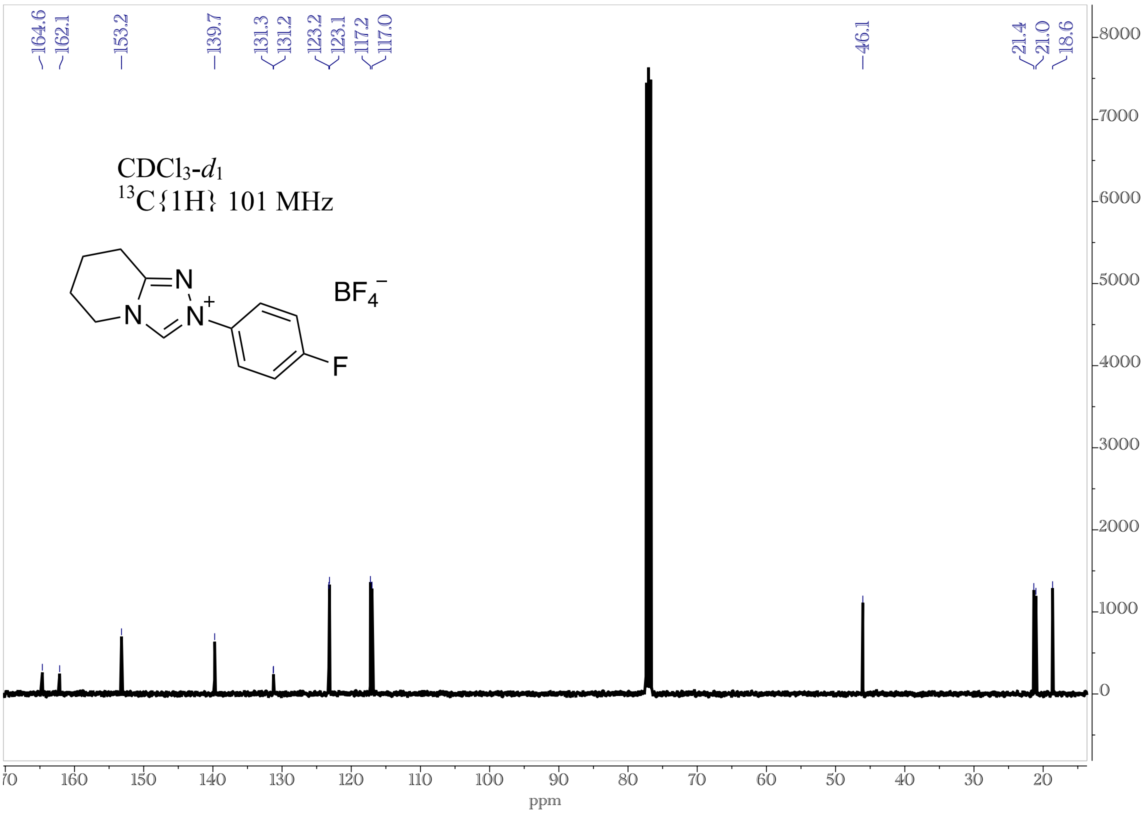
CDCl3-d1
13C{1H} 101 MHz
BF4−
F
ppm

**9a**

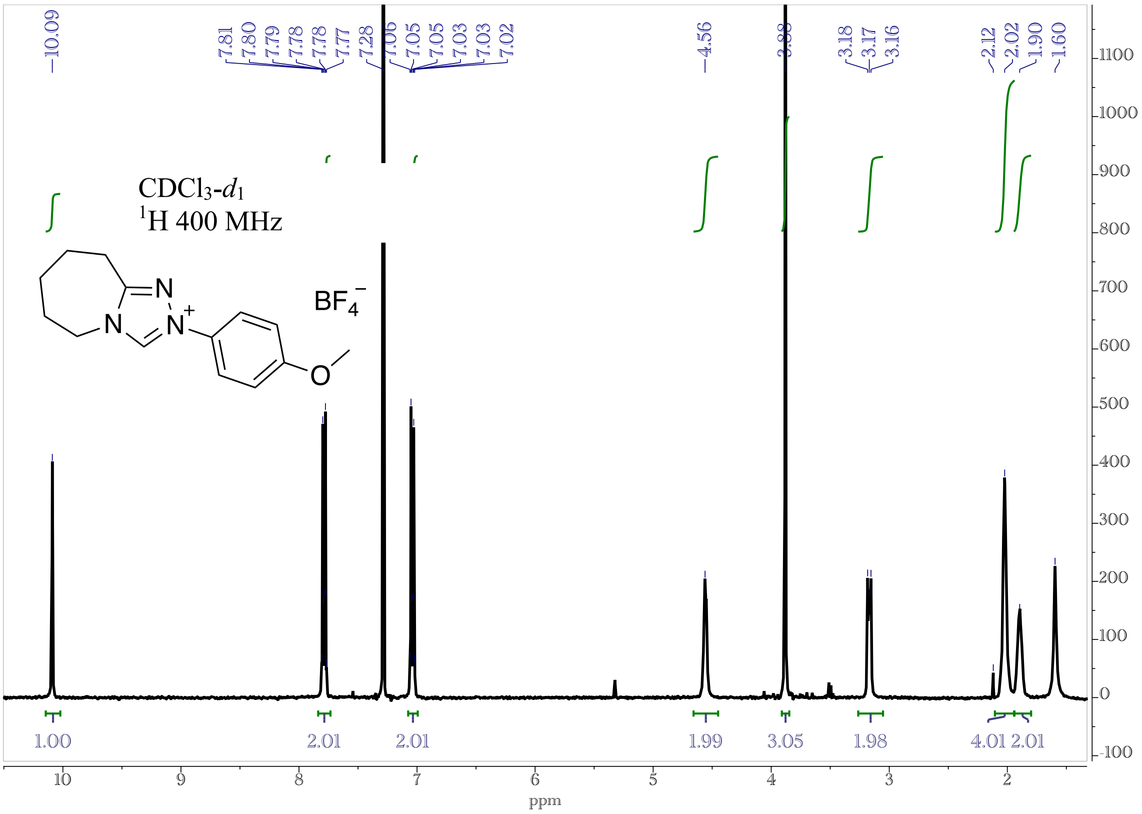


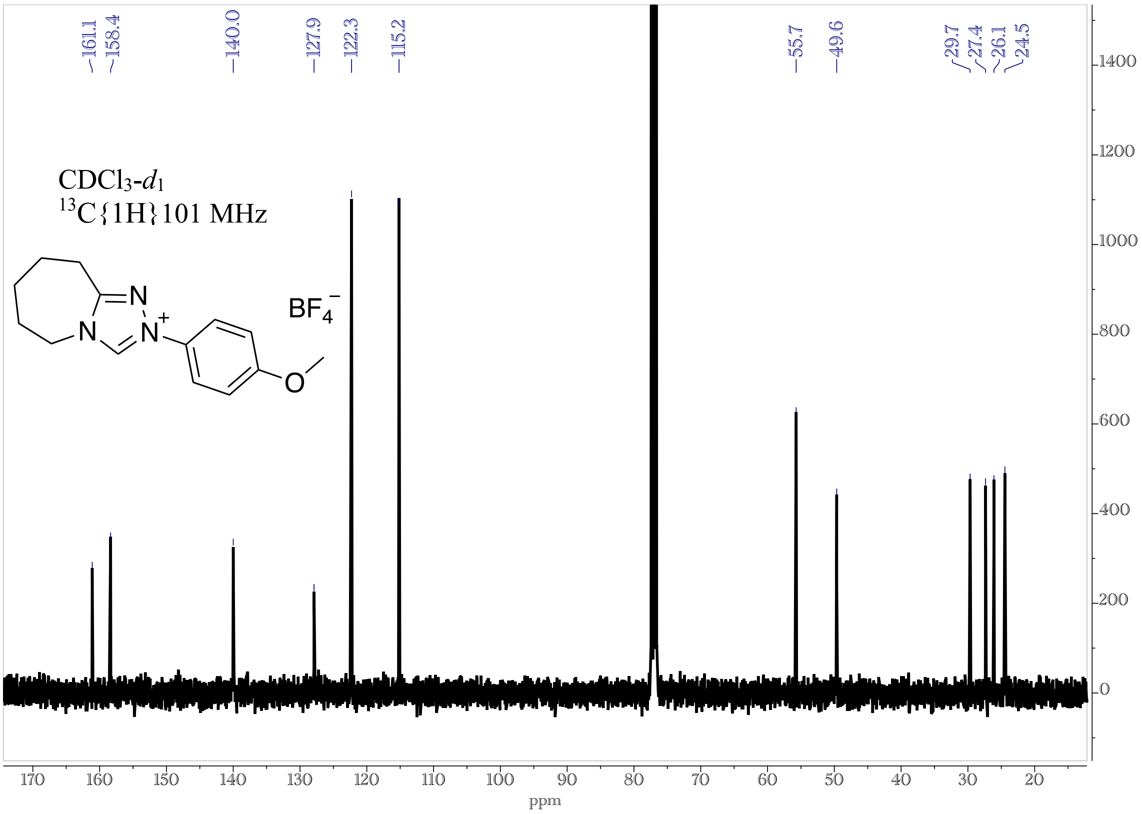

**9c**

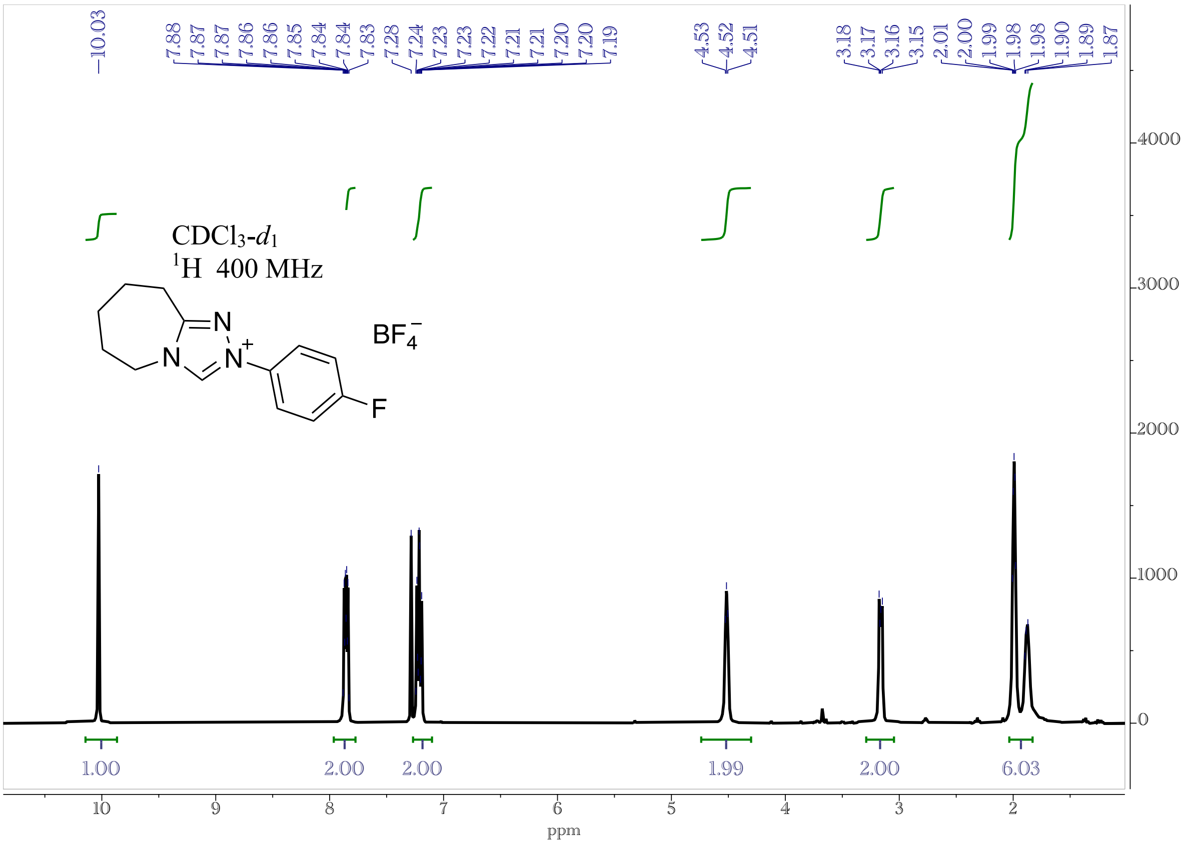

10.03
7.88
7.87
7.87
7.86
7.86
7.85
7.84
7.84
7.83
7.28
7.24
7.23
7.23
7.22
7.21
7.21
7.20
7.20
7.19
4.53
4.52
4.51
3.18
3.17
3.16
3.15
2.01
2.00
1.99
1.98
1.98
1.90
1.89
1.87
CDCl3-d1
1H 400 MHz
N
N
N+
BF4−
F
1.00
2.00
2.00
1.99
2.00
6.03
ppm


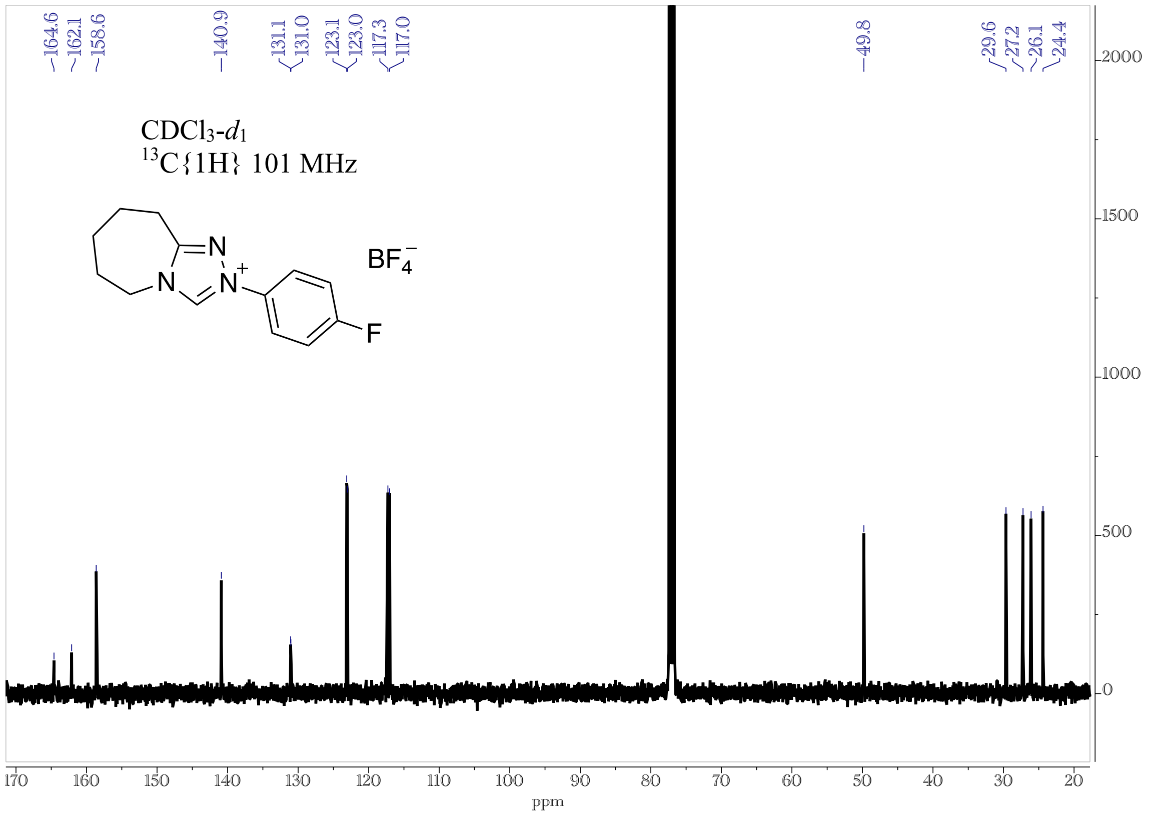

164.6
162.1
158.6
140.9
131.1
131.0
123.1
123.0
117.3
117.0
49.8
29.6
27.2
26.1
24.4
CDCl3-d1
13C{1H} 101 MHz
N
N
N+
BF4−
F
ppm

## S1.8 Synthetic ¹H NMR Spectra for Known Triazolium Salts

### 7a

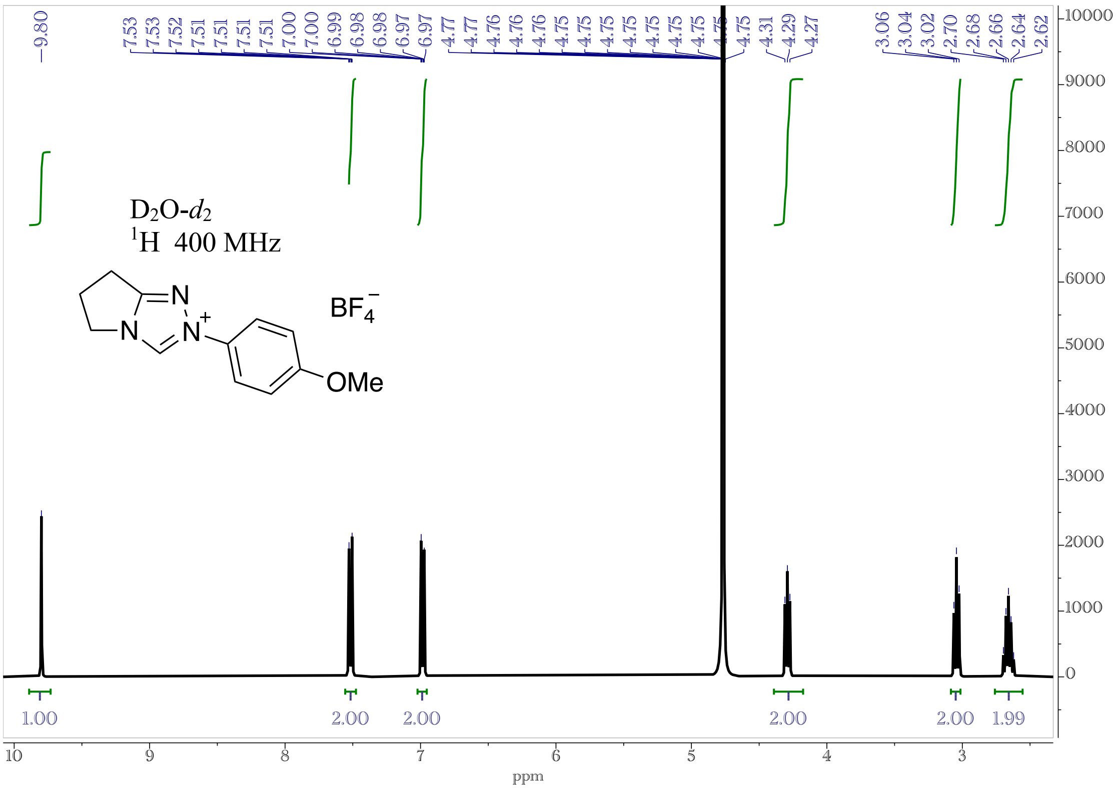


### 7b

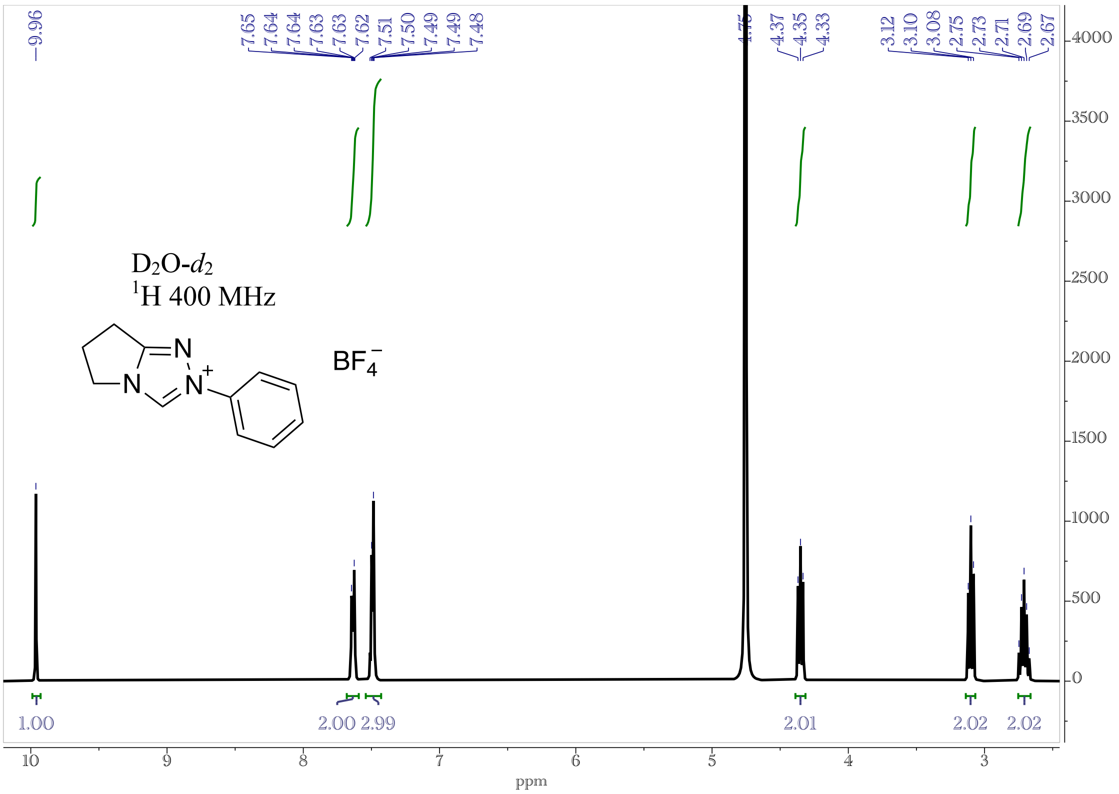

**7c**

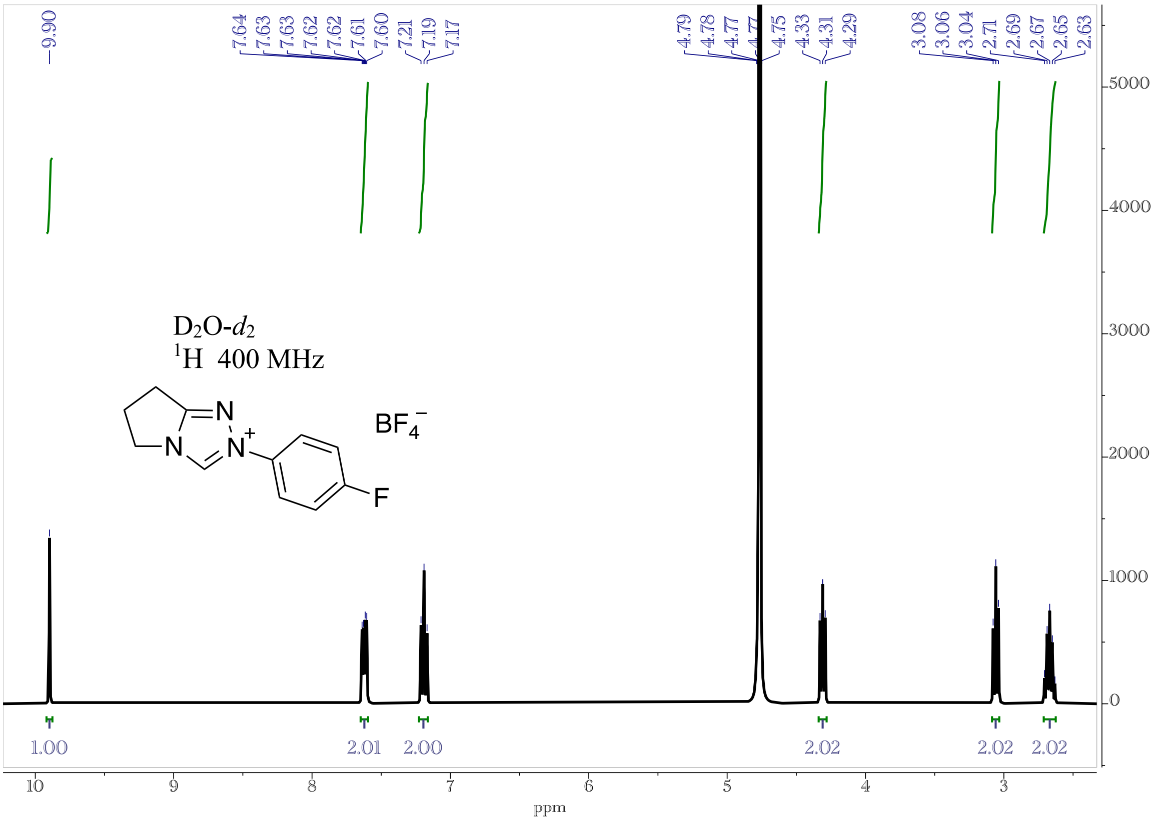

9.90
7.64
7.63
7.63
7.62
7.62
7.61
7.60
7.21
7.19
7.17
4.79
4.78
4.77
4.77
4.75
4.33
4.31
4.29
3.08
3.06
3.04
2.71
2.69
2.67
2.65
2.63
D2O-d2
1H 400 MHz
BF4
N
N
N
F
1.00
2.01
2.00
2.02
2.02
2.02
ppm


**7d**

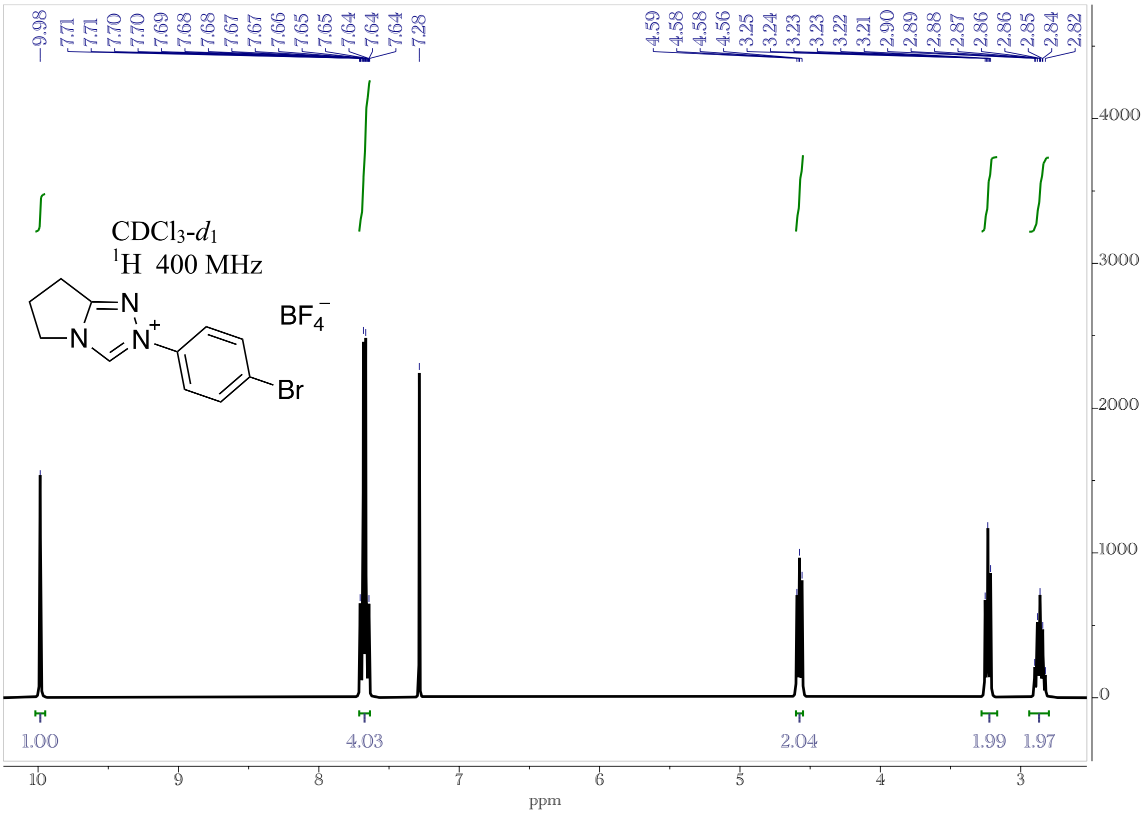

9.98
7.71
7.71
7.70
7.70
7.69
7.68
7.68
7.67
7.67
7.66
7.65
7.65
7.64
7.64
7.64
7.28
4.59
4.58
4.58
4.56
3.25
3.24
3.23
3.23
3.22
3.21
2.90
2.89
2.88
2.87
2.86
2.86
2.85
2.84
2.82
CDCl3-d1
1H 400 MHz
BF4
N
N
N
Br
1.00
4.03
2.04
1.99
1.97
ppm

**7g**

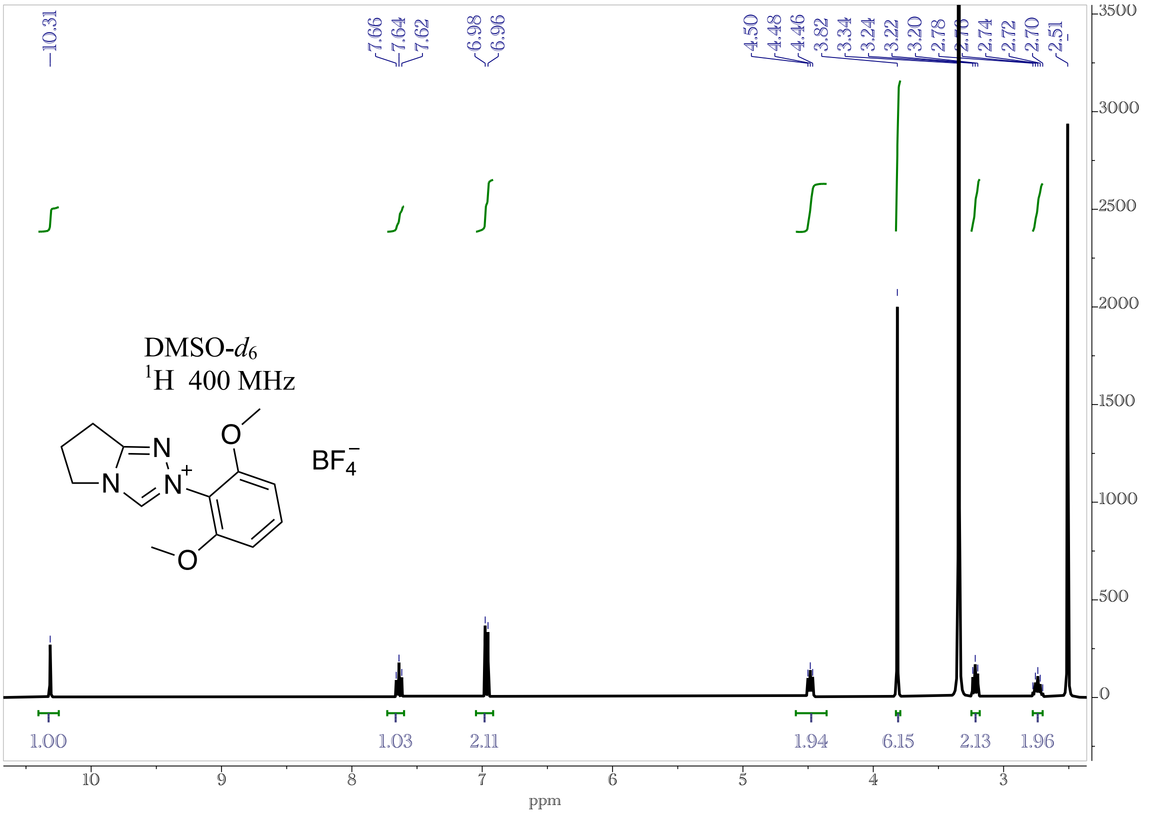


**7i**

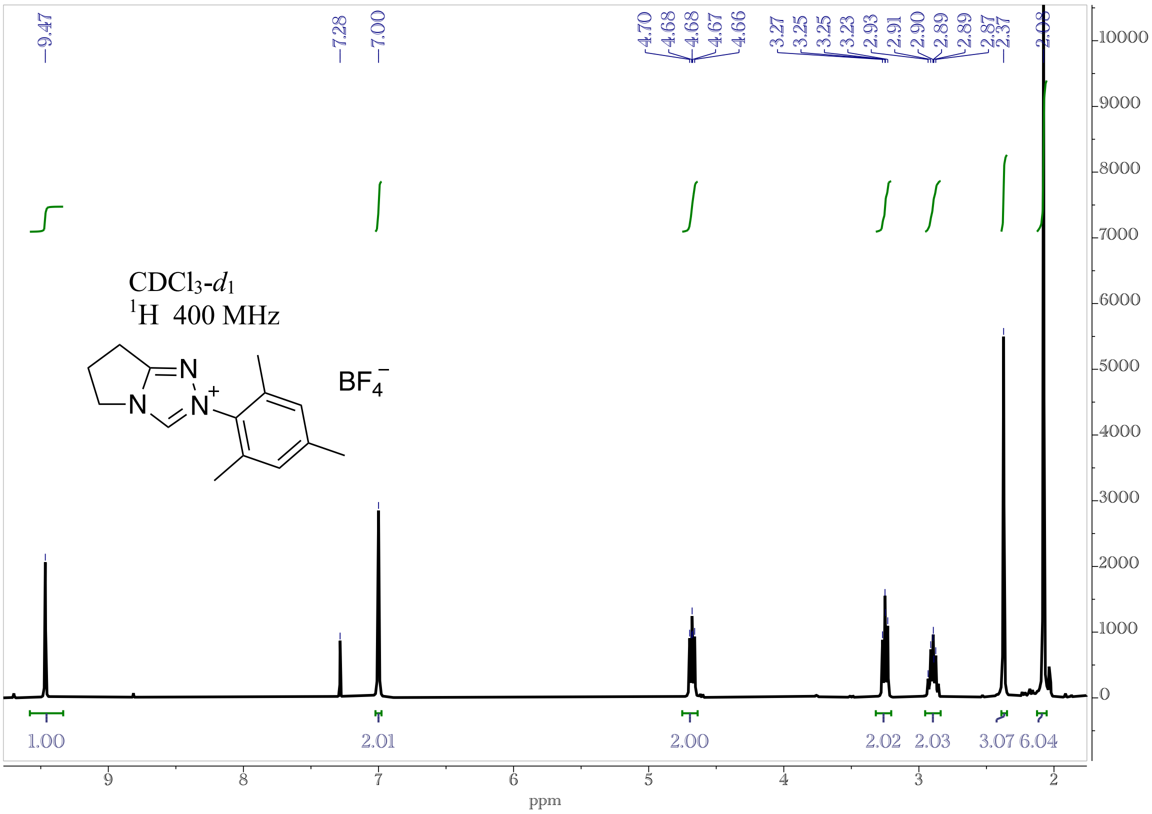

**7j**

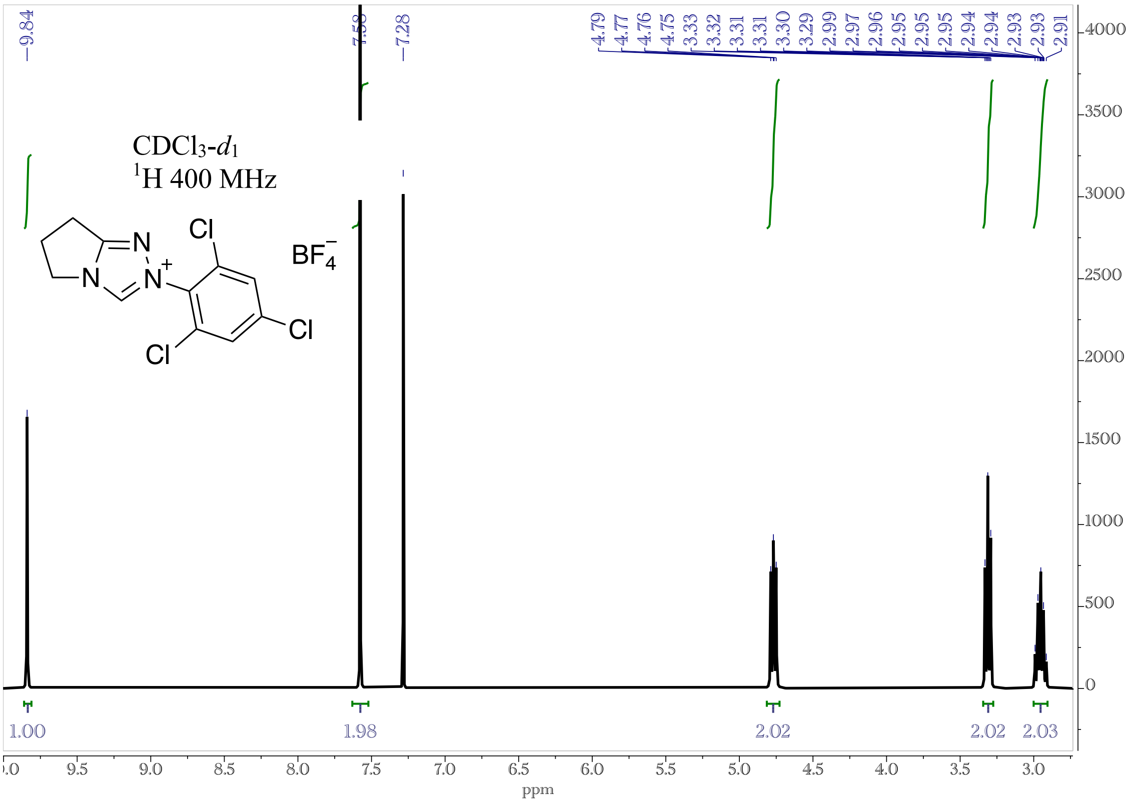

CDCl3-d1
1H 400 MHz
BF4−
Cl
Cl
Cl
N
N+
N
ppm


**7k**

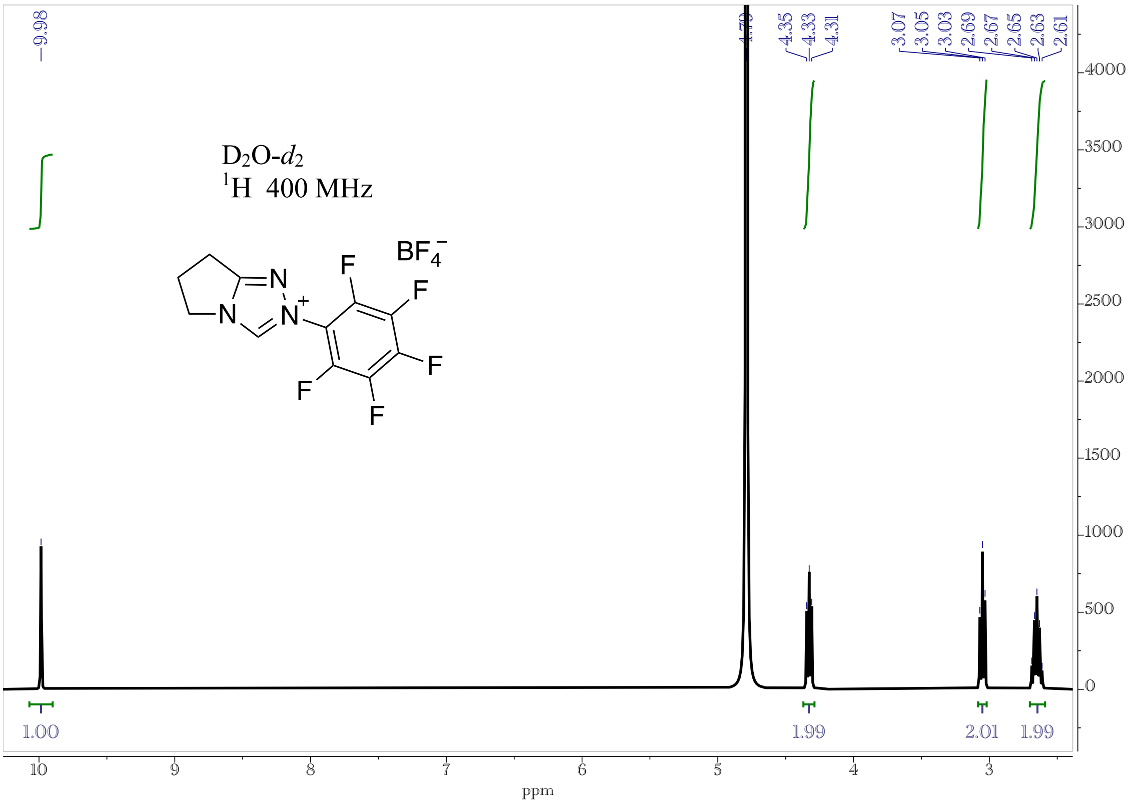

D2O-d2
1H 400 MHz
BF4−
F
F
F
F
F
N
N+
N
ppm

**8b**

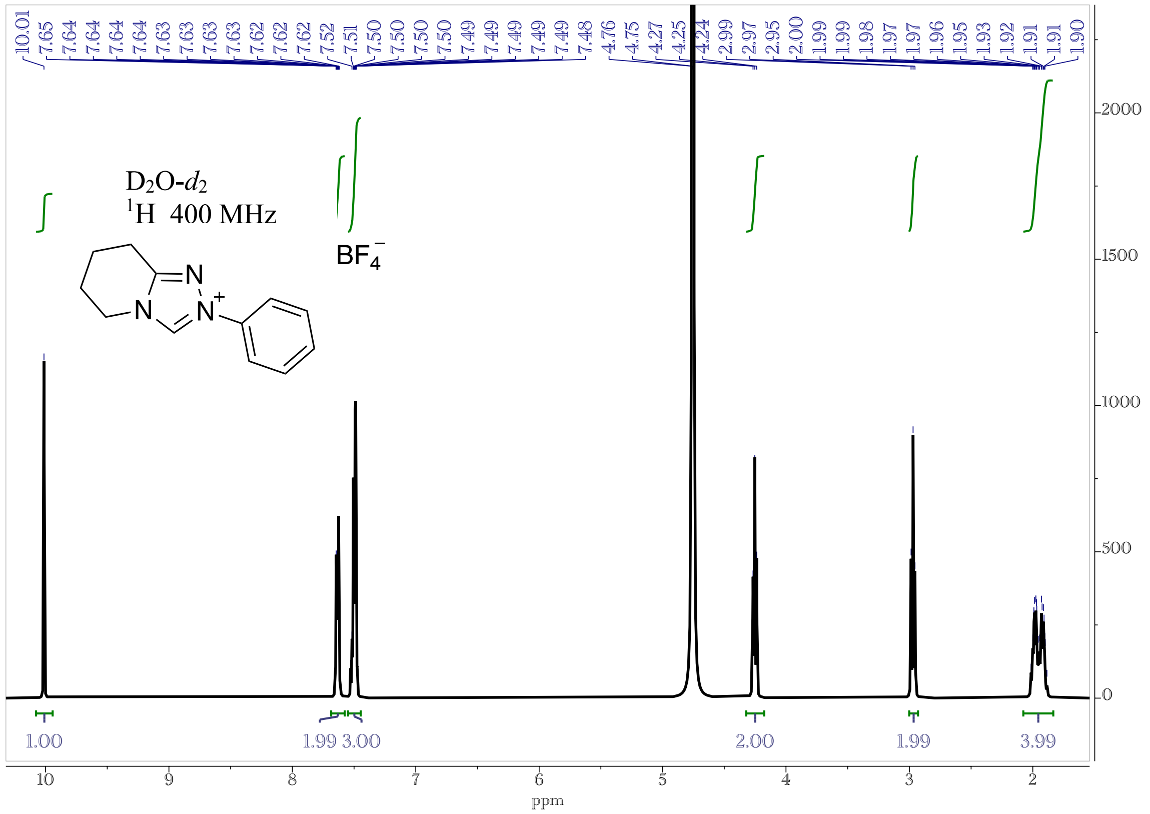


**8i**

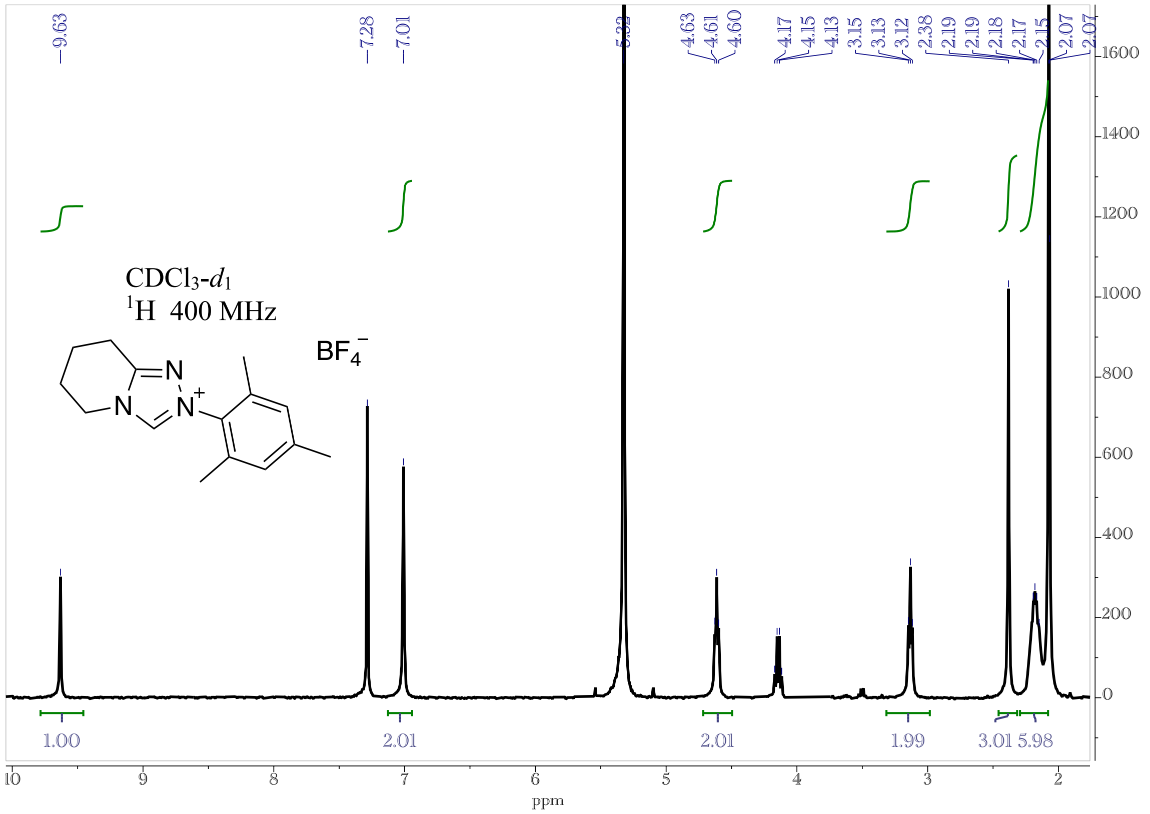

**8k**

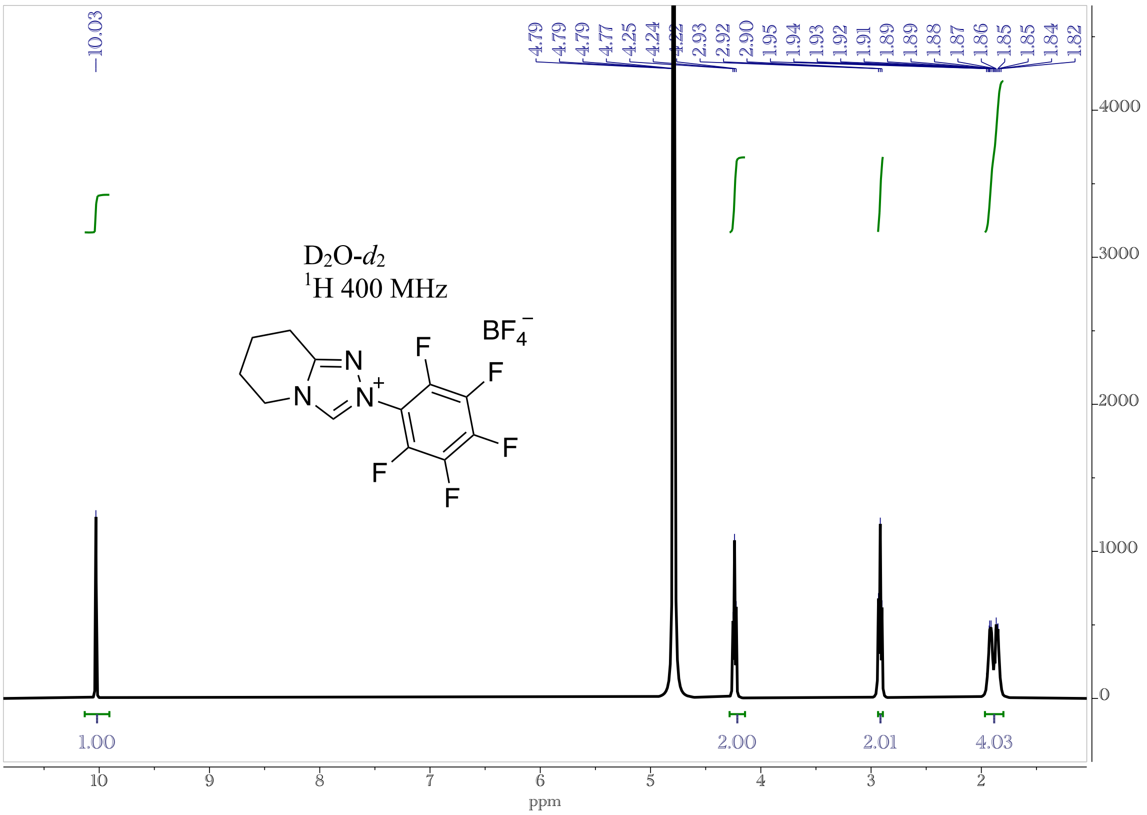


**9b**

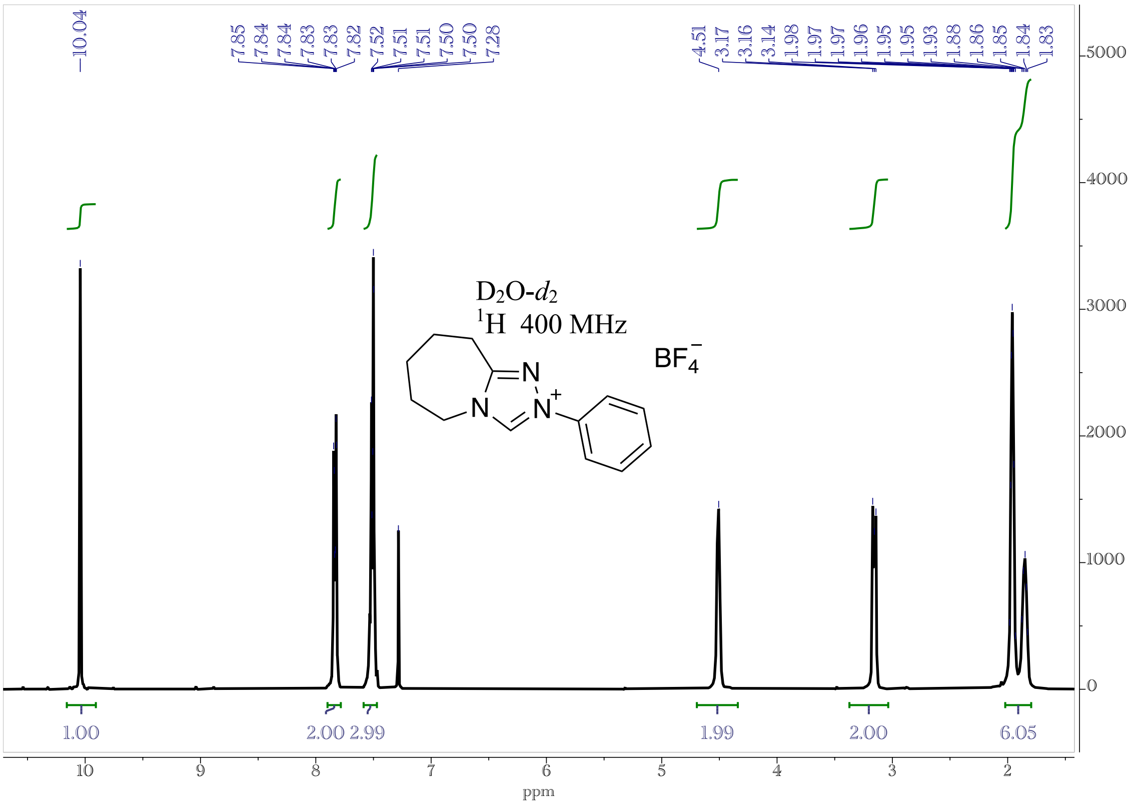

**9k**

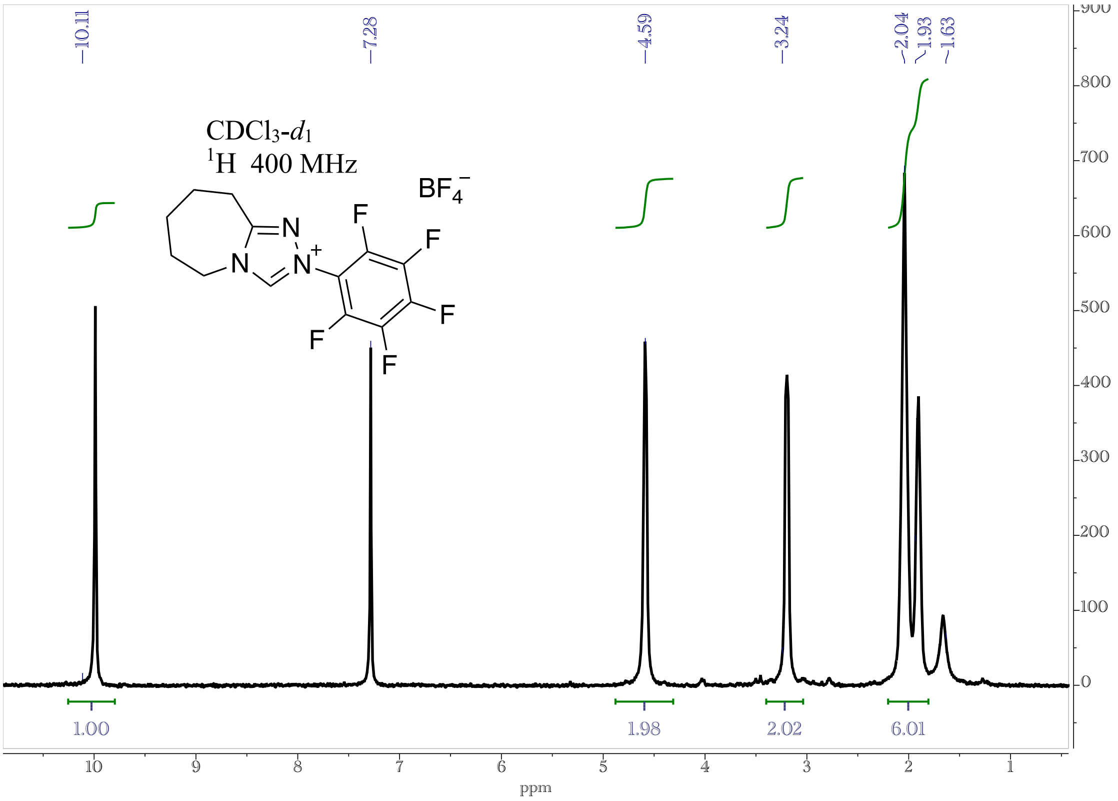

10.11
7.28
4.59
3.24
2.04
1.93
1.63
CDCl3-d1
1H 400 MHz
BF4−
N
N+
N
F
F
F
F
F
1.00
1.98
2.02
6.01
ppm

## S2. DFT Calculations

All the DFT calculations were performed using Gaussian 09 on the Durham University Hamilton HPC.[S10] The level of theory used for determining conformations for each molecule was B3LYP/6-31+g(d,p) for initial global minimima searches with subsequent structural refinement by B3LYP and M062X/6-311++g (d,p) unless stated otherwise, using redundant internal coordinates, with solvent water being modelled using an implicit polarisable continuum model (PCM).[S11] Gaussian NBO version 3.1 (within the Gaussian 09 package) was used for natural bond analysis (NBO) calculations.[S12] The imaginary frequencies of all the molecules were observed to be zero.

### S2.1 Conformation Search and Total Energies

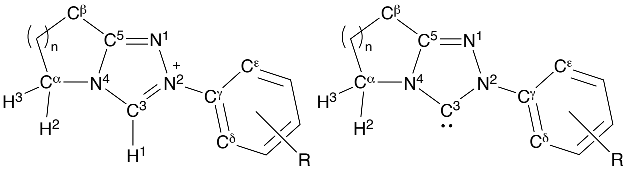

The conformations of molecules were obtained by optimizing structures with different dihedral angles between $N^1N^2$ and $C^{\gamma}C^{\varepsilon}$. The resulting structures and the corresponding energies (Hartrees) for different conformers are listed below. Only one structure is presented for conformations of the same energy. The structural differences between conformers are small.

### S2.1.1 Triazolium Salts

**7a**

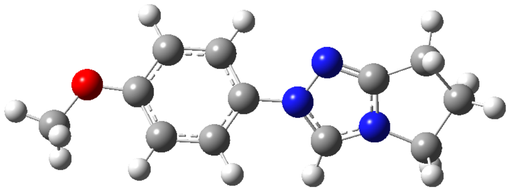

-705.053711

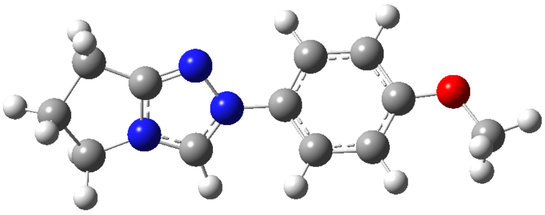

-705.053723

**7b**

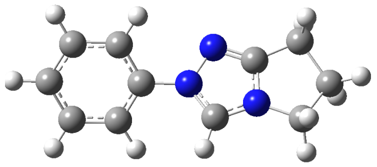

-590.521726

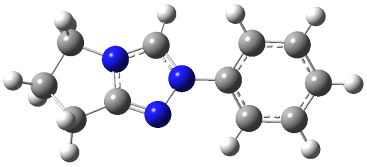

-590.521739

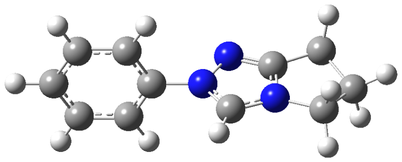

-590.520198

**7c**

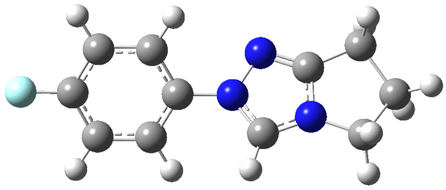

-689.760882

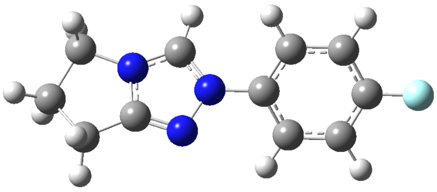

-689.760870

**7d**

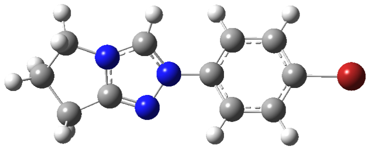

-3161.646932

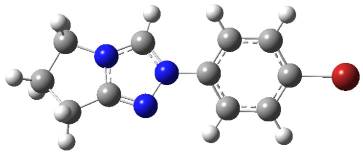

-3161.646959

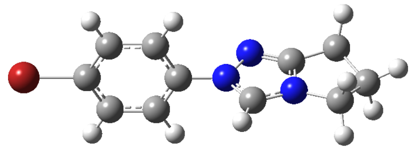

-3161.645600

**7e**

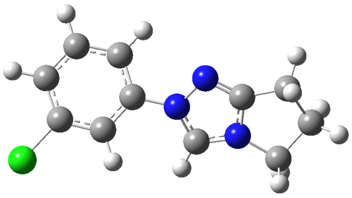

-1050.113187

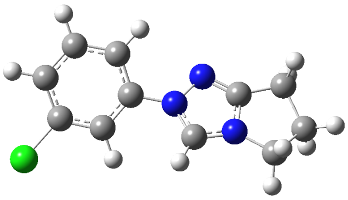

-1050.113159

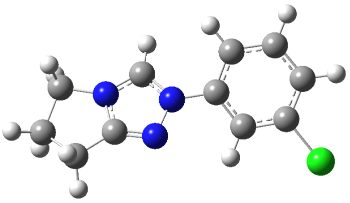

-1050.113409

**7f**

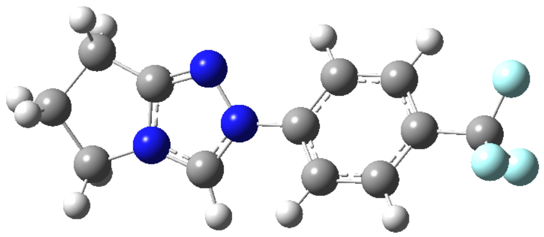

-927.578205

**7g**

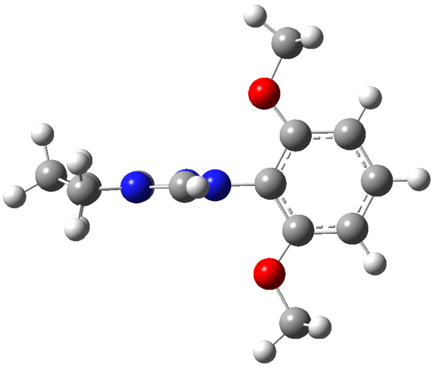

-819.580078

**7h**

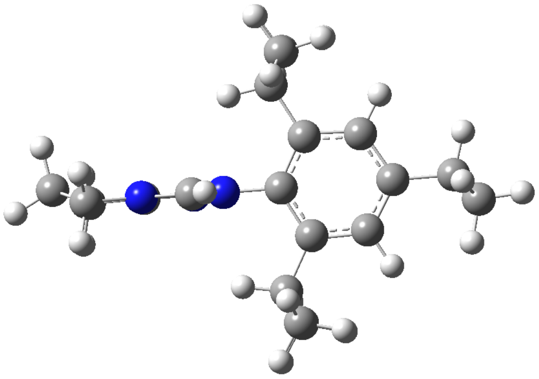

-944.381175

**7i**

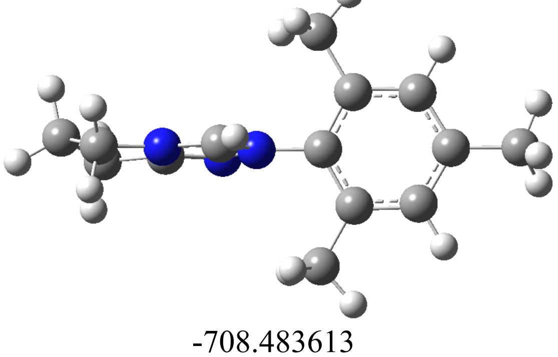

-708.483613

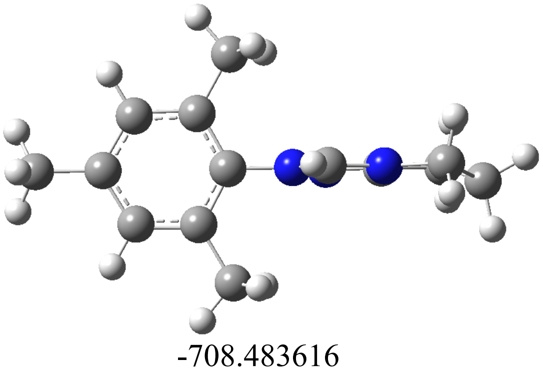

-708.483616

**7j**

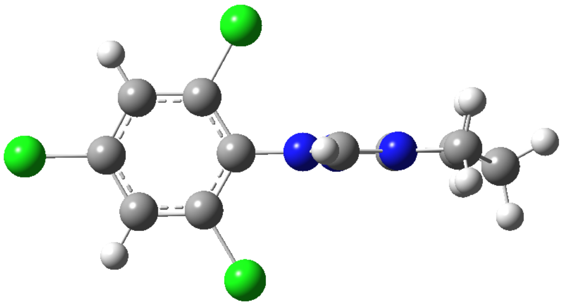

-1969.284916

**7k**

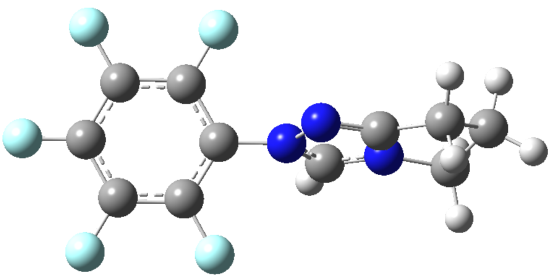

-1086.667585

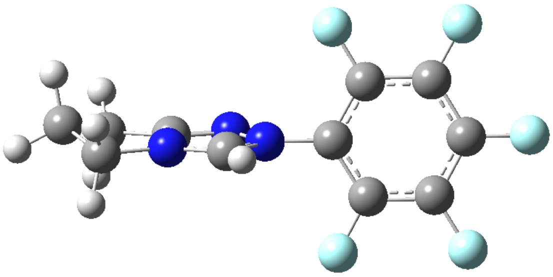

-1086.667686

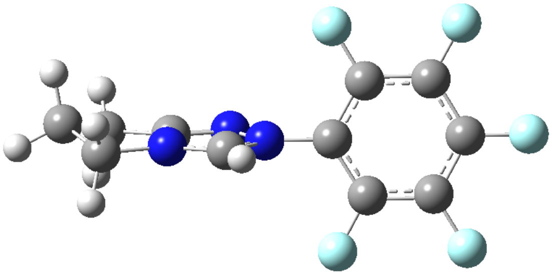

-1086.667685

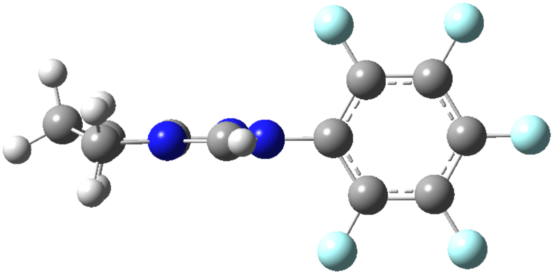

-1086.667724

**8a**

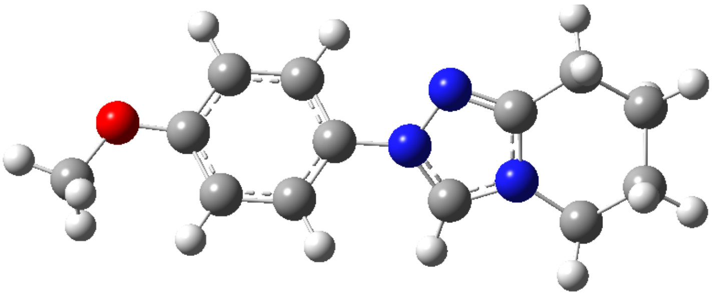

-744.38331

**8b**

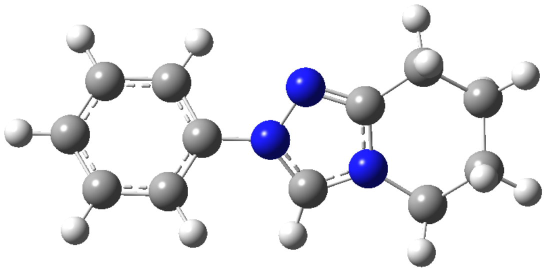

-629.851410

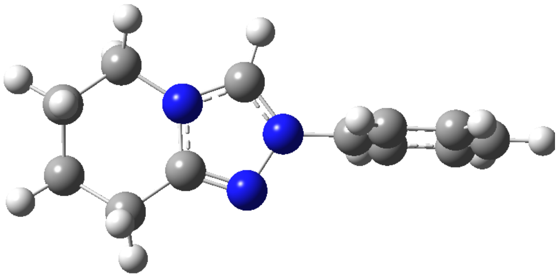

-629.849685

**8c**

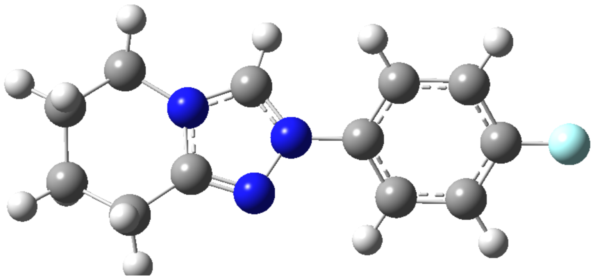

-729.09051

**8i**

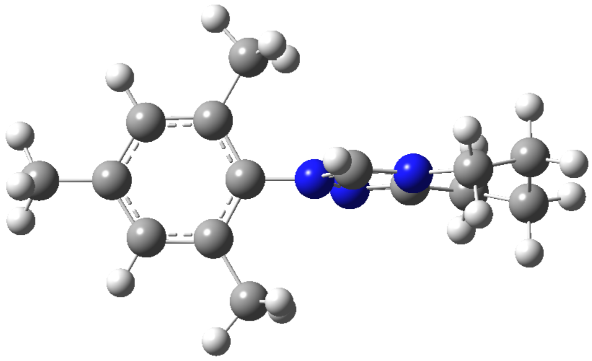

-747.813106

**8k**

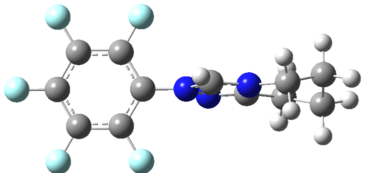

-1125.997132

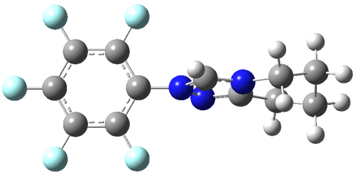

-1125.997032

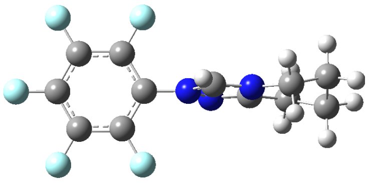

-1086.667585

**9a**

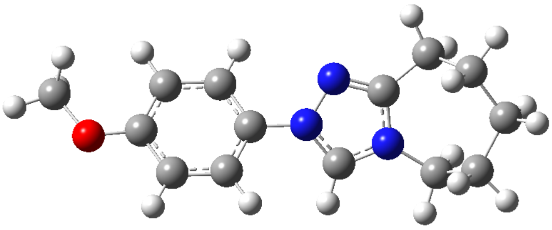

-783.700065

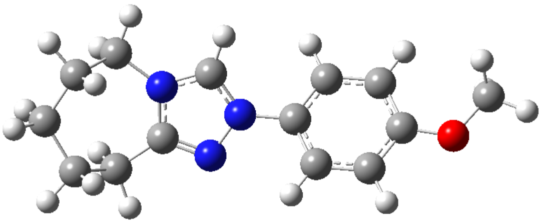

-783.700101

**9b**

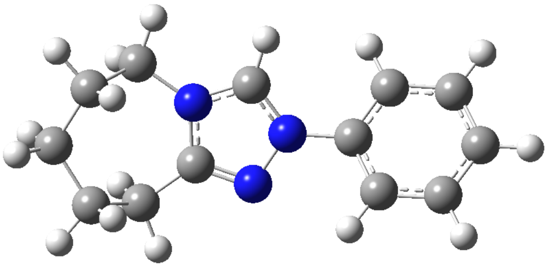

-669.168223

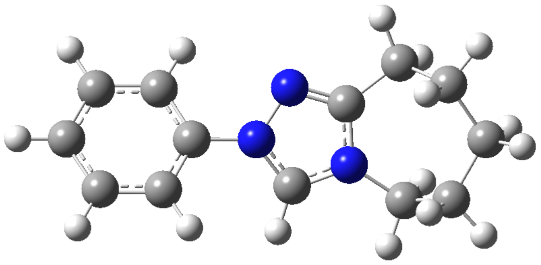

-669.168236

**9c**

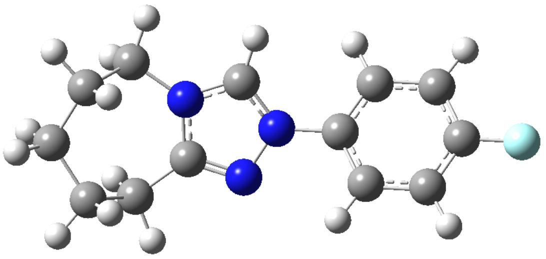

-768.407367

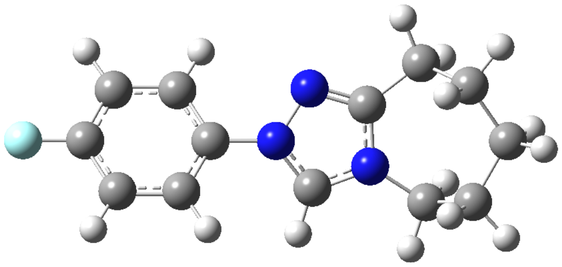

-768.407369

**9k**

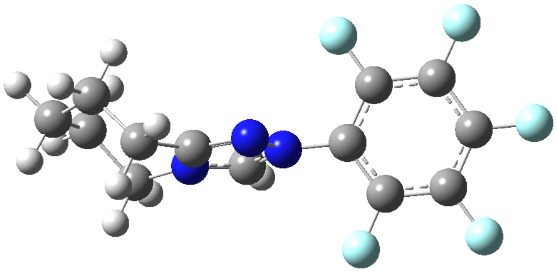

-1165.314026

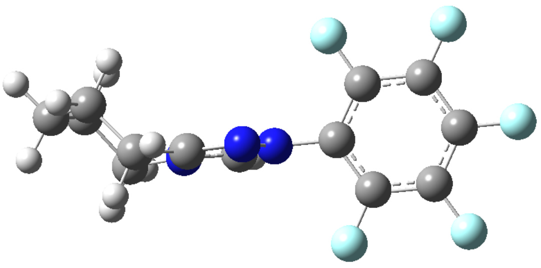

-1165.314167

## S2.1.2 Carbene

**7'a**

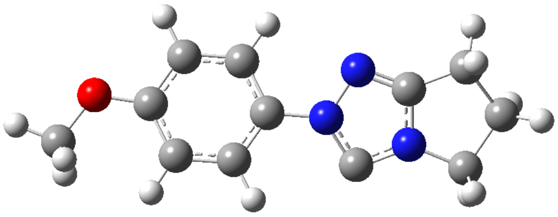

-704.573011

**7'b**

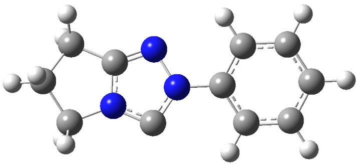

-590.042860

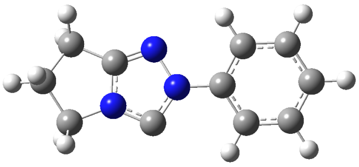

-590.042861

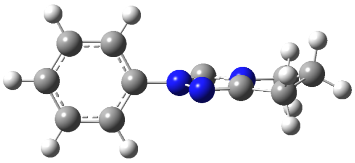

-590.03918

**7'c**

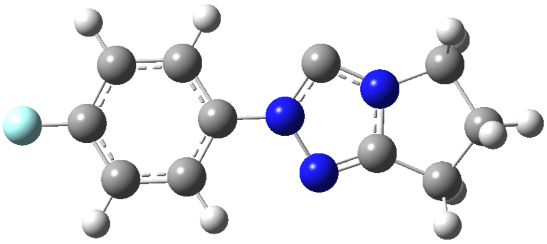

-689.282959

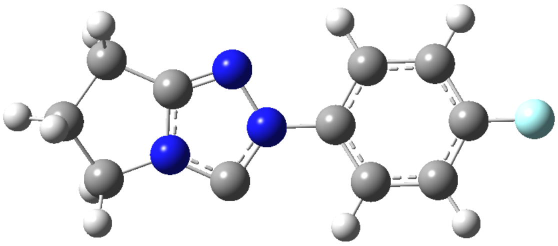

-689.282941

**7'd**

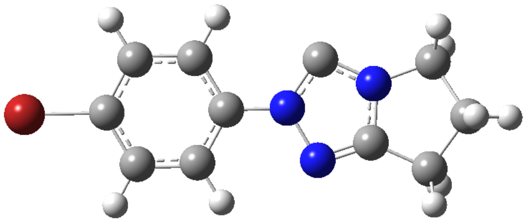

-3161.170224

**7’e**

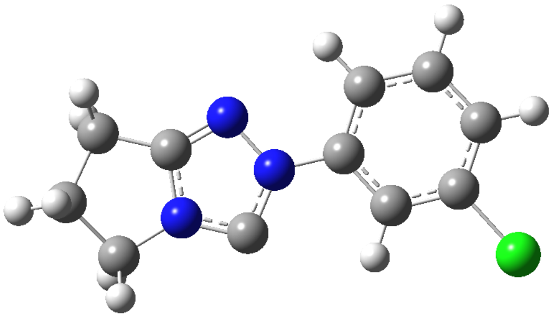

-1049.636892

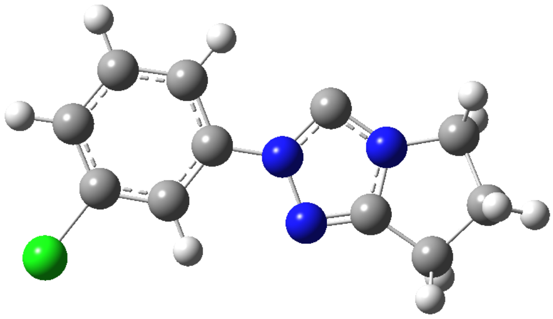

-1049.636966

**7’f**

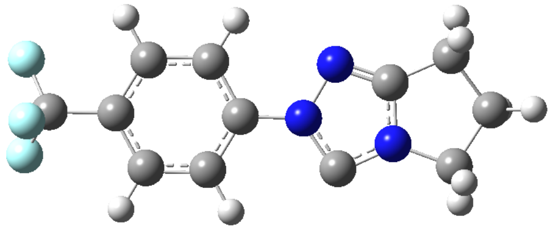

-927.103145

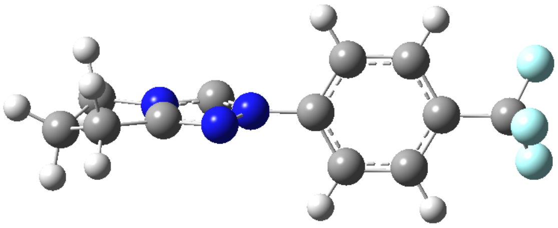

-927.097867

**7’g**

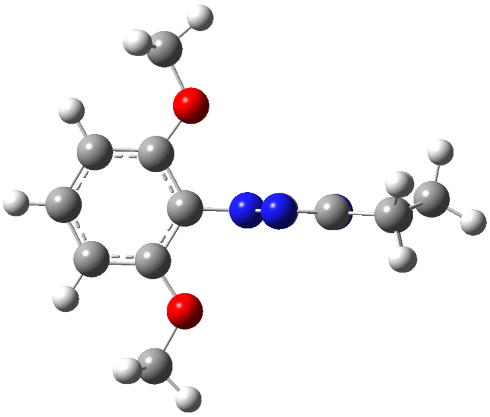

-819.097341

**7’h**

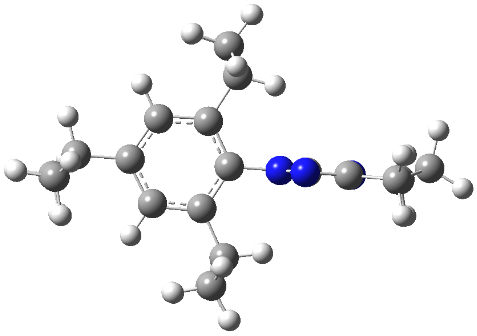

-943.898984

**7’i**

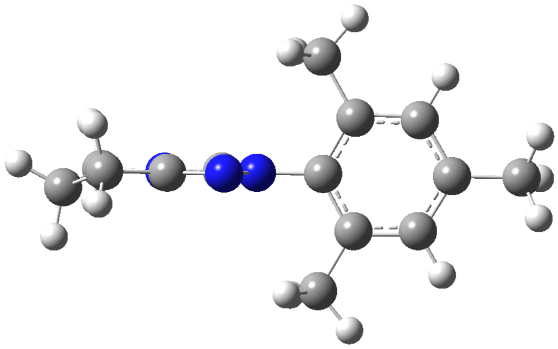

-708.001601

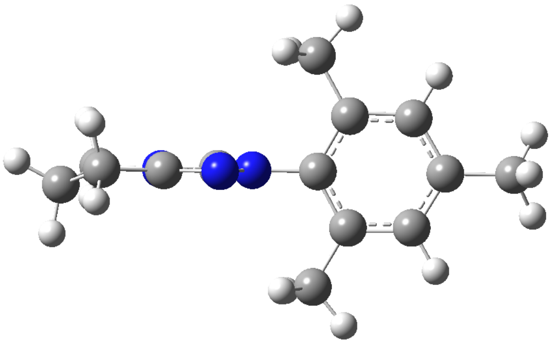

-708.001602

**7’j**

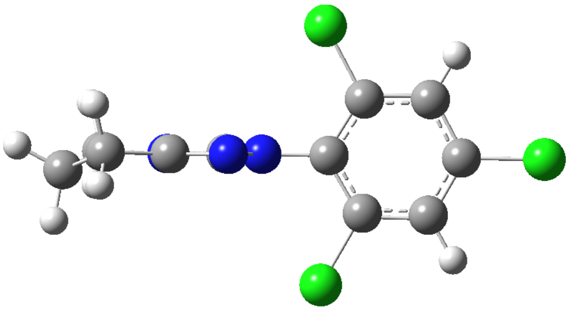

-1968.809921

**7’k**

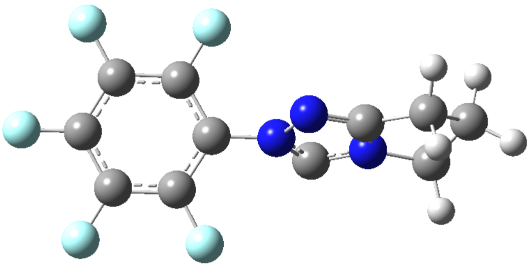

-1086.196025

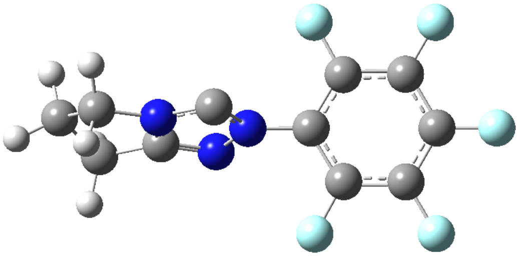

-1086.195986

**8’a**

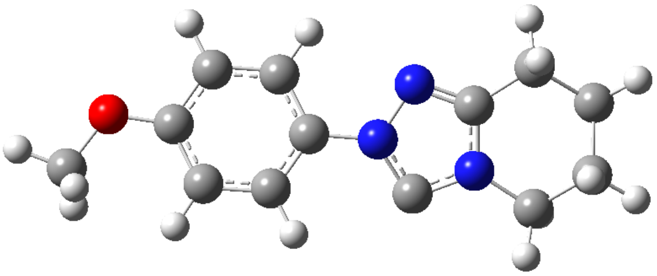

-743.901409

**8’b**

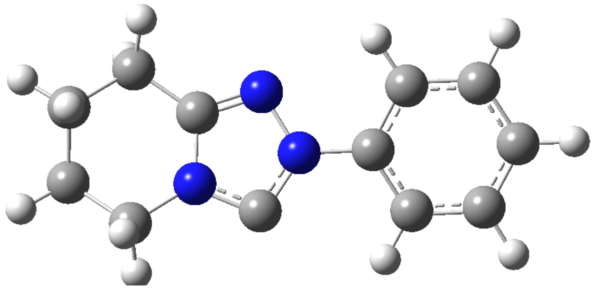

-728.611339

**8’c**

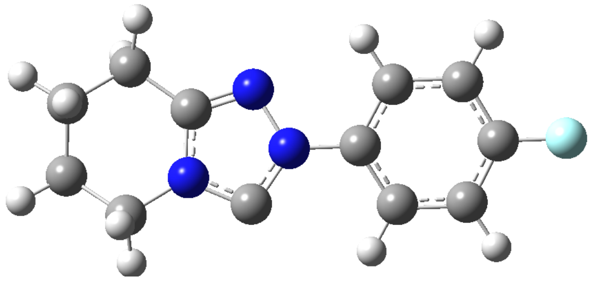

-728.611339

**8’i**

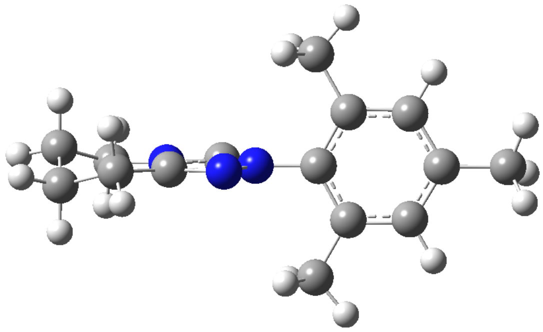

-747.329825

**8’k**

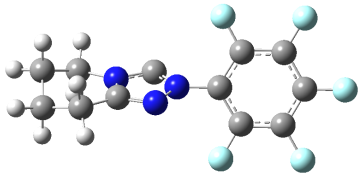

-1125.524142

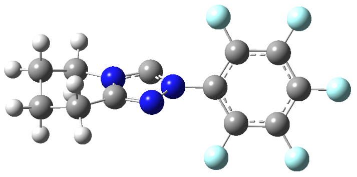

-1125.524143

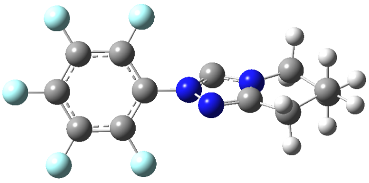

-1125.524139

**9’a**

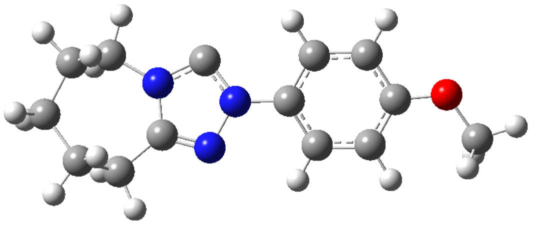

-783.218334

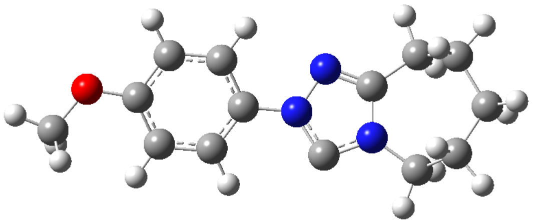

-783.218303

**9’b**

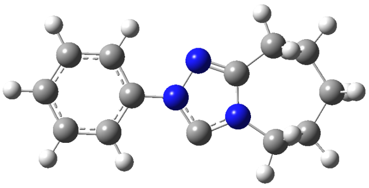

-668.688170

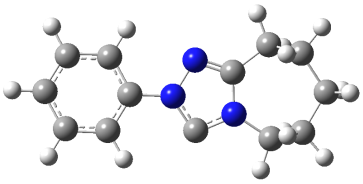

-668.688185

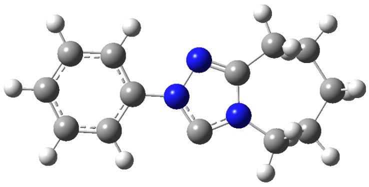

-668.688172

**9’c**

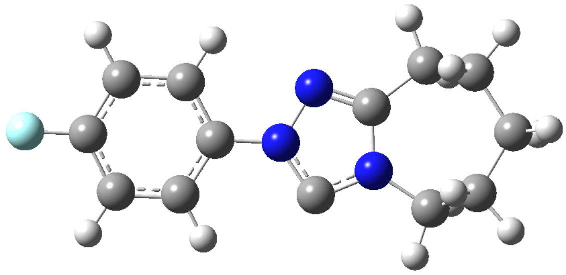

-767.928269

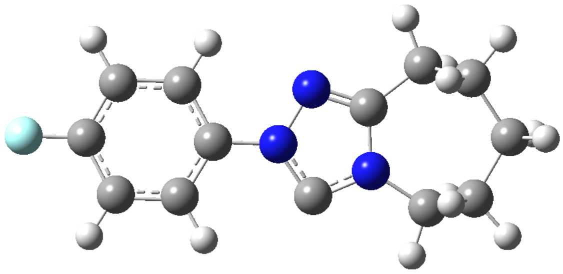

-767.928283

**9’k**

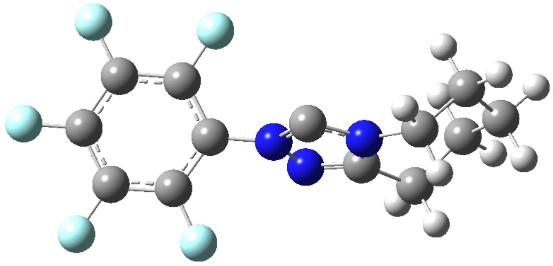

-1164.841146

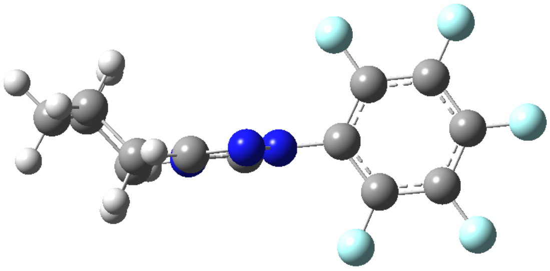

-1164.841100

## S2.2 Coordinates of Triazolium salts

### 7a (B3LYP)

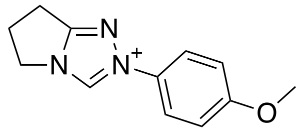

| | | | |
|---|---|---|---|
| O | 4.77379700 | 0.41104900 | -0.10491600 |
| N | -0.77315800 | 0.00387600 | -0.06778400 |
| N | -1.54293200 | 1.03797600 | 0.41556400 |
| N | -2.79399200 | -0.63793700 | -0.29134900 |
| C | -1.52909000 | -1.00402800 | -0.50582100 |
| H | -1.17725800 | -1.91805800 | -0.95262600 |
| C | -2.76843100 | 0.61378000 | 0.25838500 |
| C | -4.14932100 | 1.12716500 | 0.48785800 |
| H | -4.38901300 | 1.87664600 | -0.27122000 |
| H | -4.26307900 | 1.59030400 | 1.46693700 |
| C | -5.00282600 | -0.16532800 | 0.32324300 |
| H | -5.96336200 | 0.03675800 | -0.14636700 |
| H | -5.18797500 | -0.60686500 | 1.30287300 |
| C | -4.16064100 | -1.14768100 | -0.53126200 |
| H | -4.23891400 | -2.18365900 | -0.20998000 |
| H | -4.37296700 | -1.07685800 | -1.59818400 |
| C | 0.65729300 | 0.08157600 | -0.05533700 |
| C | 1.40668600 | -1.01083100 | 0.36413500 |
| H | 0.91979000 | -1.91239400 | 0.71543400 |
| C | 2.79820700 | -0.94046400 | 0.35285300 |

| | | | |
|---|---|---|---|
| H | 3.36822000 | -1.79652600 | 0.68433100 |
| C | 3.43074700 | 0.23652800 | -0.06360200 |
| C | 2.65884300 | 1.33729000 | -0.47027300 |
| H | 3.16236800 | 2.24058800 | -0.79129800 |
| C | 1.27756400 | 1.26214400 | -0.47104600 |
| H | 0.68149200 | 2.10530100 | -0.79518600 |
| C | 5.62588900 | -0.67089800 | 0.29117900 |
| H | 5.47250100 | -1.54413200 | -0.34819600 |
| H | 5.45693600 | -0.93948200 | 1.33718800 |
| H | 6.64193400 | -0.30294300 | 0.16873900 |

**M062X**

| | | | |
|---|---|---|---|
| O | 4.75436900 | 0.41668300 | -0.09537400 |
| N | -0.77146900 | 0.00283500 | -0.06518700 |
| N | -1.53285800 | 1.04363800 | 0.37850200 |
| N | -2.77998100 | -0.64346200 | -0.28297300 |
| C | -1.51860300 | -1.01490800 | -0.47769500 |
| H | -1.15839900 | -1.94188000 | -0.89347700 |
| C | -2.75473900 | 0.62069600 | 0.22941800 |
| C | -4.13785600 | 1.13684500 | 0.43714300 |
| H | -4.38461500 | 1.83010900 | -0.36997100 |
| H | -4.24847000 | 1.65426700 | 1.38740100 |
| C | -4.96800700 | -0.16977100 | 0.35342800 |
| H | -5.95365300 | -0.00409200 | -0.07433600 |
| H | -5.08791300 | -0.58675600 | 1.35319100 |

| | | | |
|---|---|---|---|
| C | -4.14539000 | -1.15230900 | -0.50849400 |
| H | -4.21952700 | -2.18708600 | -0.18527400 |
| H | -4.36705800 | -1.07438400 | -1.57236700 |
| C | 0.65760900 | 0.08119700 | -0.05154800 |
| C | 1.40272100 | -1.01937700 | 0.33586700 |
| H | 0.91443900 | -1.92942100 | 0.66350600 |
| C | 2.79239100 | -0.94680900 | 0.32385900 |
| H | 3.36463900 | -1.81181400 | 0.62782500 |
| C | 3.41715700 | 0.24136800 | -0.05819200 |
| C | 2.64597200 | 1.35109700 | -0.43069200 |
| H | 3.15231200 | 2.26243800 | -0.72270700 |
| C | 1.26791700 | 1.27360000 | -0.43403500 |
| H | 0.66410300 | 2.12190900 | -0.73061400 |
| C | 5.58338100 | -0.67826800 | 0.27588600 |
| H | 5.42011900 | -1.53101700 | -0.38764600 |
| H | 5.39798700 | -0.97162000 | 1.31202200 |
| H | 6.60592000 | -0.32535600 | 0.17336700 |

**7b (B3LYP)**

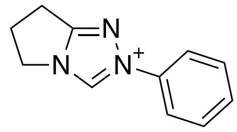

| | | | |
|---|---|---|---|
| N | 0.08613000 | 0.06190100 | 0.02057800 |
| N | -0.65230700 | -1.02355400 | 0.43186400 |
| N | -1.95113200 | 0.65032000 | -0.18821800 |
| C | -0.69869500 | 1.07265200 | -0.35799200 |
| H | -0.37429000 | 2.02224700 | -0.74802100 |
| C | -1.88953900 | -0.62882000 | 0.29321200 |
| C | -3.25499000 | -1.17578700 | 0.53554200 |
| H | -3.46309700 | -1.17535000 | 1.60888200 |
| H | -3.36334200 | -2.19617900 | 0.17093100 |
| C | -4.14794800 | -0.15631100 | -0.23294800 |
| H | -5.09895000 | 0.01213200 | 0.26818700 |
| H | -4.35221000 | -0.53431100 | -1.23514400 |
| C | -3.33174400 | 1.15805400 | -0.33652900 |
| H | -3.44455300 | 1.66998600 | -1.28936000 |
| H | -3.53214400 | 1.85220300 | 0.47980100 |
| C | 1.52058400 | 0.01840600 | 0.00995600 |
| C | 2.24022700 | 1.13516000 | 0.42889100 |
| H | 1.72961500 | 2.01730900 | 0.79481300 |
| C | 3.63194800 | 1.08800900 | 0.39198000 |
| H | 4.20223000 | 1.94989900 | 0.71603400 |
| C | 4.28470800 | -0.06588700 | -0.03980300 |

| | | | |
|---|---|---|---|
| C | 3.54592600 | -1.17953400 | -0.44127100 |
| H | 4.05123700 | -2.07665800 | -0.77755800 |
| C | 2.15458400 | -1.14383200 | -0.42352000 |
| H | 1.56888200 | -1.99539300 | -0.74377200 |
| H | 5.36741100 | -0.09922900 | -0.05946900 |

**M062X**

| | | | |
|---|---|---|---|
| N | 0.08247600 | 0.06673000 | 0.03663200 |
| N | -0.64632600 | -1.02524500 | 0.40410400 |
| N | -1.94404300 | 0.66061100 | -0.15526000 |
| C | -0.69610600 | 1.08799000 | -0.30609500 |
| H | -0.36596600 | 2.05022400 | -0.66233700 |
| C | -1.88035600 | -0.63224900 | 0.27890400 |
| C | -3.24714500 | -1.18518200 | 0.49702700 |
| H | -3.46905300 | -1.18030100 | 1.56642800 |
| H | -3.34541700 | -2.20228200 | 0.12475100 |
| C | -4.11666900 | -0.16340100 | -0.28003900 |
| H | -5.09759600 | -0.03238800 | 0.16997300 |
| H | -4.24680300 | -0.50706900 | -1.30614200 |
| C | -3.32462200 | 1.16219300 | -0.28658800 |
| H | -3.43099900 | 1.73396600 | -1.20445600 |
| H | -3.54153400 | 1.79141500 | 0.57611500 |
| C | 1.51497500 | 0.02144600 | 0.01598100 |
| C | 2.23512100 | 1.15161500 | 0.38081900 |

| | | | |
|---|---|---|---|
| H | 1.72875400 | 2.04880700 | 0.71531400 |
| C | 3.62357600 | 1.09799700 | 0.33308400 |
| H | 4.19936900 | 1.97090600 | 0.61398200 |
| C | 4.26712400 | -0.07353500 | -0.05371500 |
| C | 3.52388200 | -1.19937900 | -0.39992900 |
| H | 4.02428000 | -2.11149400 | -0.70003300 |
| C | 2.13551400 | -1.15861700 | -0.37302500 |
| H | 1.53938200 | -2.01862800 | -0.64958500 |
| H | 5.34907800 | -0.11110600 | -0.08088700 |

**7c (B3LYP)**

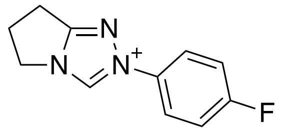


| | | | |
|---|---|---|---|
| N | 0.33370600 | -0.04402800 | 0.04626000 |
| N | 1.06509400 | 1.04031400 | -0.38172100 |
| N | 2.37410600 | -0.60812800 | 0.28442500 |
| C | 1.12456200 | -1.03635600 | 0.45906700 |
| H | 0.80531900 | -1.98234800 | 0.86221600 |
| C | 2.30458400 | 0.66234300 | -0.21909500 |
| C | 3.66623700 | 1.23924800 | -0.40726900 |
| H | 3.86427800 | 1.96795000 | 0.38338300 |
| H | 3.77419500 | 1.74401800 | -1.36620700 |
| C | 4.56954300 | -0.02347700 | -0.27864100 |
| H | 5.51495400 | 0.19967300 | 0.21155300 |
| H | 4.78446000 | -0.42028700 | -1.27123700 |
| C | 3.75748000 | -1.07024000 | 0.52728900 |
| H | 3.88167900 | -2.08990600 | 0.17054400 |
| H | 3.95080600 | -1.02888200 | 1.59928300 |
| C | -1.09917100 | -0.02488800 | 0.01256000 |
| C | -1.78916400 | -1.13807500 | -0.46200300 |
| H | -1.25632700 | -2.00283400 | -0.83645200 |
| C | -3.18056300 | -1.12147200 | -0.47155900 |
| H | -3.74839900 | -1.96779500 | -0.83503800 |
| C | -3.83112500 | 0.01730900 | -0.02339300 |

| | | | |
|---|---|---|---|
| C | -3.15581500 | 1.13846000 | 0.43686900 |
| H | -3.70719700 | 2.00472400 | 0.77833100 |
| C | -1.76621400 | 1.11436800 | 0.45894500 |
| H | -1.20761200 | 1.96627200 | 0.82292500 |
| F | -5.18540300 | 0.03877700 | -0.03985300 |

**M062X**

| | | | |
|---|---|---|---|
| N | 0.33512500 | -0.04486300 | 0.04539400 |
| N | 1.05674600 | 1.04452100 | -0.34406800 |
| N | 2.36439000 | -0.61277800 | 0.27560800 |
| C | 1.11903200 | -1.04567900 | 0.43235900 |
| H | 0.79307100 | -2.00291200 | 0.80620500 |
| C | 2.29296500 | 0.66860600 | -0.18998900 |
| C | 3.65578000 | 1.24842000 | -0.35752200 |
| H | 3.86341500 | 1.91876600 | 0.47940700 |
| H | 3.75617000 | 1.80707300 | -1.28520600 |
| C | 4.53810000 | -0.02577500 | -0.31258000 |
| H | 5.51162100 | 0.16366000 | 0.13262700 |
| H | 4.68499600 | -0.40006600 | -1.32555600 |
| C | 3.74727400 | -1.07234000 | 0.50279900 |
| H | 3.86787000 | -2.09091700 | 0.14429100 |
| H | 3.95111600 | -1.02256300 | 1.57183300 |
| C | -1.09586300 | -0.02645500 | 0.01084100 |
| C | -1.78104200 | -1.15288600 | -0.42527300 |

| | | | |
|---|---|---|---|
| H | -1.24578800 | -2.02811200 | -0.77225700 |
| C | -3.16950000 | -1.13468800 | -0.43281600 |
| H | -3.74309800 | -1.99028200 | -0.76407800 |
| C | -3.81583000 | 0.01839200 | -0.02455300 |
| C | -3.14068800 | 1.15266600 | 0.39494600 |
| H | -3.69512000 | 2.02956900 | 0.70259700 |
| C | -1.75413700 | 1.12757500 | 0.41770000 |
| H | -1.18743300 | 1.98851200 | 0.74785500 |
| F | -5.15857700 | 0.04134500 | -0.04130200 |

**7d (B3LYP)**

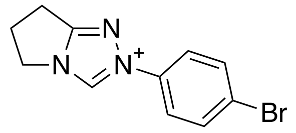


| | | | |
|---|---|---|---|
| N | -1.50113000 | 0.08407000 | 0.03221400 |
| N | -2.22537300 | -1.01486000 | 0.43353200 |
| N | -3.54508900 | 0.64167300 | -0.18951000 |
| C | -2.29912500 | 1.08458300 | -0.34863800 |
| H | -1.98751200 | 2.04093900 | -0.73298100 |
| C | -3.46723400 | -0.63883300 | 0.28735400 |
| C | -4.82562200 | -1.20797300 | 0.51674800 |
| H | -5.04230400 | -1.21312600 | 1.58839900 |
| H | -4.91442800 | -2.22911700 | 0.14902900 |
| C | -5.72791500 | -0.20060400 | -0.25673000 |
| H | -6.68641800 | -0.05008200 | 0.23563900 |
| H | -5.91578900 | -0.57800900 | -1.26233400 |
| C | -4.93288400 | 1.12752300 | -0.34714200 |
| H | -5.04566000 | 1.64242900 | -1.29835400 |
| H | -5.15113200 | 1.81401000 | 0.47105400 |
| C | -0.06857700 | 0.05765700 | 0.03005500 |
| C | 0.58216800 | -1.10014700 | -0.38865400 |
| H | 0.01237800 | -1.96328500 | -0.70583900 |
| C | 1.97245400 | -1.13102500 | -0.40038500 |
| H | 2.48974300 | -2.02351300 | -0.72551200 |
| C | 2.68238400 | -0.00067900 | -0.00178500 |

| | | | |
|---|---|---|---|
| C | 2.03213500 | 1.15595100 | 0.41844000 |
| H | 2.59361600 | 2.02316500 | 0.73848500 |
| C | 0.64059000 | 1.18213800 | 0.44369200 |
| H | 0.12630700 | 2.06541600 | 0.80071700 |
| Br | 4.59691700 | -0.04218800 | -0.02599100 |

**M062X**

| | | | |
|---|---|---|---|
| N | -1.49531600 | 0.08831300 | 0.04944700 |
| N | -2.20947000 | -1.01732300 | 0.40529300 |
| N | -3.52861400 | 0.65017100 | -0.15958200 |
| C | -2.28739900 | 1.09890500 | -0.29683400 |
| H | -1.97071700 | 2.06830900 | -0.64613300 |
| C | -3.44822200 | -0.64382900 | 0.26994600 |
| C | -4.80771400 | -1.21931000 | 0.47365700 |
| H | -5.03839700 | -1.22200900 | 1.54123200 |
| H | -4.88623700 | -2.23642600 | 0.09677500 |
| C | -5.68676900 | -0.20835000 | -0.30683600 |
| H | -6.67305800 | -0.09426000 | 0.13594400 |
| H | -5.80348100 | -0.55013400 | -1.33511900 |
| C | -4.91623900 | 1.12977700 | -0.30186800 |
| H | -5.02304700 | 1.70321500 | -1.21865500 |
| H | -5.15065100 | 1.75216300 | 0.56115000 |
| C | -0.06456300 | 0.06210300 | 0.04186000 |
| C | 0.57573900 | -1.11260600 | -0.32970600 |

| | | | |
|---|---|---|---|
| H | -0.00272700 | -1.98517600 | -0.60374300 |
| C | 1.96307200 | -1.14564900 | -0.34625900 |
| H | 2.48198000 | -2.05083200 | -0.63213300 |
| C | 2.67380100 | -0.00125900 | -0.00173700 |
| C | 2.02977600 | 1.17173300 | 0.37118400 |
| H | 2.59792300 | 2.04926900 | 0.64953000 |
| C | 0.64142900 | 1.20150100 | 0.40324600 |
| H | 0.12800100 | 2.09870400 | 0.72579200 |
| Br | 4.56962100 | -0.04511500 | -0.03430500 |

**7e (B3LYP)**

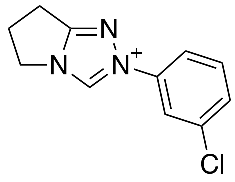


| | | | |
|---|---|---|---|
| N | -0.59942900 | 0.35076300 | 0.05761900 |
| N | -1.14202500 | -0.79585700 | 0.58992100 |
| N | -2.69616800 | 0.50757300 | -0.28147400 |
| C | -1.53894900 | 1.13763200 | -0.47309700 |
| H | -1.37841100 | 2.07522600 | -0.97706100 |
| C | -2.42203500 | -0.66596900 | 0.36785900 |
| C | -3.67232200 | -1.42670800 | 0.64952800 |
| H | -3.92669900 | -1.32875200 | 1.70833900 |
| H | -3.57198300 | -2.48703700 | 0.42268000 |
| C | -4.70110600 | -0.70117700 | -0.26798700 |
| H | -5.68904600 | -0.65308000 | 0.18524300 |
| H | -4.78491500 | -1.23543400 | -1.21479000 |
| C | -4.13850800 | 0.71977900 | -0.52870400 |
| H | -4.29811800 | 1.07503600 | -1.54386000 |
| H | -4.50265600 | 1.46158800 | 0.18225600 |
| C | 0.81464900 | 0.58033000 | 0.09413700 |
| C | 1.29754600 | 1.86670000 | 0.31731800 |
| H | 0.62105400 | 2.69195100 | 0.49818800 |
| C | 2.67519600 | 2.06841100 | 0.33000700 |
| H | 3.06853200 | 3.06206100 | 0.50344200 |

| | | | |
|---|---|---|---|
| C | 3.55352600 | 1.00383100 | 0.14334700 |
| C | 3.03381300 | -0.27148500 | -0.06271600 |
| C | 1.66507900 | -0.50608000 | -0.09683600 |
| H | 1.26745800 | -1.49621500 | -0.26864000 |
| Cl | 4.13134700 | -1.62322000 | -0.29757200 |
| H | 4.62390200 | 1.16047000 | 0.16333100 |

**M062X**

| | | | |
|---|---|---|---|
| N | -0.59968100 | 0.35599300 | 0.06918800 |
| N | -1.12820500 | -0.79825600 | 0.56566000 |
| N | -2.68783200 | 0.51983100 | -0.25277100 |
| C | -1.53738400 | 1.15634900 | -0.42998800 |
| H | -1.37696100 | 2.11169300 | -0.90271400 |
| C | -2.40573800 | -0.66915500 | 0.35794000 |
| C | -3.65432300 | -1.43876700 | 0.62060600 |
| H | -3.92236900 | -1.33440300 | 1.67430300 |
| H | -3.54330900 | -2.49619700 | 0.39206400 |
| C | -4.66127600 | -0.71335000 | -0.30826100 |
| H | -5.66890600 | -0.71252500 | 0.09950700 |
| H | -4.67891300 | -1.20817200 | -1.27922500 |
| C | -4.13048700 | 0.72594800 | -0.48252100 |
| H | -4.29772600 | 1.14027600 | -1.47285800 |
| H | -4.50060700 | 1.41151100 | 0.27905000 |
| C | 0.81319400 | 0.58347200 | 0.09446100 |

| | | | |
|---|---|---|---|
| C | 1.29537300 | 1.86912200 | 0.29595100 |
| H | 0.61833200 | 2.69597100 | 0.46897400 |
| C | 2.67135700 | 2.06603000 | 0.29796200 |
| H | 3.06961900 | 3.06035500 | 0.45352200 |
| C | 3.54193000 | 0.99716800 | 0.12189700 |
| C | 3.01851500 | -0.27718300 | -0.06235100 |
| C | 1.65176700 | -0.50823500 | -0.08549100 |
| H | 1.24661500 | -1.49947400 | -0.23854800 |
| Cl | 4.10194700 | -1.62394000 | -0.28250800 |
| H | 4.61358900 | 1.14657000 | 0.13278300 |

**7f (B3LYP)**

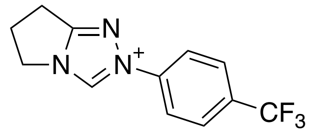


| | | | |
|---|---|---|---|
| N | -1.43454200 | 0.09553100 | 0.03368300 |
| N | -2.15040700 | -1.02485600 | 0.38779700 |
| N | -3.48229900 | 0.64983800 | -0.15534800 |
| C | -2.24123300 | 1.10795100 | -0.29839700 |
| H | -1.93956900 | 2.08280900 | -0.64180900 |
| C | -3.39464400 | -0.65081400 | 0.26410300 |
| C | -4.74867400 | -1.23749700 | 0.47309100 |
| H | -4.96261400 | -1.28565900 | 1.54426400 |
| H | -4.83151100 | -2.24398700 | 0.06589000 |
| C | -5.65823200 | -0.20567400 | -0.25817500 |
| H | -6.61923900 | -0.08513400 | 0.23744100 |
| H | -5.83954600 | -0.54028500 | -1.28005500 |
| C | -4.87439200 | 1.13157800 | -0.28770900 |
| H | -4.99290300 | 1.69025100 | -1.21306700 |
| H | -5.09572400 | 1.77642400 | 0.56296500 |
| C | -0.00250200 | 0.07177500 | 0.01921600 |
| C | 0.64265400 | -1.10403600 | -0.35640200 |
| H | 0.06806500 | -1.97767100 | -0.63110000 |
| C | 2.03115300 | -1.13159100 | -0.37796100 |
| H | 2.54227900 | -2.03852500 | -0.67289000 |
| C | 2.75634500 | 0.01138900 | -0.03761200 |

| | | | |
|---|---|---|---|
| C | 2.09828900 | 1.18195800 | 0.33841600 |
| H | 2.65952400 | 2.06557200 | 0.61170000 |
| C | 0.70886400 | 1.21445700 | 0.37596200 |
| H | 0.19963200 | 2.11372100 | 0.69654500 |
| C | 4.25995500 | -0.04073500 | -0.02032100 |
| F | 4.73662200 | -0.46647400 | 1.18024300 |
| F | 4.82115900 | 1.16760200 | -0.24732100 |
| F | 4.75520300 | -0.88931300 | -0.94937600 |

**M062X**

| | | | |
|---|---|---|---|
| N | -1.42878500 | 0.09396800 | 0.04403500 |
| N | -2.13765600 | -1.01888400 | 0.38844500 |
| N | -3.46423600 | 0.65281700 | -0.14637800 |
| C | -2.22631400 | 1.10743700 | -0.28394300 |
| H | -1.91565200 | 2.08286800 | -0.62189300 |
| C | -3.37776800 | -0.64713300 | 0.26623200 |
| C | -4.73443500 | -1.22869400 | 0.47052800 |
| H | -4.95899800 | -1.24391400 | 1.53930400 |
| H | -4.81197800 | -2.24172800 | 0.08266600 |
| C | -5.62042600 | -0.21142200 | -0.29362300 |
| H | -6.60483400 | -0.10568500 | 0.15532700 |
| H | -5.74117700 | -0.54120200 | -1.32534100 |
| C | -4.85440800 | 1.12914100 | -0.27633000 |
| H | -4.96714600 | 1.71326400 | -1.18559800 |

| | | | |
|---|---|---|---|
| H | -5.08634800 | 1.74035800 | 0.59526900 |
| C | 0.00171600 | 0.06891400 | 0.02557300 |
| C | 0.63558300 | -1.11163700 | -0.33869000 |
| H | 0.05221700 | -1.98409700 | -0.60064900 |
| C | 2.02208700 | -1.14098200 | -0.36454200 |
| H | 2.53762300 | -2.04922200 | -0.64939000 |
| C | 2.74108300 | 0.00480800 | -0.04012200 |
| C | 2.09398000 | 1.18023000 | 0.32461800 |
| H | 2.66344500 | 2.06368100 | 0.58449300 |
| C | 0.70747100 | 1.21519400 | 0.36780600 |
| H | 0.19480900 | 2.11560000 | 0.68116600 |
| C | 4.24188500 | -0.04196800 | -0.02362700 |
| F | 4.71549000 | -0.35481700 | 1.19663400 |
| F | 4.78649400 | 1.13702900 | -0.35445200 |
| F | 4.72802200 | -0.95871900 | -0.87122100 |

**7g (B3LYP)**

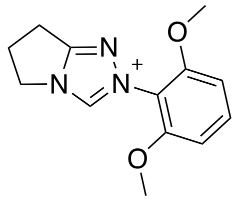

| | | | |
|---|---|---|---|
| N | -0.35075300 | -0.03309100 | 0.04184500 |
| N | -1.09160500 | -0.02664900 | -1.12234700 |
| N | -2.38594900 | -0.08594500 | 0.66695900 |
| C | -1.13353600 | -0.07216700 | 1.11782800 |
| H | -0.80064200 | -0.08268100 | 2.14201800 |
| C | -2.32640500 | -0.06179500 | -0.70227200 |
| C | -3.69412100 | -0.10819500 | -1.29577400 |
| H | -3.88616600 | -1.10969100 | -1.68999800 |
| H | -3.81781900 | 0.60464800 | -2.10968400 |
| C | -4.58965100 | 0.21433100 | -0.06270900 |
| H | -5.53236600 | -0.32838400 | -0.08929500 |
| H | -4.81053800 | 1.28202600 | -0.04214800 |
| C | -3.76454300 | -0.16299200 | 1.19452000 |
| H | -3.88979700 | 0.52827900 | 2.02450200 |
| H | -3.94671400 | -1.18264200 | 1.53449700 |
| C | 1.07823400 | 0.00772200 | 0.01877600 |
| C | 1.79462400 | -1.19980800 | -0.00621000 |
| C | 3.19167900 | -1.15467300 | -0.04034000 |
| H | 3.77616000 | -2.06296600 | -0.05793500 |
| C | 3.82721800 | 0.08435900 | -0.05295000 |
| C | 3.12362500 | 1.28593400 | -0.03148400 |

| | | | |
|---|---|---|---|
| H | 3.65660800 | 2.22543700 | -0.04181800 |
| C | 1.72631900 | 1.25333100 | 0.00296900 |
| H | 4.91025600 | 0.11470400 | -0.07974100 |
| O | 1.05043000 | -2.32733300 | 0.00928800 |
| O | 0.92071200 | 2.33751100 | 0.02760600 |
| C | 1.72020700 | -3.59692700 | -0.00171500 |
| H | 2.35269100 | -3.71010900 | 0.88166300 |
| H | 0.92841700 | -4.34190300 | 0.01573600 |
| H | 2.31629400 | -3.71396100 | -0.90952700 |
| C | 1.51875200 | 3.64255400 | 0.02020000 |
| H | 2.10604600 | 3.79552100 | -0.88802900 |
| H | 0.68673100 | 4.34219200 | 0.04135800 |
| H | 2.14544500 | 3.78785100 | 0.90303300 |

**M062X**

| | | | |
|---|---|---|---|
| N | -0.35153400 | -0.03559400 | 0.03964100 |
| N | -1.08217300 | -0.01348900 | -1.11479800 |
| N | -2.37467900 | -0.10522900 | 0.66497200 |
| C | -1.12639600 | -0.09521800 | 1.11423100 |
| H | -0.78569700 | -0.12067200 | 2.13680500 |
| C | -2.31391200 | -0.05876800 | -0.70028100 |
| C | -3.68278100 | -0.10158800 | -1.29040300 |
| H | -3.87887200 | -1.10852400 | -1.66519500 |
| H | -3.80241300 | 0.60455200 | -2.10880900 |

| | | | |
|---|---|---|---|
| C | -4.56043200 | 0.23904300 | -0.05834200 |
| H | -5.52586100 | -0.25973100 | -0.09172900 |
| H | -4.72595000 | 1.31558700 | -0.01896300 |
| C | -3.75158500 | -0.19443500 | 1.18377900 |
| H | -3.87392200 | 0.46397000 | 2.03947800 |
| H | -3.93988400 | -1.22747800 | 1.47437000 |
| C | 1.07422600 | 0.00843100 | 0.01882300 |
| C | 1.78603100 | -1.19461900 | -0.00876500 |
| C | 3.18019300 | -1.14792300 | -0.04053600 |
| H | 3.76875700 | -2.05374000 | -0.05907300 |
| C | 3.80830900 | 0.09233000 | -0.04981100 |
| C | 3.10524400 | 1.29168400 | -0.02755700 |
| H | 3.63730100 | 2.23174500 | -0.03632600 |
| C | 1.71091200 | 1.25267700 | 0.00510300 |
| H | 4.89091500 | 0.12571900 | -0.07506200 |
| O | 1.04272500 | -2.31475900 | 0.00223600 |
| O | 0.90086900 | 2.32536600 | 0.03032500 |
| C | 1.72665400 | -3.56588700 | -0.01126800 |
| H | 2.36291900 | -3.66548000 | 0.87050400 |
| H | 0.95065200 | -4.32602900 | 0.00650600 |
| H | 2.32390600 | -3.66739500 | -0.91971000 |
| C | 1.50786100 | 3.61562900 | 0.02764900 |
| H | 2.09532700 | 3.76212500 | -0.88106800 |
| H | 0.68735300 | 4.32706200 | 0.05443200 |
| H | 2.13946500 | 3.74546800 | 0.90890500 |

**7h (B3LYP)**

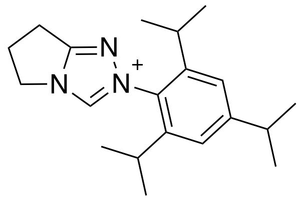

| | | | |
|---|---|---|---|
| N | -1.27531800 | -0.04804900 | 0.04704300 |
| N | -2.00925300 | -0.11911200 | -1.11845300 |
| N | -3.31258200 | -0.16844600 | 0.66369200 |
| C | -2.06351400 | -0.08108100 | 1.11993900 |
| H | -1.73812300 | -0.03794400 | 2.14571300 |
| C | -3.24568900 | -0.19470600 | -0.70370800 |
| C | -4.60551300 | -0.32665800 | -1.30202700 |
| H | -4.74937600 | -1.35166800 | -1.65430600 |
| H | -4.75494200 | 0.34487100 | -2.14613700 |
| C | -5.52229000 | 0.00657200 | -0.08775400 |
| H | -6.44101100 | -0.57635500 | -0.09731100 |
| H | -5.78814900 | 1.06389500 | -0.11138400 |
| C | -4.69024500 | -0.28472800 | 1.18752800 |
| H | -4.85066100 | 0.43297400 | 1.98848300 |
| H | -4.83012400 | -1.29702000 | 1.56700400 |
| C | 0.16802100 | 0.06161800 | 0.02421100 |
| C | 0.92771700 | -1.12101000 | 0.00906100 |
| C | 2.31589500 | -0.98108600 | -0.00471000 |
| H | 2.92637100 | -1.87645900 | -0.01221700 |
| C | 2.93913500 | 0.27018800 | -0.00973100 |

| | | | |
|---|---|---|---|
| C | 2.13599100 | 1.41097200 | -0.00664100 |
| H | 2.61322300 | 2.38474800 | -0.01505400 |
| C | 0.73995900 | 1.34269500 | 0.00567500 |
| C | 0.30150600 | -2.51137200 | 0.00879900 |
| H | -0.78436900 | -2.40135800 | -0.01028700 |
| C | -0.08636300 | 2.62362900 | -0.00223500 |
| H | -1.14366200 | 2.35195200 | -0.01832500 |
| C | 4.45565000 | 0.39569600 | -0.02103900 |
| H | 4.68573200 | 1.46629900 | -0.02327500 |
| C | 0.65573400 | -3.28910400 | 1.29043200 |
| H | 1.73119800 | -3.47355500 | 1.36096100 |
| H | 0.34688500 | -2.74227400 | 2.18509900 |
| H | 0.14908800 | -4.25808900 | 1.29219700 |
| C | 0.69106200 | -3.30606600 | -1.25187500 |
| H | 0.41171800 | -2.76864100 | -2.16155000 |
| H | 1.76671400 | -3.49861800 | -1.28830200 |
| H | 0.17890700 | -4.27211400 | -1.25657400 |
| C | 0.15049900 | 3.45447800 | 1.27303700 |
| H | -0.07283200 | 2.87443600 | 2.17216700 |
| H | 1.18742800 | 3.79495500 | 1.33954700 |
| H | -0.49290400 | 4.33860300 | 1.26996000 |
| C | 0.17991000 | 3.45712900 | -1.26990800 |
| H | 1.21489800 | 3.80740300 | -1.30873900 |
| H | -0.01499500 | 2.87548500 | -2.17437200 |
| H | -0.47087300 | 4.33572800 | -1.28311400 |

| | | | |
|---|---|---|---|
| C | 5.07051000 | -0.21230200 | -1.29517900 |
| H | 6.15172300 | -0.04823200 | -1.30814400 |
| H | 4.89626400 | -1.29110400 | -1.34647400 |
| H | 4.64739800 | 0.24170900 | -2.19513300 |
| C | 5.08877700 | -0.21107300 | 1.24473400 |
| H | 6.17004100 | -0.04676000 | 1.24230200 |
| H | 4.67843800 | 0.24351600 | 2.15031500 |
| H | 4.91554600 | -1.28987500 | 1.29931600 |

**M062X**

| | | | |
|---|---|---|---|
| N | -1.26729600 | -0.06340600 | 0.04074000 |
| N | -1.99164900 | -0.10412300 | -1.11553700 |
| N | -3.29093900 | -0.20570300 | 0.65808400 |
| C | -2.04495000 | -0.12714500 | 1.11300600 |
| H | -1.70921000 | -0.10962000 | 2.13782900 |
| C | -3.22485000 | -0.19408800 | -0.70597200 |
| C | -4.58635000 | -0.31354700 | -1.30199500 |
| H | -4.73573200 | -1.33872500 | -1.64772100 |
| H | -4.73080400 | 0.36233000 | -2.14166900 |
| C | -5.48483700 | 0.02411800 | -0.08448600 |
| H | -6.42844400 | -0.51509900 | -0.10849200 |
| H | -5.69457700 | 1.09363100 | -0.07553800 |
| C | -4.66613600 | -0.34144300 | 1.17276900 |
| H | -4.82238200 | 0.33317400 | 2.01003700 |

| | | | |
|---|---|---|---|
| H | -4.81070500 | -1.37380600 | 1.48947300 |
| C | 0.16988800 | 0.05898900 | 0.01907200 |
| C | 0.93394000 | -1.11339000 | -0.00267800 |
| C | 2.31732000 | -0.96189200 | -0.01194800 |
| H | 2.93851900 | -1.85187300 | -0.02150300 |
| C | 2.92292900 | 0.29575300 | -0.00933700 |
| C | 2.11304400 | 1.42880200 | -0.00434100 |
| H | 2.58012300 | 2.40859400 | -0.00796500 |
| C | 0.72094000 | 1.34183700 | 0.00539100 |
| C | 0.31664700 | -2.49996900 | -0.00917600 |
| H | -0.77006700 | -2.40146200 | -0.06025400 |
| C | -0.12518600 | 2.60187400 | 0.00316400 |
| H | -1.17958600 | 2.31682800 | -0.02672900 |
| C | 4.43267100 | 0.42534800 | -0.01588400 |
| H | 4.66610400 | 1.49435400 | -0.01177600 |
| C | 0.65808100 | -3.25146600 | 1.28310000 |
| H | 1.73631500 | -3.41188500 | 1.36525800 |
| H | 0.32851900 | -2.69452200 | 2.16317100 |
| H | 0.16931400 | -4.22815000 | 1.28848300 |
| C | 0.76009600 | -3.29368500 | -1.24349600 |
| H | 0.51125200 | -2.76077000 | -2.16354500 |
| H | 1.83828000 | -3.47137700 | -1.23087200 |
| H | 0.26004300 | -4.26448000 | -1.26002400 |
| C | 0.10314000 | 3.41283900 | 1.28400000 |
| H | -0.12203700 | 2.81846100 | 2.17239300 |

| | | | |
|---|---|---|---|
| H | 1.14089100 | 3.74915300 | 1.35167700 |
| H | -0.53903200 | 4.29633900 | 1.28726400 |
| C | 0.15545400 | 3.44650500 | -1.24499800 |
| H | 1.19090100 | 3.79575300 | -1.25798800 |
| H | -0.02374600 | 2.87228600 | -2.15647600 |
| H | -0.49530400 | 4.32360000 | -1.25510700 |
| C | 5.03704800 | -0.18903400 | -1.28369100 |
| H | 6.11812000 | -0.03310200 | -1.29887400 |
| H | 4.85248400 | -1.26617500 | -1.32052400 |
| H | 4.61067900 | 0.26081900 | -2.18278500 |
| C | 5.04856200 | -0.20147100 | 1.24023200 |
| H | 6.12971300 | -0.04547300 | 1.24729900 |
| H | 4.63031700 | 0.23919000 | 2.14768200 |
| H | 4.86453700 | -1.27899000 | 1.26787200 |

**7i (B3LYP)**

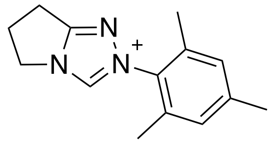

| | | | |
|---|---|---|---|
| N | 0.48241800 | 0.03273600 | 0.05075900 |
| N | 1.21936300 | -0.00626800 | -1.11526200 |
| N | 2.52332700 | 0.07253400 | 0.66533500 |
| C | 1.27191800 | 0.08330000 | 1.12209800 |
| H | 0.94575400 | 0.11882500 | 2.14809200 |
| C | 2.45757000 | 0.02218700 | -0.70161000 |
| C | 3.82265900 | 0.04073700 | -1.30242400 |
| H | 4.02370000 | 1.03174500 | -1.71811700 |
| H | 3.93405700 | -0.68991600 | -2.10221600 |
| C | 4.72052400 | -0.26692700 | -0.06720800 |
| H | 5.66856400 | 0.26551900 | -0.10842300 |
| H | 4.93063900 | -1.33620100 | -0.02687600 |
| C | 3.90504400 | 0.14291000 | 1.18616100 |
| H | 4.02536000 | -0.53462600 | 2.02810000 |
| H | 4.10011500 | 1.16634100 | 1.50708200 |
| C | -0.96284600 | 0.00951100 | 0.02823600 |
| C | -1.64761700 | 1.23111500 | -0.01191700 |
| C | -3.04251700 | 1.17819500 | -0.04133600 |
| H | -3.59774200 | 2.10953800 | -0.07631700 |
| C | -3.73665000 | -0.03485600 | -0.03389300 |
| C | -3.00456200 | -1.22554300 | -0.00665100 |

| | | | |
|---|---|---|---|
| H | -3.53033800 | -2.17445900 | -0.01338100 |
| C | -1.60909200 | -1.23375100 | 0.02346800 |
| C | -0.92373000 | 2.55456200 | -0.02167800 |
| H | -0.42278200 | 2.74360100 | 0.93257500 |
| H | -0.16200400 | 2.59252600 | -0.80421600 |
| H | -1.62699700 | 3.36947700 | -0.19354100 |
| C | -0.84250300 | -2.53254000 | 0.04687200 |
| H | -0.13533200 | -2.59608500 | -0.78412900 |
| H | -0.26957100 | -2.64470800 | 0.97203700 |
| H | -1.52680300 | -3.37790800 | -0.02506700 |
| C | -5.24481900 | -0.05896000 | -0.03260000 |
| H | -5.62860900 | -0.95118400 | -0.53129600 |
| H | -5.62690700 | -0.06675800 | 0.99378800 |
| H | -5.65712900 | 0.82161200 | -0.52903000 |

**M062X**

| | | | |
|---|---|---|---|
| N | 0.47751900 | 0.05676300 | 0.04133900 |
| N | 1.20467300 | -0.14587700 | -1.09743500 |
| N | 2.50629200 | 0.18012600 | 0.64719700 |
| C | 1.25841200 | 0.25632700 | 1.09492500 |
| H | 0.92290900 | 0.43099600 | 2.10504700 |
| C | 2.43978800 | -0.06271800 | -0.69555100 |
| C | 3.80656300 | -0.13302600 | -1.28718100 |
| H | 4.02326000 | 0.80840600 | -1.79685600 |

| | | | |
|---|---|---|---|
| H | 3.90768200 | -0.94714900 | -2.00107500 |
| C | 4.67906500 | -0.31856300 | -0.01934900 |
| H | 5.65928600 | 0.13981700 | -0.12429200 |
| H | 4.81122400 | -1.38275500 | 0.17574900 |
| C | 3.88764900 | 0.31477800 | 1.14544800 |
| H | 3.99954000 | -0.20987500 | 2.09034900 |
| H | 4.10015900 | 1.37526000 | 1.27715400 |
| C | -0.96352800 | 0.02100300 | 0.02419700 |
| C | -1.65391600 | 1.22953000 | -0.06656200 |
| C | -3.04498800 | 1.16154300 | -0.08829800 |
| H | -3.61304200 | 2.08337300 | -0.16035000 |
| C | -3.71970300 | -0.05803300 | -0.02735800 |
| C | -2.97852900 | -1.23831300 | 0.04690900 |
| H | -3.49504600 | -2.19194100 | 0.08413500 |
| C | -1.58663000 | -1.22605500 | 0.07407600 |
| C | -0.92613000 | 2.54518800 | -0.13843400 |
| H | -0.45040300 | 2.78442400 | 0.81613100 |
| H | -0.14537300 | 2.52362200 | -0.90205700 |
| H | -1.62112900 | 3.34872800 | -0.37758900 |
| C | -0.78319400 | -2.49598200 | 0.15140600 |
| H | -0.12890700 | -2.59789800 | -0.71744100 |
| H | -0.15057500 | -2.50719800 | 1.04273400 |
| H | -1.44345700 | -3.36100200 | 0.19022600 |
| C | -5.22463200 | -0.09790700 | -0.01809600 |
| H | -5.59639100 | -1.03384700 | -0.43625400 |

| | | | |
|---|---|---|---|
| H | -5.59701100 | -0.01952800 | 1.00698300 |
| H | -5.64346200 | 0.73286100 | -0.58708700 |

**7j (B3LYP)**

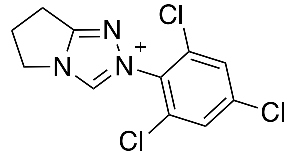


| | | | |
|---|---|---|---|
| N | 0.87186400 | 0.03821800 | 0.05898400 |
| N | 1.59446500 | 0.02737500 | -1.11679400 |
| N | 2.91154900 | 0.06020800 | 0.65822100 |
| C | 1.66987500 | 0.06052200 | 1.12925400 |
| H | 1.35109600 | 0.06965800 | 2.15852400 |
| C | 2.83427800 | 0.04394800 | -0.71186600 |
| C | 4.19365100 | 0.07182200 | -1.32295400 |
| H | 4.39487000 | 1.07318900 | -1.71280300 |
| H | 4.29296000 | -0.63703100 | -2.14348600 |
| C | 5.10015900 | -0.27366400 | -0.10421900 |
| H | 6.05137200 | 0.25308100 | -0.14139600 |
| H | 5.30230400 | -1.34506900 | -0.09271300 |
| C | 4.30044800 | 0.11155900 | 1.16661500 |
| H | 4.42355800 | -0.58541600 | 1.99185300 |
| H | 4.50148300 | 1.12703500 | 1.50777800 |
| C | -0.55505000 | 0.01345800 | 0.04189900 |
| C | -1.28271800 | 1.20897100 | 0.01596700 |
| C | -2.67114500 | 1.19544100 | -0.01477700 |
| H | -3.22658900 | 2.12212000 | -0.03072800 |
| C | -3.32348400 | -0.03294000 | -0.02524600 |
| C | -2.63049000 | -1.23882300 | -0.00743100 |

| | | | |
|---|---|---|---|
| H | -3.15449500 | -2.18375200 | -0.01764400 |
| C | -1.24237000 | -1.20579200 | 0.02412800 |
| Cl | -0.45001100 | 2.73904300 | 0.02446000 |
| Cl | -5.07010000 | -0.06179500 | -0.06159000 |
| Cl | -0.35875000 | -2.70674400 | 0.04475000 |

**M062X**

| | | | |
|---|---|---|---|
| N | 0.86324300 | 0.06437500 | 0.06017700 |
| N | 1.57396400 | 0.05823100 | -1.10716500 |
| N | 2.89188200 | 0.06922300 | 0.65815000 |
| C | 1.65481100 | 0.07295100 | 1.12898000 |
| H | 1.32948600 | 0.07396400 | 2.15777300 |
| C | 2.81119000 | 0.06436300 | -0.70901200 |
| C | 4.17156300 | 0.08630900 | -1.31735000 |
| H | 4.39128600 | 1.09969500 | -1.66033700 |
| H | 4.25637200 | -0.59448000 | -2.16109600 |
| C | 5.05487700 | -0.32124900 | -0.11005400 |
| H | 6.03624300 | 0.14513500 | -0.14468600 |
| H | 5.18310100 | -1.40351700 | -0.10467100 |
| C | 4.28084600 | 0.10364700 | 1.15655500 |
| H | 4.39459900 | -0.58005700 | 1.99312600 |
| H | 4.50204600 | 1.12345700 | 1.46935000 |
| C | -0.55976400 | 0.02672500 | 0.04485100 |
| C | -1.29218000 | 1.21210700 | 0.01784900 |

| | | | |
|---|---|---|---|
| C | -2.67744300 | 1.18217600 | -0.01418200 |
| H | -3.24635800 | 2.10156000 | -0.03044300 |
| C | -3.31092500 | -0.05347600 | -0.02573800 |
| C | -2.60698600 | -1.25066000 | -0.00915500 |
| H | -3.12141700 | -2.20166300 | -0.02205500 |
| C | -1.22257300 | -1.19917700 | 0.02366800 |
| Cl | -0.46476200 | 2.72894600 | 0.02705500 |
| Cl | -5.04329300 | -0.10327900 | -0.06266400 |
| Cl | -0.30661900 | -2.66385600 | 0.04187000 |

**7k (B3LYP)**

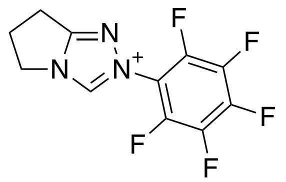


| | | | |
|---|---|---|---|
| N | -0.91321700 | -0.03901700 | 0.06481800 |
| N | -1.63543900 | -0.01031900 | -1.11283600 |
| N | -2.95131800 | -0.07275900 | 0.66400800 |
| C | -1.71243000 | -0.07907600 | 1.13650800 |
| H | -1.39310400 | -0.10429900 | 2.16558000 |
| C | -2.87413000 | -0.03456500 | -0.70710500 |
| C | -4.23358300 | -0.05371900 | -1.31780400 |
| H | -4.43295000 | -1.04888600 | -1.72408400 |
| H | -4.33323500 | 0.66842100 | -2.12661600 |
| C | -5.14058500 | 0.27062000 | -0.09363400 |
| H | -6.09066300 | -0.25742500 | -0.13965500 |
| H | -5.34466300 | 1.34125800 | -0.06453500 |
| C | -4.34113800 | -0.13406900 | 1.17097300 |
| H | -4.46442200 | 0.54894600 | 2.00768300 |
| H | -4.53933300 | -1.15564600 | 1.49500700 |
| C | 0.50873700 | -0.01504700 | 0.04838100 |
| C | 1.19240400 | 1.19959500 | 0.03437300 |
| C | 2.57902800 | 1.22605300 | -0.00154100 |
| C | 3.28869200 | 0.03064600 | -0.02921500 |
| C | 2.61849800 | -1.18757200 | -0.02050300 |

| | | | |
|---|---|---|---|
| C | 1.23171700 | -1.20654600 | 0.01516600 |
| F | 4.61734900 | 0.05290100 | -0.06183900 |
| F | 3.23095600 | 2.38875100 | -0.00912600 |
| F | 0.51677300 | 2.34699900 | 0.05994300 |
| F | 0.59335700 | -2.37542200 | 0.02077200 |
| F | 3.30766700 | -2.32829700 | -0.04740600 |

**M062X**

| | | | |
|---|---|---|---|
| N | -0.90998000 | -0.04560900 | 0.06229000 |
| N | -1.62353400 | -0.01724900 | -1.10512500 |
| N | -2.93668400 | -0.07752900 | 0.66347500 |
| C | -1.70170300 | -0.08443700 | 1.13343600 |
| H | -1.37518800 | -0.10796600 | 2.16157100 |
| C | -2.85894600 | -0.04062000 | -0.70447200 |
| C | -4.22000000 | -0.06304600 | -1.31094700 |
| H | -4.42499800 | -1.06758900 | -1.68675900 |
| H | -4.31507400 | 0.64339500 | -2.13230200 |
| C | -5.10862800 | 0.29574500 | -0.09188600 |
| H | -6.07913000 | -0.19186300 | -0.13612300 |
| H | -5.26160500 | 1.37417700 | -0.06077000 |
| C | -4.32420000 | -0.14163100 | 1.16427400 |
| H | -4.44447800 | 0.52444800 | 2.01402000 |
| H | -4.52782700 | -1.17048700 | 1.45834500 |
| C | 0.50941100 | -0.01894500 | 0.04706600 |

| | | | |
|---|---|---|---|
| C | 1.18128100 | 1.19598500 | 0.03236000 |
| C | 2.56398500 | 1.22720000 | -0.00082200 |
| C | 3.27365600 | 0.03602500 | -0.02734400 |
| C | 2.61142300 | -1.18231000 | -0.01973000 |
| C | 1.22846000 | -1.20656800 | 0.01344900 |
| F | 4.59302500 | 0.06216100 | -0.05925100 |
| F | 3.20877500 | 2.38306100 | -0.00830000 |
| F | 0.50134700 | 2.32938900 | 0.05553200 |
| F | 0.59400000 | -2.36619900 | 0.01714400 |
| F | 3.30188100 | -2.31127800 | -0.04676500 |

**8a (B3LYP)**

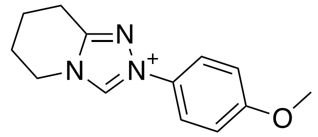

| | | | |
|---|---|---|---|
| N | 0.43202900 | -0.00941200 | -0.03428800 |
| N | 1.20274900 | 1.05127400 | 0.35848000 |
| N | 2.46373300 | -0.67103600 | -0.20288900 |
| C | 1.18952400 | -1.04144800 | -0.38204800 |
| H | 0.84697900 | -1.98963600 | -0.76004900 |
| C | 2.43891600 | 0.62964900 | 0.24657500 |
| C | -0.99804000 | 0.07458100 | -0.03916100 |
| C | -1.75657600 | -1.00722100 | 0.39134000 |
| C | -3.14744100 | -0.93122200 | 0.36408600 |
| C | -3.77082300 | 0.24050200 | -0.08008400 |
| C | -2.98980900 | 1.33027300 | -0.49800600 |
| C | -1.60874600 | 1.24966100 | -0.48281600 |
| C | 3.68842500 | -1.45281900 | -0.50834400 |
| H | 3.80060400 | -1.46293600 | -1.59385300 |
| H | 3.52724000 | -2.47203700 | -0.16070000 |
| C | 3.68386300 | 1.38358700 | 0.57393800 |
| H | 3.56555000 | 2.41847300 | 0.24887500 |
| H | 3.79007900 | 1.40056100 | 1.66381800 |
| C | 4.91311300 | 0.71641000 | -0.06608700 |
| H | 4.93333600 | 0.92751400 | -1.14010600 |
| H | 5.81666000 | 1.15119100 | 0.36349200 |

| | | | |
|---|---|---|---|
| C | 4.89350800 | -0.79971400 | 0.16626400 |
| H | 4.87706600 | -1.01390100 | 1.23942500 |
| H | 5.79279300 | -1.26440700 | -0.24211900 |
| H | -1.27911800 | -1.90493300 | 0.76464600 |
| H | -3.72425700 | -1.77895500 | 0.70503400 |
| H | -3.48576000 | 2.22997200 | -0.84021000 |
| H | -1.00575800 | 2.08447700 | -0.81513200 |
| O | -5.11280800 | 0.41966800 | -0.13805400 |
| C | -5.97314000 | -0.65175200 | 0.26858500 |
| H | -6.98645600 | -0.28217800 | 0.12951800 |
| H | -5.81694000 | -1.53680700 | -0.35363300 |
| H | -5.81577300 | -0.90243600 | 1.32085100 |

**M062X**

| | | | |
|---|---|---|---|
| N | 0.43251800 | -0.01093400 | -0.02242200 |
| N | 1.19505500 | 1.04628700 | 0.35356100 |
| N | 2.45199000 | -0.67179500 | -0.19612200 |
| C | 1.18228100 | -1.04387500 | -0.36380300 |
| H | 0.83384700 | -1.99626400 | -0.73015900 |
| C | 2.42745400 | 0.62863200 | 0.24118600 |
| C | -0.99626200 | 0.07369300 | -0.03086100 |
| C | -1.75124600 | -1.01360300 | 0.37428000 |
| C | -3.14022700 | -0.93545600 | 0.34151200 |
| C | -3.75469000 | 0.24461400 | -0.08042600 |

| | | | |
|---|---|---|---|
| C | -2.97355400 | 1.34078400 | -0.47110600 |
| C | -1.59579500 | 1.25804600 | -0.45277300 |
| C | 3.67082800 | -1.45557300 | -0.49368800 |
| H | 3.78708900 | -1.46904900 | -1.57814100 |
| H | 3.50581500 | -2.47018100 | -0.13605600 |
| C | 3.67185300 | 1.38737900 | 0.55276600 |
| H | 3.54475000 | 2.41708500 | 0.21847300 |
| H | 3.78979400 | 1.40508000 | 1.64008900 |
| C | 4.88340100 | 0.71123000 | -0.09781700 |
| H | 4.87380500 | 0.88866200 | -1.17727900 |
| H | 5.79493600 | 1.15744400 | 0.29864200 |
| C | 4.86524200 | -0.79279400 | 0.17952800 |
| H | 4.82853200 | -0.97376200 | 1.25756200 |
| H | 5.76777400 | -1.26886500 | -0.20432700 |
| H | -1.27323700 | -1.91748100 | 0.73267600 |
| H | -3.72026100 | -1.78993500 | 0.66005600 |
| H | -3.47144400 | 2.24633400 | -0.79414100 |
| H | -0.98413200 | 2.09593100 | -0.76221800 |
| O | -5.09066100 | 0.42401800 | -0.14101400 |
| C | -5.92931100 | -0.65540400 | 0.25320200 |
| H | -6.94887700 | -0.30148100 | 0.12714200 |
| H | -5.76188500 | -1.52911600 | -0.38144200 |
| H | -5.75801600 | -0.91749100 | 1.30011500 |

**8b (B3LYP)**

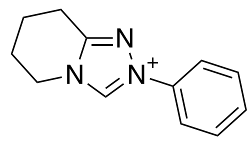


| | | | |
|---|---|---|---|
| N | -0.41365400 | -0.05554900 | -0.00990800 |
| N | 0.32623900 | 1.04474100 | 0.32695000 |
| N | 1.63456700 | -0.65615600 | -0.19043600 |
| C | 0.37222900 | -1.07585000 | -0.32981200 |
| H | 0.05560800 | -2.04950600 | -0.66343800 |
| C | 1.57361900 | 0.66003700 | 0.21117400 |
| C | -1.84820100 | -0.01893700 | 0.00246800 |
| C | -2.55613900 | -1.12904400 | 0.45737300 |
| C | -3.94842000 | -1.08760200 | 0.44254500 |
| C | -4.61305200 | 0.05411700 | -0.00335100 |
| C | -3.88539300 | 1.16132000 | -0.44105300 |
| C | -2.49378300 | 1.13128000 | -0.44525000 |
| C | 2.88047800 | -1.40788300 | -0.48674200 |
| H | 2.98235300 | -1.44459200 | -1.57268500 |
| H | 2.75339900 | -2.42125800 | -0.10935100 |
| C | 2.79782100 | 1.46272000 | 0.49819300 |
| H | 2.64600100 | 2.48078900 | 0.13614300 |
| H | 2.91047200 | 1.52341700 | 1.58591600 |
| C | 4.04278200 | 0.80955500 | -0.12604700 |
| H | 4.05181600 | 0.98418100 | -1.20655300 |
| H | 4.93501600 | 1.28512700 | 0.28338200 |

| | | | |
|---|---|---|---|
| C | 4.06954800 | -0.69781200 | 0.15738600 |
| H | 4.06588100 | -0.87662200 | 1.23704000 |
| H | 4.97969400 | -1.14897700 | -0.24196200 |
| H | -2.03712000 | -2.00134000 | 0.83505200 |
| H | -4.50971100 | -1.94425900 | 0.79489400 |
| H | -4.39964800 | 2.04932000 | -0.78782600 |
| H | -1.91671900 | 1.97799500 | -0.79259700 |
| H | -5.69604600 | 0.08308600 | -0.00554500 |

**M062X**

| | | | |
|---|---|---|---|
| N | -0.40785000 | -0.05889900 | -0.00079400 |
| N | 0.32272600 | 1.03817100 | 0.31918700 |
| N | 1.62944000 | -0.65841700 | -0.17989000 |
| C | 0.37218900 | -1.08018500 | -0.31052100 |
| H | 0.05207700 | -2.05848200 | -0.63185900 |
| C | 1.56663400 | 0.65812900 | 0.20586700 |
| C | -1.84067200 | -0.02151500 | 0.00601500 |
| C | -2.54687900 | -1.14170500 | 0.42391500 |
| C | -3.93632300 | -1.09531200 | 0.40319900 |
| C | -4.59313600 | 0.05970600 | -0.01015900 |
| C | -3.86283600 | 1.17631100 | -0.40967300 |
| C | -2.47407100 | 1.14256900 | -0.40910200 |
| C | 2.87097900 | -1.41206200 | -0.46230700 |
| H | 2.97756400 | -1.46006400 | -1.54680500 |

| | | | |
|---|---|---|---|
| H | 2.74165800 | -2.41790900 | -0.06743000 |
| C | 2.78853900 | 1.46824700 | 0.47369200 |
| H | 2.62570800 | 2.47897100 | 0.09940300 |
| H | 2.91434500 | 1.53365100 | 1.55840500 |
| C | 4.01612000 | 0.80540500 | -0.16019700 |
| H | 3.99319500 | 0.93948400 | -1.24560900 |
| H | 4.91561100 | 1.29563200 | 0.21081500 |
| C | 4.04790700 | -0.68640300 | 0.17552600 |
| H | 4.02388000 | -0.82649300 | 1.25996200 |
| H | 4.96261800 | -1.14804100 | -0.19698700 |
| H | -2.02875200 | -2.02443000 | 0.77852500 |
| H | -4.50242500 | -1.96033700 | 0.72511100 |
| H | -4.37386200 | 2.07566700 | -0.72978200 |
| H | -1.88696200 | 1.99543500 | -0.72448700 |
| H | -5.67556200 | 0.09183500 | -0.01632000 |

**8c (B3LYP)**

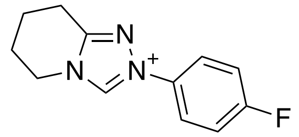


| | | | |
|---|---|---|---|
| N | 0.00026000 | 0.06341700 | -0.01339300 |
| N | -0.73400400 | -1.03661700 | 0.33705800 |
| N | -2.05025600 | 0.65259300 | -0.19885900 |
| C | -0.79032900 | 1.07646800 | -0.34504500 |
| H | -0.47817200 | 2.04759400 | -0.69042400 |
| C | -1.98321000 | -0.65886500 | 0.21741200 |
| C | 1.43298000 | 0.03250000 | 0.00058700 |
| C | 2.13825100 | 1.13669300 | 0.47397200 |
| C | 3.52933200 | 1.10870000 | 0.46457300 |
| C | 4.16487800 | -0.03187700 | -0.00040600 |
| C | 3.47426400 | -1.14397200 | -0.45928800 |
| C | 2.08461400 | -1.10860300 | -0.46279900 |
| C | -3.30005600 | 1.39635400 | -0.49992600 |
| H | -3.40405200 | 1.42205100 | -1.58594500 |
| H | -3.17619500 | 2.41378800 | -0.13253100 |
| C | -3.20350300 | -1.46410600 | 0.51351700 |
| H | -3.04755700 | -2.48483000 | 0.16080800 |
| H | -3.31423000 | -1.51507400 | 1.60192700 |
| C | -4.45216600 | -0.82230000 | -0.11511200 |
| H | -4.46232000 | -1.00775900 | -1.19381200 |
| H | -5.34164000 | -1.29754100 | 0.30063000 |

| | | | |
|---|---|---|---|
| C | -4.48477700 | 0.68761400 | 0.15351000 |
| H | -4.47984800 | 0.87721500 | 1.23132800 |
| H | -5.39752900 | 1.13101300 | -0.24855900 |
| F | 5.51915000 | -0.06431300 | -0.00167700 |
| H | 1.61899300 | 2.00320900 | 0.86335600 |
| H | 4.10864300 | 1.94780300 | 0.82666400 |
| H | 4.01372200 | -2.01261700 | -0.81348100 |
| H | 1.51417900 | -1.95342300 | -0.82448800 |

**M062X**

| | | | |
|---|---|---|---|
| N | -0.00331800 | 0.06694900 | -0.00366100 |
| N | -0.72818400 | -1.02973100 | 0.33085100 |
| N | -2.04291700 | 0.65430300 | -0.18988100 |
| C | -0.78801000 | 1.08043000 | -0.32707800 |
| H | -0.47213600 | 2.05591900 | -0.66128600 |
| C | -1.97392700 | -0.65701300 | 0.21248500 |
| C | 1.42776500 | 0.03587200 | 0.00578200 |
| C | 2.13033500 | 1.14987300 | 0.44607800 |
| C | 3.51841900 | 1.11818900 | 0.43103900 |
| C | 4.14738200 | -0.03476300 | -0.00410900 |
| C | 3.45464900 | -1.15634100 | -0.42849600 |
| C | 2.06813200 | -1.11804000 | -0.42838200 |
| C | -3.28830700 | 1.39919500 | -0.47953800 |
| H | -3.39656300 | 1.43345000 | -1.56433600 |

| | | | |
|---|---|---|---|
| H | -3.16248200 | 2.41025400 | -0.09708100 |
| C | -3.19191700 | -1.46935000 | 0.49110500 |
| H | -3.02498000 | -2.48366800 | 0.12853400 |
| H | -3.31572500 | -1.52255000 | 1.57670100 |
| C | -4.42340900 | -0.81937700 | -0.14853700 |
| H | -4.40224900 | -0.96684300 | -1.23223000 |
| H | -5.32001700 | -1.30876400 | 0.23043900 |
| C | -4.46102200 | 0.67625500 | 0.16900000 |
| H | -4.43609700 | 0.82994800 | 1.25156900 |
| H | -5.37823900 | 1.12934700 | -0.20782000 |
| F | 5.48996300 | -0.07037200 | -0.00932300 |
| H | 1.61067100 | 2.02546700 | 0.81503900 |
| H | 4.10529000 | 1.96360700 | 0.76512200 |
| H | 3.99538600 | -2.03410000 | -0.75737300 |
| H | 1.48787200 | -1.96894300 | -0.76063800 |

**8i (B3LYP)**

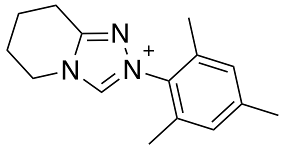

| | | | |
|---|---|---|---|
| N | 0.18034000 | 0.01431800 | 0.05749400 |
| N | 0.91853600 | -0.20506500 | -1.07734000 |
| N | 2.23200800 | 0.13575100 | 0.66502900 |
| C | 0.97014800 | 0.21587900 | 1.10136500 |
| H | 0.64990200 | 0.40291600 | 2.11315600 |
| C | 2.16682900 | -0.12736700 | -0.68679800 |
| C | -1.26553400 | 0.00830800 | 0.03183300 |
| C | -1.93545900 | 1.22940300 | -0.11102600 |
| C | -3.33155400 | 1.18951400 | -0.14171000 |
| C | -4.03908500 | -0.01066800 | -0.03931700 |
| C | -3.32020100 | -1.20348500 | 0.08934900 |
| C | -1.92564300 | -1.22444600 | 0.12746100 |
| C | 3.48197900 | 0.23238300 | 1.46180300 |
| H | 3.58999700 | -0.70819500 | 2.00450700 |
| H | 3.35716300 | 1.04211300 | 2.17901100 |
| C | 3.38937400 | -0.25691100 | -1.53303500 |
| H | 3.23503200 | -1.05923500 | -2.25630100 |
| H | 3.50076300 | 0.67110200 | -2.10372100 |
| C | 4.63714500 | -0.49554100 | -0.66587900 |
| H | 4.64852200 | -1.52933500 | -0.30623600 |
| H | 5.52760800 | -0.36011300 | -1.28118900 |

| | | | |
|---|---|---|---|
| C | 4.66546100 | 0.46918500 | 0.52574600 |
| H | 4.65449100 | 1.50425000 | 0.17016600 |
| H | 5.57928400 | 0.33658600 | 1.10766100 |
| C | -1.17296100 | -2.52440100 | 0.26339600 |
| H | -0.51072400 | -2.69396700 | -0.58966700 |
| H | -0.55291200 | -2.53815900 | 1.16437500 |
| H | -1.86908600 | -3.36094200 | 0.32368100 |
| C | -1.19776500 | 2.54048300 | -0.22499700 |
| H | -0.71183500 | 2.80923400 | 0.71784200 |
| H | -0.42256500 | 2.50383200 | -0.99435500 |
| H | -1.89021900 | 3.34276500 | -0.48002900 |
| C | -5.54747400 | -0.02162900 | -0.04489600 |
| H | -5.93585600 | -0.89734700 | -0.56941500 |
| H | -5.93465100 | -0.05651100 | 0.97891900 |
| H | -5.95043700 | 0.87460500 | -0.51986300 |
| H | -3.85649000 | -2.14397400 | 0.16008700 |
| H | -3.87573000 | 2.12109800 | -0.25467800 |

**M062X**

| | | | |
|---|---|---|---|
| N | 0.17755000 | 0.00954000 | 0.06042600 |
| N | 0.90407200 | -0.21754400 | -1.06556700 |
| N | 2.21859800 | 0.13642300 | 0.66297700 |
| C | 0.96212400 | 0.22019800 | 1.10014300 |
| H | 0.63771100 | 0.41818000 | 2.11027200 |
| C | 2.14982000 | -0.13604800 | -0.68231800 |

| | | | |
|---|---|---|---|
| C | -1.26419600 | 0.00755200 | 0.03676700 |
| C | -1.92191300 | 1.22686800 | -0.11212600 |
| C | -3.31517700 | 1.19314100 | -0.14499400 |
| C | -4.02040600 | -0.00469100 | -0.03966500 |
| C | -3.30944500 | -1.19940200 | 0.09443200 |
| C | -1.91898800 | -1.22126000 | 0.13469700 |
| C | 3.46560400 | 0.24464700 | 1.45257000 |
| H | 3.58706800 | -0.69677300 | 1.98999900 |
| H | 3.33468600 | 1.05416200 | 2.16809000 |
| C | 3.36908400 | -0.27200500 | -1.52981500 |
| H | 3.20868100 | -1.07709900 | -2.24687000 |
| H | 3.48366900 | 0.65659800 | -2.09660100 |
| C | 4.60428500 | -0.50840300 | -0.65452900 |
| H | 4.59344900 | -1.52961900 | -0.26232400 |
| H | 5.49930500 | -0.40332000 | -1.26700400 |
| C | 4.63140200 | 0.48848400 | 0.50443300 |
| H | 4.58662300 | 1.51139100 | 0.11949900 |
| H | 5.55286100 | 0.39164500 | 1.07892000 |
| C | -1.14782400 | -2.50567800 | 0.27473500 |
| H | -0.47476200 | -2.65290000 | -0.57301900 |
| H | -0.53812300 | -2.50139700 | 1.18186500 |
| H | -1.82938400 | -3.35336000 | 0.32613000 |
| C | -1.15940700 | 2.51902700 | -0.23175500 |
| H | -0.67820300 | 2.77991700 | 0.71438900 |
| H | -0.37868400 | 2.44752600 | -0.99243000 |

| | | | |
|---|---|---|---|
| H | -1.83189000 | 3.33161800 | -0.50242700 |
| C | -5.52588300 | -0.01729900 | -0.05405400 |
| H | -5.90267600 | -0.84673500 | -0.65456400 |
| H | -5.91338600 | -0.14241900 | 0.96046200 |
| H | -5.92451800 | 0.91497300 | -0.45443900 |
| H | -3.85069200 | -2.13738700 | 0.16676300 |
| H | -3.85775700 | 2.12527500 | -0.26268900 |

**8k (B3LYP)**

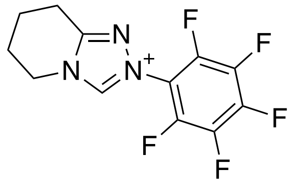


| | | | |
|---|---|---|---|
| N | -0.62053500 | -0.00957700 | 0.07415500 |
| N | -1.34547600 | 0.21755200 | -1.06959500 |
| N | -2.66872200 | -0.13728300 | 0.66715100 |
| C | -1.41814900 | -0.22017400 | 1.11709100 |
| H | -1.10234300 | -0.41346800 | 2.12973400 |
| C | -2.59380300 | 0.13568400 | -0.68675300 |
| C | 0.80165800 | -0.00688100 | 0.05251000 |
| C | 1.50751300 | -1.20325500 | -0.05875000 |
| C | 2.89451500 | -1.20119200 | -0.09666600 |
| C | 3.58139700 | 0.00581000 | -0.03363900 |
| C | 2.88816800 | 1.20671200 | 0.06990800 |
| C | 1.50152900 | 1.19676100 | 0.11179100 |
| C | -3.92980700 | -0.24089000 | 1.45133000 |
| H | -4.04266100 | 0.69894900 | 1.99377700 |
| H | -3.80811200 | -1.05243400 | 2.16666300 |
| C | -3.80925600 | 0.26705700 | -1.54127600 |
| H | -3.64984000 | 1.07387000 | -2.25821000 |
| H | -3.90942800 | -0.65864500 | -2.11769400 |
| C | -5.06568300 | 0.49490400 | -0.68396200 |
| H | -5.08570200 | 1.52655200 | -0.31920400 |

| | | | |
|---|---|---|---|
| H | -5.94854900 | 0.35815500 | -1.30950800 |
| C | -5.10118200 | -0.47751200 | 0.50094500 |
| H | -5.08258600 | -1.51052500 | 0.14027200 |
| H | -6.02056800 | -0.35156500 | 1.07533300 |
| F | 4.91026800 | 0.01187900 | -0.07062000 |
| F | 3.56756300 | -2.34739400 | -0.19739300 |
| F | 0.85282700 | -2.36133600 | -0.12747700 |
| F | 0.84126100 | 2.34842100 | 0.21660100 |
| F | 3.55602700 | 2.35866700 | 0.13240900 |

**M062X**

| | | | |
|---|---|---|---|
| N | -0.61954700 | -0.03231800 | 0.06750400 |
| N | -1.33541200 | 0.58032200 | -0.91499100 |
| N | -2.65669200 | -0.37017300 | 0.56944900 |
| C | -1.41179200 | -0.60275600 | 0.96369700 |
| H | -1.09801600 | -1.14314200 | 1.84374600 |
| C | -2.58036100 | 0.36610400 | -0.59367500 |
| C | 0.79861300 | -0.02100200 | 0.04893400 |
| C | 1.50855200 | -1.19742900 | -0.14949300 |
| C | 2.89179600 | -1.18035300 | -0.18422900 |
| C | 3.56464800 | 0.02158300 | -0.03137300 |
| C | 2.86381700 | 1.20281900 | 0.16058100 |
| C | 1.48163000 | 1.17859100 | 0.20267900 |
| C | -3.91099100 | -0.75005500 | 1.26234500 |

| | | | |
|---|---|---|---|
| H | -4.03018600 | -0.06127400 | 2.09936400 |
| H | -3.78248100 | -1.76258500 | 1.63984600 |
| C | -3.79469700 | 0.78896800 | -1.34659400 |
| H | -3.63044100 | 1.79318500 | -1.73708300 |
| H | -3.90242900 | 0.11497700 | -2.20150400 |
| C | -5.03470000 | 0.70612500 | -0.45029100 |
| H | -5.02683700 | 1.52588200 | 0.27375900 |
| H | -5.92485900 | 0.82341000 | -1.06743900 |
| C | -5.07156200 | -0.63457400 | 0.28411000 |
| H | -5.03208000 | -1.45768800 | -0.43478000 |
| H | -5.99460100 | -0.73861700 | 0.85474300 |
| F | 4.88431100 | 0.04241000 | -0.06759200 |
| F | 3.57076100 | -2.30116500 | -0.37082200 |
| F | 0.86501600 | -2.34274200 | -0.30928900 |
| F | 0.81353700 | 2.30218400 | 0.39969900 |
| F | 3.51901600 | 2.34296900 | 0.31194400 |

**9a (B3LYP)**

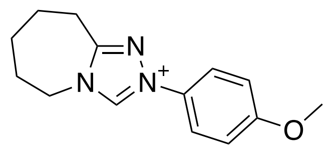


| | | | |
|---|---|---|---|
| N | -0.12376500 | 0.07485700 | -0.17074700 |
| N | -0.89371900 | -0.98040900 | -0.57814100 |
| N | -2.14669000 | 0.79063900 | -0.17169400 |
| C | -0.87867600 | 1.13493600 | 0.08327800 |
| H | -0.53952500 | 2.09010800 | 0.44700200 |
| C | -2.12491800 | -0.52880700 | -0.57381500 |
| C | 1.29974100 | -0.04186700 | -0.05593500 |
| C | 2.11646700 | 0.98402000 | -0.51530300 |
| C | 3.49952300 | 0.87881800 | -0.38353500 |
| C | 4.05716000 | -0.26762100 | 0.19388700 |
| C | 3.21854100 | -1.30204900 | 0.64016000 |
| C | 1.84452800 | -1.19118300 | 0.52088000 |
| C | -3.33459300 | 1.66269600 | -0.02508400 |
| H | -3.75578700 | 1.80882200 | -1.02181500 |
| H | -2.96079700 | 2.62326300 | 0.32499200 |
| C | -3.33105900 | -1.32152000 | -0.93832600 |
| H | -3.77413600 | -0.89425200 | -1.84522800 |
| H | -2.97947300 | -2.32130200 | -1.19569000 |
| C | -4.39408600 | -1.39869100 | 0.17782100 |
| H | -3.92020200 | -1.73207500 | 1.10711000 |
| H | -5.10052800 | -2.18172500 | -0.10895400 |

| | | | |
|---|---|---|---|
| C | -4.37002100 | 1.09311700 | 0.94447600 |
| H | -3.87673400 | 0.84131900 | 1.88911800 |
| H | -5.06173700 | 1.91162100 | 1.16273200 |
| C | -5.17932600 | -0.10161400 | 0.41782200 |
| H | -5.97245700 | -0.31322500 | 1.14063400 |
| H | -5.68239300 | 0.19092200 | -0.51181700 |
| H | 1.19651100 | -1.98266500 | 0.87413500 |
| H | 3.66411200 | -2.18241900 | 1.08621700 |
| H | 4.12204800 | 1.68328300 | -0.74807100 |
| H | 1.69105400 | 1.85916100 | -0.99106700 |
| O | 5.38645000 | -0.47243600 | 0.36034700 |
| C | 6.30313300 | 0.53998400 | -0.07345700 |
| H | 7.29362400 | 0.15925600 | 0.16468600 |
| H | 6.22311700 | 0.70453100 | -1.15115800 |
| H | 6.13244200 | 1.47793200 | 0.46125100 |

**M062X**

| | | | |
|---|---|---|---|
| N | -0.12975000 | 0.06872000 | -0.20612200 |
| N | -0.89738800 | -0.97916000 | -0.59792900 |
| N | -2.13571400 | 0.79470800 | -0.20664500 |
| C | -0.87060700 | 1.13423400 | 0.04015300 |
| H | -0.51873100 | 2.09052200 | 0.39321700 |
| C | -2.12188000 | -0.52321200 | -0.59443400 |
| C | 1.29104500 | -0.05147400 | -0.08489300 |

| | | | |
|---|---|---|---|
| C | 2.10286800 | 0.98971300 | -0.50090200 |
| C | 3.48262500 | 0.88614800 | -0.35303100 |
| C | 4.03170100 | -0.27466400 | 0.19416900 |
| C | 3.19476300 | -1.32572500 | 0.59314600 |
| C | 1.82507000 | -1.21639700 | 0.46068100 |
| C | -3.32382100 | 1.65412500 | -0.05428100 |
| H | -3.78193400 | 1.75304600 | -1.04052400 |
| H | -2.95172700 | 2.63105200 | 0.24879200 |
| C | -3.34031800 | -1.30405200 | -0.93329400 |
| H | -3.81904800 | -0.85143600 | -1.80805000 |
| H | -3.00000000 | -2.29787200 | -1.22359900 |
| C | -4.34101200 | -1.39024600 | 0.23095100 |
| H | -3.81311000 | -1.69760700 | 1.13919600 |
| H | -5.04802600 | -2.18662300 | -0.00880200 |
| C | -4.30453600 | 1.09455000 | 0.96988000 |
| H | -3.75881100 | 0.84625600 | 1.88548200 |
| H | -4.98758400 | 1.91001300 | 1.21796200 |
| C | -5.12690500 | -0.10250800 | 0.48605100 |
| H | -5.89089800 | -0.31095200 | 1.23858500 |
| H | -5.66008400 | 0.17923400 | -0.42907100 |
| H | 1.17005100 | -2.01777000 | 0.77811400 |
| H | 3.64223200 | -2.21667200 | 1.01519500 |
| H | 4.10703100 | 1.70497800 | -0.68082800 |
| H | 1.67554000 | 1.87662600 | -0.95353600 |
| O | 5.35372600 | -0.47636000 | 0.37387800 |

| | | | |
|---|---|---|---|
| C | 6.24778300 | 0.55953500 | -0.01523400 |
| H | 7.24481000 | 0.19588700 | 0.21831700 |
| H | 6.17034800 | 0.75685300 | -1.08721200 |
| H | 6.04945500 | 1.47530600 | 0.54684700 |

**9b (B3LYP)**

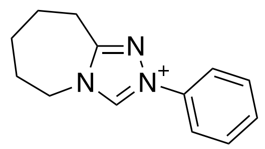


| | | | |
|---|---|---|---|
| N | -0.70596300 | -0.02346800 | -0.17686600 |
| N | 0.02942100 | 1.11237000 | 0.02189400 |
| N | 1.33478600 | -0.57796800 | -0.53690100 |
| C | 0.07900900 | -1.03752900 | -0.51652900 |
| H | -0.23565600 | -2.04054600 | -0.74971900 |
| C | 1.27165500 | 0.76076000 | -0.20481700 |
| C | -2.13267000 | -0.02298800 | -0.01876300 |
| C | -2.85649400 | 1.07746000 | -0.47215900 |
| C | -4.24083500 | 1.07338900 | -0.32647300 |
| C | -4.88511900 | -0.01880800 | 0.25569800 |
| C | -4.14301700 | -1.11041600 | 0.70465000 |
| C | -2.75568600 | -1.11616100 | 0.57859400 |
| C | 2.54835100 | -1.36082100 | -0.86979500 |
| H | 2.95435300 | -0.95027200 | -1.79679900 |
| H | 2.20787400 | -2.37493500 | -1.07171200 |
| C | 2.45191700 | 1.66126100 | -0.09845900 |
| H | 2.89652200 | 1.77929900 | -1.09332200 |
| H | 2.07100500 | 2.63900200 | 0.19860200 |
| C | 3.52270600 | 1.17614300 | 0.90031500 |
| H | 3.04857400 | 0.95809100 | 1.86313800 |
| H | 4.20189100 | 2.01504100 | 1.07182900 |

| | | | |
|---|---|---|---|
| C | 3.58614500 | -1.34816200 | 0.25340500 |
| H | 3.10412400 | -1.64557400 | 1.19053100 |
| H | 4.30427900 | -2.13586100 | 0.00899300 |
| C | 4.35060500 | -0.02807800 | 0.43021700 |
| H | 5.14763600 | -0.19770300 | 1.15974300 |
| H | 4.84837300 | 0.22489600 | -0.51373800 |
| H | -2.17440800 | -1.94780400 | 0.95684400 |
| H | -4.63824800 | -1.95465600 | 1.16841300 |
| H | -4.81537100 | 1.92250600 | -0.67604000 |
| H | -2.34489500 | 1.91253800 | -0.93208200 |
| H | -5.96316300 | -0.01684700 | 0.36312200 |

**M062X**

| | | | |
|---|---|---|---|
| N | -0.69378000 | -0.01598800 | -0.19503800 |
| N | 0.03253000 | 1.11446500 | -0.00735300 |
| N | 1.33302400 | -0.56832700 | -0.56514300 |
| C | 0.08375800 | -1.02939700 | -0.53499100 |
| H | -0.23525300 | -2.03417600 | -0.76249600 |
| C | 1.26966700 | 0.76557400 | -0.23754500 |
| C | -2.11704900 | -0.02073400 | -0.02531000 |
| C | -2.83875200 | 1.09057200 | -0.44086300 |
| C | -4.21881800 | 1.08115400 | -0.28018100 |
| C | -4.85433400 | -0.02620600 | 0.27612300 |
| C | -4.11004600 | -1.12817100 | 0.68605300 |

| | | | |
|---|---|---|---|
| C | -2.72674300 | -1.12960200 | 0.54634400 |
| C | 2.55098600 | -1.33923200 | -0.87990800 |
| H | 2.99025300 | -0.89826000 | -1.77723400 |
| H | 2.21727600 | -2.34683500 | -1.12146300 |
| C | 2.45770800 | 1.65133600 | -0.12816600 |
| H | 2.93701100 | 1.72418000 | -1.11024200 |
| H | 2.08278500 | 2.64210100 | 0.12801400 |
| C | 3.47030200 | 1.16435500 | 0.92077300 |
| H | 2.94497000 | 0.94186800 | 1.85488100 |
| H | 4.14557100 | 1.99646200 | 1.12861000 |
| C | 3.53542400 | -1.34762300 | 0.28455300 |
| H | 3.00196800 | -1.62632800 | 1.19863500 |
| H | 4.25032000 | -2.14697800 | 0.07714600 |
| C | 4.30583300 | -0.04038700 | 0.48328900 |
| H | 5.07493600 | -0.21414900 | 1.23939300 |
| H | 4.83262400 | 0.20700300 | -0.44528500 |
| H | -2.13681800 | -1.96885100 | 0.89438800 |
| H | -4.60050100 | -1.98459100 | 1.13118300 |
| H | -4.79796600 | 1.93940600 | -0.59733600 |
| H | -2.32652600 | 1.93584700 | -0.88208600 |
| H | -5.93069400 | -0.02759000 | 0.39559800 |

**9c (B3LYP)**

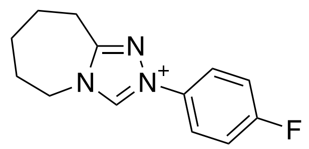


| | | | |
|---|---|---|---|
| N | -0.29922000 | -0.02198900 | -0.20997700 |
| N | 0.43219000 | 1.11205100 | 0.01425700 |
| N | 1.74626900 | -0.57059400 | -0.54816400 |
| C | 0.49103200 | -1.03132000 | -0.55191500 |
| H | 0.18016100 | -2.03131300 | -0.80297400 |
| C | 1.67763700 | 0.76393400 | -0.19997200 |
| C | -1.72602900 | -0.02281300 | -0.07065400 |
| C | -2.44809200 | 1.06368900 | -0.56044200 |
| C | -3.83257700 | 1.06951300 | -0.43516300 |
| C | -4.44848800 | -0.01713600 | 0.16824100 |
| C | -3.74256100 | -1.10334500 | 0.66084100 |
| C | -2.35567300 | -1.10059800 | 0.54754600 |
| C | 2.96543300 | -1.34839600 | -0.87345300 |
| H | 3.38518200 | -0.92442400 | -1.78821500 |
| H | 2.62841600 | -2.35959100 | -1.09495200 |
| C | 2.85615400 | 1.66251100 | -0.06305500 |
| H | 3.31753600 | 1.79238200 | -1.04867000 |
| H | 2.47072300 | 2.63667100 | 0.23982000 |
| C | 3.90936400 | 1.16340200 | 0.94759000 |
| H | 3.41841700 | 0.93179300 | 1.89874100 |
| H | 4.58514400 | 1.99989000 | 1.14263500 |

| | | | |
|---|---|---|---|
| C | 3.98511800 | -1.35161000 | 0.26624100 |
| H | 3.48807600 | -1.66298000 | 1.19088500 |
| H | 4.70757600 | -2.13536500 | 0.02192300 |
| C | 4.74575600 | -0.03397800 | 0.47507700 |
| H | 5.52983900 | -0.21381600 | 1.21610400 |
| H | 5.26015700 | 0.23244900 | -0.45613100 |
| F | -5.79805900 | -0.01381500 | 0.28586000 |
| H | -1.78057000 | -1.92237300 | 0.95444000 |
| H | -4.26365900 | -1.92342200 | 1.13703300 |
| H | -4.42541900 | 1.89560200 | -0.80550600 |
| H | -1.93616700 | 1.88943500 | -1.03597500 |

**M062X**

| | | | |
|---|---|---|---|
| N | -0.28950000 | -0.01358700 | -0.22968200 |
| N | 0.43296800 | 1.11448300 | -0.01369600 |
| N | 1.74261400 | -0.56001700 | -0.57569300 |
| C | 0.49352600 | -1.02198700 | -0.57216200 |
| H | 0.17854200 | -2.02355300 | -0.81891300 |
| C | 1.67366000 | 0.76922500 | -0.23010000 |
| C | -1.71340000 | -0.01915500 | -0.08108300 |
| C | -2.43295800 | 1.07886400 | -0.53546200 |
| C | -3.81331600 | 1.08003900 | -0.39744000 |
| C | -4.42162700 | -0.02215300 | 0.17987200 |
| C | -3.71350100 | -1.11961200 | 0.63600100 |

| | | | |
|---|---|---|---|
| C | -2.33055600 | -1.11282400 | 0.51173300 |
| C | 2.96577800 | -1.32604300 | -0.88334800 |
| H | 3.41873500 | -0.87109700 | -1.76677500 |
| H | 2.63608000 | -2.32970100 | -1.14592900 |
| C | 2.85954300 | 1.65296800 | -0.08706400 |
| H | 3.35543400 | 1.74091900 | -1.05951600 |
| H | 2.48023300 | 2.63960100 | 0.17839100 |
| C | 3.85355400 | 1.14860700 | 0.97153500 |
| H | 3.31145100 | 0.90941800 | 1.89183200 |
| H | 4.52383000 | 1.97767200 | 1.20580400 |
| C | 3.93200200 | -1.35244400 | 0.29599600 |
| H | 3.38468600 | -1.64631000 | 1.19705800 |
| H | 4.65080200 | -2.14781000 | 0.08675800 |
| C | 4.69774400 | -0.04791200 | 0.52824800 |
| H | 5.45461000 | -0.23323700 | 1.29389000 |
| H | 5.23953400 | 0.21513600 | -0.38729800 |
| F | -5.75836900 | -0.02217300 | 0.30890900 |
| H | -1.74666900 | -1.94233100 | 0.89062800 |
| H | -4.23380500 | -1.95003900 | 1.09470200 |
| H | -4.41383600 | 1.91419600 | -0.73579900 |
| H | -1.91956900 | 1.91511100 | -0.99186900 |

**9k (B3LYP)**

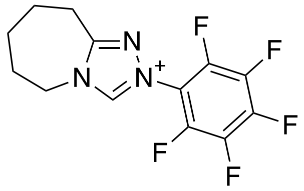


| | | | |
|---|---|---|---|
| N | -0.34688500 | -0.22488200 | -0.05750700 |
| N | -1.07547600 | -0.18199500 | 1.10528300 |
| N | -2.38557200 | -0.48316900 | -0.65163400 |
| C | -1.13866900 | -0.40262800 | -1.11039500 |
| H | -0.82259800 | -0.46633800 | -2.13894400 |
| C | -2.31771600 | -0.34303800 | 0.72620900 |
| C | 1.06848200 | -0.08299000 | -0.04475200 |
| C | 1.88803000 | -1.20802500 | 0.02855400 |
| C | 3.26857100 | -1.07241300 | 0.05592600 |
| C | 3.83444700 | 0.19695400 | 0.01871500 |
| C | 3.02741400 | 1.32732500 | -0.04653700 |
| C | 1.64775100 | 1.18420900 | -0.07510900 |
| C | -3.60631900 | -0.68076400 | -1.47319800 |
| H | -4.01913300 | -1.65544100 | -1.20635000 |
| H | -3.26694400 | -0.73111700 | -2.50622100 |
| C | -3.49430400 | -0.35799600 | 1.63714600 |
| H | -3.94284700 | -1.35765400 | 1.61532800 |
| H | -3.10646200 | -0.20434900 | 2.64474400 |
| C | -4.56163600 | 0.70619200 | 1.30479200 |
| H | -4.08351900 | 1.68838100 | 1.22912700 |

| | | | |
|---|---|---|---|
| H | -5.23705700 | 0.75438600 | 2.16243900 |
| C | -4.63002500 | 0.43881700 | -1.28592900 |
| H | -4.13797000 | 1.40491800 | -1.43897500 |
| H | -5.35028600 | 0.32418700 | -2.10078000 |
| C | -5.39281800 | 0.42149700 | 0.04680600 |
| H | -6.18545100 | 1.17264900 | -0.01317400 |
| H | -5.89618900 | -0.54633800 | 0.15879100 |
| F | 3.58086800 | 2.53958400 | -0.08175600 |
| F | 0.87820600 | 2.26929900 | -0.13841300 |
| F | 5.15673400 | 0.33153400 | 0.04463800 |
| F | 4.05076000 | -2.15000700 | 0.12065000 |
| F | 1.34951400 | -2.42550100 | 0.06906300 |

**M062X**

| | | | |
|---|---|---|---|
| N | -0.34938000 | -0.22876100 | 0.04803200 |
| N | -1.07605800 | 0.29982100 | 1.07080600 |
| N | -2.36985700 | -0.73972200 | -0.37784300 |
| C | -1.12716900 | -0.85115200 | -0.82624900 |
| H | -0.80361100 | -1.34722100 | -1.72869100 |
| C | -2.31029600 | -0.01936700 | 0.79844700 |
| C | 1.06094200 | -0.08942400 | 0.00498800 |
| C | 1.88103200 | -1.19560700 | 0.18081600 |
| C | 3.25721900 | -1.05615800 | 0.14617400 |
| C | 3.81259000 | 0.19848300 | -0.05145100 |

| | | | |
|---|---|---|---|
| C | 3.00136300 | 1.31096500 | -0.21684900 |
| C | 1.62637700 | 1.16412700 | -0.19033700 |
| C | -3.59056800 | -1.26448100 | -1.02321300 |
| H | -4.02964200 | -1.98853700 | -0.33401400 |
| H | -3.25595500 | -1.79531300 | -1.91255800 |
| C | -3.50100100 | 0.34417300 | 1.60934500 |
| H | -3.97474800 | -0.57652200 | 1.96632300 |
| H | -3.12845100 | 0.88173100 | 2.48103600 |
| C | -4.51880400 | 1.19860900 | 0.83528400 |
| H | -3.99959900 | 2.03070600 | 0.34965300 |
| H | -5.19663900 | 1.63475700 | 1.57128600 |
| C | -4.56938600 | -0.15054800 | -1.37523700 |
| H | -4.03237400 | 0.64154900 | -1.90608800 |
| H | -5.27974100 | -0.58166100 | -2.08425800 |
| C | -5.34736500 | 0.42159800 | -0.18839700 |
| H | -6.11609900 | 1.09025200 | -0.58222700 |
| H | -5.87406100 | -0.39419600 | 0.31956700 |
| F | 3.54415900 | 2.50257100 | -0.41100400 |
| F | 0.85160800 | 2.22130300 | -0.36173800 |
| F | 5.12534900 | 0.33479200 | -0.08695100 |
| F | 4.04123600 | -2.11041800 | 0.30586800 |
| F | 1.34969000 | -2.39071600 | 0.38194400 |

## S2.3 Coordinates of Carbenes

### 7a (B3LYP)

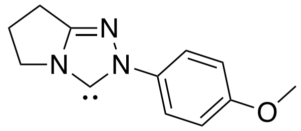

| | | | |
|---|---|---|---|
| O | -4.75960800 | 0.46185500 | -0.11844500 |
| N | 0.80000900 | -0.07760100 | 0.00518900 |
| N | 1.56136100 | 1.05231100 | 0.34494200 |
| N | 2.78115600 | -0.71466300 | -0.13502300 |
| C | 1.51728200 | -1.19192900 | -0.30128200 |
| C | 2.77512600 | 0.60216600 | 0.24466600 |
| C | 4.16268400 | 1.11424900 | 0.45170800 |
| H | 4.38013900 | 1.17925500 | 1.52209500 |
| H | 4.32075800 | 2.10182300 | 0.01869100 |
| C | 5.01123400 | 0.00584100 | -0.24025100 |
| H | 5.96513700 | -0.15661800 | 0.25959500 |
| H | 5.21614100 | 0.29834200 | -1.27178700 |
| C | 4.13427500 | -1.27333300 | -0.23306000 |
| H | 4.24096800 | -1.87079900 | -1.13705100 |
| H | 4.33148000 | -1.90583000 | 0.63536700 |
| C | -0.62163200 | 0.03735600 | -0.01186100 |
| C | -1.41745600 | -1.07349700 | 0.25428700 |
| H | -0.95151100 | -2.02110700 | 0.48968700 |
| C | -2.80856400 | -0.97307100 | 0.21943100 |
| H | -3.40132300 | -1.85248300 | 0.42997400 |

| | | | |
|---|---|---|---|
| C | -3.41178300 | 0.25495500 | -0.06784300 |
| C | -2.60745900 | 1.37330500 | -0.32241700 |
| H | -3.08194600 | 2.32163600 | -0.54493300 |
| C | -1.22452100 | 1.26592000 | -0.29996300 |
| H | -0.60906000 | 2.13155900 | -0.50487400 |
| C | -5.63030500 | -0.64358700 | 0.13360000 |
| H | -5.48469200 | -1.03910100 | 1.14296100 |
| H | -5.47929600 | -1.44076000 | -0.60010500 |
| H | -6.64089700 | -0.25134200 | 0.04008800 |

**M062X**

| | | | |
|---|---|---|---|
| O | -4.74043100 | 0.46468300 | -0.11198500 |
| N | 0.79860700 | -0.07415400 | 0.02005300 |
| N | 1.55139700 | 1.05158800 | 0.32141800 |
| N | 2.76607600 | -0.71960000 | -0.10846600 |
| C | 1.50480600 | -1.19926700 | -0.25676500 |
| C | 2.76204300 | 0.60346900 | 0.23197300 |
| C | 4.15222500 | 1.11576300 | 0.41618800 |
| H | 4.38215000 | 1.17400300 | 1.48295700 |
| H | 4.30243700 | 2.10036300 | -0.02252500 |
| C | 4.97662600 | 0.00413200 | -0.28456700 |
| H | 5.95595200 | -0.13439300 | 0.16879100 |
| H | 5.11715800 | 0.26901300 | -1.33340400 |
| C | 4.11587500 | -1.27591000 | -0.19627800 |

| | | | |
|---|---|---|---|
| H | 4.20947200 | -1.91933300 | -1.06853500 |
| H | 4.32878100 | -1.85413000 | 0.70454900 |
| C | -0.62111500 | 0.04184900 | -0.00053300 |
| C | -1.41201000 | -1.07374200 | 0.23493300 |
| H | -0.94198100 | -2.02502100 | 0.44732800 |
| C | -2.80074700 | -0.97282800 | 0.19776200 |
| H | -3.39382000 | -1.85734600 | 0.38529800 |
| C | -3.39869300 | 0.26001100 | -0.06194100 |
| C | -2.59665000 | 1.38227900 | -0.28716000 |
| H | -3.07504800 | 2.33316600 | -0.48836100 |
| C | -1.21677000 | 1.27559300 | -0.26243600 |
| H | -0.59673300 | 2.14303300 | -0.44618400 |
| C | -5.58640600 | -0.65539700 | 0.09938200 |
| H | -5.43599900 | -1.07810700 | 1.09630900 |
| H | -5.41238000 | -1.42565600 | -0.65677800 |
| H | -6.60420500 | -0.28361100 | 0.01206100 |

**7b (B3LYP)**

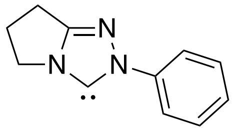

| | | | |
|---|---|---|---|
| N | 0.06537700 | -0.10885100 | -0.02262400 |
| N | -0.65966700 | 1.07139600 | -0.25497500 |
| N | -1.93528700 | -0.68954600 | 0.08332600 |
| C | -0.68993400 | -1.22189600 | 0.19249800 |
| C | -1.88634300 | 0.65541900 | -0.18275500 |
| C | -3.25660900 | 1.22938300 | -0.33305200 |
| H | -3.47603000 | 1.39627300 | -1.39185600 |
| H | -3.37834200 | 2.17936900 | 0.18690600 |
| C | -4.13990300 | 0.09350300 | 0.26486300 |
| H | -5.09888500 | 0.00616500 | -0.24382400 |
| H | -4.33446700 | 0.30292900 | 1.31829500 |
| C | -3.30632900 | -1.20895700 | 0.14461200 |
| H | -3.42818700 | -1.87565800 | 0.99670800 |
| H | -3.52790500 | -1.76045300 | -0.77170700 |
| C | 1.48944500 | -0.04613500 | -0.00410300 |
| C | 2.23815600 | -1.20783700 | -0.21359300 |
| H | 1.72875500 | -2.14397800 | -0.39753100 |
| C | 3.62881800 | -1.14499500 | -0.18323100 |
| H | 4.20416200 | -2.04920900 | -0.34618900 |
| C | 4.28011900 | 0.06856800 | 0.04207500 |
| C | 3.52534400 | 1.22371800 | 0.24126500 |

| | | | |
|---|---|---|---|
| H | 4.01870800 | 2.17262200 | 0.41826600 |
| C | 2.13241800 | 1.17284400 | 0.22279200 |
| H | 1.54535400 | 2.06648200 | 0.38319300 |
| H | 5.36278700 | 0.11277400 | 0.05942900 |

**M062X**

| | | | |
|---|---|---|---|
| N | 0.06104300 | -0.10817800 | -0.02889800 |
| N | -0.65241100 | 1.05866300 | -0.26435600 |
| N | -1.92791600 | -0.68917200 | 0.07812300 |
| C | -0.68620600 | -1.22170600 | 0.19072300 |
| C | -1.87717600 | 0.65005400 | -0.19268500 |
| C | -3.24833800 | 1.21973700 | -0.34595000 |
| H | -3.47595900 | 1.34037500 | -1.40799300 |
| H | -3.36355700 | 2.18550200 | 0.14221000 |
| C | -4.11077500 | 0.10330500 | 0.29861900 |
| H | -5.09537600 | 0.02378700 | -0.15733800 |
| H | -4.23928500 | 0.31731500 | 1.36054400 |
| C | -3.29735600 | -1.20028000 | 0.13941100 |
| H | -3.41387800 | -1.88636200 | 0.97549300 |
| H | -3.53012500 | -1.72086700 | -0.79105800 |
| C | 1.48320700 | -0.04568800 | -0.00529300 |
| C | 2.22705500 | -1.20578700 | -0.21284100 |
| H | 1.71531600 | -2.14050600 | -0.39897300 |
| C | 3.61491200 | -1.14133600 | -0.17972800 |

| | | | |
|---|---|---|---|
| H | 4.19109300 | -2.04442300 | -0.34255100 |
| C | 4.26250300 | 0.07073800 | 0.04710200 |
| C | 3.50893000 | 1.22327500 | 0.24531200 |
| H | 4.00155500 | 2.17174200 | 0.42363400 |
| C | 2.11874300 | 1.17191200 | 0.22386500 |
| H | 1.52768200 | 2.06327500 | 0.38430100 |
| H | 5.34452500 | 0.11562400 | 0.06644000 |

**7c (B3LYP)**

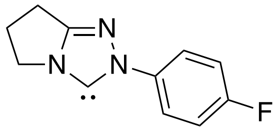


| | | | |
|---|---|---|---|
| N | 0.35890500 | 0.11632800 | -0.02519100 |
| N | 1.07669500 | -1.06719900 | -0.26123800 |
| N | 2.36163400 | 0.68514900 | 0.08749400 |
| C | 1.11913400 | 1.22437300 | 0.19649500 |
| C | 2.30579300 | -0.65839500 | -0.18399500 |
| C | 3.67298200 | -1.23970400 | -0.33299600 |
| H | 3.89372200 | -1.40428100 | -1.39187800 |
| H | 3.78779800 | -2.19216200 | 0.18400100 |
| C | 4.56140400 | -0.11100600 | 0.27091000 |
| H | 5.52185600 | -0.02721900 | -0.23558600 |
| H | 4.75263200 | -0.32543000 | 1.32394100 |
| C | 3.73555000 | 1.19659700 | 0.15379600 |
| H | 3.85914400 | 1.85930900 | 1.00873100 |
| H | 3.96204200 | 1.75032500 | -0.75994900 |
| C | -1.06424300 | 0.06157500 | -0.01121800 |
| C | -1.80621800 | 1.22635700 | -0.22570000 |
| H | -1.29418100 | 2.16045900 | -0.41046300 |
| C | -3.19697400 | 1.18080600 | -0.20025600 |
| H | -3.78810100 | 2.07288600 | -0.36483300 |
| C | -3.81923500 | -0.03649100 | 0.02642400 |
| C | -3.10704500 | -1.20571700 | 0.23187700 |

| | | | |
|---|---|---|---|
| H | -3.62842100 | -2.13828100 | 0.40729600 |
| C | -1.71486000 | -1.15288100 | 0.21655300 |
| H | -1.13603100 | -2.05068000 | 0.38105700 |
| F | -5.18098100 | -0.08421500 | 0.04432100 |

**M062X**

| | | | |
|---|---|---|---|
| N | 0.36088500 | 0.11705500 | -0.03464000 |
| N | 1.06716900 | -1.05085200 | -0.28401700 |
| N | 2.35189500 | 0.68428500 | 0.08830200 |
| C | 1.11274800 | 1.22267500 | 0.20459800 |
| C | 2.29430300 | -0.65062100 | -0.20142100 |
| C | 3.66253100 | -1.22676900 | -0.35640900 |
| H | 3.89427700 | -1.33383700 | -1.41899700 |
| H | 3.76924900 | -2.19998600 | 0.11874100 |
| C | 4.52877700 | -0.12502200 | 0.30795200 |
| H | 5.51585500 | -0.04505600 | -0.14250600 |
| H | 4.65118500 | -0.35503800 | 1.36722900 |
| C | 3.72425000 | 1.18587800 | 0.16404000 |
| H | 3.84045100 | 1.85830000 | 1.01120700 |
| H | 3.96500200 | 1.71915200 | -0.75709700 |
| C | -1.06061600 | 0.06195800 | -0.01637000 |
| C | -1.79810800 | 1.22279000 | -0.23943300 |
| H | -1.28342600 | 2.15363600 | -0.43531800 |
| C | -3.18584300 | 1.17552900 | -0.20914400 |

| | | | |
|---|---|---|---|
| H | -3.78218400 | 2.06254000 | -0.38114100 |
| C | -3.80387300 | -0.03865200 | 0.03123900 |
| C | -3.09160600 | -1.20354400 | 0.24497100 |
| H | -3.61533000 | -2.13241600 | 0.43105400 |
| C | -1.70259900 | -1.14956400 | 0.22469700 |
| H | -1.11816600 | -2.04287600 | 0.39686700 |
| F | -5.15292700 | -0.08730900 | 0.05490300 |

**7d (B3LYP)**

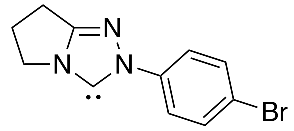


| | | | |
|---|---|---|---|
| N | 1.53476600 | 0.11563700 | -0.04084900 |
| N | 2.24470900 | -1.09577900 | -0.03896400 |
| N | 3.54216500 | 0.68172800 | -0.04471600 |
| C | 2.30579900 | 1.24088300 | -0.04738800 |
| C | 3.47613100 | -0.68907300 | -0.04477100 |
| C | 4.83897600 | -1.29789500 | -0.07344000 |
| H | 5.05765700 | -1.67032900 | -1.07871900 |
| H | 4.94725300 | -2.13022600 | 0.62157400 |
| C | 5.73555700 | -0.07805800 | 0.29525400 |
| H | 6.69757700 | -0.10449500 | -0.21438800 |
| H | 5.92272400 | -0.07847400 | 1.37060600 |
| C | 4.92039100 | 1.18535400 | -0.08462500 |
| H | 5.05048900 | 2.00728600 | 0.61732100 |
| H | 5.14865600 | 1.53908700 | -1.09232600 |
| C | 0.11268400 | 0.07702100 | -0.02466000 |
| C | -0.61778400 | 1.26927400 | -0.00876900 |
| H | -0.09687000 | 2.21645300 | -0.00702600 |
| C | -2.00839700 | 1.23635300 | 0.00488400 |
| H | -2.56804400 | 2.16262200 | 0.01733900 |
| C | -2.66386500 | 0.00820700 | 0.00301600 |
| C | -1.94994200 | -1.18409100 | -0.01251100 |

| | | | |
|---|---|---|---|
| H | -2.46285500 | -2.13703000 | -0.01398100 |
| C | -0.55706000 | -1.14847100 | -0.02675400 |
| H | 0.00588100 | -2.07052200 | -0.03924800 |
| Br | -4.58596800 | -0.03892900 | 0.02311800 |

**M062X**

| | | | |
|---|---|---|---|
| N | 1.52918100 | 0.11474500 | -0.04521600 |
| N | 2.22817800 | -1.08392400 | -0.07145100 |
| N | 3.52522200 | 0.68128000 | -0.03648200 |
| C | 2.29293300 | 1.24092300 | -0.02509900 |
| C | 3.45727800 | -0.68479100 | -0.06840600 |
| C | 4.82093000 | -1.28974600 | -0.11042300 |
| H | 5.04878200 | -1.60240500 | -1.13245700 |
| H | 4.92097700 | -2.15310000 | 0.54437900 |
| C | 5.69775700 | -0.08707000 | 0.32692200 |
| H | 6.68268900 | -0.10324100 | -0.13477500 |
| H | 5.82465900 | -0.11088900 | 1.41006400 |
| C | 4.90134500 | 1.17832900 | -0.06235000 |
| H | 5.02554100 | 1.99850000 | 0.64127500 |
| H | 5.14097600 | 1.52388300 | -1.06929400 |
| C | 0.10872800 | 0.07639300 | -0.02653200 |
| C | -0.61606200 | 1.26775100 | -0.03573300 |
| H | -0.09258900 | 2.21352700 | -0.05623000 |
| C | -2.00357900 | 1.23345700 | -0.01934800 |

| | | | |
|---|---|---|---|
| H | -2.56826500 | 2.15677600 | -0.02660300 |
| C | -2.65610600 | 0.00698600 | 0.00496300 |
| C | -1.94397400 | -1.18342700 | 0.01414500 |
| H | -2.46063600 | -2.13424800 | 0.03407500 |
| C | -0.55400800 | -1.14791000 | -0.00119900 |
| H | 0.01274000 | -2.06798100 | 0.00639500 |
| Br | -4.55841100 | -0.03916900 | 0.02495900 |

**7e (B3LYP)**

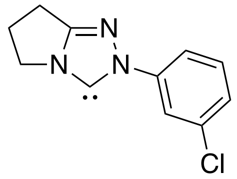


| | | | |
|---|---|---|---|
| N | 0.63065200 | 0.38495400 | -0.03559600 |
| N | 1.10755100 | -0.93567200 | -0.04426700 |
| N | 2.70713300 | 0.57574800 | -0.04584700 |
| C | 1.59416600 | 1.35124100 | -0.03937300 |
| C | 2.39224400 | -0.76030200 | -0.05359100 |
| C | 3.62097400 | -1.60733500 | -0.09027000 |
| H | 3.76505200 | -2.00820700 | -1.09804500 |
| H | 3.57676600 | -2.44890300 | 0.60057900 |
| C | 4.72635900 | -0.57381500 | 0.28102800 |
| H | 5.66518400 | -0.77203900 | -0.23398800 |
| H | 4.91501400 | -0.61580900 | 1.35524700 |
| C | 4.15407300 | 0.81997100 | -0.08682800 |
| H | 4.43299900 | 1.59862800 | 0.62102800 |
| H | 4.44092800 | 1.13447800 | -1.09257600 |
| C | -0.77411400 | 0.60711100 | -0.01116100 |
| C | -1.27781500 | 1.91157500 | 0.01186900 |
| H | -0.59295500 | 2.74729800 | 0.01282000 |
| C | -2.65321300 | 2.11451100 | 0.03360600 |
| H | -3.04167700 | 3.12586900 | 0.05142100 |
| C | -3.54292500 | 1.03968200 | 0.03355900 |

| | | | |
|---|---|---|---|
| C | -3.01387200 | -0.24469800 | 0.01070500 |
| C | -1.64503900 | -0.48532600 | -0.01184500 |
| H | -1.25663400 | -1.49232000 | -0.02933600 |
| Cl | -4.11141500 | -1.62812700 | 0.01033400 |
| H | -4.61300000 | 1.19825400 | 0.05094500 |

**M062X**

| | | | |
|---|---|---|---|
| N | 0.63058700 | 0.37337300 | -0.02732000 |
| N | 1.10317600 | -0.92490300 | 0.10410800 |
| N | 2.69316600 | 0.56279300 | -0.13955000 |
| C | 1.58144200 | 1.33390800 | -0.18661900 |
| C | 2.38270900 | -0.75925800 | 0.02689400 |
| C | 3.61428800 | -1.60179200 | 0.04927200 |
| H | 3.73833500 | -2.08946600 | -0.92078700 |
| H | 3.58753400 | -2.36964100 | 0.81991600 |
| C | 4.70867400 | -0.52974000 | 0.29245100 |
| H | 5.65399400 | -0.78851700 | -0.17957600 |
| H | 4.87639500 | -0.42481200 | 1.36520200 |
| C | 4.13326300 | 0.79251000 | -0.26231500 |
| H | 4.43164600 | 1.66691200 | 0.31184400 |
| H | 4.38553900 | 0.94465600 | -1.31299400 |
| C | -0.77138900 | 0.59800800 | 0.01288200 |
| C | -1.26526300 | 1.89900200 | 0.10238900 |
| H | -0.57489000 | 2.72931300 | 0.14891700 |

| | | | |
|---|---|---|---|
| C | -2.63774700 | 2.10478400 | 0.13124900 |
| H | -3.02364300 | 3.11435800 | 0.20117200 |
| C | -3.52724600 | 1.03525500 | 0.07942100 |
| C | -3.00446500 | -0.24645200 | -0.00391100 |
| C | -1.63892300 | -0.49001000 | -0.04027800 |
| H | -1.25405800 | -1.49708500 | -0.10963800 |
| Cl | -4.09788100 | -1.61023300 | -0.07048600 |
| H | -4.59742600 | 1.19210800 | 0.10492400 |

**7f (B3LYP)**

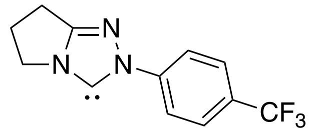


| | | | |
|---|---|---|---|
| N | 1.46476000 | -0.11304300 | 0.02960300 |
| N | 2.17765600 | 1.09754100 | 0.04681400 |
| N | 3.47027400 | -0.68468000 | 0.04129900 |
| C | 2.23479500 | -1.24145400 | 0.02812300 |
| C | 3.40723900 | 0.68757600 | 0.05688900 |
| C | 4.77132700 | 1.29206000 | 0.10172900 |
| H | 4.98401400 | 1.65080400 | 1.11322800 |
| H | 4.88559400 | 2.13312000 | -0.58164500 |
| C | 5.66724500 | 0.07487200 | -0.27678300 |
| H | 6.62653600 | 0.09338300 | 0.23824200 |
| H | 5.85999500 | 0.08760300 | -1.35104900 |
| C | 4.84774600 | -1.19109000 | 0.08415100 |
| H | 4.98035700 | -2.00570800 | -0.62569700 |
| H | 5.06853500 | -1.55577900 | 1.08952700 |
| C | 0.04651500 | -0.07278500 | 0.00505100 |
| C | -0.62019700 | 1.15506400 | -0.01399000 |
| H | -0.05352800 | 2.07443800 | -0.01328300 |
| C | -2.01019300 | 1.18744600 | -0.03581200 |
| H | -2.51901100 | 2.14263000 | -0.05286700 |
| C | -2.74076800 | -0.00002200 | -0.04327700 |
| C | -2.07132300 | -1.22688600 | -0.02316700 |

| | | | |
|---|---|---|---|
| H | -2.62879100 | -2.15527400 | -0.03045900 |
| C | -0.68474100 | -1.26658300 | -0.00024700 |
| H | -0.16237400 | -2.21240700 | 0.01169800 |
| C | -4.23822700 | 0.03211200 | -0.01381600 |
| F | -4.73456300 | -0.08781900 | 1.25172800 |
| F | -4.79446700 | -0.97898600 | -0.72618500 |
| F | -4.74726500 | 1.18531600 | -0.50718800 |

**M062X**

| | | | |
|---|---|---|---|
| N | -1.46030700 | 0.11191000 | 0.04047300 |
| N | -2.16173400 | -1.08602900 | 0.06935800 |
| N | -3.45491800 | 0.68309500 | 0.04100300 |
| C | -2.22351300 | 1.24082100 | 0.02395600 |
| C | -3.38937600 | -0.68427500 | 0.07221900 |
| C | -4.75412600 | -1.28590500 | 0.11937700 |
| H | -4.97860700 | -1.59689100 | 1.14267400 |
| H | -4.85811500 | -2.14977200 | -0.53406600 |
| C | -5.62995600 | -0.08172600 | -0.31573300 |
| H | -6.61359000 | -0.09625900 | 0.14869800 |
| H | -5.75977700 | -0.10523100 | -1.39851700 |
| C | -4.83050900 | 1.18216000 | 0.07172900 |
| H | -4.95533100 | 2.00313200 | -0.63077000 |
| H | -5.06575700 | 1.52708600 | 1.07986000 |
| C | -0.04299500 | 0.07303900 | 0.01457000 |

| | | | |
|---|---|---|---|
| C | 0.61694200 | -1.15399600 | -0.01764300 |
| H | 0.04662000 | -2.07142400 | -0.02500400 |
| C | 2.00461400 | -1.18502200 | -0.04390100 |
| H | 2.51964900 | -2.13737300 | -0.07377900 |
| C | 2.72611800 | 0.00286500 | -0.04223500 |
| C | 2.06472100 | 1.22775500 | -0.00786600 |
| H | 2.62814500 | 2.15294800 | -0.00861700 |
| C | 0.68010200 | 1.26717900 | 0.01964800 |
| H | 0.15345700 | 2.21069600 | 0.04244200 |
| C | 4.22175200 | -0.03146400 | -0.01801000 |
| F | 4.71168600 | -0.02350000 | 1.23921600 |
| F | 4.76542200 | 1.02872300 | -0.63786900 |
| F | 4.71837700 | -1.13059400 | -0.60750600 |

**7g (B3LYP)**

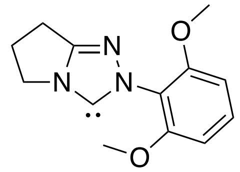


| | | | |
|---|---|---|---|
| N | 0.37887400 | -0.03778400 | -0.09912700 |
| N | 1.09954700 | -0.05724600 | 1.11016300 |
| N | 2.37874500 | -0.07476400 | -0.68187100 |
| C | 1.12981700 | -0.05110000 | -1.22684000 |
| C | 2.32701200 | -0.08292500 | 0.68892100 |
| C | 3.69744200 | -0.14399600 | 1.28203000 |
| H | 3.90149900 | -1.15584300 | 1.64490800 |
| H | 3.83067500 | 0.54312400 | 2.11743400 |
| C | 4.58691400 | 0.21526900 | 0.05462400 |
| H | 5.54007200 | -0.31167900 | 0.06649800 |
| H | 4.79378500 | 1.28717000 | 0.05907200 |
| C | 3.74932700 | -0.14130300 | -1.20088100 |
| H | 3.88692800 | 0.56158500 | -2.02109300 |
| H | 3.95497500 | -1.15155300 | -1.56198900 |
| C | -1.04392400 | 0.00705200 | -0.04673000 |
| C | -1.77791800 | -1.19024700 | -0.01334200 |
| C | -3.17620500 | -1.14590500 | 0.03894100 |
| H | -3.76105500 | -2.05406300 | 0.06210700 |
| C | -3.81337100 | 0.09137100 | 0.06325800 |
| C | -3.10199700 | 1.28752400 | 0.03781400 |

| | | | |
|---|---|---|---|
| H | -3.63067500 | 2.22951200 | 0.05971100 |
| C | -1.70368700 | 1.24693100 | -0.01406000 |
| H | -4.89614700 | 0.12449500 | 0.10389500 |
| O | -1.04985600 | -2.33500300 | -0.03732000 |
| O | -0.90724900 | 2.34498700 | -0.03801400 |
| C | -1.74106600 | -3.58843600 | -0.01417600 |
| H | -2.39006800 | -3.69597300 | -0.88718800 |
| H | -0.96492000 | -4.35021900 | -0.04144000 |
| H | -2.32828300 | -3.69730700 | 0.90139400 |
| C | -1.51951700 | 3.63878700 | -0.01319200 |
| H | -2.09905900 | 3.78261600 | 0.90248200 |
| H | -0.69761300 | 4.35100900 | -0.03935000 |
| H | -2.16040300 | 3.78767900 | -0.88612800 |

**M062X**

| | | | |
|---|---|---|---|
| N | 0.37945400 | -0.04537800 | -0.09641300 |
| N | 1.08752100 | -0.06528400 | 1.09918900 |
| N | 2.36707900 | -0.08899300 | -0.68035000 |
| C | 1.12247800 | -0.06124400 | -1.22641500 |
| C | 2.31328800 | -0.09549500 | 0.68575000 |
| C | 3.68382000 | -0.15939000 | 1.27637300 |
| H | 3.89246000 | -1.18001900 | 1.60643800 |
| H | 3.81117500 | 0.50969500 | 2.12511600 |
| C | 4.55675800 | 0.23408500 | 0.05604600 |

| | | | |
|---|---|---|---|
| H | 5.53085200 | -0.25045700 | 0.06918300 |
| H | 4.70960600 | 1.31421300 | 0.05792700 |
| C | 3.73541000 | -0.15896500 | -1.19207900 |
| H | 3.86902800 | 0.52545600 | -2.02712100 |
| H | 3.94816500 | -1.17767100 | -1.52106400 |
| C | -1.03952600 | 0.00739000 | -0.04490500 |
| C | -1.77377100 | -1.18283100 | -0.01254800 |
| C | -3.16901200 | -1.13140700 | 0.03806400 |
| H | -3.76184900 | -2.03450100 | 0.06031300 |
| C | -3.79413400 | 0.10941200 | 0.06155500 |
| C | -3.07868500 | 1.30057900 | 0.03714100 |
| H | -3.60297300 | 2.24494900 | 0.05935700 |
| C | -1.68352800 | 1.24849200 | -0.01306300 |
| H | -4.87625200 | 0.14963800 | 0.10115100 |
| O | -1.05076500 | -2.32255200 | -0.03605300 |
| O | -0.87852000 | 2.33195800 | -0.03610400 |
| C | -1.75867000 | -3.55595300 | -0.01236400 |
| H | -2.41328600 | -3.64545300 | -0.88249300 |
| H | -1.00047400 | -4.33411500 | -0.04409500 |
| H | -2.34471800 | -3.65057500 | 0.90490300 |
| C | -1.49359800 | 3.61412600 | -0.01394200 |
| H | -2.07206900 | 3.75254900 | 0.90261900 |
| H | -0.67994700 | 4.33403400 | -0.04477800 |
| H | -2.13879900 | 3.75178300 | -0.88486000 |

**7h (B3LYP)**

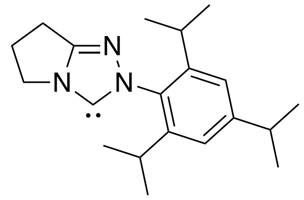

| | | | |
|---|---|---|---|
| N | 1.29816600 | -0.04780500 | -0.10594300 |
| N | 2.00493500 | -0.10231100 | 1.10959500 |
| N | 3.30274700 | -0.17850000 | -0.66630200 |
| C | 2.06091000 | -0.09549600 | -1.22494300 |
| C | 3.23598800 | -0.18569300 | 0.70229000 |
| C | 4.59536700 | -0.31001000 | 1.31079200 |
| H | 4.74925100 | -1.33093700 | 1.67301700 |
| H | 4.74986000 | 0.36837400 | 2.14965200 |
| C | 5.51337300 | 0.01158000 | 0.09399900 |
| H | 6.44257800 | -0.55624800 | 0.11540700 |
| H | 5.76630400 | 1.07354000 | 0.10246900 |
| C | 4.67457800 | -0.30665500 | -1.17092000 |
| H | 4.85226100 | 0.38939500 | -1.98921900 |
| H | 4.83804400 | -1.32512700 | -1.53042300 |
| C | -0.13580700 | 0.06114700 | -0.06207500 |
| C | -0.90881900 | -1.11443000 | -0.03933500 |
| C | -2.29846400 | -0.98060600 | 0.00143900 |
| H | -2.90704900 | -1.87808200 | 0.01545300 |
| C | -2.92511100 | 0.26841200 | 0.02657000 |
| C | -2.12033700 | 1.40776800 | 0.01080600 |

| | | | |
|---|---|---|---|
| H | -2.59739700 | 2.38228800 | 0.03094300 |
| C | -0.72372100 | 1.33618500 | -0.03001000 |
| C | -0.28155600 | -2.50444000 | -0.05872700 |
| H | 0.80220700 | -2.38182000 | -0.08000800 |
| C | 0.10159500 | 2.61793100 | -0.04269800 |
| H | 1.15552100 | 2.33622900 | -0.06007900 |
| C | -4.44191400 | 0.39371600 | 0.06937100 |
| H | -4.67108900 | 1.46450300 | 0.09953700 |
| C | -0.67514700 | -3.28811600 | -1.32505100 |
| H | -1.75187800 | -3.47772900 | -1.36115800 |
| H | -0.39783900 | -2.74219100 | -2.23061000 |
| H | -0.16555100 | -4.25599500 | -1.34299200 |
| C | -0.62591700 | -3.30115300 | 1.21371900 |
| H | -0.31521900 | -2.76416700 | 2.11380300 |
| H | -1.70017000 | -3.49298900 | 1.28896900 |
| H | -0.11490500 | -4.26838900 | 1.20253000 |
| C | -0.16945400 | 3.45162400 | -1.30910200 |
| H | 0.02827300 | 2.87109100 | -2.21401900 |
| H | -1.20779600 | 3.79365300 | -1.34776500 |
| H | 0.47438500 | 4.33596600 | -1.32552100 |
| C | -0.12655200 | 3.45389300 | 1.23070500 |
| H | -1.16004700 | 3.80523900 | 1.30105200 |
| H | 0.09380500 | 2.87275700 | 2.13001800 |
| H | 0.52441200 | 4.33311400 | 1.22679400 |
| C | -5.03673800 | -0.24121100 | 1.33974300 |

| | | | |
|---|---|---|---|
| H | -6.11772800 | -0.07747500 | 1.37656000 |
| H | -4.86238400 | -1.32105900 | 1.36424000 |
| H | -4.59689000 | 0.19188800 | 2.24216000 |
| C | -5.10117600 | -0.18363800 | -1.19687300 |
| H | -6.18274100 | -0.02113000 | -1.17116600 |
| H | -4.70814200 | 0.29148200 | -2.09971800 |
| H | -4.92759300 | -1.26072100 | -1.27956000 |

**M062X**

| | | | |
|---|---|---|---|
| N | -1.28995800 | -0.06240200 | 0.08912500 |
| N | -1.98843800 | -0.08415700 | -1.11033000 |
| N | -3.27856500 | -0.21712300 | 0.65574800 |
| C | -2.03789700 | -0.14400300 | 1.21171800 |
| C | -3.21629500 | -0.18428200 | -0.70788900 |
| C | -4.57827700 | -0.29506400 | -1.31117300 |
| H | -4.73751900 | -1.31489200 | -1.66984000 |
| H | -4.72989200 | 0.38990500 | -2.14307600 |
| C | -5.47584000 | 0.02636700 | -0.08773600 |
| H | -6.42729700 | -0.50025600 | -0.12088200 |
| H | -5.67601600 | 1.09845400 | -0.06221600 |
| C | -4.64555700 | -0.36322800 | 1.15552000 |
| H | -4.81532800 | 0.29064400 | 2.00830300 |
| H | -4.81320800 | -1.39936600 | 1.45477000 |
| C | 0.13749500 | 0.05912600 | 0.04387400 |

| | | | |
|---|---|---|---|
| C | 0.91386100 | -1.10676900 | 0.01234300 |
| C | 2.29898100 | -0.96251700 | -0.01592500 |
| H | 2.91771400 | -1.85491100 | -0.03083900 |
| C | 2.90951000 | 0.29244400 | -0.02381100 |
| C | 2.09910100 | 1.42464100 | -0.00665000 |
| H | 2.56712000 | 2.40467600 | -0.01536600 |
| C | 0.70603500 | 1.33559300 | 0.02430600 |
| C | 0.29302500 | -2.49188700 | 0.02386900 |
| H | -0.79171400 | -2.37930600 | -0.02673000 |
| C | -0.13767700 | 2.59726000 | 0.04510400 |
| H | -1.18944400 | 2.30340400 | 0.03796000 |
| C | 4.42000900 | 0.41986600 | -0.05070000 |
| H | 4.65490800 | 1.48893000 | -0.05548200 |
| C | 0.62788900 | -3.22487100 | 1.32832500 |
| H | 1.70612400 | -3.38454300 | 1.41839700 |
| H | 0.29460200 | -2.65180000 | 2.19659500 |
| H | 0.13864100 | -4.20167800 | 1.35007100 |
| C | 0.73321200 | -3.31157700 | -1.19427700 |
| H | 0.49073200 | -2.79356700 | -2.12484200 |
| H | 1.81026400 | -3.49781200 | -1.17805200 |
| H | 0.22670900 | -4.27968500 | -1.19677000 |
| C | 0.11551200 | 3.40178500 | 1.32524100 |
| H | -0.09305100 | 2.80077000 | 2.21332100 |
| H | 1.15500600 | 3.73713100 | 1.37618100 |
| H | -0.52546600 | 4.28647400 | 1.34843300 |

| | | | |
|---|---|---|---|
| C | 0.11641600 | 3.45289100 | -1.20110300 |
| H | 1.15150900 | 3.80325000 | -1.23417300 |
| H | -0.08045300 | 2.88536700 | -2.11348500 |
| H | -0.53426400 | 4.33065100 | -1.19404500 |
| C | 5.01057300 | -0.20165700 | -1.32142600 |
| H | 6.09226200 | -0.05013800 | -1.35020400 |
| H | 4.82136500 | -1.27833000 | -1.35106800 |
| H | 4.57556500 | 0.24483000 | -2.21821000 |
| C | 5.05336800 | -0.19994000 | 1.20020000 |
| H | 6.13519400 | -0.04672000 | 1.19350400 |
| H | 4.64722300 | 0.24610800 | 2.11063600 |
| H | 4.86677300 | -1.27688400 | 1.23644300 |

**7i (B3LYP)**

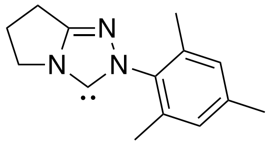

| | | | |
|---|---|---|---|
| N | -0.50531800 | 0.03898800 | -0.10695100 |
| N | -1.21660000 | 0.01945100 | 1.10788800 |
| N | -2.51224700 | 0.06270700 | -0.67120200 |
| C | -1.26652800 | 0.06884100 | -1.22650700 |
| C | -2.44880000 | 0.03874900 | 0.69788000 |
| C | -3.81456500 | 0.06673700 | 1.30417600 |
| H | -4.02891800 | 1.06645200 | 1.69376400 |
| H | -3.93107300 | -0.64240400 | 2.12355300 |
| C | -4.71004800 | -0.27461500 | 0.07597000 |
| H | -5.66978500 | 0.23936000 | 0.10838600 |
| H | -4.90308700 | -1.34891600 | 0.05633700 |
| C | -3.88790500 | 0.12267500 | -1.17756100 |
| H | -4.02283600 | -0.56263600 | -2.01295100 |
| H | -4.10997800 | 1.13817000 | -1.51338600 |
| C | 0.93082900 | 0.01203500 | -0.06400800 |
| C | 1.58874500 | -1.22573300 | -0.04897000 |
| C | 2.98572200 | -1.22506700 | -0.00473200 |
| H | 3.50893600 | -2.17621400 | 0.00211400 |
| C | 3.72236100 | -0.03807000 | 0.02917300 |
| C | 3.02826500 | 1.17486600 | 0.01251800 |
| H | 3.58487800 | 2.10669300 | 0.03317400 |

| | | | |
|---|---|---|---|
| C | 1.63235400 | 1.22550100 | -0.03053300 |
| C | 0.82058000 | -2.52355900 | -0.08781800 |
| H | 0.13604200 | -2.60671700 | 0.76070400 |
| H | 0.21531900 | -2.59838700 | -0.99561800 |
| H | 1.50245900 | -3.37454400 | -0.06071300 |
| C | 5.22989400 | -0.06489000 | 0.10952600 |
| H | 5.56351400 | -0.05666700 | 1.15291000 |
| H | 5.63725400 | -0.96463100 | -0.35656700 |
| H | 5.67001500 | 0.80657800 | -0.38015200 |
| C | 0.91037000 | 2.55009200 | -0.04867300 |
| H | 0.31823300 | 2.66571500 | -0.96079700 |
| H | 0.21953700 | 2.63879200 | 0.79414900 |
| H | 1.62099900 | 3.37595900 | 0.00431200 |

**M062X**

| | | | |
|---|---|---|---|
| N | -0.50054600 | 0.06644300 | -0.09643000 |
| N | -1.19971800 | -0.10259000 | 1.09261300 |
| N | -2.49493000 | 0.15762500 | -0.65596400 |
| C | -1.25282800 | 0.23373000 | -1.20634800 |
| C | -2.43023900 | -0.03498100 | 0.69510700 |
| C | -3.79681400 | -0.08405600 | 1.29617600 |
| H | -4.02198300 | 0.87477300 | 1.76962000 |
| H | -3.90500700 | -0.86927500 | 2.04188200 |
| C | -4.67079700 | -0.31656200 | 0.03634600 |

| | | | |
|---|---|---|---|
| H | -5.65623900 | 0.13611900 | 0.12507900 |
| H | -4.79737100 | -1.38902300 | -0.11915900 |
| C | -3.86884300 | 0.27438700 | -1.14426600 |
| H | -3.99591000 | -0.28020600 | -2.07155500 |
| H | -4.10409000 | 1.32607700 | -1.31732300 |
| C | 0.93111400 | 0.02545600 | -0.05829800 |
| C | 1.56647300 | -1.21645100 | -0.08967400 |
| C | 2.96006000 | -1.23638900 | -0.04884500 |
| H | 3.47420700 | -2.19220900 | -0.07811000 |
| C | 3.70535100 | -0.05974900 | 0.02359100 |
| C | 3.03013500 | 1.16098500 | 0.05123600 |
| H | 3.59988800 | 2.08395200 | 0.10351000 |
| C | 1.63834200 | 1.22704200 | 0.01591200 |
| C | 0.76181000 | -2.48518800 | -0.17827300 |
| H | 0.08270000 | -2.57559500 | 0.67265900 |
| H | 0.14773600 | -2.48952500 | -1.08247200 |
| H | 1.41633600 | -3.35611700 | -0.19685600 |
| C | 5.20978900 | -0.10327500 | 0.09833100 |
| H | 5.54212000 | -0.04826400 | 1.13873300 |
| H | 5.59871400 | -1.02947900 | -0.32644900 |
| H | 5.65464100 | 0.73879500 | -0.43415600 |
| C | 0.91174500 | 2.54489400 | 0.04758400 |
| H | 0.37720700 | 2.71411000 | -0.89026500 |
| H | 0.16969400 | 2.56116200 | 0.84935800 |
| H | 1.61192900 | 3.36530300 | 0.20249500 |

**7j (B3LYP)**

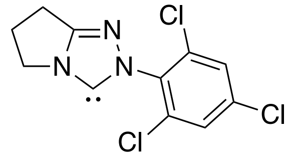

| | | | |
|---|---|---|---|
| N | -0.89825800 | 0.04415000 | -0.10010600 |
| N | -1.60484000 | 0.04013900 | 1.11827100 |
| N | -2.89676900 | 0.05781900 | -0.66807800 |
| C | -1.65678900 | 0.05697100 | -1.22594900 |
| C | -2.83453000 | 0.05256500 | 0.70406600 |
| C | -4.20045300 | 0.08365100 | 1.30771300 |
| H | -4.41763600 | 1.08892600 | 1.68066300 |
| H | -4.31293400 | -0.61257600 | 2.13835500 |
| C | -5.09312900 | -0.28095100 | 0.08421800 |
| H | -6.05560800 | 0.22788900 | 0.10966700 |
| H | -5.27931800 | -1.35650300 | 0.07992100 |
| C | -4.27428900 | 0.10421300 | -1.17481100 |
| H | -4.40395600 | -0.59261200 | -2.00107000 |
| H | -4.49920700 | 1.11456200 | -1.52283500 |
| C | 0.51960500 | 0.01649400 | -0.06163500 |
| C | 1.26487400 | 1.20177600 | -0.03193500 |
| C | 2.65454800 | 1.19131600 | 0.00784000 |
| H | 3.21020300 | 2.11794600 | 0.02593000 |
| C | 3.30546300 | -0.03628900 | 0.02435400 |
| C | 2.60866600 | -1.23844400 | 0.00387200 |
| H | 3.12879600 | -2.18556200 | 0.01881000 |

| | | | |
|---|---|---|---|
| C | 1.21964400 | -1.19619000 | -0.03657600 |
| Cl | 0.44497000 | 2.74505500 | -0.04777400 |
| Cl | 5.05741300 | -0.06918900 | 0.07261200 |
| Cl | 0.34156200 | -2.70689500 | -0.05936700 |

**M062X**

| | | | |
|---|---|---|---|
| N | -0.88983400 | 0.07536500 | -0.09634900 |
| N | -1.58431400 | 0.04555800 | 1.10736700 |
| N | -2.87608700 | 0.08507800 | -0.66642600 |
| C | -1.64001100 | 0.10028500 | -1.22455100 |
| C | -2.81241800 | 0.05679200 | 0.70101700 |
| C | -4.17939400 | 0.06478800 | 1.30122000 |
| H | -4.41586100 | 1.07146300 | 1.65422600 |
| H | -4.27792600 | -0.62590700 | 2.13616400 |
| C | -5.04754100 | -0.32940600 | 0.07778600 |
| H | -6.03822300 | 0.11887300 | 0.11108600 |
| H | -5.16064300 | -1.41414300 | 0.05313300 |
| C | -4.25180000 | 0.12535700 | -1.16543900 |
| H | -4.36837200 | -0.53872100 | -2.01898000 |
| H | -4.49765300 | 1.14617700 | -1.46228400 |
| C | 0.52385800 | 0.03263500 | -0.05852600 |
| C | 1.27651000 | 1.20608800 | -0.02647900 |
| C | 2.66301500 | 1.17588600 | 0.01066500 |
| H | 3.23415800 | 2.09390700 | 0.03034700 |

| | | | |
|---|---|---|---|
| C | 3.29295500 | -0.06012200 | 0.02311800 |
| C | 2.58232600 | -1.25184300 | 0.00168300 |
| H | 3.09106300 | -2.20594700 | 0.01458700 |
| C | 1.19727000 | -1.18839800 | -0.03719200 |
| Cl | 0.46597500 | 2.73788600 | -0.03463500 |
| Cl | 5.02985000 | -0.11755700 | 0.06706000 |
| Cl | 0.28396800 | -2.66033400 | -0.06479200 |

**7k (B3LYP)**

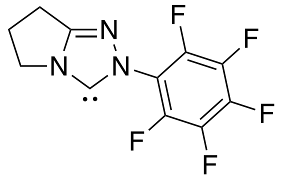


| | | | |
|---|---|---|---|
| N | 0.93789200 | -0.08694300 | -0.07610000 |
| N | 1.65059600 | 0.44353400 | 1.01905300 |
| N | 2.93274800 | -0.35844000 | -0.58724400 |
| C | 1.69371300 | -0.59456900 | -1.08876600 |
| C | 2.87707800 | 0.24525700 | 0.64680600 |
| C | 4.24530500 | 0.47366700 | 1.19925500 |
| H | 4.46028700 | -0.26988700 | 1.97226000 |
| H | 4.36241200 | 1.46175400 | 1.64329800 |
| C | 5.13357600 | 0.26452100 | -0.06305600 |
| H | 6.09430700 | -0.18699300 | 0.17961500 |
| H | 5.32302000 | 1.22964800 | -0.53626200 |
| C | 4.30882300 | -0.62748300 | -1.02597600 |
| H | 4.43673700 | -0.36163500 | -2.07368500 |
| H | 4.52793500 | -1.68947800 | -0.89812200 |
| C | -0.47407600 | -0.04239600 | -0.04923000 |
| C | -1.15295300 | 1.17303700 | -0.13091700 |
| C | -2.53907600 | 1.22729000 | -0.09552200 |
| C | -3.27130700 | 0.05213300 | 0.01348700 |
| C | -2.61568200 | -1.16919600 | 0.09289700 |
| C | -1.22797700 | -1.20920700 | 0.07024300 |

| | | | |
|---|---|---|---|
| F | -4.60476700 | 0.09740200 | 0.04132200 |
| F | -3.17247400 | 2.40213800 | -0.18008900 |
| F | -0.46943100 | 2.31429700 | -0.26317800 |
| F | -0.62189200 | -2.39568600 | 0.17437300 |
| F | -3.32231300 | -2.29912700 | 0.20619500 |

**M062X**

| | | | |
|---|---|---|---|
| N | 0.93567000 | -0.11809300 | -0.04729300 |
| N | 1.63922500 | 0.55947000 | 0.94485100 |
| N | 2.91759000 | -0.45662200 | -0.52355000 |
| C | 1.68219500 | -0.76545200 | -0.98204400 |
| C | 2.86282800 | 0.31685500 | 0.60700600 |
| C | 4.23304600 | 0.62756600 | 1.11010300 |
| H | 4.46720400 | -0.02642500 | 1.95356500 |
| H | 4.33882300 | 1.66057200 | 1.43483200 |
| C | 5.09450700 | 0.28592300 | -0.13321400 |
| H | 6.08453200 | -0.07559200 | 0.13572200 |
| H | 5.20792200 | 1.18056500 | -0.74706200 |
| C | 4.29143200 | -0.77095500 | -0.92243300 |
| H | 4.40558400 | -0.68358500 | -2.00056400 |
| H | 4.53147700 | -1.78917800 | -0.61252500 |
| C | -0.47218300 | -0.05964200 | -0.03171300 |
| C | -1.12897000 | 1.16089500 | -0.13752700 |
| C | -2.51053100 | 1.23315700 | -0.11522100 |

| | | | |
|---|---|---|---|
| C | -3.25614600 | 0.07180900 | 0.00179700 |
| C | -2.61957700 | -1.15352200 | 0.10296400 |
| C | -1.23604800 | -1.21330200 | 0.09560900 |
| F | -4.57979500 | 0.13331600 | 0.01613100 |
| F | -3.12360400 | 2.40609900 | -0.22069700 |
| F | -0.43284500 | 2.28124500 | -0.28348900 |
| F | -0.65116500 | -2.39698200 | 0.22405200 |
| F | -3.33773000 | -2.26363800 | 0.22467100 |

**8a (B3LYP)**

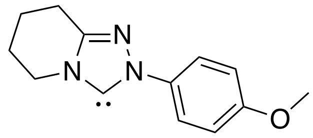


| | | | |
|---|---|---|---|
| N | 0.45610500 | -0.06478100 | -0.03238800 |
| N | 1.21543000 | 1.06232700 | 0.26723700 |
| N | 2.45007800 | -0.70887400 | -0.15757800 |
| C | 1.17347500 | -1.17674600 | -0.31025400 |
| C | 2.44123500 | 0.62880700 | 0.18007000 |
| C | -0.96654000 | 0.04312600 | -0.03277400 |
| C | -1.75360400 | -1.06542800 | 0.26569400 |
| C | -3.14552000 | -0.97142000 | 0.24637800 |
| C | -3.75774700 | 0.24778600 | -0.05898200 |
| C | -2.96151100 | 1.36407100 | -0.34648600 |
| C | -1.57796800 | 1.26313400 | -0.33805300 |
| C | 3.67327900 | -1.48591900 | -0.41666800 |
| H | 3.80074600 | -1.56909900 | -1.49968000 |
| H | 3.52360700 | -2.48828900 | -0.01611500 |
| C | 3.69008800 | 1.40843900 | 0.44737600 |
| H | 3.58075900 | 2.41799500 | 0.04612200 |
| H | 3.80596700 | 1.50961500 | 1.53248700 |
| C | 4.91945100 | 0.69368100 | -0.13717400 |
| H | 4.93874000 | 0.81726300 | -1.22553500 |
| H | 5.82747700 | 1.15755200 | 0.25355900 |
| C | 4.88783100 | -0.80044300 | 0.20952400 |

| | | | |
|---|---|---|---|
| H | 4.86387300 | -0.92566600 | 1.29770200 |
| H | 5.78901400 | -1.30082000 | -0.15213900 |
| H | -1.28043300 | -2.00591300 | 0.51484900 |
| H | -3.73168400 | -1.84863800 | 0.48284000 |
| H | -3.44293500 | 2.30559700 | -0.58260400 |
| H | -0.96862500 | 2.12696500 | -0.56753600 |
| O | -5.10681300 | 0.44804900 | -0.09714700 |
| C | -5.96994900 | -0.65521400 | 0.18812200 |
| H | -6.98318800 | -0.26865800 | 0.09962000 |
| H | -5.82514500 | -1.46648500 | -0.53123200 |
| H | -5.81008200 | -1.02976600 | 1.20330100 |

**M062X**

| | | | |
|---|---|---|---|
| N | 0.45680800 | -0.06165300 | -0.02540000 |
| N | 1.20782200 | 1.05830400 | 0.25226700 |
| N | 2.43775600 | -0.70975400 | -0.14657000 |
| C | 1.16457400 | -1.17933500 | -0.28829700 |
| C | 2.43012900 | 0.62739500 | 0.16948300 |
| C | -0.96378000 | 0.04693400 | -0.02598100 |
| C | -1.74578600 | -1.06743300 | 0.24147700 |
| C | -3.13542200 | -0.97239000 | 0.22357100 |
| C | -3.74200100 | 0.25323200 | -0.04967100 |
| C | -2.94815100 | 1.37454900 | -0.30762100 |
| C | -1.56775700 | 1.27360800 | -0.30089600 |

| | | | |
|---|---|---|---|
| C | 3.65475400 | -1.49277400 | -0.38546400 |
| H | 3.78699900 | -1.59465700 | -1.46555500 |
| H | 3.49990000 | -2.48417000 | 0.03825200 |
| C | 3.67789800 | 1.41296600 | 0.41264100 |
| H | 3.55855600 | 2.41292100 | -0.00600100 |
| H | 3.80663700 | 1.52468800 | 1.49385000 |
| C | 4.88904600 | 0.68305700 | -0.17615500 |
| H | 4.87549900 | 0.76059800 | -1.26809300 |
| H | 5.80537300 | 1.15966000 | 0.17369600 |
| C | 4.85883400 | -0.79118700 | 0.23070800 |
| H | 4.81320500 | -0.86973000 | 1.32189400 |
| H | 5.76307700 | -1.30525300 | -0.09833800 |
| H | -1.26838300 | -2.01253900 | 0.46477700 |
| H | -3.72231500 | -1.85521800 | 0.43683200 |
| H | -3.43367700 | 2.31950500 | -0.51921600 |
| H | -0.95357000 | 2.13972400 | -0.50935700 |
| O | -5.08498800 | 0.45224200 | -0.08241300 |
| C | -5.92378100 | -0.66799900 | 0.15534100 |
| H | -6.94406700 | -0.30144100 | 0.07527100 |
| H | -5.75571800 | -1.44821600 | -0.59189400 |
| H | -5.75966200 | -1.07582500 | 1.15630200 |

**8b (B3LYP)**

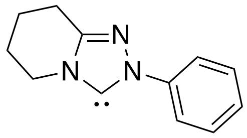

| | | | |
|---|---|---|---|
| N | 0.39609600 | -0.10808500 | -0.00207300 |
| N | -0.33087900 | 1.06772300 | 0.15847800 |
| N | -1.61535700 | -0.70065200 | -0.09895900 |
| C | -0.35489600 | -1.22326600 | -0.16383500 |
| C | -1.56802700 | 0.66540500 | 0.09558700 |
| C | 1.82076100 | -0.04669100 | 0.00037800 |
| C | 2.46741400 | 1.15829000 | -0.28259400 |
| C | 3.86048500 | 1.20831700 | -0.28275100 |
| C | 4.61105600 | 0.06575000 | -0.00885500 |
| C | 3.95570400 | -1.13375600 | 0.27291900 |
| C | 2.56464200 | -1.19504200 | 0.28496200 |
| C | -2.86112200 | -1.48415100 | -0.14999800 |
| H | -2.99364500 | -1.97451300 | 0.81845900 |
| H | -2.73779700 | -2.25828800 | -0.90697600 |
| C | -2.79434300 | 1.51935900 | 0.17154000 |
| H | -2.65445000 | 2.28905800 | 0.93320900 |
| H | -2.90853900 | 2.03906300 | -0.78654100 |
| C | -4.04338400 | 0.66574600 | 0.44639800 |
| H | -4.05976800 | 0.35775000 | 1.49758900 |
| H | -4.93771100 | 1.26841900 | 0.27571200 |
| C | -4.05354300 | -0.57661500 | -0.45264700 |

| | | | |
|---|---|---|---|
| H | -4.02956900 | -0.27109300 | -1.50458700 |
| H | -4.96971700 | -1.15299200 | -0.30575100 |
| H | 1.88294900 | 2.04151000 | -0.50038800 |
| H | 4.35737400 | 2.14600300 | -0.50403200 |
| H | 4.52797800 | -2.02741000 | 0.49439500 |
| H | 2.05149300 | -2.11950400 | 0.51241000 |
| H | 5.69390000 | 0.10900800 | -0.01223700 |

**M062X**

| | | | |
|---|---|---|---|
| N | 0.38970800 | -0.10824800 | -0.00137100 |
| N | -0.32636500 | 1.05716300 | 0.15895800 |
| N | -1.61062200 | -0.69868900 | -0.10101700 |
| C | -0.35457900 | -1.22293900 | -0.16627700 |
| C | -1.56072300 | 0.66162300 | 0.09533100 |
| C | 1.81242200 | -0.04737800 | -0.00002000 |
| C | 2.45163900 | 1.15437100 | -0.29242500 |
| C | 3.84192800 | 1.20552800 | -0.29180600 |
| C | 4.59089600 | 0.06768100 | -0.00803800 |
| C | 3.93923200 | -1.12856300 | 0.28234600 |
| C | 2.55091300 | -1.19201100 | 0.29351900 |
| C | -2.85253900 | -1.47740100 | -0.16470800 |
| H | -2.99316300 | -1.97016600 | 0.80049300 |
| H | -2.72424500 | -2.24334500 | -0.92826500 |
| C | -2.78374500 | 1.51611200 | 0.18174600 |

| | | | |
|---|---|---|---|
| H | -2.63333000 | 2.28217900 | 0.94320600 |
| H | -2.90875200 | 2.02851800 | -0.77729300 |
| C | -4.01697900 | 0.65316500 | 0.46653800 |
| H | -4.00238000 | 0.31397400 | 1.50734400 |
| H | -4.91803200 | 1.25237000 | 0.33140700 |
| C | -4.03106200 | -0.55935800 | -0.46491500 |
| H | -3.98264500 | -0.22347700 | -1.50592100 |
| H | -4.95269300 | -1.13087500 | -0.34645300 |
| H | 1.86259800 | 2.03287800 | -0.51885700 |
| H | 4.33888700 | 2.14095000 | -0.51969800 |
| H | 4.51276900 | -2.01866900 | 0.51232900 |
| H | 2.03440400 | -2.11305000 | 0.52834300 |
| H | 5.67311000 | 0.11215700 | -0.01036900 |

**8c (B3LYP)**

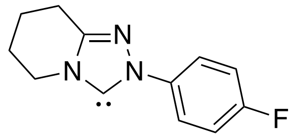


| | | | |
|---|---|---|---|
| N | 0.02152000 | -0.11655300 | -0.00789300 |
| N | 0.74161500 | 1.05578100 | 0.19977200 |
| N | 2.03514200 | -0.69690000 | -0.11512100 |
| C | 0.77737800 | -1.22058300 | -0.21491400 |
| C | 1.98083400 | 0.66117500 | 0.12731900 |
| C | -1.40209500 | -0.06236100 | -0.00172700 |
| C | -2.14046800 | -1.22506700 | 0.23350100 |
| C | -3.53150600 | -1.17962400 | 0.22651200 |
| C | -4.15631700 | 0.03562700 | -0.00364300 |
| C | -3.44737700 | 1.20296200 | -0.22982000 |
| C | -2.05511300 | 1.15014400 | -0.23188000 |
| C | 3.28430800 | -1.44614100 | -0.33112400 |
| H | 3.40582400 | -1.60025400 | -1.40689000 |
| H | 3.17374900 | -2.42234200 | 0.14027300 |
| C | 3.20271700 | 1.50200900 | 0.32302900 |
| H | 3.05313400 | 2.47463900 | -0.14992800 |
| H | 3.32224600 | 1.68671300 | 1.39657500 |
| C | 4.45295900 | 0.79046100 | -0.21987700 |
| H | 4.46069100 | 0.83586200 | -1.31442500 |
| H | 5.34604100 | 1.31323000 | 0.12833500 |
| C | 4.47714700 | -0.67480800 | 0.23342000 |

| | | | |
|---|---|---|---|
| H | 4.46452500 | -0.72250300 | 1.32795300 |
| H | 5.39322700 | -1.16777300 | -0.09966200 |
| F | -5.51853000 | 0.08339100 | -0.00368700 |
| H | -1.62548900 | -2.15717300 | 0.42023900 |
| H | -4.12057000 | -2.06983700 | 0.40755600 |
| H | -3.97129000 | 2.13373800 | -0.40697300 |
| H | -1.47807000 | 2.04612700 | -0.41195700 |

**M062X**

| | | | |
|---|---|---|---|
| N | -0.02548600 | -0.11862100 | 0.00391700 |
| N | -0.73415700 | 1.03909100 | -0.22622000 |
| N | -2.02806500 | -0.69358600 | 0.12651400 |
| C | -0.77460100 | -1.21744000 | 0.23671600 |
| C | -1.97080700 | 0.65325600 | -0.14505900 |
| C | 1.39626000 | -0.06450200 | -0.00003300 |
| C | 2.12985000 | -1.21993100 | -0.25906400 |
| C | 3.51797100 | -1.17185500 | -0.25013500 |
| C | 4.13867200 | 0.03767600 | 0.00611000 |
| C | 3.42955500 | 1.19745000 | 0.25611700 |
| C | 2.04057000 | 1.14249400 | 0.25614600 |
| C | -3.27360600 | -1.43466700 | 0.35424400 |
| H | -3.39918000 | -1.56319000 | 1.43225500 |
| H | -3.16138800 | -2.41828400 | -0.09976500 |
| C | -3.18883500 | 1.49359200 | -0.35154900 |

| | | | |
|---|---|---|---|
| H | -3.02709000 | 2.47508000 | 0.09554400 |
| H | -3.32156500 | 1.64272500 | -1.42770100 |
| C | -4.42252500 | 0.79326100 | 0.22599600 |
| H | -4.39617500 | 0.83525600 | 1.31962900 |
| H | -5.32208900 | 1.31696800 | -0.09902500 |
| C | -4.45421300 | -0.66719000 | -0.22749700 |
| H | -4.42213700 | -0.71328200 | -1.32093500 |
| H | -5.37480300 | -1.15543500 | 0.09540700 |
| F | 5.48785300 | 0.08732400 | 0.00805600 |
| H | 1.61207500 | -2.14645900 | -0.46711900 |
| H | 4.11244300 | -2.05395200 | -0.45143700 |
| H | 3.95593400 | 2.12251700 | 0.45336000 |
| H | 1.45750500 | 2.03108700 | 0.45585800 |

**8i (B3LYP)**

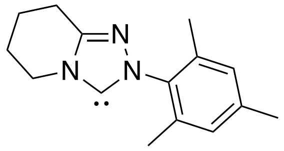


| | | | |
|---|---|---|---|
| N | -0.20046000 | 0.01692800 | -0.11398000 |
| N | -0.91734600 | -0.13726700 | 1.07243200 |
| N | -2.21665100 | 0.09814100 | -0.68901500 |
| C | -0.95434700 | 0.16007200 | -1.22059900 |
| C | -2.15864000 | -0.08305300 | 0.67732300 |
| C | 1.23608700 | 0.00950700 | -0.06385900 |
| C | 1.91916200 | 1.22750300 | 0.05046800 |
| C | 3.31606400 | 1.19314200 | 0.10348500 |
| C | 4.02708800 | -0.00796400 | 0.04956200 |
| C | 3.30692200 | -1.20175200 | -0.05495300 |
| C | 1.91134100 | -1.21864300 | -0.11367100 |
| C | -3.46674500 | 0.12946200 | -1.46539500 |
| H | -3.59567100 | -0.84529300 | -1.94450300 |
| H | -3.35532600 | 0.87837500 | -2.24930900 |
| C | -3.37999900 | -0.15186800 | 1.54057800 |
| H | -3.23144500 | -0.90076700 | 2.32116700 |
| H | -3.49826200 | 0.81331300 | 2.04601400 |
| C | -4.63224000 | -0.44834200 | 0.69907700 |
| H | -4.64199100 | -1.50374900 | 0.40518500 |
| H | -5.52457600 | -0.27692500 | 1.30470600 |
| C | -4.65703500 | 0.43369100 | -0.55527100 |

| | | | |
|---|---|---|---|
| H | -4.64010300 | 1.48972600 | -0.26345600 |
| H | -5.57538700 | 0.27188600 | -1.12433900 |
| C | 1.16080500 | -2.52223900 | -0.22891300 |
| H | 0.48744500 | -2.67063000 | 0.61962400 |
| H | 0.54579200 | -2.54610000 | -1.13290500 |
| H | 1.85465200 | -3.36311400 | -0.26530500 |
| C | 1.17948400 | 2.54094500 | 0.11426800 |
| H | 0.62119300 | 2.72531200 | -0.80785100 |
| H | 0.45560600 | 2.55196500 | 0.93351000 |
| H | 1.87600500 | 3.36721700 | 0.26247500 |
| C | 5.53662300 | -0.01870400 | 0.07924300 |
| H | 5.91716400 | -0.87516900 | 0.64085200 |
| H | 5.94487700 | -0.08784000 | -0.93482500 |
| H | 5.93240100 | 0.89292400 | 0.53140700 |
| H | 3.84285000 | -2.14525100 | -0.09090200 |
| H | 3.85854300 | 2.12897000 | 0.19433600 |

**M062X**

| | | | |
|---|---|---|---|
| N | -0.19803800 | 0.02127600 | -0.11398400 |
| N | -0.90168400 | -0.18831100 | 1.05318400 |
| N | -2.20298200 | 0.12935500 | -0.68146300 |
| C | -0.94591800 | 0.21843900 | -1.21245200 |
| C | -2.14109900 | -0.11557500 | 0.66925300 |
| C | 1.23408900 | 0.01299900 | -0.06562600 |

| | | | |
|---|---|---|---|
| C | 1.90926700 | 1.22521000 | 0.07302000 |
| C | 3.30326300 | 1.19136300 | 0.12658900 |
| C | 4.00807300 | -0.00871400 | 0.04989200 |
| C | 3.29182600 | -1.20015600 | -0.07922800 |
| C | 1.90033800 | -1.21238900 | -0.13942400 |
| C | -3.45035300 | 0.20520600 | -1.44896500 |
| H | -3.58992100 | -0.74711900 | -1.96675400 |
| H | -3.33341300 | 0.98962000 | -2.19551200 |
| C | -3.35813600 | -0.23075100 | 1.53038100 |
| H | -3.20025100 | -1.01361100 | 2.27305600 |
| H | -3.48311100 | 0.71238600 | 2.07143400 |
| C | -4.59719800 | -0.49440800 | 0.66903200 |
| H | -4.58078800 | -1.52440900 | 0.29825100 |
| H | -5.49428500 | -0.38205700 | 1.27897100 |
| C | -4.62417100 | 0.47279500 | -0.51511800 |
| H | -4.57733300 | 1.50347600 | -0.14851200 |
| H | -5.54946800 | 0.36755700 | -1.08340900 |
| C | 1.12688300 | -2.49529000 | -0.28374900 |
| H | 0.43718200 | -2.63085200 | 0.55256300 |
| H | 0.52690000 | -2.48276500 | -1.19719300 |
| H | 1.80205000 | -3.34965400 | -0.32176700 |
| C | 1.14958500 | 2.52177000 | 0.16008500 |
| H | 0.62944200 | 2.72800800 | -0.77851100 |
| H | 0.39155800 | 2.47651300 | 0.94565600 |
| H | 1.82533900 | 3.34969300 | 0.37279900 |

| | | | |
|---|---|---|---|
| C | 5.51452000 | -0.02605900 | 0.08677500 |
| H | 5.88102700 | -0.85064500 | 0.70070000 |
| H | 5.92113200 | -0.15945200 | -0.91936500 |
| H | 5.90989100 | 0.90821700 | 0.48684400 |
| H | 3.82973500 | -2.14202100 | -0.13329200 |
| H | 3.84744300 | 2.12422900 | 0.23708700 |

**8k (B3LYP)**

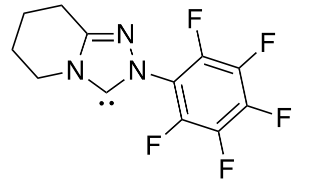


| | | | |
|---|---|---|---|
| N | -0.64330200 | 0.05129900 | -0.09935900 |
| N | -1.35725100 | -0.48597400 | 0.97424300 |
| N | -2.64900600 | 0.32640600 | -0.61912300 |
| C | -1.39462500 | 0.55917800 | -1.10391100 |
| C | -2.59441200 | -0.29752700 | 0.61443500 |
| C | 0.76949700 | 0.02884400 | -0.05962700 |
| C | 1.50245300 | 1.20174200 | 0.11441200 |
| C | 2.89035000 | 1.18293100 | 0.15022800 |
| C | 3.56637500 | -0.02356800 | 0.02670800 |
| C | 2.85470000 | -1.20491600 | -0.13817400 |
| C | 1.46813000 | -1.17139600 | -0.18354200 |
| C | -3.90114600 | 0.61353600 | -1.34248300 |
| H | -4.02752000 | -0.15107000 | -2.11356700 |
| H | -3.78623200 | 1.57797700 | -1.83597700 |
| C | -3.81728900 | -0.64183500 | 1.40489100 |
| H | -3.67001100 | -1.60501400 | 1.89701400 |
| H | -3.93087000 | 0.10602100 | 2.19760500 |
| C | -5.06899600 | -0.64175300 | 0.51240800 |
| H | -5.08243300 | -1.54266600 | -0.11048700 |
| H | -5.96010400 | -0.67505100 | 1.14232500 |

| | | | |
|---|---|---|---|
| C | -5.09054400 | 0.60369400 | -0.38260200 |
| H | -5.07283600 | 1.50563300 | 0.23902900 |
| H | -6.00770600 | 0.63906600 | -0.97470600 |
| F | 4.90003800 | -0.04839200 | 0.06543600 |
| F | 3.57746100 | 2.31825900 | 0.31612900 |
| F | 0.87519900 | 2.37291700 | 0.25915200 |
| F | 0.80341300 | -2.31688500 | -0.36561900 |
| F | 3.50807400 | -2.36485100 | -0.26488000 |

**M062X**

| | | | |
|---|---|---|---|
| N | -0.64310400 | 0.07830000 | -0.07883000 |
| N | -1.34857400 | -0.60298400 | 0.89381700 |
| N | -2.63627500 | 0.41918500 | -0.56211800 |
| C | -1.38639800 | 0.72298700 | -1.00511000 |
| C | -2.58224300 | -0.37147900 | 0.56583600 |
| C | 0.76577300 | 0.04386800 | -0.04591900 |
| C | 1.50630600 | 1.20400300 | 0.14198200 |
| C | 2.89052100 | 1.16809000 | 0.16651500 |
| C | 3.54989200 | -0.04039100 | 0.01856300 |
| C | 2.82705900 | -1.20834600 | -0.16048800 |
| C | 1.44468700 | -1.15930900 | -0.19644800 |
| C | -3.88257000 | 0.80443500 | -1.23732300 |
| H | -4.00999800 | 0.15296700 | -2.10510700 |
| H | -3.76530900 | 1.82900500 | -1.58679200 |

| | | | |
|---|---|---|---|
| C | -3.80378400 | -0.83252100 | 1.29256300 |
| H | -3.64540300 | -1.85182600 | 1.64593000 |
| H | -3.93276300 | -0.19669800 | 2.17394200 |
| C | -5.03580100 | -0.71773300 | 0.38997100 |
| H | -5.01092000 | -1.50120100 | -0.37424800 |
| H | -5.93611900 | -0.87141400 | 0.98558900 |
| C | -5.06208700 | 0.65425000 | -0.28515700 |
| H | -5.02474000 | 1.44035700 | 0.47599200 |
| H | -5.98273300 | 0.79101500 | -0.85404400 |
| F | 4.87396900 | -0.07976600 | 0.04841200 |
| F | 3.58699100 | 2.28400700 | 0.34642200 |
| F | 0.89746800 | 2.36989700 | 0.31433900 |
| F | 0.76917500 | -2.28369800 | -0.39739900 |
| F | 3.46301100 | -2.36386500 | -0.31191400 |

**9a (B3LYP)**

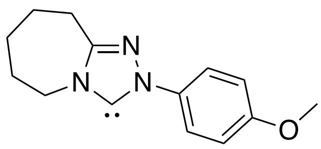

| | | | |
|---|---|---|---|
| N | 0.15038300 | -0.15706700 | -0.20786200 |
| N | 0.82135800 | 1.05070200 | -0.07040300 |
| N | 2.17867900 | -0.62727700 | -0.49985600 |
| C | 0.94464400 | -1.21938500 | -0.47461000 |
| C | 2.07121700 | 0.72865600 | -0.25908000 |
| C | -1.26993700 | -0.17965800 | -0.07514100 |
| C | -2.02150700 | 0.95374200 | -0.37011700 |
| C | -3.41226600 | 0.93308900 | -0.24915200 |
| C | -4.05853300 | -0.23548800 | 0.16170000 |
| C | -3.29733100 | -1.37450000 | 0.45817700 |
| C | -1.91550800 | -1.34624900 | 0.34834800 |
| C | 3.43061600 | -1.34922200 | -0.76260700 |
| H | 3.85467500 | -0.98547000 | -1.70396800 |
| H | 3.14086000 | -2.38838000 | -0.90969200 |
| C | 3.21522000 | 1.68564400 | -0.20283600 |
| H | 3.67894100 | 1.75146000 | -1.19477100 |
| H | 2.79730400 | 2.66950900 | 0.01635100 |
| C | 4.29184300 | 1.32442300 | 0.84141600 |
| H | 3.81152700 | 1.15516700 | 1.81177200 |
| H | 4.93689300 | 2.19947500 | 0.96281400 |
| C | 4.45572000 | -1.23273800 | 0.36885300 |

| | | | |
|---|---|---|---|
| H | 3.96797300 | -1.48048000 | 1.31845700 |
| H | 5.20921900 | -2.00722100 | 0.19408900 |
| C | 5.17165900 | 0.12208000 | 0.47273000 |
| H | 5.96245100 | 0.03294300 | 1.22437700 |
| H | 5.67727800 | 0.32951200 | -0.47905200 |
| H | -1.33074800 | -2.22397100 | 0.58921100 |
| H | -3.80455400 | -2.27413200 | 0.78591500 |
| H | -3.97022200 | 1.82851200 | -0.48553300 |
| H | -1.52479200 | 1.85759000 | -0.69646000 |
| O | -5.40946500 | -0.36408600 | 0.30660700 |
| C | -6.23737700 | 0.76643100 | 0.02378400 |
| H | -7.25856600 | 0.43865800 | 0.20723800 |
| H | -5.99951500 | 1.60536600 | 0.68419600 |
| H | -6.13671700 | 1.07870000 | -1.01974800 |

**M062X**

| | | | |
|---|---|---|---|
| N | 0.15673800 | -0.15847400 | -0.21858100 |
| N | 0.81653700 | 1.04315300 | -0.11369600 |
| N | 2.17186900 | -0.63188500 | -0.50769400 |
| C | 0.94499200 | -1.22698200 | -0.46330700 |
| C | 2.06217200 | 0.72225600 | -0.29758500 |
| C | -1.26146000 | -0.18171900 | -0.08499300 |
| C | -2.00531200 | 0.96050800 | -0.34032300 |
| C | -3.39410800 | 0.93902900 | -0.21721200 |

| | | | |
|---|---|---|---|
| C | -4.03753300 | -0.23953500 | 0.15470700 |
| C | -3.28111700 | -1.38807200 | 0.41075200 |
| C | -1.90295000 | -1.36021900 | 0.29937900 |
| C | 3.43005300 | -1.33839900 | -0.74404800 |
| H | 3.88077800 | -0.95048700 | -1.66274200 |
| H | 3.15449100 | -2.37750200 | -0.91534700 |
| C | 3.21279600 | 1.66729600 | -0.25967300 |
| H | 3.71086700 | 1.66549700 | -1.23592100 |
| H | 2.80000700 | 2.66478100 | -0.10655600 |
| C | 4.23464400 | 1.33326100 | 0.83893600 |
| H | 3.70814500 | 1.17561000 | 1.78614400 |
| H | 4.87381300 | 2.20882900 | 0.97475800 |
| C | 4.40558700 | -1.21985100 | 0.42438200 |
| H | 3.87059700 | -1.43993500 | 1.35431800 |
| H | 5.15775600 | -2.00268300 | 0.29663300 |
| C | 5.12393400 | 0.12813700 | 0.52604900 |
| H | 5.88759200 | 0.05127000 | 1.30454100 |
| H | 5.65615700 | 0.31538900 | -0.41434100 |
| H | -1.31585100 | -2.24438400 | 0.50995400 |
| H | -3.79461800 | -2.29437500 | 0.70824600 |
| H | -3.95045700 | 1.84324900 | -0.42234500 |
| H | -1.50361700 | 1.87207200 | -0.63678300 |
| O | -5.38219100 | -0.36713100 | 0.29803500 |
| C | -6.18080700 | 0.78408000 | 0.07040000 |
| H | -7.20885800 | 0.47695500 | 0.24545800 |

| | | | |
|---|---|---|---|
| H | -5.91579300 | 1.58704700 | 0.76352300 |
| H | -6.07484800 | 1.13743200 | -0.95880100 |

**9b (B3LYP)**

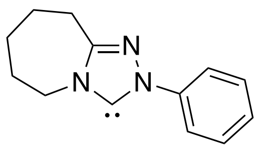


| | | | |
|---|---|---|---|
| N | -0.69074900 | -0.09366900 | -0.17724200 |
| N | 0.03361700 | 1.09007000 | -0.12382600 |
| N | 1.31148300 | -0.66978000 | -0.46092300 |
| C | 0.05654500 | -1.20505800 | -0.38393800 |
| C | 1.26505200 | 0.70308900 | -0.30386200 |
| C | -2.10812800 | -0.04819100 | -0.02712400 |
| C | -2.78955600 | 1.15935900 | -0.19512700 |
| C | -4.17584700 | 1.19343300 | -0.05194800 |
| C | -4.88673100 | 0.03328800 | 0.25159200 |
| C | -4.19689500 | -1.16851000 | 0.41774200 |
| C | -2.81159700 | -1.21491800 | 0.28557100 |
| C | 2.52890200 | -1.46066800 | -0.68698500 |
| H | 2.95825500 | -1.17522300 | -1.65251100 |
| H | 2.19292400 | -2.49327300 | -0.76582400 |
| C | 2.44981900 | 1.61018000 | -0.31977600 |
| H | 2.90418600 | 1.59052100 | -1.31790900 |
| H | 2.07745700 | 2.62357200 | -0.16166500 |
| C | 3.52095800 | 1.26947200 | 0.73695000 |
| H | 3.04414800 | 1.18334800 | 1.71983400 |
| H | 4.20415200 | 2.12159200 | 0.79663800 |
| C | 3.56906400 | -1.31730600 | 0.42750600 |

| | | | |
|---|---|---|---|
| H | 3.08023900 | -1.48236600 | 1.39437000 |
| H | 4.28631700 | -2.13379000 | 0.29745600 |
| C | 4.34424000 | 0.00869200 | 0.43936200 |
| H | 5.13758900 | -0.06723200 | 1.18971500 |
| H | 4.84908600 | 0.13283600 | -0.52718800 |
| H | -2.27172000 | -2.14199700 | 0.42152800 |
| H | -4.73689400 | -2.07685700 | 0.65964000 |
| H | -4.69909300 | 2.13360600 | -0.18414600 |
| H | -2.23747600 | 2.05730400 | -0.43531000 |
| H | -5.96459600 | 0.06445300 | 0.35951300 |

**M062X**

| | | | |
|---|---|---|---|
| N | -0.67908600 | -0.09395700 | -0.18371600 |
| N | 0.03383700 | 1.08152700 | -0.13716900 |
| N | 1.30968000 | -0.66544700 | -0.48166400 |
| C | 0.06148900 | -1.20514300 | -0.39522200 |
| C | 1.26073200 | 0.70033900 | -0.32418400 |
| C | -2.09383400 | -0.04973900 | -0.02864500 |
| C | -2.76884000 | 1.15725200 | -0.19202300 |
| C | -4.15174200 | 1.19236000 | -0.04270000 |
| C | -4.86010300 | 0.03420600 | 0.26148600 |
| C | -4.17322300 | -1.16676600 | 0.42198500 |
| C | -2.79124800 | -1.21554800 | 0.28344500 |
| C | 2.53302400 | -1.43539600 | -0.70612700 |

| | | | |
|---|---|---|---|
| H | 2.98486400 | -1.10550800 | -1.64656900 |
| H | 2.20951500 | -2.46737700 | -0.82949000 |
| C | 2.45037100 | 1.59614400 | -0.33889900 |
| H | 2.93309700 | 1.53489200 | -1.32083200 |
| H | 2.08196800 | 2.61521600 | -0.21906400 |
| C | 3.47277200 | 1.26071400 | 0.75868600 |
| H | 2.95372900 | 1.16063200 | 1.71775800 |
| H | 4.14961300 | 2.11301500 | 0.85283200 |
| C | 3.53144500 | -1.31131300 | 0.44210000 |
| H | 3.00390500 | -1.47264800 | 1.38819000 |
| H | 4.24878800 | -2.12901200 | 0.33422700 |
| C | 4.30628000 | 0.00848900 | 0.47972900 |
| H | 5.07916000 | -0.07039200 | 1.24882600 |
| H | 4.82939600 | 0.13721800 | -0.47543900 |
| H | -2.24945800 | -2.14233700 | 0.41527600 |
| H | -4.71389400 | -2.07396400 | 0.66432400 |
| H | -4.67442000 | 2.13272800 | -0.17115600 |
| H | -2.21314200 | 2.05299800 | -0.43357100 |
| H | -5.93686500 | 0.06607800 | 0.37474400 |

**9c (B3LYP)**

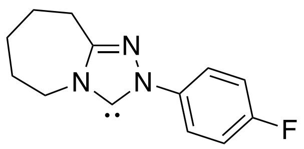


| | | | |
|---|---|---|---|
| N | -0.28053300 | -0.09690200 | -0.20587900 |
| N | 0.43844900 | 1.08835000 | -0.12588600 |
| N | 1.72609100 | -0.66328500 | -0.47009400 |
| C | 0.47171700 | -1.20333900 | -0.41811800 |
| C | 1.67351700 | 0.70715500 | -0.29493100 |
| C | -1.69859900 | -0.05705900 | -0.07484400 |
| C | -2.38501700 | 1.14136200 | -0.28098700 |
| C | -3.77243400 | 1.17979100 | -0.15844200 |
| C | -4.44409100 | 0.01243600 | 0.16141400 |
| C | -3.78583100 | -1.18894200 | 0.37015600 |
| C | -2.39902000 | -1.21989600 | 0.25677000 |
| C | 2.94870700 | -1.44760300 | -0.69215700 |
| H | 3.38820700 | -1.14803100 | -1.64877700 |
| H | 2.61680500 | -2.48001200 | -0.78864300 |
| C | 2.85526200 | 1.61815400 | -0.28319200 |
| H | 3.32147100 | 1.61460900 | -1.27602700 |
| H | 2.47747400 | 2.62789000 | -0.11492600 |
| C | 3.91488900 | 1.26566600 | 0.78117000 |
| H | 3.42687600 | 1.16403800 | 1.75703900 |
| H | 4.59439800 | 2.11905800 | 0.86112000 |
| C | 3.97530700 | -1.31615300 | 0.43624100 |

| | | | |
|---|---|---|---|
| H | 3.47604300 | -1.49625300 | 1.39504200 |
| H | 4.69670400 | -2.12846800 | 0.30312100 |
| C | 4.74582200 | 0.01196800 | 0.47531400 |
| H | 5.53087300 | -0.07185800 | 1.23350000 |
| H | 5.26115000 | 0.15128400 | -0.48359400 |
| F | -5.80184400 | 0.04585600 | 0.27847600 |
| H | -1.85815100 | -2.14097400 | 0.42352800 |
| H | -4.34528500 | -2.07986800 | 0.62620500 |
| H | -4.32154100 | 2.09964300 | -0.31519300 |
| H | -1.83783700 | 2.03785500 | -0.53602100 |

**M062X**

| | | | |
|---|---|---|---|
| N | -0.27113100 | -0.09643600 | -0.21442900 |
| N | 0.43622000 | 1.08055400 | -0.13984800 |
| N | 1.72239300 | -0.65757700 | -0.49228200 |
| C | 0.47461700 | -1.20238100 | -0.43189700 |
| C | 1.66714500 | 0.70549800 | -0.31515600 |
| C | -1.68703800 | -0.05847200 | -0.07925500 |
| C | -2.36772800 | 1.13883700 | -0.28226300 |
| C | -3.75156400 | 1.17774100 | -0.15385100 |
| C | -4.42082500 | 0.01224100 | 0.16800300 |
| C | -3.76372100 | -1.18804900 | 0.37210500 |
| C | -2.38045200 | -1.22060400 | 0.25286500 |
| C | 2.95120700 | -1.42028500 | -0.71244500 |

| | | | |
|---|---|---|---|
| H | 3.41405300 | -1.07422900 | -1.64163400 |
| H | 2.63277900 | -2.45119000 | -0.85637400 |
| C | 2.85406800 | 1.60483300 | -0.29978100 |
| H | 3.35027400 | 1.55996000 | -1.27586200 |
| H | 2.48093000 | 2.62091000 | -0.16962300 |
| C | 3.86235400 | 1.25529600 | 0.80633400 |
| H | 3.33084200 | 1.13883100 | 1.75666300 |
| H | 4.53514900 | 2.10800900 | 0.92308000 |
| C | 3.93394000 | -1.31122200 | 0.45071700 |
| H | 3.39459500 | -1.48932200 | 1.38710000 |
| H | 4.65531600 | -2.12487300 | 0.33928800 |
| C | 4.70375500 | 0.01020700 | 0.51918100 |
| H | 5.46665700 | -0.07804900 | 1.29714500 |
| H | 5.23906600 | 0.15550000 | -0.42679200 |
| F | -5.76476700 | 0.04606800 | 0.29162500 |
| H | -1.83624600 | -2.14057800 | 0.41686000 |
| H | -4.32677900 | -2.07607300 | 0.62991700 |
| H | -4.30412100 | 2.09588200 | -0.30706500 |
| H | -1.81652200 | 2.03298300 | -0.53876800 |

**9k (B3LYP)**

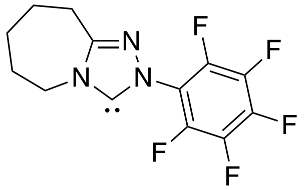


| | | | |
|---|---|---|---|
| N | 0.36854000 | -0.15558200 | 0.19776200 |
| N | 1.07756100 | -0.71780600 | -0.86271500 |
| N | 2.36936700 | -0.08092300 | 0.80890600 |
| C | 1.12003900 | 0.25452100 | 1.24648900 |
| C | 2.31177700 | -0.65918400 | -0.44883700 |
| C | -1.03777300 | -0.04749000 | 0.10168400 |
| C | -1.84762400 | -1.17899100 | 0.18887800 |
| C | -3.22866800 | -1.08525000 | 0.08985200 |
| C | -3.82162500 | 0.15739400 | -0.09363900 |
| C | -3.03359200 | 1.29734800 | -0.18166300 |
| C | -1.65231300 | 1.18858700 | -0.09177000 |
| C | 3.59654200 | 0.14258600 | 1.58864200 |
| H | 4.03434500 | -0.82953700 | 1.83520700 |
| H | 3.26779900 | 0.60430500 | 2.51809000 |
| C | 3.49281400 | -1.12913000 | -1.23123900 |
| H | 3.95996800 | -1.96999700 | -0.70447900 |
| H | 3.11283500 | -1.51941000 | -2.17658000 |
| C | 4.54734600 | -0.03539000 | -1.49516600 |
| H | 4.05471300 | 0.84746600 | -1.91772300 |
| H | 5.22248000 | -0.41349400 | -2.26818400 |

| | | | |
|---|---|---|---|
| C | 4.62197800 | 1.03161000 | 0.87915100 |
| H | 4.12297100 | 1.94246500 | 0.52963500 |
| H | 5.34592600 | 1.34392200 | 1.63810000 |
| C | 5.38575400 | 0.36364300 | -0.27325100 |
| H | 6.16982300 | 1.05070000 | -0.60669800 |
| H | 5.90163200 | -0.52549900 | 0.11092000 |
| F | -3.60747100 | 2.49127900 | -0.36501600 |
| F | -0.91459900 | 2.29752500 | -0.20165600 |
| F | -5.14915700 | 0.25601500 | -0.18435100 |
| F | -3.99017300 | -2.18066300 | 0.18209000 |
| F | -1.29734300 | -2.38107500 | 0.38662700 |

**M062X**

| | | | |
|---|---|---|---|
| N | 0.37328300 | -0.14442400 | 0.24494300 |
| N | 1.06790200 | -0.88321500 | -0.69081500 |
| N | 2.36385000 | 0.03485600 | 0.82581200 |
| C | 1.12322900 | 0.44787700 | 1.20233000 |
| C | 2.29979500 | -0.75444600 | -0.30285200 |
| C | -1.02727300 | -0.04188000 | 0.12659400 |
| C | -1.83176700 | -1.17055000 | 0.22837100 |
| C | -3.20660600 | -1.08089600 | 0.09916900 |
| C | -3.79552300 | 0.15281100 | -0.12462800 |
| C | -3.01023400 | 1.28874600 | -0.22416000 |
| C | -1.63440200 | 1.18578200 | -0.10707100 |

| | | | |
|---|---|---|---|
| C | 3.60236000 | 0.39242500 | 1.51971100 |
| H | 4.07758400 | -0.52587100 | 1.87718300 |
| H | 3.29367700 | 0.97346000 | 2.38676300 |
| C | 3.48473400 | -1.33875900 | -0.99030100 |
| H | 3.99772400 | -2.02168600 | -0.30424900 |
| H | 3.10782600 | -1.93384400 | -1.82231700 |
| C | 4.47110400 | -0.27276600 | -1.49474800 |
| H | 3.91877200 | 0.50078200 | -2.03844600 |
| H | 5.13653400 | -0.75234500 | -2.21602100 |
| C | 4.56155700 | 1.19190400 | 0.64237200 |
| H | 4.00483500 | 1.99975200 | 0.15571200 |
| H | 5.28807500 | 1.66303100 | 1.30942000 |
| C | 5.32439600 | 0.36502800 | -0.39644800 |
| H | 6.06801200 | 1.01164900 | -0.86936200 |
| H | 5.88229300 | -0.42413300 | 0.12163100 |
| F | -3.57796300 | 2.46767100 | -0.44962200 |
| F | -0.90155300 | 2.28364200 | -0.23716500 |
| F | -5.11168500 | 0.24559500 | -0.24491000 |
| F | -3.96370400 | -2.16641300 | 0.20303400 |
| F | -1.28718200 | -2.35637900 | 0.46978200 |

## S2.4 Summary of Bond Lengths, Bond Angles, and Dihedral Angles from Computational Analysis

### S2.4.1 Summary for Triazolium Computational Analysis

Table S21. Computational structural data for individual triazolium salts **7a-k** (n=1) obtained from DFT optimized structures (B3LYP, n=1).

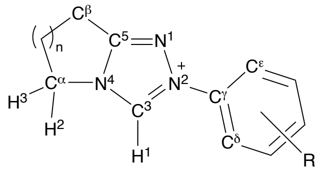


| Backbone Bond Length | **7a** | **7b** | **7c** | **7d** | **7e** | **7f** | **7g** | **7h** | **7i** | **7j** | **7k** |
|---|---|---|---|---|---|---|---|---|---|---|---|
| $N^1N^2$ | 1.38 | 1.38 | 1.38 | 1.38 | 1.38 | 1.38 | 1.38 | 1.38 | 1.38 | 1.38 | 1.38 |
| $N^2C^3$ | 1.33 | 1.33 | 1.33 | 1.34 | 1.34 | 1.34 | 1.33 | 1.33 | 1.33 | 1.34 | 1.34 |
| $C^3N^4$ | 1.33 | 1.33 | 1.33 | 1.33 | 1.33 | 1.33 | 1.33 | 1.33 | 1.33 | 1.33 | 1.33 |
| $N^4C^5$ | 1.37 | 1.37 | 1.37 | 1.37 | 1.37 | 1.37 | 1.37 | 1.37 | 1.37 | 1.37 | 1.37 |
| $C^5N^1$ | 1.31 | 1.31 | 1.31 | 1.31 | 1.31 | 1.31 | 1.30 | 1.31 | 1.31 | 1.30 | 1.30 |
| $C^5C^\beta$ | 1.49 | 1.49 | 1.49 | 1.49 | 1.49 | 1.49 | 1.49 | 1.49 | 1.49 | 1.49 | 1.49 |
| $N^4C^\alpha$ | 1.48 | 1.48 | 1.48 | 1.48 | 1.48 | 1.48 | 1.48 | 1.48 | 1.48 | 1.48 | 1.48 |
| $N^2C^\gamma$ | 1.43 | 1.44 | 1.43 | 1.43 | 1.43 | 1.43 | 1.43 | 1.45 | 1.45 | 1.43 | 1.42 |
| $C^\gamma C^\delta$ | 1.39 | 1.39 | 1.39 | 1.39 | 1.39 | 1.39 | 1.40 | 1.40 | 1.40 | 1.40 | 1.39 |
| $C^\gamma C^\varepsilon$ | 1.40 | 1.39 | 1.39 | 1.39 | 1.39 | 1.39 | 1.40 | 1.41 | 1.40 | 1.40 | 1.39 |
| C-H Bond Length | | | | | | | | | | | |
| $C^3H^1$ | 1.08 | 1.08 | 1.08 | 1.08 | 1.08 | 1.08 | 1.08 | 1.08 | 1.08 | 1.08 | 1.08 |
| $C^\alpha H^2$ | 1.09 | 1.09 | 1.09 | 1.09 | 1.09 | 1.09 | 1.09 | 1.09 | 1.09 | 1.09 | 1.09 |
| $C^\alpha H^3$ | 1.09 | 1.09 | 1.09 | 1.09 | 1.09 | 1.09 | 1.09 | 1.09 | 1.09 | 1.09 | 1.09 |
| H-H Distance | | | | | | | | | | | |
| $H^1H^2$ | 3.16 | 3.14 | 3.16 | 3.14 | 3.14 | 3.13 | 3.15 | 3.15 | 3.15 | 3.15 | 3.14 |
| $H^1H^3$ | 3.37 | 3.39 | 3.37 | 3.39 | 3.39 | 3.39 | 3.39 | 3.39 | 3.38 | 3.39 | 3.38 |
| Bond Angles | | | | | | | | | | | |
| $C^5N^1N^2$ | 103.8 | 103.8 | 103.7 | 103.8 | 103.8 | 103.8 | 103.7 | 103.8 | 103.8 | 103.5 | 103.4 |
| $N^1N^2C^3$ | 111.5 | 111.5 | 111.5 | 111.5 | 111.6 | 111.5 | 111.5 | 111.4 | 111.4 | 111.7 | 111.8 |
| $N^2C^3N^4$ | 106.1 | 106.0 | 106.0 | 106.0 | 106.0 | 106.0 | 106.2 | 106.3 | 106.3 | 105.9 | 105.8 |
| $C^3N^4C^5$ | 107.3 | 107.4 | 107.4 | 107.4 | 107.4 | 107.5 | 107.3 | 107.3 | 107.3 | 107.6 | 107.7 |
| $N^4C^5N^1$ | 111.3 | 111.3 | 111.3 | 111.3 | 111.2 | 111.2 | 111.3 | 111.2 | 111.3 | 111.3 | 111.4 |
| $C^\beta C^5N^4$ | 111.1 | 111.0 | 111.1 | 111.0 | 111.0 | 111.0 | 110.9 | 111.0 | 111.0 | 111.0 | 110.9 |
| $C^5N^4C^\alpha$ | 113.5 | 113.5 | 113.5 | 113.4 | 113.4 | 113.4 | 113.4 | 113.4 | 113.5 | 113.3 | 113.3 |
| Torsion Angles | | | | | | | | | | | |
| $H^1C^3{*}C^\alpha H^2$ | 43.1 | 40.0 | 42.9 | 39.9 | 40.1 | 39.6 | 41.3 | 41.4 | 41.4 | 41.2 | 41.1 |
| $H^1C^3{*}C^\alpha H^3$ | 68.7 | 71.8 | 69.0 | 71.9 | 71.7 | 72.2 | 70.6 | 70.5 | 70.4 | 70.7 | 70.7 |
| $N^1N^2{*}C^\gamma C^\delta$ | 44.7 | 40.7 | 44.4 | 39.8 | 37.5 | 34.6 | 88.4 | 89.0 | 87.8 | 88.4 | 87.7 |
| $N^1N^2{*}C^\gamma C^\varepsilon$ | 135.4 | 139.4 | 135.7 | 140.2 | 142.4 | 145.5 | 90.7 | 90.4 | 91.1 | 90.3 | 90.8 |

Table S22. Computational structural data for individual triazolium salts **8a-c**, **i**, **k** **(n=2)** and **9a-c**, **k (n=3)** obtained from DFT optimized structures (B3LYP, n=2 and 3).

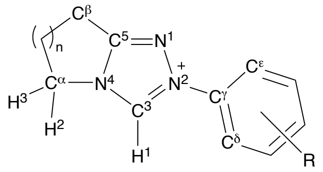


| Backbone Bond Length | **8a** | **8b** | **8c** | **8i** | **8k** | **9a** | **9b** | **9c** | **9k** |
|---|---|---|---|---|---|---|---|---|---|
| $N^1N^2$ | 1.37 | 1.37 | 1.37 | 1.37 | 1.37 | 1.37 | 1.37 | 1.37 | 1.37 |
| $N^2C^3$ | 1.33 | 1.33 | 1.33 | 1.32 | 1.33 | 1.33 | 1.33 | 1.33 | 1.33 |
| $C^3N^4$ | 1.34 | 1.34 | 1.34 | 1.34 | 1.33 | 1.34 | 1.34 | 1.34 | 1.33 |
| $N^4C^5$ | 1.38 | 1.38 | 1.38 | 1.38 | 1.38 | 1.38 | 1.38 | 1.38 | 1.39 |
| $C^5N^1$ | 1.31 | 1.31 | 1.31 | 1.31 | 1.31 | 1.31 | 1.31 | 1.31 | 1.31 |
| $C^5C^\beta$ | 1.49 | 1.49 | 1.49 | 1.49 | 1.49 | 1.49 | 1.49 | 1.49 | 1.49 |
| $N^4C^\alpha$ | 1.48 | 1.48 | 1.49 | 1.49 | 1.49 | 1.48 | 1.48 | 1.48 | 1.48 |
| $N^2C^\gamma$ | 1.43 | 1.44 | 1.43 | 1.45 | 1.42 | 1.43 | 1.44 | 1.43 | 1.42 |
| $C^\gamma C^\delta$ | 1.40 | 1.39 | 1.39 | 1.40 | 1.39 | 1.39 | 1.39 | 1.39 | 1.39 |
| $C^\gamma C^\varepsilon$ | 1.39 | 1.39 | 1.39 | 1.40 | 1.39 | 1.40 | 1.39 | 1.39 | 1.39 |
| C-H Bond Length | | | | | | | | | |
| $C^3H^1$ | 1.08 | 1.08 | 1.08 | 1.08 | 1.08 | 1.08 | 1.08 | 1.08 | 1.08 |
| $C^\alpha H^2$ | 1.09 | 1.09 | 1.09 | 1.09 | 1.09 | 1.09 | 1.09 | 1.09 | 1.09 |
| $C^\alpha H^3$ | 1.09 | 1.09 | 1.09 | 1.09 | 1.09 | 1.09 | 1.09 | 1.09 | 1.09 |
| H-H Distance | | | | | | | | | |
| $H^1H^2$ | 3.11 | 3.12 | 3.12 | 3.14 | 3.15 | 3.55 | 3.53 | 3.53 | 3.54 |
| $H^1H^3$ | 2.79 | 2.78 | 2.78 | 2.78 | 2.78 | 2.48 | 2.49 | 2.49 | 2.49 |
| Bond Angles | | | | | | | | | |
| $C^5N^1N^2$ | 104.9 | 104.9 | 104.9 | 104.9 | 104.5 | 105.2 | 105.2 | 105.1 | 104.7 |
| $N^1N^2C^3$ | 110.9 | 110.9 | 111.0 | 110.8 | 111.3 | 110.7 | 110.7 | 110.8 | 111.0 |
| $N^2C^3N^4$ | 107.0 | 107.0 | 107.0 | 107.2 | 106.8 | 107.3 | 107.3 | 107.2 | 107.0 |
| $C^3N^4C^5$ | 106.7 | 106.8 | 106.8 | 106.7 | 107.0 | 106.7 | 106.7 | 106.7 | 106.9 |
| $N^4C^5N^1$ | 110.4 | 110.4 | 110.4 | 110.4 | 110.5 | 110.2 | 110.2 | 110.2 | 110.3 |
| $C^\beta C^5N^4$ | 122.3 | 122.3 | 122.3 | 122.3 | 122.3 | 124.6 | 124.6 | 124.6 | 124.6 |
| $C^5N^4C^\alpha$ | 125.4 | 125.4 | 125.4 | 125.3 | 125.1 | 127.2 | 127.1 | 127.1 | 127.1 |
| Torsion Angles | | | | | | | | | |
| $H^1C^3{*}C^\alpha H^2$ | 39.9 | 39.1 | 39.3 | 37.9 | 37.7 | 1.0 | 1.6 | 1.5 | 0.6 |
| $H^1C^3{*}C^\alpha H^3$ | 69.1 | 69.9 | 69.8 | 71.2 | 71.5 | 106.4 | 104.0 | 104.0 | 104.9 |
| $N^1N^2{*}C^\gamma C^\delta$ | 40.7 | 38.9 | 40.4 | 84.0 | 82.1 | 43.0 | 39.5 | 41.7 | 86.1 |
| $N^1N^2{*}C^\gamma C^\varepsilon$ | 139.5 | 141.2 | 139.6 | 94.9 | 96.4 | 137.1 | 140.6 | 138.2 | 92.4 |

Table S23. Computational structural data for individual triazolium salts **7a-k** (n=1) obtained from DFT optimized structures (M062X, n=1).

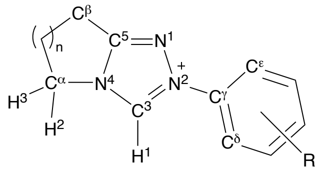


| Backbone Bond Length | **7a** | **7b** | **7c** | **7d** | **7e** | **7f** | **7g** | **7h** | **7i** | **7j** | **7k** |
|---|---|---|---|---|---|---|---|---|---|---|---|
| $N^1N^2$ | 1.36 | 1.36 | 1.36 | 1.36 | 1.36 | 1.36 | 1.37 | 1.36 | 1.37 | 1.37 | 1.37 |
| $N^2C^3$ | 1.33 | 1.33 | 1.33 | 1.33 | 1.33 | 1.33 | 1.33 | 1.33 | 1.33 | 1.33 | 1.33 |
| $C^3N^4$ | 1.33 | 1.33 | 1.33 | 1.33 | 1.33 | 1.33 | 1.33 | 1.33 | 1.33 | 1.32 | 1.32 |
| $N^4C^5$ | 1.36 | 1.37 | 1.37 | 1.37 | 1.37 | 1.37 | 1.37 | 1.37 | 1.37 | 1.37 | 1.37 |
| $C^5N^1$ | 1.30 | 1.30 | 1.30 | 1.30 | 1.30 | 1.30 | 1.30 | 1.30 | 1.30 | 1.30 | 1.30 |
| $C^5C^\beta$ | 1.49 | 1.49 | 1.49 | 1.49 | 1.49 | 1.49 | 1.49 | 1.49 | 1.49 | 1.49 | 1.49 |
| $N^4C^\alpha$ | 1.47 | 1.47 | 1.47 | 1.48 | 1.48 | 1.48 | 1.47 | 1.47 | 1.47 | 1.48 | 1.48 |
| $N^2C^\gamma$ | 1.43 | 1.43 | 1.43 | 1.43 | 1.43 | 1.43 | 1.43 | 1.44 | 1.44 | 1.42 | 1.42 |
| $C^\gamma C^\delta$ | 1.38 | 1.39 | 1.39 | 1.39 | 1.39 | 1.39 | 1.40 | 1.40 | 1.39 | 1.39 | 1.39 |
| $C^\gamma C^\varepsilon$ | 1.39 | 1.39 | 1.39 | 1.39 | 1.39 | 1.39 | 1.40 | 1.40 | 1.39 | 1.39 | 1.39 |
| C-H Bond Length | | | | | | | | | | | |
| $C^3H^1$ | 1.08 | 1.08 | 1.08 | 1.08 | 1.08 | 1.08 | 1.08 | 1.08 | 1.08 | 1.08 | 1.08 |
| $C^\alpha H^2$ | 1.09 | 1.09 | 1.09 | 1.09 | 1.09 | 1.09 | 1.09 | 1.09 | 1.09 | 1.09 | 1.09 |
| $C^\alpha H^3$ | 1.09 | 1.09 | 1.09 | 1.09 | 1.09 | 1.09 | 1.09 | 1.09 | 1.09 | 1.09 | 1.09 |
| H-H Distance | | | | | | | | | | | |
| $H^1H^2$ | 3.15 | 3.13 | 3.15 | 3.13 | 3.13 | 3.12 | 3.14 | 3.15 | 3.14 | 3.14 | 3.14 |
| $H^1H^3$ | 3.39 | 3.42 | 3.39 | 3.42 | 3.41 | 3.41 | 3.41 | 3.41 | 3.42 | 3.41 | 3.40 |
| Bond Angles | | | | | | | | | | | |
| $C^5N^1N^2$ | 103.8 | 103.8 | 103.8 | 103.8 | 103.8 | 103.8 | 103.7 | 103.8 | 103.8 | 103.5 | 103.4 |
| $N^1N^2C^3$ | 111.8 | 111.8 | 111.9 | 111.8 | 111.9 | 111.9 | 111.9 | 111.9 | 111.8 | 112.1 | 112.1 |
| $N^2C^3N^4$ | 105.9 | 105.9 | 105.9 | 105.9 | 105.8 | 105.8 | 106.0 | 106.0 | 106.1 | 105.7 | 105.6 |
| $C^3N^4C^5$ | 107.2 | 107.3 | 107.3 | 107.3 | 107.4 | 107.4 | 107.2 | 107.2 | 107.2 | 107.5 | 107.6 |
| $N^4C^5N^1$ | 111.2 | 111.2 | 111.2 | 111.2 | 111.1 | 111.1 | 111.2 | 111.1 | 111.2 | 111.2 | 111.2 |
| $C^\beta C^5N^4$ | 110.8 | 110.8 | 110.8 | 110.8 | 110.8 | 110.8 | 110.7 | 110.8 | 110.7 | 110.7 | 110.7 |
| $C^5N^4C^\alpha$ | 113.2 | 113.2 | 113.2 | 113.2 | 113.1 | 113.2 | 113.3 | 113.2 | 113.2 | 113.1 | 113.1 |
| Torsion Angles | | | | | | | | | | | |
| $H^1C^3{*}C^\alpha H^2$ | 41.4 | 38.2 | 41.3 | 38.3 | 38.6 | 38.3 | 40.0 | 39.9 | 39.4 | 39.6 | 39.9 |
| $H^1C^3{*}C^\alpha H^3$ | 70.9 | 74.1 | 71.1 | 74.1 | 73.7 | 74.0 | 72.4 | 72.4 | 73.0 | 72.8 | 72.4 |
| $N^1N^2{*}C^\gamma C^\delta$ | 41.2 | 35.9 | 40.6 | 35.0 | 34.7 | 33.5 | 87.6 | 88.8 | 81.8 | 88.1 | 87.6 |
| $N^1N^2{*}C^\gamma C^\varepsilon$ | 138.9 | 144.2 | 139.4 | 144.9 | 145.2 | 146.5 | 91.4 | 90.5 | 97.0 | 90.3 | 90.8 |

Table S24. Computational structural data for individual triazolium salts **8a**-**c**, **i**, **k** **(n=2)** and **9a**-**c**, **k (n=3)** obtained from DFT optimized structures (M062X, n=2 and 3).

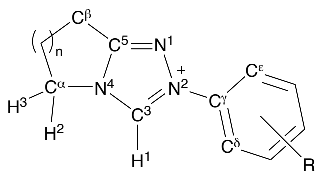


| Backbone Bond Length | **8a** | **8b** | **8c** | **8i** | **8k** | **9a** | **9b** | **9c** | **9k** |
|---|---|---|---|---|---|---|---|---|---|
| $N^1N^2$ | 1.36 | 1.36 | 1.36 | 1.36 | 1.36 | 1.36 | 1.36 | 1.36 | 1.36 |
| $N^2C^3$ | 1.32 | 1.32 | 1.32 | 1.32 | 1.33 | 1.32 | 1.32 | 1.32 | 1.33 |
| $C^3N^4$ | 1.33 | 1.33 | 1.33 | 1.33 | 1.33 | 1.33 | 1.33 | 1.33 | 1.33 |
| $N^4C^5$ | 1.37 | 1.37 | 1.37 | 1.37 | 1.38 | 1.37 | 1.37 | 1.38 | 1.38 |
| $C^5N^1$ | 1.31 | 1.31 | 1.31 | 1.31 | 1.30 | 1.31 | 1.31 | 1.31 | 1.30 |
| $C^5C^{\beta}$ | 1.49 | 1.49 | 1.49 | 1.49 | 1.49 | 1.49 | 1.49 | 1.49 | 1.49 |
| $N^4C^{\alpha}$ | 1.48 | 1.48 | 1.48 | 1.48 | 1.48 | 1.47 | 1.48 | 1.48 | 1.48 |
| $N^2C^{\gamma}$ | 1.43 | 1.43 | 1.43 | 1.44 | 1.42 | 1.43 | 1.43 | 1.43 | 1.42 |
| $C^{\gamma}C^{\delta}$ | 1.39 | 1.39 | 1.39 | 1.40 | 1.39 | 1.38 | 1.39 | 1.39 | 1.39 |
| $C^{\gamma}C^{\varepsilon}$ | 1.38 | 1.39 | 1.39 | 1.39 | 1.39 | 1.39 | 1.39 | 1.39 | 1.39 |
| C-H Bond Length | | | | | | | | | |
| $C^3H^1$ | 1.08 | 1.08 | 1.08 | 1.08 | 1.08 | 1.08 | 1.08 | 1.08 | 1.08 |
| $C^{\alpha}H^2$ | 1.09 | 1.09 | 1.09 | 1.09 | 1.09 | 1.09 | 1.09 | 1.09 | 1.09 |
| $C^{\alpha}H^3$ | 1.09 | 1.09 | 1.09 | 1.09 | 1.09 | 1.09 | 1.09 | 1.09 | 1.09 |
| H-H Distance | | | | | | | | | |
| $H^1H^2$ | 2.78 | 2.77 | 2.77 | 2.77 | 2.76 | 2.50 | 2.50 | 2.50 | 2.50 |
| $H^1H^3$ | 3.12 | 3.12 | 3.12 | 3.16 | 3.14 | 3.58 | 3.57 | 3.57 | 3.57 |
| Bond Angles | | | | | | | | | |
| $C^5N^1N^2$ | 104.9 | 104.9 | 104.9 | 104.9 | 104.5 | 105.1 | 105.1 | 105.1 | 104.7 |
| $N^1N^2C^3$ | 111.2 | 111.2 | 111.3 | 111.2 | 111.6 | 111.1 | 111.1 | 111.1 | 111.4 |
| $N^2C^3N^4$ | 106.8 | 106.8 | 106.8 | 107.0 | 106.5 | 107.0 | 107.0 | 107.0 | 106.7 |
| $C^3N^4C^5$ | 106.7 | 106.7 | 106.8 | 106.6 | 107.0 | 106.7 | 106.7 | 106.7 | 107.0 |
| $N^4C^5N^1$ | 110.3 | 110.3 | 110.3 | 110.3 | 110.4 | 110.2 | 110.2 | 110.2 | 110.3 |
| $C^{\beta}C^5N^4$ | 122.3 | 122.3 | 122.3 | 122.2 | 122.2 | 124.1 | 124.0 | 124.0 | 123.9 |
| $C^5N^4C^{\alpha}$ | 125.5 | 125.5 | 125.5 | 125.4 | 125.3 | 126.6 | 126.6 | 126.6 | 126.4 |
| Torsion Angles | | | | | | | | | |
| $H^1C^3{*}C^{\alpha}H^2$ | 39.8 | 39.4 | 39.5 | 37.3 | 37.8 | 3.0 | 0.8 | 0.9 | 2.0 |
| $H^1C^3{*}C^{\alpha}H^3$ | 69.7 | 70.0 | 70.0 | 72.2 | 71.8 | 108.9 | 106.8 | 106.9 | 108.1 |
| $N^1N^2{*}C^{\gamma}C^{\delta}$ | 38.7 | 36.2 | 38.0 | 83.7 | 65.8 | 40.4 | 37.7 | 40.0 | 67.5 |
| $N^1N^2{*}C^{\gamma}C^{\varepsilon}$ | 141.4 | 143.8 | 141.9 | 94.9 | 112.8 | 140.0 | 142.3 | 139.8 | 111.3 |

Table S25. Summary of average bond angles and distances of triazolium salts **7a-k** (n=1); **8a-c, 8i**, **8k** (n=2); **9a-c**, **9k** (n=3) and corresponding standard deviations obtained from from DFT calculation (B3LYP).

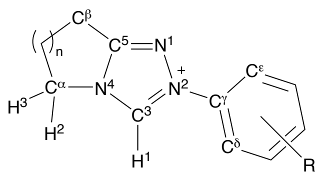


| Backbone Bond Length | Average (n=1) | SD (n=1) | Average (n=2) | SD (n=2) | Average (n=3) | SD (n=3) | Differences of averaged values | n=1 vs n=2 | n=2 vs n=3 |
|---|---|---|---|---|---|---|---|---|---|
| $N^1N^2$ | 1.38 | 0.00 | 1.37 | 0.00 | 1.37 | 0.00 | | -0.01 | 0 |
| $N^2C^3$ | 1.33 | 0.00 | 1.33 | 0.00 | 1.33 | 0.00 | | -0.01 | 0 |
| $C^3N^4$ | 1.33 | 0.00 | 1.34 | 0.00 | 1.34 | 0.00 | | 0.01 | 0 |
| $N^4C^5$ | 1.37 | 0.00 | 1.38 | 0.00 | 1.38 | 0.00 | | 0.01 | 0 |
| $C^5N^1$ | 1.31 | 0.00 | 1.31 | 0.00 | 1.31 | 0.00 | | 0 | 0 |
| $C^5C^\beta$ | 1.49 | 0.00 | 1.49 | 0.00 | 1.49 | 0.00 | | 0 | 0 |
| $N^4C^\alpha$ | 1.48 | 0.00 | 1.49 | 0.00 | 1.48 | 0.00 | | 0.01 | 0 |
| $N^2C^\gamma$ | 1.43 | 0.01 | 1.43 | 0.01 | 1.43 | 0.01 | | 0 | 0 |
| $C^\gamma C^\delta$ | 1.40 | 0.01 | 1.40 | 0.00 | 1.39 | 0.00 | | 0 | 0 |
| $C^\gamma C^\varepsilon$ | 1.40 | 0.01 | 1.39 | 0.00 | 1.39 | 0.00 | | 0 | 0 |
| C-H Bond Length | | | | | | | | | |
| $C^3H^1$ | 1.08 | 0.00 | 1.08 | 0.00 | 1.08 | 0.00 | | 0.00 | 0.00 |
| $C^\alpha H^2$ | 1.09 | 0.00 | 1.09 | 0.00 | 1.09 | 0.00 | | 0.00 | 0.00 |
| $C^\alpha H^3$ | 1.09 | 0.00 | 1.09 | 0.00 | 1.09 | 0.00 | | 0.00 | 0.00 |
| H-H Distance | | | | | | | | | |
| $H^1H^2$ | 3.15 | 0.01 | 3.13 | 0.01 | 3.54 | 0.01 | | -0.02 | 0.41 |
| $H^1H^3$ | 3.38 | 0.01 | 2.78 | 0.00 | 2.49 | 0.00 | | -0.60 | -0.30 |
| Bond Angles | | | | | | | | | |
| $C^5N^1N^2$ | 103.7 | 0.1 | 104.8 | 0.2 | 105.1 | 0.2 | | 1.1 | 0.25 |
| $N^1N^2C^3$ | 111.5 | 0.1 | 111.0 | 0.2 | 110.8 | 0.2 | | -0.55 | -0.18 |
| $N^2C^3N^4$ | 106.1 | 0.2 | 107.0 | 0.2 | 107.2 | 0.1 | | 0.94 | 0.2 |
| $C^3N^4C^5$ | 107.4 | 0.1 | 106.8 | 0.1 | 106.7 | 0.1 | | -0.63 | -0.07 |
| $N^4C^5N^1$ | 111.3 | 0.0 | 110.4 | 0.1 | 110.2 | 0.1 | | -0.86 | -0.21 |
| $C^\beta C^5N^4$ | 111.0 | 0.1 | 122.3 | 0.0 | 124.6 | 0.0 | | 11.27 | 2.32 |
| $C^5N^4C^\alpha$ | 113.4 | 0.1 | 125.3 | 0.1 | 127.1 | 0.0 | | 11.9 | 1.82 |
| Torsion Angles | | | | | | | | | |
| $H^1C^3*C^\alpha H^2$ | 41.1 | 1.2 | 38.8 | 1.0 | 1.2 | 0.5 | | -2.31 | -37.59 |
| $H^1C^3*C^\alpha H^3$ | 70.7 | 1.2 | 70.3 | 1.0 | 104.8 | 1.1 | | -0.44 | 34.53 |
| $N^1N^2*C^\gamma C^\delta$ | 62.1 | 25.2 | 57.2 | 23.6 | 52.6 | 22.4 | | -4.89 | -4.65 |
| $N^1N^2*C^\gamma C^\varepsilon$ | 117.4 | 25.8 | 122.3 | 24.4 | 127.1 | 23.1 | | 4.88 | 4.78 |

Table S26. Summary of average bond angles and distances of triazolium salts **7a-k** (n=1); **8a-c, 8i**, **8k** (n=2); **9a-c**, **9k** (n=3) and corresponding standard deviations obtained from from DFT calculation (M062X).

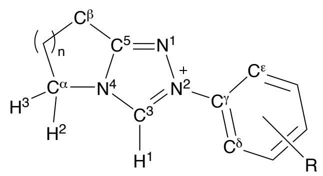


| Backbone Bond Length | Average (n=1) | SD (n=1) | Average (n=2) | SD (n=2) | Average (n=3) | SD (n=3) | Differences of averaged values | n=1 vs n=2 | n=2 vs n=3 |
|---|---|---|---|---|---|---|---|---|---|
| $N^1N^2$ | 1.36 | 0.00 | 1.36 | 0.00 | 1.36 | 0.00 | | -0.01 | 0 |
| $N^2C^3$ | 1.33 | 0.00 | 1.32 | 0.00 | 1.32 | 0.00 | | -0.01 | 0 |
| $C^3N^4$ | 1.33 | 0.00 | 1.33 | 0.00 | 1.33 | 0.00 | | 0 | 0 |
| $N^4C^5$ | 1.37 | 0.00 | 1.37 | 0.00 | 1.38 | 0.00 | | 0.01 | 0 |
| $C^5N^1$ | 1.30 | 0.00 | 1.31 | 0.00 | 1.31 | 0.00 | | 0 | 0 |
| $C^5C^\beta$ | 1.49 | 0.00 | 1.49 | 0.00 | 1.49 | 0.00 | | 0 | 0 |
| $N^4C^\alpha$ | 1.47 | 0.00 | 1.48 | 0.00 | 1.48 | 0.00 | | 0.01 | 0 |
| $N^2C^\gamma$ | 1.43 | 0.01 | 1.43 | 0.01 | 1.43 | 0.01 | | 0 | 0 |
| $C^\gamma C^\delta$ | 1.39 | 0.00 | 1.39 | 0.00 | 1.39 | 0.00 | | 0 | 0 |
| $C^\gamma C^\varepsilon$ | 1.39 | 0.00 | 1.39 | 0.00 | 1.39 | 0.00 | | 0 | 0 |
| C-H Bond Length | | | | | | | | | |
| $C^3H^1$ | 1.08 | 0.00 | 1.08 | 0.00 | 1.08 | 0.00 | | 0.00 | 0.00 |
| $C^\alpha H^2$ | 1.09 | 0.00 | 1.09 | 0.00 | 1.09 | 0.00 | | 0.00 | 0.00 |
| $C^\alpha H^3$ | 1.09 | 0.00 | 1.09 | 0.00 | 1.09 | 0.00 | | 0.00 | 0.00 |
| H-H Distance | | | | | | | | | |
| $H^1H^2$ | 3.14 | 0.01 | 2.77 | 0.01 | 2.50 | 0.00 | | -0.37 | -0.27 |
| $H^1H^3$ | 3.41 | 0.01 | 3.13 | 0.02 | 3.57 | 0.01 | | -0.28 | 0.44 |
| Bond Angles | | | | | | | | | |
| $C^5N^1N^2$ | 103.7 | 0.1 | 104.8 | 0.2 | 105.0 | 0.2 | | 1.08 | 0.16 |
| $N^1N^2C^3$ | 111.9 | 0.1 | 111.3 | 0.1 | 111.2 | 0.2 | | -0.6 | -0.14 |
| $N^2C^3N^4$ | 105.9 | 0.1 | 106.8 | 0.2 | 106.9 | 0.1 | | 0.92 | 0.12 |
| $C^3N^4C^5$ | 107.3 | 0.1 | 106.8 | 0.1 | 106.8 | 0.1 | | -0.56 | 0 |
| $N^4C^5N^1$ | 111.2 | 0.0 | 110.3 | 0.0 | 110.2 | 0.1 | | -0.84 | -0.13 |
| $C^\beta C^5N^4$ | 110.8 | 0.1 | 122.3 | 0.0 | 124.0 | 0.1 | | 11.49 | 1.73 |
| $C^5N^4C^\alpha$ | 113.2 | 0.0 | 125.4 | 0.1 | 126.5 | 0.1 | | 12.24 | 1.1 |
| Torsion Angles | | | | | | | | | |
| $H^1C^3{*}C^\alpha H^2$ | 39.5 | 1.1 | 38.8 | 1.1 | 1.6 | 1.0 | | -0.78 | -37.11 |
| $H^1C^3{*}C^\alpha H^3$ | 72.8 | 1.1 | 70.7 | 1.2 | 107.7 | 1.0 | | -2.07 | 36.96 |
| $N^1N^2{*}C^\gamma C^\delta$ | 59.5 | 26.3 | 52.5 | 21.3 | 46.4 | 14.1 | | -7.05 | -6.08 |
| $N^1N^2{*}C^\gamma C^\varepsilon$ | 119.9 | 26.9 | 127.0 | 22.1 | 133.4 | 14.7 | | 7.05 | 6.4 |

Table S27. Summary of average* bond angles and distances of triazolium salts **7a-c, k** (n=1); **8a-c, 8k** (n=2); **9a-c**, **9k** (n=3) and corresponding standard deviations (SD) obtained from DFT calculation (M062X).

*Averages calculated using only data for the 12 triazolium salts related to C(3)-H/D exchange studies. These average values and trends are in excellent agreement with data in Table S30 as expected given the small standard deviations.

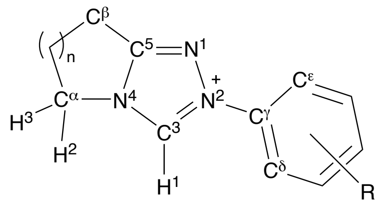


| Backbone Bond Length | Average (n=1) | SD (n=1) | Average (n=2) | SD (n=2) | Average (n=3) | SD (n=3) |
|---|---|---|---|---|---|---|
| $N^1N^2$ | 1.36 | 0.00 | 1.36 | 0.00 | 1.36 | 0.00 |
| $N^2C^3$ | 1.33 | 0.00 | 1.32 | 0.00 | 1.32 | 0.00 |
| $C^3N^4$ | 1.33 | 0.01 | 1.33 | 0.00 | 1.33 | 0.00 |
| $N^4C^5$ | 1.37 | 0.00 | 1.37 | 0.00 | 1.38 | 0.00 |
| $C^5N^1$ | 1.30 | 0.00 | 1.31 | 0.00 | 1.31 | 0.00 |
| $C^5C^\beta$ | 1.49 | 0.00 | 1.49 | 0.00 | 1.49 | 0.00 |
| $N^4C^\alpha$ | 1.47 | 0.00 | 1.48 | 0.00 | 1.48 | 0.00 |
| $N^2C^\gamma$ | 1.43 | 0.01 | 1.43 | 0.01 | 1.43 | 0.01 |
| $C^\gamma C^\delta$ | 1.39 | 0.00 | 1.39 | 0.00 | 1.39 | 0.00 |
| $C^\gamma C^\varepsilon$ | 1.39 | 0.00 | 1.39 | 0.00 | 1.39 | 0.00 |
| C-H Bond Length | | | | | | |
| $C^3H^1$ | 1.08 | 0.00 | 1.08 | 0.00 | 1.08 | 0.00 |
| $C^\alpha H^2$ | 1.09 | 0.00 | 1.09 | 0.00 | 1.09 | 0.00 |
| $C^\alpha H^3$ | 1.09 | 0.00 | 1.09 | 0.00 | 1.09 | 0.00 |
| H-H Distance | | | | | | |
| $H^1H^2$ | 3.14 | 0.01 | 2.77 | 0.01 | 2.50 | 0.00 |
| $H^1H^3$ | 3.40 | 0.01 | 3.12 | 0.01 | 3.57 | 0.01 |
| Bond Angles | | | | | | |
| $C^5N^1N^2$ | 103.69 | 0.20 | 104.80 | 0.20 | 104.97 | 0.20 |
| $N^1N^2C^3$ | 111.90 | 0.14 | 111.31 | 0.17 | 111.16 | 0.15 |
| $N^2C^3N^4$ | 105.82 | 0.15 | 106.73 | 0.13 | 106.91 | 0.14 |
| $C^3N^4C^5$ | 107.36 | 0.16 | 106.80 | 0.14 | 106.76 | 0.13 |
| $N^4C^5N^1$ | 111.19 | 0.01 | 110.32 | 0.05 | 110.20 | 0.06 |
| $C^\beta C^5N^4$ | 110.79 | 0.07 | 122.27 | 0.05 | 123.99 | 0.06 |
| $C^5N^4C^\alpha$ | 113.18 | 0.06 | 125.45 | 0.10 | 126.53 | 0.10 |

| Torsion Angles | | | | | | |
|---|---|---|---|---|---|---|
| $H^1C^3{*}C^{\alpha}H^2$ | 40.21 | 1.51 | 39.13 | 0.90 | 1.65 | 1.05 |
| $H^1C^3{*}C^{\alpha}H^3$ | 72.12 | 1.49 | 70.37 | 0.96 | 107.69 | 1.00 |
| $N^1N^2{*}C^{\gamma}C^{\delta}$ | 51.32 | 24.30 | 44.68 | 14.14 | 46.41 | 14.12 |
| $N^1N^2{*}C^{\gamma}C^{\varepsilon}$ | 128.33 | 25.13 | 134.99 | 14.80 | 133.37 | 14.74 |

## S2.4.2 Summary for Triazolylidene Computational Analysis

Table S28. Computational structural data for individual triazolylidenes **7'a-k** (n=1) obtained from DFT optimized structures (B3LYP, n=1).

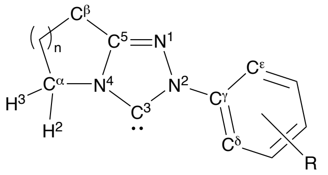


| Backbone Bond Length | 7’a | 7’b | 7’c | 7’d | 7’e | 7’f | 7’g | 7’h | 7’i | 7’j | 7’k |
|---|---|---|---|---|---|---|---|---|---|---|---|
| $N^1N^2$ | 1.40 | 1.40 | 1.40 | 1.40 | 1.40 | 1.40 | 1.41 | 1.41 | 1.41 | 1.41 | 1.41 |
| $N^2C^3$ | 1.36 | 1.36 | 1.36 | 1.36 | 1.36 | 1.37 | 1.35 | 1.36 | 1.35 | 1.36 | 1.36 |
| $C^3N^4$ | 1.36 | 1.36 | 1.36 | 1.36 | 1.36 | 1.36 | 1.36 | 1.36 | 1.36 | 1.36 | 1.36 |
| $N^4C^5$ | 1.37 | 1.37 | 1.37 | 1.37 | 1.37 | 1.37 | 1.37 | 1.37 | 1.37 | 1.37 | 1.37 |
| $C^5N^1$ | 1.30 | 1.30 | 1.30 | 1.30 | 1.30 | 1.30 | 1.30 | 1.30 | 1.30 | 1.30 | 1.30 |
| $C^5C^{\beta}$ | 1.49 | 1.49 | 1.49 | 1.49 | 1.49 | 1.49 | 1.49 | 1.49 | 1.49 | 1.49 | 1.49 |
| $N^4C^{\alpha}$ | 1.47 | 1.47 | 1.47 | 1.47 | 1.47 | 1.47 | 1.47 | 1.47 | 1.47 | 1.47 | 1.47 |
| $N^2C^{\gamma}$ | 1.43 | 1.43 | 1.42 | 1.42 | 1.42 | 1.42 | 1.42 | 1.44 | 1.44 | 1.42 | 1.41 |
| $C^{\gamma}C^{\delta}$ | 1.40 | 1.40 | 1.40 | 1.40 | 1.40 | 1.40 | 1.40 | 1.40 | 1.40 | 1.40 | 1.39 |
| $C^{\gamma}C^{\varepsilon}$ | 1.39 | 1.40 | 1.40 | 1.40 | 1.40 | 1.40 | 1.40 | 1.41 | 1.40 | 1.40 | 1.39 |
| C-H Bond Length | | | | | | | | | | | |
| $C^{\alpha}H^2$ | 1.09 | 1.09 | 1.09 | 1.09 | 1.09 | 1.09 | 1.09 | 1.09 | 1.09 | 1.09 | 1.09 |
| $C^{\alpha}H^3$ | 1.09 | 1.09 | 1.09 | 1.09 | 1.09 | 1.09 | 1.09 | 1.09 | 1.09 | 1.09 | 1.09 |
| C-H Distance | | | | | | | | | | | |
| $C^3H^2$ | 2.93 | 2.93 | 2.93 | 2.93 | 2.93 | 2.92 | 2.93 | 2.93 | 2.94 | 2.93 | 2.92 |
| $C^3H^3$ | 3.05 | 3.05 | 3.05 | 3.04 | 3.04 | 3.04 | 3.05 | 3.05 | 3.05 | 3.05 | 3.04 |
| Bond Angles | | | | | | | | | | | |
| $C^5N^1N^2$ | 102.1 | 102.1 | 102.0 | 102.1 | 102.1 | 102.1 | 101.9 | 102.0 | 101.9 | 101.5 | 101.4 |
| $N^1N^2C^3$ | 115.3 | 115.2 | 115.3 | 115.2 | 115.2 | 115.2 | 115.5 | 115.4 | 115.4 | 115.9 | 115.9 |
| $N^2C^3N^4$ | 100.1 | 100.1 | 100.0 | 100.1 | 100.1 | 100.0 | 100.1 | 100.2 | 100.2 | 99.7 | 99.6 |
| $C^3N^4C^5$ | 111.5 | 111.5 | 111.6 | 111.6 | 111.6 | 111.6 | 111.4 | 111.4 | 111.4 | 111.6 | 111.8 |
| $N^4C^5N^1$ | 111.0 | 111.0 | 111.0 | 111.0 | 111.0 | 111.1 | 111.1 | 111.0 | 111.1 | 111.2 | 111.3 |
| $C^{\beta}C^5N^4$ | 111.3 | 111.3 | 111.3 | 111.3 | 111.3 | 111.3 | 111.2 | 111.3 | 111.3 | 111.2 | 111.2 |
| $C^5N^4C^{\alpha}$ | 112.9 | 112.9 | 112.9 | 112.8 | 112.8 | 112.8 | 112.9 | 112.9 | 112.9 | 112.8 | 112.7 |
| Torsion Angles | | | | | | | | | | | |
| $C^3N^1{*}C^{\alpha}H^2$ | 52.3 | 52.6 | 52.6 | 52.3 | 52.4 | 52.2 | 52.3 | 52.2 | 52.3 | 52.2 | 52.1 |
| $C^3N^1{*}C^{\alpha}H^3$ | 83.6 | 83.3 | 83.3 | 83.5 | 83.4 | 83.6 | 83.6 | 83.7 | 83.6 | 83.7 | 83.8 |
| $N^1N^2{*}C^{\gamma}C^{\delta}$ | 29.0 | 20.8 | 21.3 | 0.9 | 0.7 | 0.6 | 89.5 | 89.5 | 88.7 | 88.9 | 68.1 |
| $N^1N^2{*}C^{\gamma}C^{\varepsilon}$ | 151.2 | 159.2 | 158.8 | 179.2 | 179.3 | 179.4 | 89.8 | 89.9 | 90.9 | 90.3 | 111.4 |

Table S29. Computational structural data for individual triazolylidenes **8'a-c**, **i**, **k (n=2)** and **9'a-c**, **k (n=3)** obtained from DFT optimized structures (B3LYP, n=2 and 3).

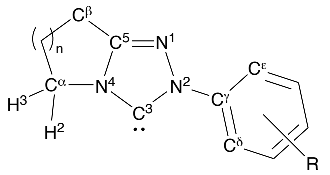


| Backbone Bond Length | 8’a | 8’b | 8’c | 8’i | 8’k | 9’a | 9’b | 9’c | 9’k |
|---|---|---|---|---|---|---|---|---|---|
| $N^1N^2$ | 1.39 | 1.39 | 1.39 | 1.39 | 1.40 | 1.39 | 1.39 | 1.39 | 1.39 |
| $N^2C^3$ | 1.35 | 1.35 | 1.35 | 1.35 | 1.35 | 1.35 | 1.36 | 1.35 | 1.35 |
| $C^3N^4$ | 1.37 | 1.37 | 1.37 | 1.37 | 1.36 | 1.37 | 1.37 | 1.37 | 1.37 |
| $N^4C^5$ | 1.38 | 1.38 | 1.38 | 1.38 | 1.38 | 1.38 | 1.38 | 1.38 | 1.39 |
| $C^5N^1$ | 1.30 | 1.30 | 1.30 | 1.30 | 1.30 | 1.30 | 1.30 | 1.30 | 1.30 |
| $C^5C^\beta$ | 1.50 | 1.50 | 1.50 | 1.50 | 1.50 | 1.49 | 1.49 | 1.49 | 1.49 |
| $N^4C^\alpha$ | 1.47 | 1.47 | 1.47 | 1.47 | 1.47 | 1.47 | 1.47 | 1.47 | 1.47 |
| $N^2C^\gamma$ | 1.43 | 1.43 | 1.42 | 1.44 | 1.41 | 1.43 | 1.43 | 1.42 | 1.41 |
| $C^\gamma C^\delta$ | 1.40 | 1.40 | 1.40 | 1.40 | 1.39 | 1.39 | 1.40 | 1.40 | 1.39 |
| $C^\gamma C^\varepsilon$ | 1.39 | 1.40 | 1.40 | 1.40 | 1.39 | 1.40 | 1.40 | 1.40 | 1.39 |
| C-H Bond Length | | | | | | | | | |
| $C^\alpha H^2$ | 1.09 | 1.09 | 1.09 | 1.09 | 1.09 | 1.09 | 1.09 | 1.09 | 1.09 |
| $C^\alpha H^3$ | 1.09 | 1.09 | 1.09 | 1.09 | 1.09 | 1.09 | 1.09 | 1.09 | 1.09 |
| C-H Distance | | | | | | | | | |
| $C^3H^2$ | 2.91 | 2.91 | 2.91 | 2.92 | 2.91 | 3.17 | 3.17 | 3.17 | 3.16 |
| $C^3H^3$ | 2.71 | 2.70 | 2.70 | 2.71 | 2.70 | 2.53 | 2.52 | 2.52 | 2.52 |
| Bond Angles | | | | | | | | | |
| $C^5N^1N^2$ | 103.3 | 103.3 | 103.2 | 103.1 | 102.6 | 103.5 | 103.6 | 103.5 | 102.8 |
| $N^1N^2C^3$ | 114.9 | 114.8 | 114.9 | 115.0 | 115.5 | 114.8 | 114.7 | 114.8 | 115.4 |
| $N^2C^3N^4$ | 101.0 | 101.0 | 101.0 | 101.1 | 100.5 | 101.1 | 101.2 | 101.1 | 100.7 |
| $C^3N^4C^5$ | 110.7 | 110.7 | 110.7 | 110.6 | 110.9 | 110.6 | 110.6 | 110.6 | 110.8 |
| $N^4C^5N^1$ | 110.2 | 110.2 | 110.2 | 110.2 | 110.4 | 110.0 | 110.0 | 110.0 | 110.3 |
| $C^\beta C^5N^4$ | 122.9 | 122.9 | 122.9 | 122.9 | 122.9 | 125.2 | 125.2 | 125.2 | 125.1 |
| $C^5N^4C^\alpha$ | 124.1 | 124.1 | 124.0 | 124.1 | 124.0 | 125.4 | 125.4 | 125.4 | 125.4 |
| Torsion Angles | | | | | | | | | |
| $C^3N^1{*}C^\alpha H^2$ | 82.3 | 82.4 | 82.5 | 82.3 | 82.4 | 0.1 | 0.0 | 0.1 | 0.3 |
| $C^3N^1{*}C^\alpha H^3$ | 42.0 | 42.0 | 41.8 | 42.1 | 42.0 | 118.3 | 118.4 | 118.3 | 118.5 |
| $N^1N^2{*}C^\gamma C^\delta$ | 28.8 | 21.2 | 21.1 | 86.0 | 70.3 | 27.1 | 17.5 | 19.7 | 72.0 |
| $N^1N^2{*}C^\gamma C^\varepsilon$ | 151.4 | 158.8 | 159.0 | 93.2 | 109.3 | 153.2 | 162.6 | 160.4 | 107.6 |

Table S30. Computational structural data for individual triazolylidenes **7'a-k** (n=1) obtained from DFT optimized structures (M062X, n=1).

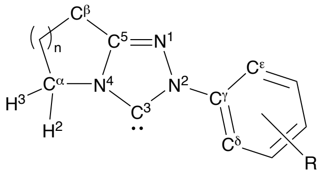


| Backbone Bond Length | **7'a** | **7'b** | **7'c** | **7'd** | **7'e** | **7'f** | **7'g** | **7'h** | **7'i** | **7'j** | **7'k** |
|---|---|---|---|---|---|---|---|---|---|---|---|
| $N^1N^2$ | 1.39 | 1.39 | 1.39 | 1.39 | 1.39 | 1.39 | 1.39 | 1.39 | 1.39 | 1.39 | 1.39 |
| $N^2C^3$ | 1.36 | 1.36 | 1.36 | 1.36 | 1.36 | 1.36 | 1.35 | 1.35 | 1.35 | 1.36 | 1.36 |
| $C^3N^4$ | 1.36 | 1.36 | 1.36 | 1.35 | 1.35 | 1.35 | 1.36 | 1.36 | 1.36 | 1.36 | 1.35 |
| $N^4C^5$ | 1.37 | 1.37 | 1.37 | 1.37 | 1.37 | 1.37 | 1.37 | 1.37 | 1.37 | 1.37 | 1.37 |
| $C^5N^1$ | 1.29 | 1.29 | 1.29 | 1.29 | 1.29 | 1.29 | 1.29 | 1.30 | 1.29 | 1.29 | 1.29 |
| $C^5C^{\beta}$ | 1.49 | 1.49 | 1.49 | 1.49 | 1.49 | 1.49 | 1.49 | 1.49 | 1.49 | 1.49 | 1.49 |
| $N^4C^{\alpha}$ | 1.46 | 1.46 | 1.46 | 1.46 | 1.46 | 1.46 | 1.46 | 1.46 | 1.46 | 1.46 | 1.46 |
| $N^2C^{\gamma}$ | 1.42 | 1.42 | 1.42 | 1.42 | 1.42 | 1.42 | 1.42 | 1.43 | 1.43 | 1.41 | 1.41 |
| $C^{\gamma}C^{\delta}$ | 1.39 | 1.39 | 1.39 | 1.39 | 1.39 | 1.39 | 1.40 | 1.40 | 1.40 | 1.39 | 1.39 |
| $C^{\gamma}C^{\varepsilon}$ | 1.39 | 1.39 | 1.39 | 1.39 | 1.39 | 1.40 | 1.40 | 1.40 | 1.40 | 1.39 | 1.39 |
| C-H Bond Length | | | | | | | | | | | |
| $C^{\alpha}H^2$ | 1.09 | 1.09 | 1.09 | 1.09 | 1.09 | 1.09 | 1.09 | 1.09 | 1.09 | 1.09 | 1.09 |
| $C^{\alpha}H^3$ | 1.09 | 1.09 | 1.09 | 1.09 | 1.09 | 1.09 | 1.09 | 1.09 | 1.09 | 1.09 | 1.09 |
| C-H Distance | | | | | | | | | | | |
| $C^3H^2$ | 2.91 | 2.92 | 2.91 | 2.91 | 2.91 | 2.91 | 2.92 | 2.92 | 2.92 | 2.91 | 2.91 |
| $C^3H^3$ | 3.05 | 3.05 | 3.05 | 3.05 | 3.05 | 3.05 | 3.05 | 3.06 | 3.06 | 3.05 | 3.05 |
| Bond Angles | | | | | | | | | | | |
| $C^5N^1N^2$ | 102.2 | 102.2 | 102.2 | 102.3 | 102.2 | 102.2 | 102.0 | 102.1 | 102.1 | 101.7 | 101.6 |
| $N^1N^2C^3$ | 115.8 | 115.7 | 115.8 | 115.6 | 115.7 | 115.6 | 116.0 | 116.0 | 116.0 | 116.4 | 116.4 |
| $N^2C^3N^4$ | 99.7 | 99.7 | 99.6 | 99.7 | 99.7 | 99.7 | 99.7 | 99.7 | 99.7 | 99.3 | 99.2 |
| $C^3N^4C^5$ | 111.5 | 111.5 | 111.6 | 111.6 | 111.6 | 111.6 | 111.4 | 111.4 | 111.4 | 111.6 | 111.8 |
| $N^4C^5N^1$ | 110.8 | 110.8 | 110.8 | 110.8 | 110.8 | 110.9 | 110.9 | 110.8 | 110.9 | 111.0 | 111.1 |
| $C^{\beta}C^5N^4$ | 111.1 | 111.1 | 111.1 | 111.1 | 111.1 | 111.1 | 111.1 | 111.1 | 111.1 | 111.0 | 111.0 |
| $C^5N^4C^{\alpha}$ | 112.7 | 112.7 | 112.7 | 112.7 | 112.7 | 112.6 | 112.7 | 112.7 | 112.7 | 112.6 | 112.5 |
| Torsion Angles | | | | | | | | | | | |
| $C^3N^1*C^{\alpha}H^2$ | 50.6 | 50.6 | 50.7 | 50.7 | 50.6 | 50.7 | 50.7 | 50.6 | 50.5 | 50.4 | 50.4 |
| $C^3N^1*C^{\alpha}H^3$ | 85.4 | 85.3 | 85.3 | 85.2 | 85.3 | 85.3 | 85.3 | 85.4 | 85.4 | 85.6 | 85.7 |
| $N^1N^2*C^{\gamma}C^{\delta}$ | 25.9 | 21.0 | 22.5 | 1.6 | 10.5 | 1.7 | 89.4 | 88.3 | 83.4 | 87.5 | 60.2 |
| $N^1N^2*C^{\gamma}C^{\varepsilon}$ | 154.4 | 159.0 | 157.6 | 178.3 | 169.6 | 178.4 | 90.0 | 91.3 | 96.3 | 91.8 | 119.5 |

Table S31. Computational structural data for individual triazolylidenes **8'a-c**, **i**, **k** **(n=2)** and **9'a-c**, **k (n=3)** obtained from DFT optimized structures (M062X, n=2 and 3).

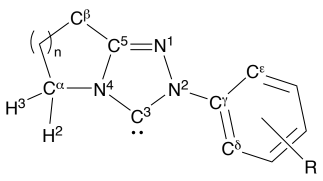


| Backbone Bond Length | 8'a | 8'b | 8'c | 8'i | 8'k | 9'a | 9'b | 9'c | 9'k |
|---|---|---|---|---|---|---|---|---|---|
| $N^1N^2$ | 1.38 | 1.38 | 1.38 | 1.38 | 1.38 | 1.37 | 1.38 | 1.38 | 1.38 |
| $N^2C^3$ | 1.35 | 1.35 | 1.35 | 1.34 | 1.35 | 1.35 | 1.35 | 1.35 | 1.35 |
| $C^3N^4$ | 1.36 | 1.36 | 1.36 | 1.37 | 1.36 | 1.36 | 1.36 | 1.36 | 1.36 |
| $N^4C^5$ | 1.37 | 1.38 | 1.38 | 1.37 | 1.38 | 1.37 | 1.38 | 1.38 | 1.38 |
| $C^5N^1$ | 1.30 | 1.30 | 1.30 | 1.30 | 1.30 | 1.30 | 1.30 | 1.30 | 1.30 |
| $C^5C^\beta$ | 1.49 | 1.49 | 1.49 | 1.50 | 1.49 | 1.49 | 1.49 | 1.49 | 1.49 |
| $N^4C^\alpha$ | 1.47 | 1.47 | 1.47 | 1.47 | 1.47 | 1.46 | 1.46 | 1.46 | 1.46 |
| $N^2C^\gamma$ | 1.42 | 1.42 | 1.42 | 1.43 | 1.41 | 1.42 | 1.42 | 1.42 | 1.41 |
| $C^\gamma C^\delta$ | 1.39 | 1.39 | 1.39 | 1.40 | 1.39 | 1.39 | 1.39 | 1.39 | 1.39 |
| $C^\gamma C^\varepsilon$ | 1.39 | 1.39 | 1.39 | 1.39 | 1.39 | 1.40 | 1.39 | 1.39 | 1.39 |
| **C-H Bond Length** | | | | | | | | | |
| $C^\alpha H^2$ | 1.09 | 1.09 | 1.09 | 1.09 | 1.09 | 1.09 | 1.09 | 1.09 | 1.09 |
| $C^\alpha H^3$ | 1.09 | 1.09 | 1.09 | 1.09 | 1.09 | 1.09 | 1.09 | 1.09 | 1.09 |
| **C-H Distance** | | | | | | | | | |
| $C^3H^2$ | 2.70 | 2.69 | 2.69 | 2.69 | 2.69 | 2.53 | 2.53 | 2.53 | 2.53 |
| $C^3H^3$ | 2.90 | 2.91 | 2.90 | 2.91 | 2.90 | 3.18 | 3.18 | 3.18 | 3.18 |
| **Bond Angles** | | | | | | | | | |
| $C^5N^1N^2$ | 103.3 | 103.3 | 103.3 | 103.2 | 102.7 | 103.5 | 103.5 | 103.5 | 102.8 |
| $N^1N^2C^3$ | 115.3 | 115.2 | 115.3 | 115.5 | 115.9 | 115.2 | 115.1 | 115.2 | 115.8 |
| $N^2C^3N^4$ | 100.6 | 100.6 | 100.6 | 100.6 | 100.1 | 100.7 | 100.7 | 100.7 | 100.2 |
| $C^3N^4C^5$ | 110.7 | 110.7 | 110.7 | 110.6 | 111.0 | 110.7 | 110.7 | 110.7 | 111.0 |
| $N^4C^5N^1$ | 110.0 | 110.1 | 110.1 | 110.1 | 110.3 | 110.0 | 110.0 | 110.0 | 110.2 |
| $C^\beta C^5N^4$ | 123.0 | 123.0 | 123.0 | 122.9 | 122.9 | 124.6 | 124.6 | 124.6 | 124.4 |
| $C^5N^4C^\alpha$ | 124.2 | 124.2 | 124.1 | 124.2 | 124.0 | 124.7 | 124.7 | 124.7 | 124.5 |
| **Torsion Angles** | | | | | | | | | |
| $C^3N^1{*}C^\alpha H^2$ | 42.4 | 42.4 | 42.0 | 41.8 | 41.8 | 2.8 | 2.7 | 2.7 | 3.2 |
| $C^3N^1{*}C^\alpha H^3$ | 82.4 | 82.4 | 82.7 | 82.9 | 82.9 | 121.4 | 121.3 | 121.3 | 121.9 |
| $N^1N^2{*}C^\gamma C^\delta$ | 26.4 | 21.7 | 23.4 | 84.3 | 62.8 | 23.6 | 17.2 | 19.5 | 63.2 |
| $N^1N^2{*}C^\gamma C^\varepsilon$ | 153.8 | 158.3 | 156.6 | 94.7 | 117.0 | 156.6 | 162.9 | 160.6 | 116.2 |

Table S32. Summary of average bond angles and distances of triazolylidenes **7'a-k** (n=1); **8'a-c, i**, **k** (n=2); **9'a-c**, **k** (n=3) and corresponding standard deviations obtained from DFT calculation (B3LYP).

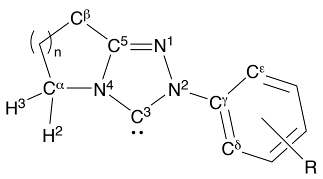


| Backbone Bond Length | Average (n=1) | SD (n=1) | Average (n=2) | SD (n=2) | Average (n=3) | SD (n=3) | Differences of averaged values | n=1 vs n=2 | n=2 vs n=3 |
|---|---|---|---|---|---|---|---|---|---|
| $N^1N^2$ | 1.41 | 0.00 | 1.39 | 0.00 | 1.39 | 0.00 | | -0.01 | 0 |
| $N^2C^3$ | 1.36 | 0.00 | 1.35 | 0.00 | 1.35 | 0.00 | | -0.01 | 0 |
| $C^3N^4$ | 1.36 | 0.00 | 1.37 | 0.00 | 1.37 | 0.00 | | 0.01 | 0 |
| $N^4C^5$ | 1.37 | 0.00 | 1.38 | 0.00 | 1.38 | 0.00 | | 0.01 | 0 |
| $C^5N^1$ | 1.30 | 0.00 | 1.30 | 0.00 | 1.30 | 0.00 | | 0.01 | 0 |
| $C^5C^\beta$ | 1.49 | 0.00 | 1.50 | 0.00 | 1.49 | 0.00 | | 0 | 0 |
| $N^4C^\alpha$ | 1.47 | 0.00 | 1.47 | 0.00 | 1.47 | 0.00 | | 0 | 0 |
| $N^2C^\gamma$ | 1.42 | 0.01 | 1.43 | 0.01 | 1.42 | 0.01 | | 0 | 0 |
| $C^\gamma C^\delta$ | 1.40 | 0.00 | 1.40 | 0.00 | 1.39 | 0.00 | | 0 | 0 |
| $C^\gamma C^\varepsilon$ | 1.40 | 0.00 | 1.40 | 0.00 | 1.40 | 0.00 | | 0 | 0 |
| C-H Bond Length | | | | | | | | | |
| $C^\alpha H^2$ | 1.09 | 0.00 | 1.09 | 0.00 | 1.09 | 0.00 | | 0.00 | 0.00 |
| $C^\alpha H^3$ | 1.09 | 0.00 | 1.09 | 0.00 | 1.09 | 0.00 | | 0.00 | 0.00 |
| C-H Distance | | | | | | | | | |
| $C^3H^2$ | 2.93 | 0.00 | 2.91 | 0.00 | 3.17 | 0.00 | | -0.02 | 0.25 |
| $C^3H^3$ | 3.05 | 0.00 | 2.70 | 0.00 | 2.52 | 0.00 | | -0.34 | -0.18 |
| Bond Angles | | | | | | | | | |
| $C^5N^1N^2$ | 101.9 | 0.2 | 103.1 | 0.3 | 103.4 | 0.4 | | 1.2 | 0.3 |
| $N^1N^2C^3$ | 115.4 | 0.3 | 115.0 | 0.3 | 114.9 | 0.3 | | -0.4 | -0.1 |
| $N^2C^3N^4$ | 100.0 | 0.2 | 100.9 | 0.2 | 101.0 | 0.2 | | 0.9 | 0.1 |
| $C^3N^4C^5$ | 111.5 | 0.1 | 110.7 | 0.1 | 110.6 | 0.1 | | -0.8 | -0.1 |
| $N^4C^5N^1$ | 111.1 | 0.1 | 110.2 | 0.1 | 110.1 | 0.1 | | -0.8 | -0.2 |
| $C^\beta C^5N^4$ | 111.3 | 0.0 | 122.9 | 0.0 | 125.1 | 0.1 | | 11.6 | 2.2 |
| $C^5N^4C^\alpha$ | 112.8 | 0.0 | 124.1 | 0.1 | 125.4 | 0.0 | | 11.2 | 1.4 |
| Torsion Angles | | | | | | | | | |
| $C^3N^1$*$C^\alpha H^2$ | 52.3 | 0.2 | 82.4 | 0.1 | 0.1 | 0.1 | | 30.1 | -82.3 |
| $C^3N^1$*$C^\alpha H^3$ | 83.6 | 0.2 | 42.0 | 0.1 | 118.4 | 0.1 | | -41.6 | 76.4 |
| $N^1N^2$*$C^\gamma C^\delta$ | 45.3 | 39.5 | 45.5 | 30.5 | 34.1 | 25.6 | | 0.2 | -11.4 |
| $N^1N^2$*$C^\gamma C^\varepsilon$ | 134.5 | 39.8 | 134.3 | 30.9 | 145.9 | 25.9 | | -0.2 | 11.6 |

Table S33. Summary of average bond angles and distances of triazolylidenes **7'a-k** (n=1); **8'a-c, i**, **k** (n=2); **9'a-c**, **k** (n=3) and corresponding standard deviations obtained from DFT calculation (M062X). Data highlighted in purple is included in Table 3 in the main text.

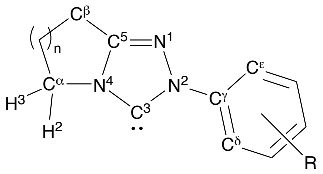


| Backbone Bond Length | Average (n=1) | SD (n=1) | Average (n=2) | SD (n=2) | Average (n=3) | SD (n=3) | Differences of averaged values | n=1 vs n=2 | n=2 vs n=3 |
|---|---|---|---|---|---|---|---|---|---|
| $N^1N^2$ | 1.39 | 0.00 | 1.38 | 0.00 | 1.38 | 0.00 | | -0.01 | 0 |
| $N^2C^3$ | 1.36 | 0.00 | 1.35 | 0.00 | 1.35 | 0.00 | | -0.01 | 0 |
| $C^3N^4$ | 1.36 | 0.00 | 1.36 | 0.00 | 1.36 | 0.00 | | 0.01 | 0 |
| $N^4C^5$ | 1.37 | 0.00 | 1.38 | 0.00 | 1.38 | 0.00 | | 0.01 | 0 |
| $C^5N^1$ | 1.29 | 0.00 | 1.30 | 0.00 | 1.30 | 0.00 | | 0 | 0 |
| $C^5C^{\beta}$ | 1.49 | 0.00 | 1.49 | 0.00 | 1.49 | 0.00 | | 0 | -0.01 |
| $N^4C^{\alpha}$ | 1.46 | 0.00 | 1.47 | 0.00 | 1.46 | 0.00 | | 0 | 0 |
| $N^2C^{\gamma}$ | 1.42 | 0.01 | 1.42 | 0.01 | 1.42 | 0.01 | | 0 | 0 |
| $C^{\gamma}C^{\delta}$ | 1.39 | 0.00 | 1.39 | 0.00 | 1.39 | 0.00 | | 0 | 0 |
| $C^{\gamma}C^{\varepsilon}$ | 1.39 | 0.00 | 1.39 | 0.00 | 1.39 | 0.00 | | 0 | 0 |
| C-H Bond Length | | | | | | | | | |
| $C^{\alpha}H^2$ | 1.09 | 0.00 | 1.09 | 0.00 | 1.09 | 0.00 | | 0.01 | 0.00 |
| $C^{\alpha}H^3$ | 1.09 | 0.00 | 1.09 | 0.00 | 1.09 | 0.00 | | 0.00 | 0.00 |
| C-H Distance | | | | | | | | | |
| $C^3H^2$ | 2.92 | 0.00 | 2.69 | 0.00 | 2.53 | 0.00 | | -0.22 | -0.16 |
| $C^3H^3$ | 3.05 | 0.00 | 2.91 | 0.00 | 3.18 | 0.00 | | -0.14 | 0.28 |
| Bond Angles | | | | | | | | | |
| $C^5N^1N^2$ | 102.1 | 0.2 | 103.2 | 0.3 | 103.3 | 0.3 | | 1.1 | 0.2 |
| $N^1N^2C^3$ | 115.9 | 0.3 | 115.4 | 0.3 | 115.3 | 0.3 | | -0.5 | -0.1 |
| $N^2C^3N^4$ | 99.6 | 0.2 | 100.5 | 0.2 | 100.6 | 0.3 | | 0.9 | 0.1 |
| $C^3N^4C^5$ | 111.6 | 0.1 | 110.8 | 0.2 | 110.8 | 0.1 | | -0.8 | 0 |
| $N^4C^5N^1$ | 110.9 | 0.1 | 110.1 | 0.1 | 110.0 | 0.1 | | -0.8 | -0.1 |
| $C^{\beta}C^5N^4$ | 111.1 | 0.0 | 122.9 | 0.0 | 124.5 | 0.1 | | 11.9 | 1.6 |
| $C^5N^4C^{\alpha}$ | 112.7 | 0.1 | 124.1 | 0.1 | 124.7 | 0.1 | | 11.5 | 0.5 |
| Torsion Angles | | | | | | | | | |
| $N^1\ C^3{*}C^{\alpha}H^2$ | 50.6 | 0.1 | 42.1 | 0.3 | 2.9 | 0.3 | | -8.5 | -39.2 |
| $N^1C^3{*}C^{\alpha}H^3$ | 85.4 | 0.1 | 82.6 | 0.2 | 121.5 | 0.3 | | -2.7 | 38.8 |
| $N^1N^2{*}C^{\gamma}C^{\delta}$ | 44.7 | 37.1 | 43.7 | 28.3 | 30.9 | 21.7 | | -1.0 | -12.9 |
| $N^1N^2{*}C^{\gamma}C^{\varepsilon}$ | 135.1 | 37.4 | 136.1 | 28.8 | 149.1 | 22.1 | | 1.0 | 13.0 |

Table S34. Summary of average* bond angles and distances of triazolylidenes **7'a-c, k** (n=1); **8'a-c, k** (n=2); **9'a-c**, **k** (n=3) and corresponding standard deviations (SD) obtained from obtained from DFT calculation (M062X).

*Averages calculated using only data for the 12 triazolylidenes related to C(3)-H/D exchange studies. These average values and trends are in excellent agreement with data in Table S32 as expected given the small standard deviations.

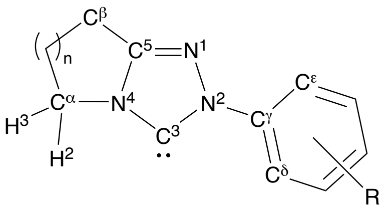


| Backbone Bond Length | Average (n=1) | SD (n=1) | Average (n=2) | SD (n=2) | Average (n=3) | SD (n=3) |
|---|---|---|---|---|---|---|
| $N^1N^2$ | 1.39 | 0.00 | 1.38 | 0.00 | 1.38 | 0.00 |
| $N^2C^3$ | 1.36 | 0.00 | 1.35 | 0.00 | 1.35 | 0.00 |
| $C^3N^4$ | 1.36 | 0.00 | 1.36 | 0.00 | 1.36 | 0.00 |
| $N^4C^5$ | 1.37 | 0.00 | 1.38 | 0.00 | 1.38 | 0.00 |
| $C^5N^1$ | 1.29 | 0.00 | 1.30 | 0.00 | 1.30 | 0.00 |
| $C^5C^\beta$ | 1.49 | 0.00 | 1.49 | 0.00 | 1.49 | 0.00 |
| $N^4C^\alpha$ | 1.46 | 0.00 | 1.47 | 0.00 | 1.46 | 0.00 |
| $N^2C^\gamma$ | 1.42 | 0.01 | 1.42 | 0.01 | 1.42 | 0.01 |
| $C^\gamma C^\delta$ | 1.39 | 0.00 | 1.39 | 0.00 | 1.39 | 0.00 |
| $C^\gamma C^\varepsilon$ | 1.39 | 0.00 | 1.39 | 0.00 | 1.39 | 0.00 |
| C-H Bond Length | | | | | | |
| $C^\alpha H^2$ | 1.09 | 0.00 | 1.09 | 0.00 | 1.09 | 0.00 |
| $C^\alpha H^3$ | 1.09 | 0.00 | 1.09 | 0.00 | 1.09 | 0.00 |
| C-H Distance | | | | | | |
| $C^3H^2$ | 2.91 | 0.00 | 2.69 | 0.00 | 2.53 | 0.00 |
| $C^3H^3$ | 3.05 | 0.00 | 2.90 | 0.00 | 3.18 | 0.00 |
| Bond Angles | | | | | | |
| $C^5N^1N^2$ | 102.1 | 0.3 | 103.2 | 0.3 | 103.3 | 0.3 |
| $N^1N^2C^3$ | 115.9 | 0.3 | 115.4 | 0.3 | 115.3 | 0.3 |
| $N^2C^3N^4$ | 99.6 | 0.2 | 100.5 | 0.3 | 100.6 | 0.3 |
| $C^3N^4C^5$ | 111.6 | 0.1 | 110.8 | 0.1 | 110.8 | 0.1 |
| $N^4C^5N^1$ | 110.9 | 0.1 | 110.1 | 0.1 | 110.0 | 0.1 |
| $C^\beta C^5N^4$ | 111.1 | 0.0 | 122.9 | 0.0 | 124.5 | 0.1 |
| $C^5N^4C^\alpha$ | 112.6 | 0.1 | 124.1 | 0.1 | 124.7 | 0.1 |

| Torsion Angles | | | | | | |
|---|---|---|---|---|---|---|
| $C^3N^1{*}C^{\alpha}H^2$ | 50.6 | 0.1 | 42.1 | 0.3 | 2.9 | 0.3 |
| $C^3N^1{*}C^{\alpha}H^3$ | 85.4 | 0.2 | 82.6 | 0.2 | 121.5 | 0.3 |
| $N^1N^2{*}C^{\gamma}C^{\delta}$ | 32.4 | 18.7 | 33.6 | 19.6 | 30.9 | 21.7 |
| $N^1N^2{*}C^{\gamma}C^{\varepsilon}$ | 147.6 | 18.8 | 146.4 | 19.7 | 149.1 | 22.1 |

## S2.5 Computational Analysis of Conformational Changes in Fused Ring

By fixing the torsion angle between $H^2C^\alpha$ relative to $C^3H^1$ for **7-9b** and between $H^2C^\alpha$ relative to $C^3N^1$ for **7'-9'b**, the energy increments caused by conformational changes in the fused ring in the vicinity of the carbenic position could be evaluated.

Table S35. Energy and conformer distribution of triazolium **7b** obtained from DFT calculation (M062X).

| Torsion Angle (°) | $E_i$ (a.u.)[b] | Torsion Angle Difference (°)[c] | $E_{diff}$ (a.u.)[d] | Populational Ratio (%)[e] |
|---|---|---|---|---|
| 26.0 | -590.384464 | -12.0 | -0.000843 | 26.10 |
| 28.0 | -590.384718 | -10.0 | -0.000589 | 39.12 |
| 30.0 | -590.384926 | -8.0 | -0.000381 | 54.49 |
| 32.0 | -590.385089 | -6.0 | -0.000218 | 70.65 |
| 34.0 | -590.385207 | -4.0 | -1E-04 | 85.27 |
| 36.0 | -590.385279 | -2.0 | -2.8E-05 | 95.64 |
| 38.0[a] | -590.385307 | 0.0 | 0 | 100.00 |
| 40.0 | -590.38529 | 2.0 | -1.7E-05 | 97.33 |
| 42.0 | -590.385232 | 4.0 | -7.5E-05 | 88.73 |
| 44.0 | -590.385136 | 6.0 | -0.000171 | 76.15 |
| 46.0 | -590.385006 | 8.0 | -0.000301 | 61.90 |
| 48.0 | -590.384845 | 10.0 | -0.000462 | 47.89 |
| 50.0 | -590.384654 | 12.0 | -0.000653 | 35.32 |
| 52.0 | -590.384438 | 14.0 | -0.000869 | 25.04 |

[a]Reference taken as torsion angle associated with minimum energy value. [b]Energy for each individual conformation obtained from DFT calculation (M062X). [c]Angle Difference = Torsion angle – Reference. [d]$E_{diff} = E_i - E_{ref}$. [d]Populational ratio calculated using Boltzmann distribution.

Table S36. Energy and conformer distribution of triazolium **8b** obtained from DFT calculation (M062X).

| Torsion Angle (°) | $E_i$ (a.u.)[b] | Torsion Angle Difference (°)[c] | $E_{diff}$ (a.u.)[d] | Populational Ratio (%)[e] |
|---|---|---|---|---|
| 26.0 | -629.702606 | -13.4 | -0.000808 | 27.59 |
| 28.0 | -629.702826 | -11.4 | -0.000588 | 39.18 |
| 30.0 | -629.703013 | -9.4 | -0.000401 | 52.78 |
| 32.0 | -629.703165 | -7.4 | -0.000249 | 67.25 |
| 34.0 | -629.703282 | -5.4 | -0.000132 | 81.03 |
| 36.0 | -629.703362 | -3.4 | -5.2E-05 | 92.05 |
| 38.0 | -629.703405 | -1.4 | -9E-06 | 98.58 |
| 39.4[a] | -629.703414 | 0.0 | 0 | 100.00 |
| 40.0 | -629.703412 | 0.6 | -2E-06 | 99.68 |
| 42.0 | -629.703383 | 2.6 | -3.1E-05 | 95.18 |
| 44.0 | -629.703317 | 4.6 | -9.7E-05 | 85.68 |
| 46.0 | -629.703215 | 6.6 | -0.000199 | 72.82 |
| 48.0 | -629.703081 | 8.6 | -0.000333 | 58.82 |
| 50.0 | -629.702913 | 10.6 | -0.000501 | 45.00 |
| 52.0 | -629.702717 | 12.6 | -0.000697 | 32.93 |

[a]Reference taken as torsion angle associated with minimum energy value. [b]Energy for each individual conformation obtained from DFT calculation (M062X). [c]Angle Difference = Torsion angle – Reference. [d]$E_{diff}$ = $E_i$ – $E_{ref}$. [d]Populational ratio calculated using Boltzmann distribution.

Table S37. Energy and conformer distribution of triazolium **9b** obtained from DFT calculation (M062X).

| Torsion Angle (°) | $E_i$ (a.u.)[b] | Torsion Angle Difference (°)[c] | $E_{diff}$ (a.u.)[d] | Populational Ratio (%)[e] |
|---|---|---|---|---|
| -14.0 | -669.007195 | -14.8 | -0.000935 | 22.54 |
| -12.0 | -669.007425 | -12.8 | -0.000705 | 32.51 |
| -10.0 | -669.007622 | -10.8 | -0.000508 | 44.51 |
| -8.0 | -669.007789 | -8.8 | -0.000341 | 58.08 |
| -6.0 | -669.007923 | -6.8 | -0.000207 | 71.90 |
| -4.0 | -669.008025 | -4.8 | -0.000105 | 84.59 |
| -2.0 | -669.008094 | -2.8 | -3.6E-05 | 94.42 |
| 0.0[a] | -669.008127 | -0.8 | -3E-06 | 99.52 |
| 0.8 | -669.00813 | 0.0 | 0 | 100.00 |
| 2.0 | -669.008122 | 1.2 | -8E-06 | 98.73 |
| 4.0 | -669.008076 | 3.2 | -5.4E-05 | 91.75 |
| 6.0 | -669.007987 | 5.2 | -0.000143 | 79.62 |
| 8.0 | -669.007856 | 7.2 | -0.000274 | 64.62 |
| 10.0 | -669.007684 | 9.2 | -0.000446 | 49.13 |
| 12.0 | -669.007469 | 11.2 | -0.000661 | 34.88 |

[a]Reference taken as torsion angle associated with minimum energy value. [b]Energy for each individual conformation obtained from DFT calculation (M062X). [c]Angle Difference = Torsion angle – Reference. [d]$E_{diff}$ = $E_i$ – $E_{ref}$. [d]Populational ratio calculated using Boltzmann distribution.

Table S38. Energy and conformer distribution of carbene **7'b** obtained from DFT calculation (M062X).

| Torsion Angle (°) | $E_i$ (a.u.)[b] | Torsion Angle Difference (°)[c] | $E_{diff}$ (a.u.)[d] | Populational Ratio (%)[e] |
|---|---|---|---|---|
| 26.0 | -589.912786 | -15.5 | -0.001309 | 12.42 |
| 28.0 | -589.913112 | -13.5 | -0.000983 | 20.88 |
| 30.0 | -589.91339 | -11.5 | -0.000705 | 32.51 |
| 32.0 | -589.913621 | -9.5 | -0.000474 | 46.98 |
| 34.0 | -589.913805 | -7.5 | -0.00029 | 62.99 |
| 36.0 | -589.913942 | -5.5 | -0.000153 | 78.36 |
| 38.0 | -589.914034 | -3.5 | -6.1E-05 | 90.74 |
| 40.0 | -589.914084 | -1.5 | -1.1E-05 | 98.26 |
| 41.5[a] | -589.914095 | 0.0 | 0 | 100.00 |
| 42.0 | -589.914094 | 0.5 | -1E-06 | 99.84 |
| 44.0 | -589.914065 | 2.5 | -3E-05 | 95.33 |
| 46.0 | -589.913998 | 4.5 | -9.7E-05 | 85.68 |
| 48.0 | -589.913894 | 6.5 | -0.000201 | 72.59 |
| 50.0 | -589.913754 | 8.5 | -0.000341 | 58.08 |
| 52.0 | -589.913579 | 10.5 | -0.000516 | 43.94 |

[a]Reference taken as torsion angle associated with minimum energy value. [b]Energy for each individual conformation obtained from DFT calculation (M062X). [c]Angle Difference = Torsion angle – Reference. [d]$E_{diff}$ = $E_i – E_{ref}$. [d]Populational ratio calculated using Boltzmann distribution.

Table S39. Energy and conformer distribution of carbene **8’b** obtained from DFT calculation (M062X).

| Torsion Angle (°) | $E_i$ (a.u.)[b] | Torsion Angle Difference (°)[c] | $E_{diff}$ (a.u.)[d] | Populational Ratio (%)[e] |
|---|---|---|---|---|
| 22.0 | -629.230307 | -14.3 | -0.000862 | 25.32 |
| 24.0 | -629.230528 | -12.3 | -0.000641 | 36.01 |
| 26.0 | -629.230717 | -10.3 | -0.000452 | 48.66 |
| 28.0 | -629.230875 | -8.3 | -0.000294 | 62.59 |
| 30.0 | -629.231 | -6.3 | -0.000169 | 76.39 |
| 32.0 | -629.231091 | -4.3 | -7.8E-05 | 88.31 |
| 34.0 | -629.231147 | -2.3 | -2.2E-05 | 96.55 |
| 36.0 | -629.231169 | -0.3 | 0 | 100.00 |
| 36.3 | -629.231169 | 0.0 | 0 | 100.00 |
| 38.0 | -629.231157 | 1.7 | -1.2E-05 | 98.11 |
| 40.0 | -629.231113 | 3.7 | -5.6E-05 | 91.46 |
| 42.0 | -629.231037 | 5.7 | -0.000132 | 81.03 |
| 44.0 | -629.230933 | 7.7 | -0.000236 | 68.65 |
| 46.0 | -629.230802 | 9.7 | -0.000367 | 55.72 |
| 48.0 | -629.230644 | 11.7 | -0.000525 | 43.32 |

[a]Reference taken as torsion angle associated with minimum energy value. [b]Energy for each individual conformation obtained from DFT calculation (M062X). [c]Angle Difference = Torsion angle – Reference. [d]$E_{diff}$ = $E_i$ – $E_{ref}$. [d]Populational ratio calculated using Boltzmann distribution.

Table S40. Energy and conformer distribution of carbene **9’b** obtained from DFT calculation (M062X).

| Torsion Angle (°) | $E_i$ (a.u.)[b] | Torsion Angle Difference (°)[c] | $E_{diff}$ (a.u.)[d] | Populational Ratio (%)[e] |
|---|---|---|---|---|
| -12.0 | -668.535105 | -14.3 | -0.000942 | 22.29 |
| -10.0 | -668.535348 | -12.3 | -0.000699 | 32.83 |
| -8.0 | -668.535556 | -10.3 | -0.000491 | 45.73 |
| -6.0 | -668.535729 | -8.3 | -0.000318 | 60.24 |
| -2.0 | -668.535966 | -4.3 | -8.1E-05 | 87.89 |
| 0.0 | -668.536026 | -2.3 | -2.1E-05 | 96.71 |
| 2.3 | -668.536047 | -1.7 | 0 | 100.00 |
| 4.0 | -668.53603 | 1.7 | -1.7E-05 | 97.33 |
| 6.0 | -668.535974 | 3.7 | -7.3E-05 | 89.02 |
| 8.0 | -668.535878 | 5.7 | -0.000169 | 76.39 |
| 10.0 | -668.535742 | 7.7 | -0.000305 | 61.50 |
| 12.0 | -668.535564 | 9.7 | -0.000483 | 46.31 |
| 14.0 | -668.535343 | 11.7 | -0.000704 | 32.57 |

[a]Reference taken as torsion angle associated with minimum energy value. [b]Energy for each individual conformation obtained from DFT calculation (M062X). [c]Angle Difference = Torsion angle – Reference. [d]$E_{diff}$ = $E_i$ – $E_{ref}$. [d]Populational ratio calculated using Boltzmann distribution.

Figure S51. Plots of populational ratio of triazolium salts **7-9b** against torsion angle between $C^{\alpha}H^{2}$ relative to $C^{3}H^{1}$.

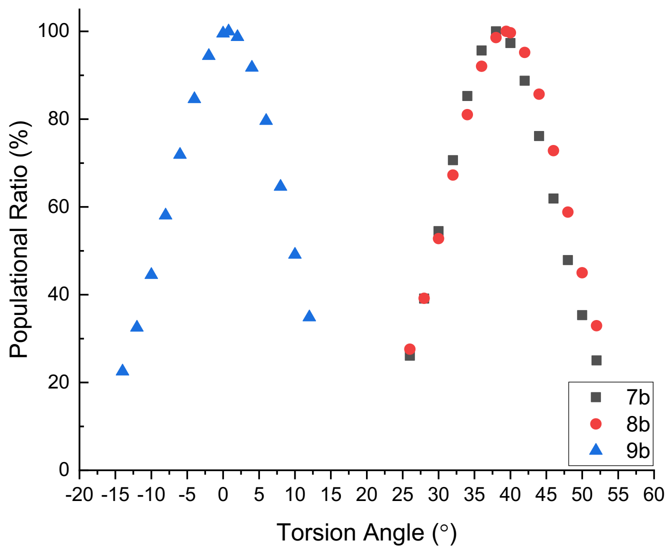


Figure S52. Plots of populational ratio of carbenes **7'-9'b** against torsion angle between $C^{\alpha}H^{2}$ relative to $C^{3}N^{1}$.

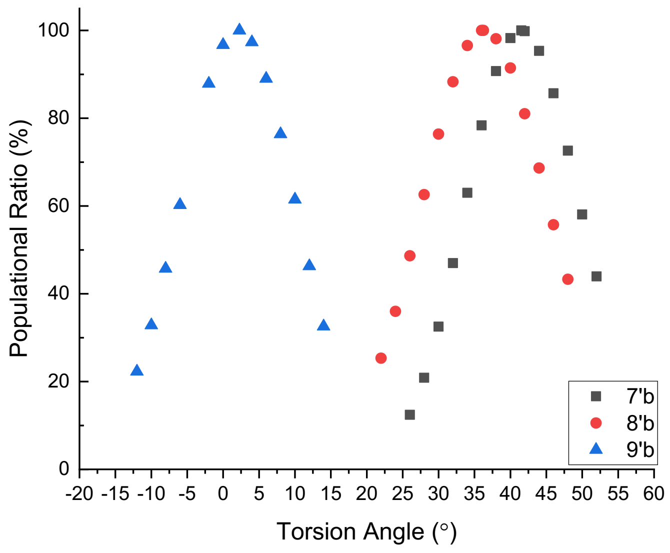

Figure S53. Superimposed populational ratio of triazolium salts **7-9b** and carbenes **7’-9’b** against torsion angle differences using the data from Tables S35-S40.

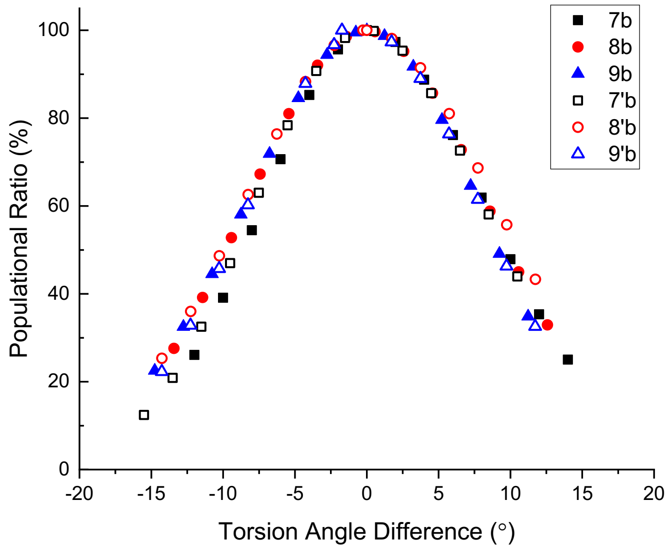


## S2.6 NBO analysis

For the *para*-fluorophenyl triazolium ion **7c** (n=1), NBO analyses suggest that the C(3)-H bond contains 38.9% s-character and 61.0% *p*-character. The s-character occupancy changed to 38.7% in both **8c** and **9c**, and the *p*-character occupancy changed to 61.2% in both cases.

## S3. References


(S1) (a) Kerr, M. S.; Read de Alaniz, J.; Rovis, T. An efficient synthesis of achiral and chiral 1,2,4-triazolium salts: Bench stable precursors for N-heterocyclic carbenes. *J. Org. Chem.* **2005**, *70*, 5725. (b) Langdon, S. M.; Wilde, M. M.; Thai, K.; Gravel, M. Chemoselective N-heterocyclic carbene-catalyzed cross-benzoin reactions: Importance of the fused ring in triazolium salts. *J. Am. Chem. Soc.* **2014**, *136*, 7539.(c) Samanta, R. C.; Maji, B.; De Sarkar, S.; Bergander, K.; Frohlich, R.; Muck-Lichtenfeld, C.; Mayr, H.; Studer, A. Nucleophilic addition of enols and enamines to α, β-unsaturated acyl azoliums: Mechanistic studies. *Angew. Chem., Int. Ed.* **2012**, *51*, 5234. (d) Ling, K. B.; Smith, A. D. α-Aroyloxyaldehydes: scope and limitations as alternatives to α-haloaldehydes for NHC-catalysed redox transformations. *Chem. Commun.* **2011**, *47*, 373. (e) Suenaga, K.; Shimogawa, H.; Nakagawa, S.; Uemura, D. Catharsitoxins from the Chinese remedy qiung laug. *Tetrahedron Lett.* **2001**, *42*, 7079. (f) Chiang, P.-C.; Kim, Y.; Bode, J. W. Catalytic amide formation with α′-hydroxyenones as acylating reagents. *Chem. Commun.* **2009**, 4566. (g) Davidson, R. W.; Fuchter, M. J. Direct NHC-catalysed redox amidation using $CO_2$ for traceless masking of amine nucleophiles. *Chem. Commun.* **2016**, *52*, 11638. (h) Thomson, J. E.; Campbell, C. D.; Concellon, C.; Duguet, N.; Rix, K.; Slawin, A. M.; Smith, A. D. Probing the efficiency of N-heterocyclic carbene promoted O- to C-carboxyl transfer of oxazolyl carbonates. *J. Org. Chem.* **2008**, *73*, 2784. (i) Thomson, J. E.; Rix, K.; Smith, A. D. Efficient N-heterocyclic carbene-catalyzed O- to C-acyl transfer. *Org. Lett.* **2006**, *8*, 3785. (j) Li, Z.; Li, X.; Cheng, J.-P. An acidity scale of triazolium-based NHC precursors in DMSO. *J. Org. Chem.* **2017**, *82*, 9675. (h) Schedler, M.; Fröhlich, R.; Daniliuc, C. G.; Glorius, F. 2, 6-dimethoxyphenyl-substituted N-heterocyclic carbenes (NHCs): A family of highly electron-rich organocatalysts. *Eur. J. Org. Chem.* **2012**, *22*, 4164. (g) Campbell, C. D.; Collett, C. J.; Thomson, J. E.; Slawin, A. M.; Smith, A. D. Organic base effects in NHC promoted O-to C-carboxyl transfer; chemoselectivity profiles, mechanistic studies and domino catalysis. *Org. Biomol. Chem.* **2011**, *9*, 4205.

(S2) Glasoe, P. K.; Long, F. Use of glass electrodes to measure acidities in deuterium oxide[1,2]. *J. Phys. Chem.* **1960**, *64*, 188.

(S3) Covington, A.; Robinson, R.; Bates, R. G. Ionization constant of deuterium oxide from 5 to 50 deg. *J. Phys. Chem.* **1966**, *70*, 3820.

(S4) Massey, R. S.; Collett, C. J.; Lindsay, A. G.; Smith, A. D.; O'Donoghue, A. C. Proton transfer reactions of triazol-3-ylidenes: Kinetic acidities and carbon acid $pK_a$ values for twenty triazolium salts in aqueous solution. *J. Am. Chem. Soc.* **2012**, *134*, 20421.

(S5) (a) Tucker, D. E.; Quinn, P.; Massey, R. S.; Collett, C. J.; Jasiewicz, D. J.; Bramley, C. R.; Smith, A. D.; O'Donoghue, A. C. Proton transfer reactions of N-aryl triazolium salts: unusual *ortho*-substituent effects. *J. Phys. Org. Chem.* **2015**, *28*, 108. (b) Quinn, P.; Smith, M. S.; Zhu, J.; Hodgson, D. R.; O'Donoghue, A. C. Triazolium salt organocatalysis: mechanistic evaluation of unusual *ortho*-substituent effects on deprotonation. *Catalysts* **2021**, *11*, 1055.

(S6) Hansch, C.; Leo, A.; Taft, R. A survey of Hammett substituent constants and resonance and field parameters. *Chem. Rev.* **1991**, *91*, 165.

(S7) Charton, M. The application of the Hammett equation to *ortho*-substituted benzene reaction series. *Can. J. Chem.* **1960**, *38*, 2493.

(S8) Korenaga, T.; Kadowaki, K.; Ema, T.; Sakai, T. Reestimation of the Taft's substituent constant of the pentafluorophenyl group. *J. Org. Chem.* **2004**, *69*, 7340.

(S9) Tyler, A. R.; Ragbirsingh, R.; McMonagle, C. J.; Waddell, P. G.; Heaps, S. E.; Steed, J. W.; Thaw, P.; Hall, M. J.; Probert, M. R. Encapsulated nanodroplet crystallization of organic-soluble small molecules. *Chem.* **2020**, *6*, 1755.

(S10) Frisch, M. J.; Trucks, G. W.; Schlegel, H. B.; Scuseria, G. E.; Robb, M. A.; Cheeseman, J. R.; Scalmani, G.; Barone, V.; Petersson, G. A.; Nakatsuji, H.; Li, X.; Caricato, M.; Marenich, A. V.; Bloino, J.; Janesko, B. G.; Gomperts, R.; Mennucci, B.; Hratchian, H. P.; Ortiz, J. V.; Izmaylov, A. F.; Sonnenberg, J. L.; Williams-Young, D.; Ding, F.; Lipparini, F.; Egidi, F.; Goings, J.; Peng, B.; Petrone, A.; Henderson, T.; Ranasinghe, D.; Zakrzewski, V. G.; Gao, J.; Rega, N.; Zheng, G.; Liang, W.; Hada, M.; Ehara, M.; Toyota, K.; Fukuda, R.; Hasegawa, J.; Ishida, M.; Nakajima, T.; Honda, Y.; Kitao, O.; Nakai, H.; Vreven, T.; Throssell, K.; Montgomery, J. A., Jr.; Peralta, J. E.; Ogliaro, F.; Bearpark, M. J.; Heyd, J. J.; Brothers, E. N.; Kudin, K. N.; Staroverov, V. N.; Keith, T. A.; Kobayashi, R.; Normand, J.; Raghavachari, K.; Rendell, A. P.; Burant, J. C.; Iyengar, S. S.; Tomasi, J.; Cossi, M.; Millam, J. M.; Klene, M.; Adamo, C.; Cammi, R.; Ochterski, J. W.; Martin, R. L.; Morokuma, K.; Farkas, O.; Foresman, J. B.; Fox, D. J. *Inc., Wallingford CT* **2016**.

(S11) (a) Lee, C.; Yang, W.; Parr, R. G. Development of the Colle-Salvetti correlation-energy formula into a functional of the electron density. *Phys. Rev. B* **1988**, *37*, 785. (b) Clark, T.; Chandrasekhar, J.; Spitznagel, G. W.; Schleyer, P. V. R. Efficient diffuse function-augmented basis sets for anion calculations. III. The 3-21+ G basis set for first-row elements, Li–F. *J. Comput. Chem.* **1983**, *4*, 294. (c) Krishnan, R.; Binkley, J. S.; Seeger, R.; Pople, J. A. Self-consistent molecular orbital methods. XX. A basis set for correlated wave functions. *J. Phys. Chem.* **1980**, *72*, 650.(d) Tomasi, J.; Mennucci, B.; Cammi, R. Quantum mechanical continuum solvation models. *Chem. Rev.* **2005**, *105*, 2999. (e) Zhao, Y.; Truhlar, D. G. The M06 suite of density functionals for main group thermochemistry, thermochemical kinetics, noncovalent interactions, excited states, and transition elements: two new functionals and systematic testing of four M06-class functionals and 12 other functionals. *Theor. Chem. Acc.* **2008**, *120*, 215. (f) Wang, Y.; Wei, D.; Zhang, W.; *ChemCatChem*. **2018**, *10*, 338.

(S12) Weinhold, F.; Carpenter, in *The structure of small molecules and ions*; Naaman, R.; Vager, Z. Plenum New York. **1988**.
